# Supplementary material for: Experiences of ‘traditional’ and ‘one-stop’ MRI-based prostate cancer diagnostic pathways in England: a qualitative study with patients and GPs
Source: BMJ Open. 2022 Jul 26;12(7):e054045. doi: 10.1136/bmjopen-2021-054045 (PMC9330318; doi:10.1136/bmjopen-2021-054045)
Supplement: Supplementary data [file bmjopen-2021-054045supp001.pdf]

Patient participant interview guide

Introduction (5mins)
Thank you for agreeing to participate in this interview.

Introduce myself and my role.

This study seeks to understand your knowledge and understanding of diagnostic tests for prostate cancer, and your experiences of the current prostate cancer diagnostic pathway in the region where you live. It is part of my PhD at the University of Exeter. This study has been funded by Cancer Research UK, and has ethical approval from the NHS Health Research Authority and the University of Exeter.

As we've talked about with the consent form, participating in this study is voluntary and you can stop at any time. We want to know about your experiences and what you think, so there are no right or wrong answers.

This interview is being recorded for the purposes of qualitative analysis by the researchers. You can ask for the recording to be stopped at any time. What you say will be kept confidential and anonymous, unless we discuss something that suggests there is a significant risk to yourself or someone else. Everyone being interviewed will be asked the same questions, so if you don't have an answer to any of the questions that's fine, just say so and we can move on.

This interview study if focused on your experience of diagnostic test for prostate cancer. However, if you have a partner, family member or significant other who you wish to be present that's fine. Ideally we would start the interview without them, and then invite them in later on. If you and they are happy for them to participate, they would need to complete a consent form as well.

Ensure participant has copy of participant information sheet

Answer any questions

Ensure consent form is completed correctly

Commence audio recording

Merriel SWD, *et al. BMJ Open* 2022; 12:e054045. doi: 10.1136/bmjopen-2021-054045

Basic demographics (5 minutes)
"To start with, can you tell me a little bit about yourself and your background"
Check - Age, Ethnicity, City/town lived in

Prostate cancer diagnosis journey (5-10 minutes)
"Now I would like to talk a bit about how you came to have tests for possible prostate cancer."
When did you first notice symptoms (if any)? Which symptoms were they?
How long until consulted you consulted your GP? What affected that decision?
How did GP assess? PSA? DRE?
Decision to refer – what do you remember about that discussion?

mpMRI for prostate cancer (10-15 minutes)
"I would now like to ask some questions about having an MRI scan for possible prostate cancer."
Did you have any concerns or reservations about having an MRI? What were your expectations?
What do you understand about how an MRI works?
What was your experience of having an MRI?

mpMRI result (10 minutes)
"If you are happy to discuss, I would like to ask a few questions about the results of your MRI scan and the next steps."
What was the result of your mpMRI? Did you understand it? How much did you trust the findings?
How were the results communicated? Did you have any questions as a result?
What were you told about the results' meaning?
What else did you discuss with the specialist?

Doctor(s) involved in cancer diagnosis (5 minutes)
"Finally, I would like to ask about your thoughts regarding the way we organise testing for prostate cancer."
What works well? What could be done better?
Who do you feel should be involved in investigating for a possible cancer diagnosis?
If you could design the ideal cancer diagnosis testing service, how would it work?

Interview close (2 minutes)
Thank you for participating in this interview. The data you have provided will be transcribed under a pseudonym, and analysed by the research team. You will be sent a final study report after the analysis has been completed.
If you have any questions or concerns about the study, please contact Ms Pam Baxter at the Research Ethics and Governance Office at the University of Exeter on 01392 723588 or via email p.r.baxter2@exeter.ac.uk Her details are on your patient information leaflet.

Merriel SWD, *et al. BMJ Open* 2022; 12:e054045. doi: 10.1136/bmjopen-2021-054045
