# Supplementary material for: Experiences of ‘traditional’ and ‘one-stop’ MRI-based prostate cancer diagnostic pathways in England: a qualitative study with patients and GPs
Source: BMJ Open. 2022 Jul 26;12(7):e054045. doi: 10.1136/bmjopen-2021-054045 (PMC9330318; doi:10.1136/bmjopen-2021-054045)
Supplement: Supplementary data [file bmjopen-2021-054045supp002.pdf]

GP participant interview guide

Introduction (5mins)

Thank you for agreeing to participate in this interview.

Introduce myself and my role.

This study seeks to understand your knowledge and understanding of diagnostic tests for prostate cancer, and your experiences of the current prostate cancer diagnostic pathway in the region where you work. It is part of my PhD at the University of Exeter. This study has been funded by Cancer Research UK, and has ethical approval from the NHS Health Research Authority and the University of Exeter.

As we've talked about with the consent form, participating in this study is voluntary and you can stop at any time. We want to know about your experiences and what you think, so there are no right or wrong answers.

This interview is being recorded for the purposes of qualitative analysis by the researchers. You can ask for the recording to be stopped at any time. What you say will be kept confidential and anonymous, unless we discuss something that suggests there is a significant risk to yourself or someone else. This interview is not assessing your clinical competence, and we want to hear about your approach and experiences. Everyone being interviewed will be asked the same questions, so if you don't have an answer to any of the questions that's fine, just say so and we can move on.

This interview study if focused on your experience of diagnostic test for prostate cancer. However, if you have a partner, family member or significant other who you wish to be present that's fine. Ideally we would start the interview without them, and then invite them in later on. If you and they are happy for them to participate, they would need to complete a consent form as well.

Ensure participant has copy of participant information sheet

Answer any questions

Ensure consent form is completed correctly

Commence audio recording

Merriel SWD, *et al. BMJ Open* 2022; 12:e054045. doi: 10.1136/bmjopen-2021-054045

Basic demographics (5 minutes)
"To start with, can you tell me a little bit about yourself and your background"

Age, Gender, Years of GP experience, Main CCG area you work in

Decision to refer for suspected prostate cancer (10-15 minutes)
"I would like to now move on to your current practice around referring men with suspected prostate cancer for further investigation"

"What symptoms/signs do you enquire about when assessing a man for suspected prostate cancer? How do they affect your decision to refer?"

PSA use – When would you offer it to a man? What are the important points you make about PSA when counselling a man about the test? What do you do with a negative PSA?

"What other factors, if any, affect your decision to refer a man for further investigation?"

"What are the key points you discuss with men when making a referral?"

Diagnostic testing for prostate cancer (15-20 minutes)
"Now I would like to ask some questions about diagnostic tests for prostate cancer."

What is your experience of the prostate cancer diagnosis pathway in your region?
What do you know about current diagnostic tests? How accurate do you believe current diagnostic tests are for prostate cancer?
PROMIS trial – have you heard of it? Are you aware of use of mpMRI for prostate cancer?
Do you feel incorporating mpMRI into the prostate cancer diagnosis pathway would be beneficial for patients? Do you believe it could be cost effective?
What would be the characteristics of an ideal diagnostic test for prostate cancer?

Men diagnosed with prostate cancer (5-10 minutes)
"Finally, I would like to ask about any of your patients who have been diagnosed with prostate cancer"

Are you aware of any of your patients diagnosed with low-grade prostate cancer?
If so, what has been the impact of the diagnosis on patient?
Are you aware of any of your patients being put on active surveillance – what is your experience of interacting with these men after diagnosis?

Interview close (2 minutes)
Thank you for participating in this interview. The data you have provided will be transcribed under a pseudonym, and analysed by the researchers. You will be sent a final study report after the analysis has been completed. If you have any questions or concerns about the study, please contact Ms Pam Baxter at the Research Ethics and Governance Office at the University of Exeter on 01392 723588 or via email p.r.baxter2@exeter.ac.uk

Merriel SWD, *et al. BMJ Open* 2022; 12:e054045. doi: 10.1136/bmjopen-2021-054045
