# Supplementary material for: Experiences of ‘traditional’ and ‘one-stop’ MRI-based prostate cancer diagnostic pathways in England: a qualitative study with patients and GPs
Source: BMJ Open. 2022 Jul 26;12(7):e054045. doi: 10.1136/bmjopen-2021-054045 (PMC9330318; doi:10.1136/bmjopen-2021-054045)
Supplement: Supplementary data [file bmjopen-2021-054045supp003.pdf]

‘Acceptability, understanding and experience of diagnostic tests for prostate cancer: a qualitative study with patients and GPs’

IRAS ID: 259602

Protocol Version Number: 1.0

Protocol date: 21/01/2019

Sponsor: University of Exeter

Sponsor Protocol Reference Number: 1819/03

Chief Investigator: Dr Sam Merriel

Supervisors:

Dr Fiona Walter, University of Cambridge

Dr Alice Forster, University College London

Professor Willie Hamilton, University of Exeter

This protocol has regard for the HRA guidance and order of content

Merriel SWD, *et al. BMJ Open* 2022; 12:e054045. doi: 10.1136/bmjopen-2021-054045

SIGNATURE PAGE

The undersigned confirm that the following protocol has been agreed and accepted and that the Chief Investigator agrees to conduct the study in compliance with the approved protocol and will adhere to the principles outlined in the Declaration of Helsinki, the Sponsor's SOPs, and other regulatory requirement.

I agree to ensure that the confidential information contained in this document will not be used for any other purpose other than the evaluation or conduct of the investigation without the prior written consent of the Sponsor

I also confirm that I will make the findings of the study publically available through publication or other dissemination tools without any unnecessary delay and that an honest accurate and transparent account of the study will be given; and that any discrepancies from the study as planned in this protocol will be explained.

For and on behalf of the Study Sponsor:

Signature:

Date:
30/01/2019

P.R Baxter

Name (please print): Ms Pam Baxter

Position: Senior Research Governance Officer

University of Exeter

Chief Investigator:

Date:
30/01/2019

Signature:

Name: (please print): Dr Sam Merriel

LIST of CONTENTS

GENERAL INFORMATION	Page No.
HRA PROTOCOL COMPLIANCE DECLARATION AND SIGNATURE PAGE	ii
LIST OF CONTENTS	iii
KEY STUDY CONTACTS	iv
STUDY SUMMARY	v
FUNDING	v
ROLE OF SPONSOR AND FUNDER	vi
ROLES & RESPONSIBILITIES OF STUDY STEERING GROUPS AND INDIVIDUALS	vi
**SECTION**	
STUDY FLOW CHARTS	1
1. BACKGROUND	4
2. RATIONALE	4
3. THEORETICAL FRAMEWORK	5
4. RESEARCH QUESTION/AIM(S)	5
5. STUDY DESIGN/METHODS	6
6. SAMPLE AND RECRUITMENT	7
7. ETHICAL AND REGULATORY COMPLIANCE	8
8. DISSEMINATION POLICY	12
9. REFERENCES	12
10. APPENDICES	15

KEY STUDY CONTACTS

Chief Investigator<br>('The researcher')	Dr Sam Merriel<br>Clinical Senior Research Fellow, Exeter Medical School, University of Exeter<br>1.18, College House, St Luke's Campus, University of Exeter, Heavitree Road, Exeter EX1 2LU<br>s.w.d.merriel@exeter.ac.uk<br>01392 726090
Supervisors	Dr Fiona Walter<br>Principal Researcher in Primary Care Cancer Research, Department of Public Health and Primary Care, University of Cambridge<br>The Primary Care Unit, University of Cambridge School of Clinical Medicine, Box 113 Cambridge Biomedical Campus, Cambridge, CB2 0SR<br>fmw22@medschl.cam.ac.uk<br>01223 762514<br>Dr Alice Forster<br>Senior Research Fellow, Institute of Epidemiology & Health, Faculty of Population Health Sciences, University College London<br>1-19 Torrington place, London, WC1E 6BT<br>alice.forster@ucl.ac.uk<br>Professor Willie Hamilton<br>Professor of Primary Care Diagnostics, Exeter Medical School, University of Exeter<br>1.17, College House, St Luke's Campus, University of Exeter, Heavitree Road, Exeter EX1 2LU<br>w.hamilton@exeter.ac.uk<br>01392 726097
Sponsor	University of Exeter<br>Sponsor's representative<br>Ms Pam Baxter<br>Senior Research Governance Officer<br>Research Ethics and Governance Office,<br>University of Exeter<br>Lafrowda House, St Germans Road, Exeter,<br>Devon, EX4 6TL<br>p.r.baxter2@exeter.ac.uk<br>01392 723588

Funder(s)	Cancer Research UK

STUDY SUMMARY

Study Title	'Acceptability, understanding and experience of diagnostic tests for prostate cancer: a qualitative study with patients and GPs'
Internal ref. no. (or short title)	Acceptability of mpMRI for prostate cancer diagnosis in primary care
Study Design	Qualitative interview study
Study Participants	Males who have undergone multiparametric Magnetic Resonance Imaging (mpMRI) for suspected prostate cancer.<br>General Practitioners (GPs)
Planned Size of Sample (if applicable)	Purposive sample of approximately 10 GPs and 20 patients
Planned Study Period	01/03/2019 – 01/07/2020
Research Question/Aim(s)	**Aim**<br>To understand, from the perspective of patients and GPs, the acceptability of multiparametric magnetic resonance imaging for men as a diagnostic test for prostate cancer<br>**Objectives**<br>1. Elicit men's experiences of diagnostic tests for suspected prostate cancer<br>2. Explore the knowledge and understanding of diagnostic tests for suspected prostate cancer amongst patients and GPs<br>3. Understand the acceptability of mpMRI as a diagnostic test for prostate cancer from a patient's perspective<br>4. Understand the acceptability of mpMRI as a diagnostic test for prostate cancer from a GP's perspective

FUNDING AND SUPPORT IN KIND

FUNDER(S)	FINANCIAL AND NON FINANCIALSUPPORT GIVEN

Cancer Research UK	Funding: CanTest (Cancer Research UK Catalyst Award) - Clinical Research Fellow<br>Duration: 3 years 8 months (at 60% FTE)<br>Start date: 02/08/2018<br>Travel costs for researchers and participants to attend face to face interviews<br>Service support costs for recruitment sites<br>Transcription of interviews
National Institute for Health Research Clinical Research Network	Adoption of study onto NIHR portfolio<br>Recruitment of GP participants through CRN practices

ROLES AND RESPONSIBILITIES OF STUDY MANAGEMENT COMMITEES/GROUPS & INDIVIDUALS

The study management group with comprise the researcher, the supervisors, and a PPI representative. The researcher will report to the group, who will oversee the recruitment, data collection and analysis for the duration of the study. The study management group will be independent from the sponsor and the funders.

PROTOCOL CONTRIBUTORS

The study sponsor provided support to the researcher in the preparation of the study protocol, consent forms, patient information leaflets and interview guides. The sponsor will have no role in any other aspect of the conduct, analysis, or dissemination of the study.

The funder had no role in any aspect of the preparation of the study, and will have no role in the conduct, analysis or dissemination of the study.

Patients and members of the public have been involved with writing the lay summary, consent forms, patient information leaflets and interview schedules.

SPONSOR

The study sponsor (the University of Exeter) has ensured that the research team, research protocol and research sites are suitable and that indemnity arrangements are in place. In reviewing the research and ethics documentation, the sponsor has further ensured that appropriate risk management is in place and that the study is managed and conducted in accordance with relevant legislation and codes of good practice. The sponsor will ensure that relevant approvals are in place before the study begins, that the study is conducted in accordance with the protocol and relevant approvals, and that appropriate record-keeping and data management is maintained. The sponsor must approve any study amendments or modifications and will be notified of any significant developments or adverse events in accordance with appropriate guidelines. The sponsor has reviewed plans for data storage and retention and plans for dissemination of the research findings.

KEY WORDS: Prostate cancer; MRI; diagnosis; primary care; acceptability

STUDY FLOW CHART 1 – Patient participant recruitment at Imperial College Healthcare

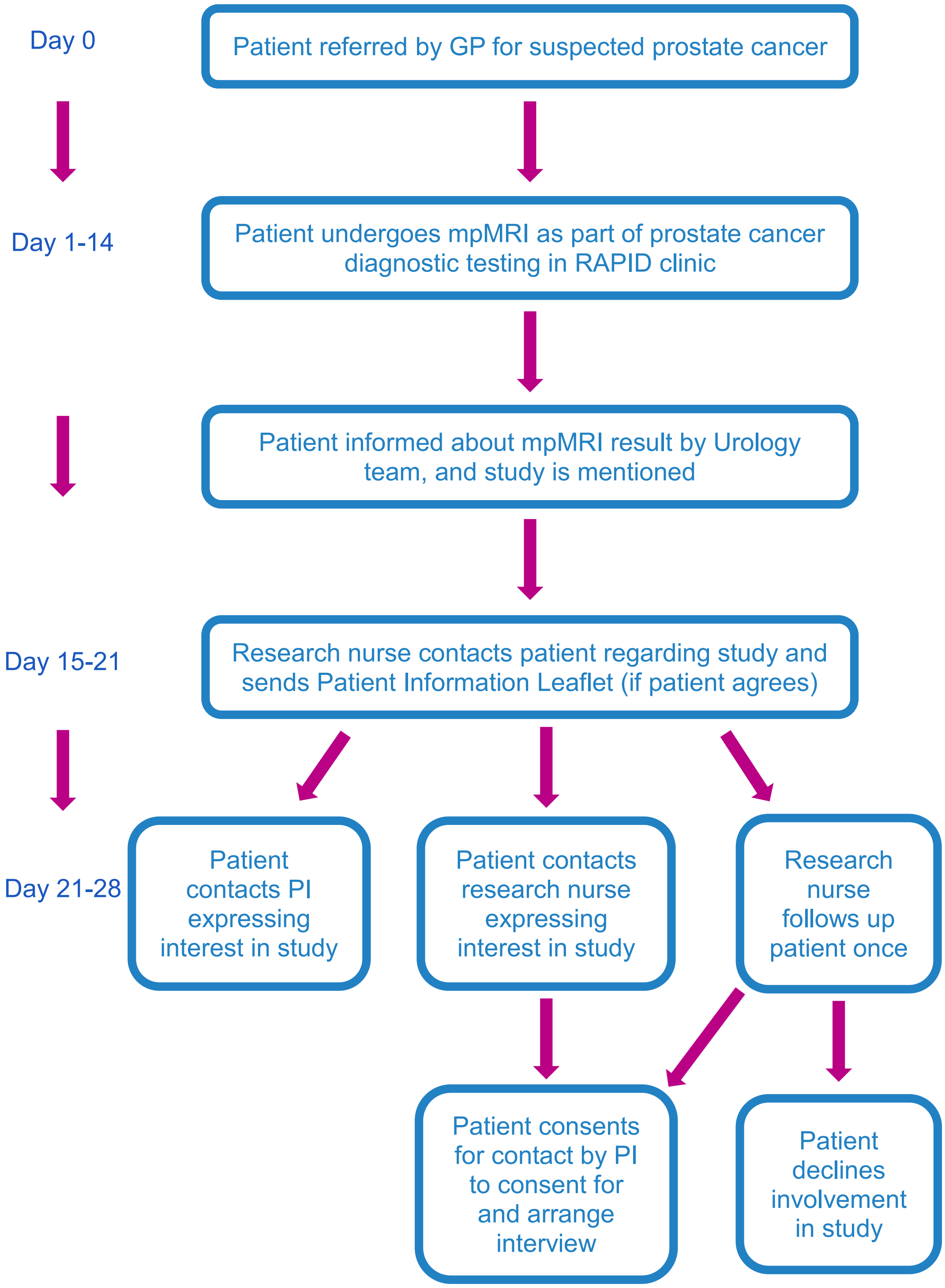

STUDY FLOW CHART 2 – Patient participant recruitment at Royal Devon & Exeter

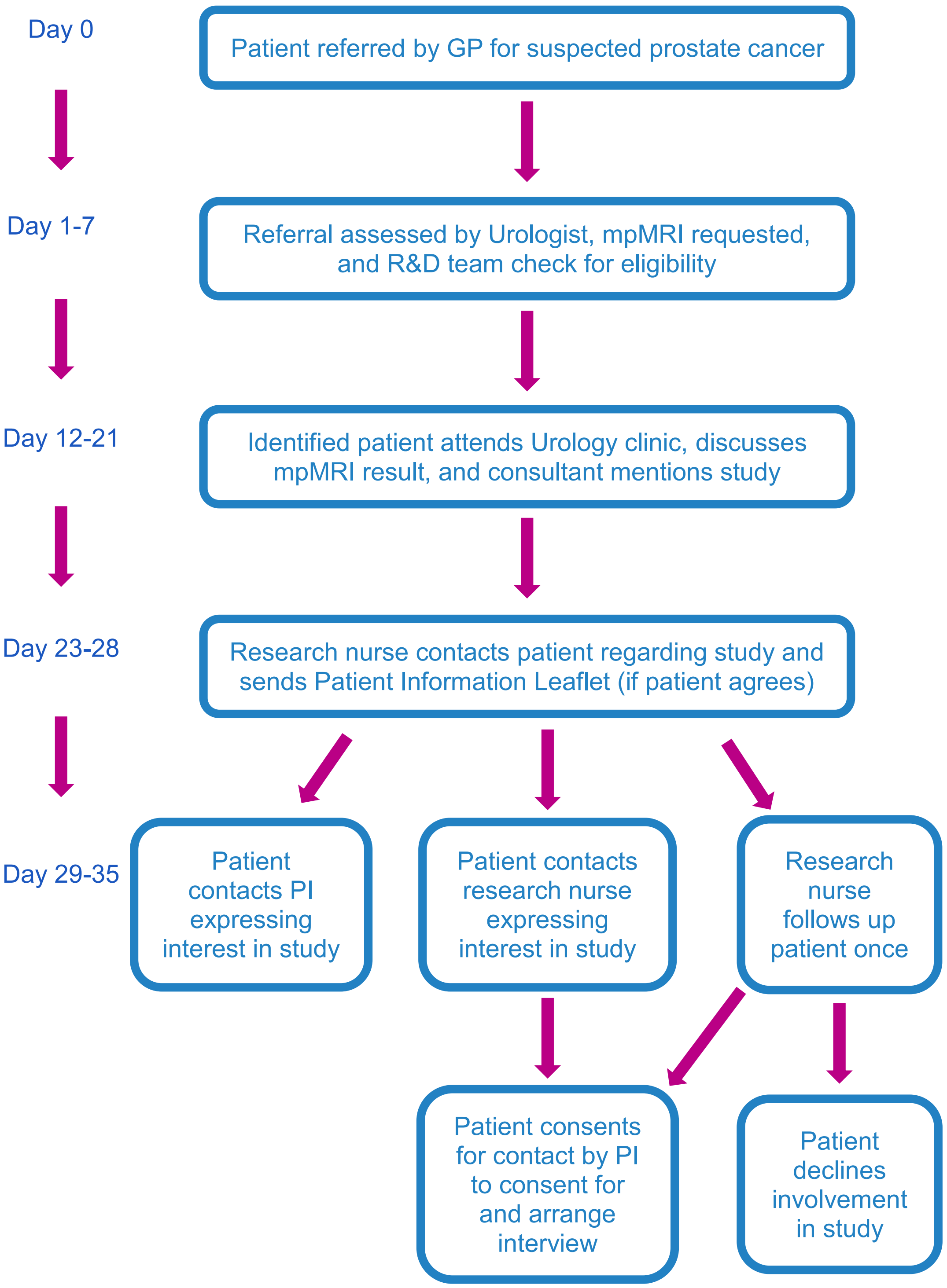

STUDY FLOW CHART 3 – GP participant recruitment via Clinical Research Networks

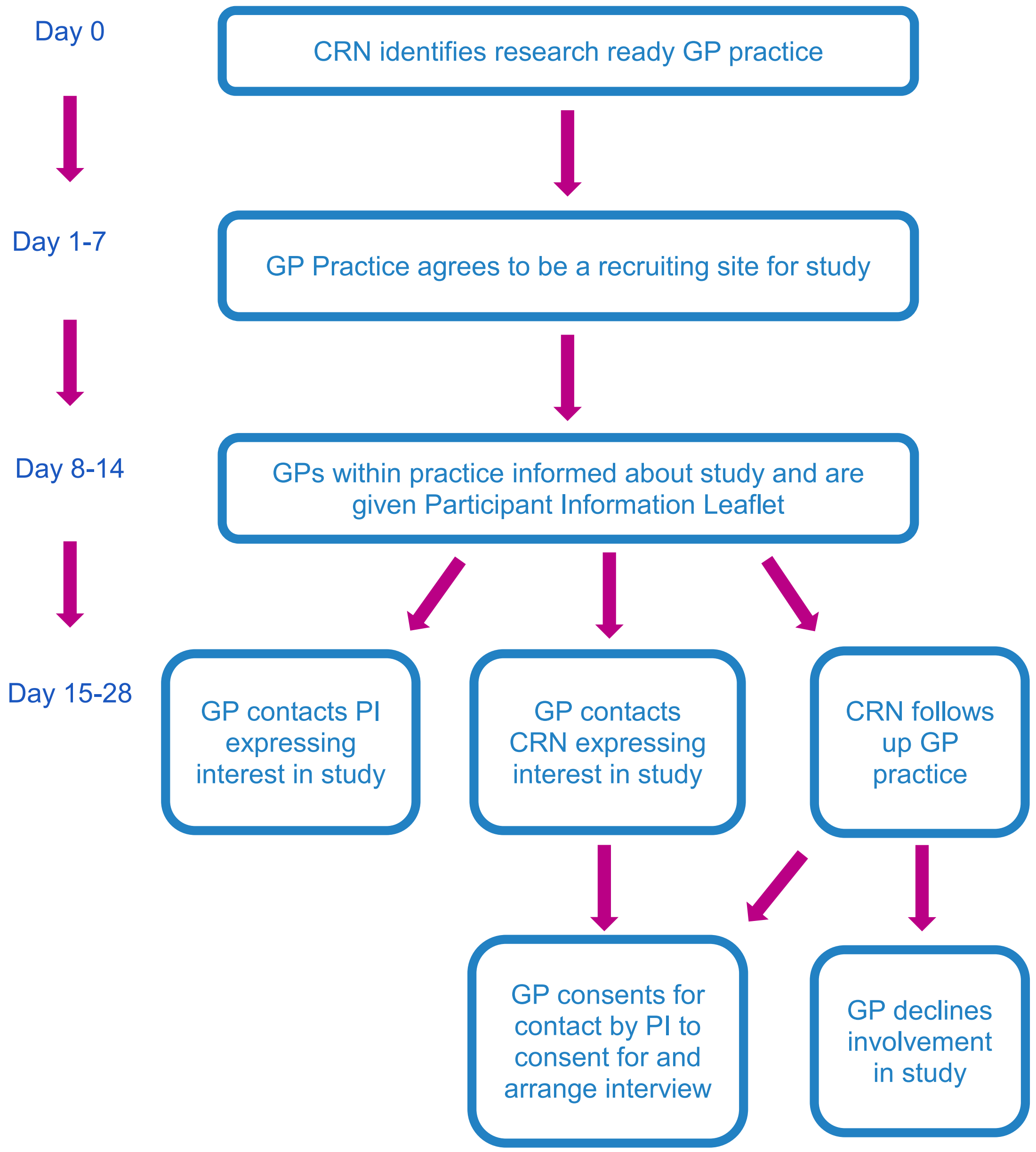

STUDY PROTOCOL

'Acceptability, understanding and experience of diagnostic tests for prostate cancer: a qualitative study with patients and GPs'

1 BACKGROUND

Implementation of diagnostic tests into routine clinical practice should follow a rigorous process of evaluation from showing analytical validity and diagnostic accuracy, through to acceptability and cost effectiveness. A number of frameworks for assessing and evaluating tests for use in healthcare have been proposed(1–5). They suggest the test should be able to be performed by the operator(s); it should demonstrate more patient benefit than harm; it should be cost effective relative to currently available tests; it should be able to be integrated into the diagnostic pathway; and it should be acceptable to patients and clinicians.

Cancer diagnosis pathways in the NHS in the UK involve primary and secondary care clinicians(6). Some diagnostic tests can be ordered through 'direct access' by a patient's General Practitioner (GP) if they present with symptoms or signs that could indicate an undiagnosed cancer. These include gastroscopy for oesophageal or gastric cancer; colonoscopy for bowel cancer; flexible sigmoidoscopy for rectal cancer; and CT or MRI head for brain tumours(6). Diagnostic tests for prostate cancer currently requires a referral to secondary care.

The current gold standard diagnostic test for prostate cancer is a transrectal ultrasound-guided (TRUS) biopsy of the prostate. TRUS biopsy procedures take 6-12 samples from different regions of the prostate, which are then examined by a histopathologist for signs of prostate cancer(7). TRUS biopsy carries a risk of infection and sepsis, and there is a risk of under- or misdiagnosis as a result of the random nature of sampling the prostate(8). Multiparametric MRI (mpMRI) scanning of the prostate, and reporting using the PiRADS(9) reporting system, has recently been compared to TRUS biopsy in recent large, multicentre trials(10,11) with favourable results in terms of diagnostic accuracy. Few studies have been performed assessing other aspects of the implementation of mpMRI for prostate cancer diagnosis, including patient experience and clinician acceptability.

2 RATIONALE

Prostate cancer is the most common malignancy in males in the United Kingdom(UK)(12). Whilst prostate cancer is responsible for a significant number of cancer-related deaths, the 5- and 10-year survival rates for men with prostate cancer is high(13). This has partly been driven by an increase in the numbers of clinically insignificant prostate cancer cases being diagnosed in the last three decades(14). Better diagnostic tests and diagnostic pathways are needed to reduce rates of over-diagnosis of clinically insignificant prostate cancer, and mpMRI may have a role in this. Following on from the PROMIS trial(10) and others like it(11,15,16), NHS England issued guidance for 'Implementing a timed prostate cancer diagnostic pathway' to NHS Cancer Alliances, reinforcing the benefits for patients of integrating mpMRI into local diagnostic pathways(17).

Studies of patient acceptance of TRUS prostate biopsy for prostate cancer, the current diagnostic test, focus on prevalence of side effects and patient anxiety relating to the test(18–20). Two studies to date have assessed patient acceptance of mpMRI guided biopsy, which also involved questionnaires assessing side effects and attitudes towards the test(21,22). There are no studies that examine acceptability of mpMRI as a diagnostic test for prostate cancer with any theoretical underpinning, and questions remain about men's experience of undergoing the test and receiving the results. There are also very few studies of General Practitioners (GPs), or primary care clinicians, exploring their understanding of diagnostic tests for prostate cancer.

3 THEORETICAL FRAMEWORK

Acceptability of diagnostic tests has been measured in a number of ways, but no agreed definition for acceptability exists(23). Sekhon et al have proposed a ‘Theoretical Framework of Acceptability’ relating to healthcare interventions, not just diagnostic tests, which includes seven key constructs (See Figure 1): Affective attitude, Burden, Ethicality, Intervention coherence, Opportunity costs, Perceived effectiveness, Self-efficacy(24). This Framework has been developed to be applicable to both patients and clinicians involved in healthcare interventions, and has a number of key constructs that are particularly relevant to the study aims. Eliciting how a patient feels about undergoing mpMRI (‘Affective attitude’), the extent to which they understand the test and its purpose (‘Intervention coherence’), and how likely they perceive mpMRI will achieve the purpose of diagnosing prostate cancer (‘Perceived effectiveness’) will aid understanding in the acceptability of mpMRI as a diagnostic test.

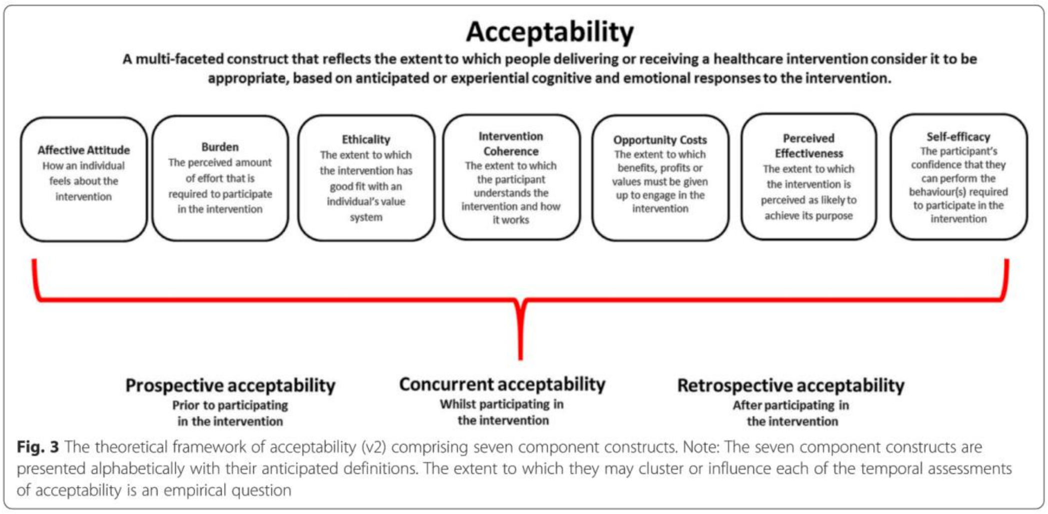

Figure 1 – Sekhon’s Theoretical Framework of Acceptability(24)

Qualitative research methods lend themselves to answering questions of patient and clinician acceptability with regard to diagnostic tests. Such methods allow researchers to “uncover the nature of a person’s experience with a phenomenon”, such as cancer and “understand what lies behind any phenomena”(25). Interview studies provide the opportunity to dig deeper and explore how and why patients and clinicians form their beliefs and understanding. It is assumed by the researchers that every man will experience the prostate cancer diagnostic pathway and the diagnostic tests differently, influenced by both internal and external factors. Therefore, a constructivist approach will be taken to capture a range of experiences(26).

4 RESEARCH QUESTION/AIM(S)

Aim - To understand, from the perspective of patients and GPs, the acceptability of multiparametric magnetic resonance imaging for men as a diagnostic test for prostate cancer

4.1 Objectives

1. Elicit men’s experiences of diagnostic tests for suspected prostate cancer
2. Explore the knowledge and understanding of diagnostic tests for suspected prostate cancer amongst patients and GPs

3. Understand the acceptability of mpMRI as a diagnostic test for prostate cancer from a patient's perspective
4. Understand the acceptability of mpMRI as a diagnostic test for prostate cancer from a GP's perspective

4.2 Outcome

This study seeks to understand the acceptability of mpMRI as a diagnostic test for prostate cancer amongst patients and GPs. mpMRI is increasingly being used as part of the assessment of men with suspected prostate cancer by Urologists prior to undertaking a prostate biopsy, however it is unknown how men experience mpMRI scanning. Studies suggest that mpMRI has a negative predictive value of 85-89%(16,27), and that up to 27% of men could avoid a prostate biopsy based on mpMRI findings(11). Within the NHS, some GPs already have the ability to order 'direct access' diagnostic tests for suspected cancers of the oesophagus, stomach, colon, pancreas and brain, and there is some evidence that pre-biopsy mpMRI could also be used as a 'rule out' test in a prostate cancer diagnostic pathway for some patients(28). Before such an approach could be tested and implemented, the experience of patients undergoing mpMRI, and the acceptability of mpMRI as a diagnostic test for patients and GPs needs to be understood.

5 STUDY DESIGN and METHODS of DATA COLLECTION AND DATA ANALYIS

5.1 Study design

This qualitative study will employ semi-structured interviews with men referred from primary care with suspected prostate cancer who have undergone mpMRI, and GPs who have referred men with suspected prostate cancer for further investigation.

5.2 Data collection

Interviews will be conducted by the lead researcher (SM). Interview data will be gathered using an encrypted recording device. The location of the patient interviews will be agreed between the participant and the interviewer prior to the day of the interview. Ideally they will be conducted face-to-face in the patient's home, but other venues and telephone/Skype interviews will be considered. GP interviews will either be held face-to-face at the GP clinic, or via telephone/Skype. The interviewer will utilise a 'buddy system' of informing a colleague if they are travelling to a private residence unaccompanied to conduct an interview with a patient participant.

The interviews will be conducted in a semi-structured manner, allowing participants to share their experiences of diagnostic tests for prostate cancer freely, whilst also meeting the study objectives. Interviews will be supported by topic guides (see 10.1.6 and 10.1.7) for patient and GP participants, which will be used by the interviewer in a flexible way depending on the length and direction of the interview. These topic guides were developed by the researchers based on their experience and knowledge in the field and the study objectives, and will be adapted iteratively as the initial interviews are conducted to enrich data collection.

It is important to treat all participants equally, regardless of their age, culture, education, language ability, or beliefs. Efforts will be made to respect participants' needs, however this study is not sufficiently funded to meet all possible participant needs, such as interpreters for participants with English as their second language.

Following completion of each interview, audio recordings will immediately be downloaded onto an encrypted university laptop computer and the interviewer will make reflective and summary notes. Audio recordings will be transferred to an independent transcribing service securely and transcribed

verbatim. All participants will be given a pseudonym and any potentially identifying information will be removed.

5.3 Data analysis

A Framework Analysis approach will be taken for this study. Framework analysis is a type of thematic analysis developed by Jane Ritchie and Liz Spencer for applied social policy research(29), and is used in a wide range of areas including health research. Framework Analysis follows seven stages(30):

1. Transcription
2. Familiarisation
3. Coding
4. Developing a working framework
5. Applying framework
6. Charting data into matrix
7. Interpretation

After transcription of the interviews and familiarisation with the data by the investigators, early interview data will be coded using pre-specified codes based on Sekhon's Framework. Refinement and addition of codes and themes will occur with a second researcher (AF) and patient/public representative after coding of initial transcripts using constant-comparison method. The analysis team (SM, AF, and FW) will meet regularly to iteratively develop and agree a coding structure to underpin coding of the remaining transcripts, and a framework will be developed and applied using the agreed codes. SM will perform the final coding of the data. Key themes and narratives within the data will be drawn together from the matrix. Charted data will be imported into NVivo v12 to help manage the data to complete the analysis. Convergence and divergence of views from patients with positive and negative mpMRI scans, and between patients and GPs, will be sought to triangulate key findings.

5.4 Role of the researchers

Three of the researchers (SM, FW, and WH) are trained as GPs, and two are still practicing (SM, FW). All members of the research team will maintain an awareness of their individual biases, beliefs and attitudes that could influence the undertaking of research into men being investigated for prostate cancer. Reflective notes, analysis team meetings, and constant comparison techniques will be used to understand these influences.

6 SAMPLE AND RECRUITMENT

6.1 Eligibility Criteria

This study will recruit participants from two populations;

Patients with suspected prostate cancer who have undergone mpMRI as part of their diagnostic workup.

GPs who have referred at least one male for investigation for suspected prostate cancer within the preceding 12 months.

6.2 Sampling

6.2.1 Size of sample

Approximately 30 participants (10 GPs and 20 patients) will be interviewed for this study, although the final number of participants will depend on when no new themes emerge during interview coding.

6.2.2 Sampling technique

A purposive sampling approach will be taken for participant recruitment to this study. This will allow recruitment of a sample of men with a range of PiRADS scores (1-2 being low risk of prostate cancer; 3-5 being medium-high risk), ages (<70 years or 70+ years), geographical locations (urban or rural/countryside), and ethnic backgrounds (any white background or BME). In terms of GPs, a purposive sampling approach will allow recruitment of clinicians with a range of ages, genders, practice locations (urban or rural/countryside) and levels of experience.

6.3 Recruitment

6.3.1 Sample identification

6.3.1.1 Patient participants

Patient participants for this study will be recruited from two NHS Trusts; the Imperial College Healthcare NHS Trust in London and the Royal Devon & Exeter NHS Foundation Trust in Exeter. Men referred by their GP for suspected prostate cancer undergo an mpMRI prior to clinical review by a Urologist and potentially a prostate biopsy, depending on the mpMRI report (see study flow charts [pg 1-2]). Research nurses and/or fellows working within the clinic will identify potentially eligible men and contact them within days of undergoing an mpMRI to discuss this study and offer the men a Patient Information Leaflet (PIL – See 11.1.2). The PI and staff at the study sites will regularly communicate about potential recruits to ensure a range of age, ethnicity and geographical backgrounds are present in the included participants. Follow-up contact will be made by the research nurse/fellow if the man does not contact the lead researcher to check whether they wish to participate in the study or not.

Both NHS Trusts involved with this study have reviewed and approved this protocol, and they have expressed confidence that recruitment targets will be met. However, in the event that insufficient men are recruited for participation in this study 12 months after commencing recruitment, a further NHS Trust providing urology services that includes mpMRI for possible prostate cancer will be approached to aid recruitment.

Reasonable travel costs for patient participants to attend any face to face interview will be reimbursed, and participants will be offered a £20 gift voucher in recognition of their participation in the study.

6.3.1.2 GP participants

GP participants will be recruited through two National Institute for Health Research (NIHR) Clinical Research Networks (CRNs); North West London CRN and the South-West Peninsula CRN (see study flow chart [pg 3]). The CRNs will identify local practices from which to recruit eligible GPs to participate in this study, favouring Research Site Initiative (RSI) practices as these practices have an ongoing commitment to research and may have allocated research clinician time. In practices that do not have funded research clinician time, the CRN may provide support for participation in the study. Eligible GPs will be identified by the CRN and the practices, and they will regularly communicate with the PI to determine which GPs to approach for participation. GPs chosen for invitation into the study will be given a PIL (See 11.1.3) to consider participating in the study, and follow-up contact will be made by the CRN to confirm participation.

The NIHR CRN in South-West Peninsula and North West London have reviewed and approved this protocol, and they have expressed confidence in meeting recruitment targets. National adoption of this study within the NIHR CRN portfolio will allow the possibility of recruitment from other CRNs if there is any difficulty recruiting GPs in these two regions.

GP practices will be reimbursed £44.10 per 30 minute interview for the GP's time to participate.

6.3.2 Consent

All participants contacted for participation in this study will be given a PIL after being contacted as a potentially eligible participant. After reading the PIL, if the participant is willing to participate they will be contacted by the lead researcher to arrange an interview.

Consent will be taken at the start of the interview. The purpose of the study and the interview will be explained in conjunction with the information presented in the PIL. The lead researcher will assess the patient's ability to consent for participation. The participant will then be presented with a consent form (See 10.1.2 and 10.1.3) to complete if they are still willing to participate in the study.

Conducting patient interviews in their own home may result in other parties being present during the interview, such as the patient's spouse. If another person is present, the patient participant will be asked if they are happy to initially be interviewed in private. If the patient participant wishes for another person to be present, the additional person will be consented for participation and asked to complete a consent form before they join the interview.

6.3.3 Completing recruitment

Recruitment of patients and GPs will continue alongside analysis of interviews conducted to date, until no new themes or ideas emerge from the data.

7 ETHICAL AND REGULATORY CONSIDERATIONS

7.1 Data protection

All data will be collected, stored and processed in accordance with the General Data Protection Regulations 2018 and the Data Protection Act 2018. Informed consent will be obtained from all participants for all aspects of the study. Permission for the collection, storage and use of patient identifiable data (PID) in the study will be provided by consenting participants.

Participants will be free to withdraw consent for participation for any reason and at any time. Where consent is withdrawn, all participant identifiable information held by the research team will be destroyed, and the participant will not receive any further contact regarding the study.

7.2 Data anonymisation

All collected data will be fully anonymised before transfer to professional transcription services. Direct quotations from interview may be used in presenting the study results, however interviewees will not be identifiable in any way in any quotations used.

7.3 Data Storage

Encrypted voice recorders will be used for the interviews. Audio data will be downloaded and kept on secure servers at the University of Exeter until fully anonymised transcripts are created. Any audio files sent to professional transcription services will be anonymously labelled with a unique code, and encrypted for transfer. All physical data such as consent forms and transcripts of interviews will be stored within locked filing cabinets, within a locked office within the University of Exeter Medical School. The keys will be stored separately and only be accessed by the local research team.

All personal data will be securely destroyed within 12 months after the end of the study.

7.4 Assessment and management of risk

This study may be viewed as potentially sensitive in that it explores experiences of personal and intimate symptoms and body systems, in the context of a potentially serious diagnosis (cancer). Although there is a potential ethical problem with interviewing patients around the time of a cancer diagnosis, the proposed recruitment approaches have been successfully used in previous UK early

diagnosis of cancer studies, led by my supervisor, Dr Fiona Walter. These studies include questionnaire and interview studies people in patients with symptoms suggestive of lung, colorectal and pancreatic cancer (the SYMPTOM study)(31,32), an interview study set among people recently diagnosed with melanoma(33), and the on-going ECASS study (weekly case note reviews and subsequent GP and patient interviews in people with gastro-oesophageal symptoms).

Nevertheless, there are a number of strategies in place to minimise any possible distress that may arise in patients (and informal carers) during the study:

Interviews will be conducted by SM, who has been trained in qualitative interviewing. Combining with his experience as a GP, he would have had experience of communicating sensitively, empathetically and sympathetically with patients when breaking bad news and around sensitive topics, including cancer. Interviews will be conducted at a time and location convenient to participants, and in an unhurried manner, with participants being free to stop the discussion at any time. Appropriate leave-taking will also be practised to ensure that participants are not left in a distressed state following the interview.

It would be reasonable to assume that patients being recruited for interview are aware that they are being investigated with mpMRI due to the suspicion of them having prostate cancer. Local NHS protocols for referring men on the two-week wait urgent cancer referral pathway include the need for GPs to inform the patient they are being referred for a suspected diagnosis of cancer. However, the interviews for this study will not be conducted with that assumption. Early in the interview the patient's understanding for the reason for their referral for mpMRI and other investigations will be explored, to ensure that they patient's underlying knowledge and assumptions are clear to the interviewer.

SM is a practicing GP, and there is the possibility that patients will want to seek advice or an opinion form SM about their healthcare relating to the issue of possible prostate cancer or another health issue. SM will be clear that he is conducting the interview in his capacity as a researcher, and will refer any questions about the patient's healthcare back to their own GP.

A procedure is followed in the event of a participant becoming distressed, which includes the interviewer expressing concern as early as possible about the participant's comfort, offering them tissues or water, and asking whether they would like to take a break or discontinue the interview. Support mechanisms are in place (see Box 1) and the interviewer will inform the participant of these. Participants will also be reminded that they can withdraw from the study or complete the interview at another time, and that this is entirely their decision.

SM will meet regularly during data collection with his supervisors, FW and AF, who have extensive clinical and research experience with patients about cancer symptoms and pathways to diagnosis. This will ensure that should any issues arise they can be dealt with expediently, and learning applied for subsequent interviews.

Box 1: Guidelines for researchers conducting sensitive interviews

1. All interviews are to be conducted from the outset with the greatest of sensitivity and concern for the respondent's welfare.

2. The interviewer should be observant of the respondent's level of comfort and watch for early signs of distress, such as breaks in speech or nervous body movements. Should early signs appear the interviewer should express concern about the respondent's comfort and ask questions such as (gauged by respondent's signs): would they like a glass of water; if a break is needed; if they would prefer to complete the interview another time; or if they would prefer to discontinue.

3. If overt distress occurs, the interview should cease immediately and actions taken to support the respondent, such as offering tissues or water; seeking immediate additional support from a more familiar person, if available; and staying with the respondent until they are ready to express their wishes on the options available to them.

4. If it becomes apparent that a distressed respondent has particular areas of need concerning their illness or circumstances, where appropriate, the interviewer should offer to assist the respondent to make contact with a relevant support, such as their GP surgery.

5. Concerning the interview, the options eventually offered to a distressed respondent should be (in order): withdraw from the study; or complete the interview another time. The interview should only be continued after a break if the respondent requests this as their unprompted decision.

7.5 Adverse Events

An adverse event will be defined as 'an event that arises directly from participation in the research', including complications that occur in the course of investigation. All adverse events will be discussed with the Supervisors, both of whom (especially FW) have had extensive experience in carrying out similar studies involving early diagnosis of cancer. Appropriate subsequent course of action will be taken after discussion with the full research team, and the Sponsor will be notified.

Appropriate safety procedures will be followed by the researcher(s) when interviewing participants. Should any disclosures requiring action be made, the researchers will have access to the support of the full research team.

7.6 Insurance

Arrangements have been made through the University of Exeter for insurance and/or indemnity to meet the potential legal liability of the sponsor for harm to participants arising from the management, design or conduct of the research.

NHS indemnity scheme will apply for insurance and/or indemnity to meet the potential legal liability of the investigator arising from harm to participants in the conduct of the research at NHS sites.

There are no arrangements in place for payment of compensation in the event of harm to the research participants where no legal liability arises.

7.7 Research Ethics Committee (REC) and other Regulatory review & reports

The researcher will seek NHS research governance and compliance approval, and NHS ethical review, through the HRA approvals process via the Integrated Research Application System (IRAS).

The researcher will ensure that the protocol and all supporting participant-facing documentation receive HRA approval. Following review, research will only take place once appropriate HRA and Sponsor approvals are in place and confirmation of capacity and capability received from each local NHS site.

A copy of the approved study documents will be submitted to the R&D Office or practice manager at each local site prior to the commencement of any study procedures.

The Chief Investigator is responsible for keeping all correspondence with the REC, producing annual reports, notifying the REC at the end of the study and producing final reports.

7.8 Protocol compliance

Any accidental protocol deviations will be adequately documented on the relevant forms and reported to the Researcher and Sponsor. All deviations from the protocol which are found to frequently recur will require immediate action and could potentially be classified as a serious breach.

Notification of Serious Breaches to GCP and/or the protocol: A 'serious breach' is a breach which is likely to affect to a significant degree;

The safety or physical or mental integrity of the subjects of the study; or

The scientific value of the study

The Sponsor will be notified immediately of any case where the above definition applies during the study.

7.9 Amendments

For any amendment to the study, the Chief Investigator or designee, in agreement with the sponsor will submit information to the appropriate body in order for them to issue approval for the amendment. The Chief Investigator or designee will work with sites (R&D departments at NHS sites as well as the study delivery team) so they can put the necessary arrangements in place to implement the amendment to confirm their support for the study as amended.

If the sponsor wishes to make a substantial amendment to the REC application or the supporting documents, the sponsor must submit a valid notice of amendment to the REC for consideration. The REC will provide a response regarding the amendment within 35 days of receipt of the notice. It is the sponsor's responsibility to decide whether an amendment is substantial or non-substantial for the purposes of submission to the REC.

Amendments will also be notified to the HRA national coordinating function of England where the lead NHS R&D office is based and communicated to the participating organisations (R&D office and local research team) and departments of participating sites to assess whether the amendment affects the NHS permission for that site.

Amendments considered to be non-substantial for the purposes of REC will still be notified to the HRA for approval after confirmation from the Sponsor.

7.10 Peer review

The PhD proposal, which this study forms a key part of, has been subject to peer review by two senior researchers within the CanTest programme. Both reviewers are external to the University of Exeter and are not involved with this study in any way. They are both very experienced and widely published primary care cancer researchers. Feedback from the peer review was utilised to refine and enchance the development of this study.

7.11 Patient & Public Involvement

The NIHR Clinical Research Facility Peninsula Research Bank steering committee includes a panel of lay members. 14 members of the panel, including two members who had previously been service users investigated for possible prostate cancer, provided input into the acceptability and design of this study. The lay members assisted in the writing of the lay summary, and they reviewed drafts of the consent form, participant information leaflets, and interview guides. Feedback received from these service users and members of the public has been integrated into this protocol and associated documents.

Separately, a PPI group of men (with no history of prostate cancer) is currently being assembled to help steer the PhD that this study forms a part of. This PPI group will be involved with the analysis of results and the dissemination of findings from this study.

7.12 Access to the final study dataset

Access to the full dataset will be limited to the researcher and the supervisors for this study. In line with Cancer Research UK (CRUK) policy, fully anonymised interview transcripts will potentially be made available to researchers for analysis in future related studies, subject to consent obtained from participants.

Any research nurses or fellows involved with recruitment will not have any access to the data collected.

8 DISSEMINIATION POLICY

8.1 Dissemination policy

The data arising from the study will be owned by the University of Exeter.

On completion of the study, data will be analysed and synthesised into a chapter for the PhD of the researcher, registered at the University of Exeter. Access to the full study report, including the protocol, will be made through the Open Research Exeter (ORE) online portal, hosted by the University of Exeter, after the thesis has been accepted by The University.

CRUK are the major funders of this study, through a Catalyst Award (‘CanTest’). CanTest funds the researcher’s salary, training, PhD fees and research costs. CRUK will be acknowledged in any publications associated with this study.

All participants will receive an abridged study report, outlining the major findings of the study.

8.2 Authorship eligibility guidelines and any intended use of professional writers

Authorship for the final study report and any publications associated with this study will be agreed in accordance with the International Committee of Medical Journal Editors guidance.

9 REFERENCES

1. Lijmer JG, Leeflang M, Bossuyt PMM. Proposals for a phased evaluation of medical tests. Med Decis Mak. 2009;29(5):13–21.
2. Fryback DG, Thornbury JR, Fryback DG, Thornbury JR. Medical Decision Making The Efficacy of Diagnostic Imaging. Med Decis Mak. 1991;11:88–94.
3. Horvath AR, Lord SJ, StJohn A, Sandberg S, Cobbaert CM, Lorenz S, et al. From biomarkers to medical tests: The changing landscape of test evaluation. Clin Chim Acta [Internet]. 2014;427:49–57. Available from: http://dx.doi.org/10.1016/j.cca.2013.09.018
4. Lin JS, Thompson M, Goddard KAB, Piper MA, Heneghan C, Whitlock EP. Evaluating genomic

tests from bench to bedside: A practical framework. BMC Med Inform Decis Mak [Internet]. 2012;12(1):1. Available from: BMC Medical Informatics and Decision Making

5. Thompson M, Weigl B, Fitzpatrick A, Ide N. More Than Just Accuracy: A Novel Method to Incorporate Multiple Test Attributes in Evaluating Diagnostic Tests Including Point of Care Tests. IEEE J Transl Eng Heal Med. 2016;4(January).

6. National Collaborating Centre for Cancer. Suspected cancer [Internet]. NICE. London; 2015. Available from: http://journals.rcni.com/doi/10.7748/ns.29.47.17.s20

7. Mottet N, Bellmunt J, Briers E, Bolla M, Bourke L, Cornford P, et al. EAU - ESTRO - ESUR - SIOG Guidelines on Prostate Cancer [Internet]. European Association of Urology; 2017. Available from: https://uroweb.org/wp-content/uploads/09-Prostate-Cancer_2017_web.pdf

8. Loeb S, Vellekoop A, Ahmed HU, Catto J, Emberton M, Nam R, et al. Systematic review of complications of prostate biopsy. Eur Urol [Internet]. 2013 Dec 1;64(6):876–92. Available from: http://dx.doi.org/10.1016/j.eururo.2013.05.049

9. Hassanzadeh E, Glazer DI, Dunne RM, Fennessy FM, Harisinghani MG, Tempany CM. Prostate imaging reporting and data system version 2 (PI-RADS v2): a pictorial review. Abdom Radiol (New York) [Internet]. 2017;42(1):278–89. Available from: http://www.ncbi.nlm.nih.gov/pubmed/27522352%0Ahttp://www.pubmedcentral.nih.gov/articlerender.fcgi?artid=PMC5247306%0Ahttp://www.ncbi.nlm.nih.gov/pubmed/27522352%5Cnhttp://www.pubmedcentral.nih.gov/articlerender.fcgi?artid=PMC5247306

10. Ahmed HU, Bosaily AE-S, Brown LC, Gabe R, Kaplan R, Parmar MK, et al. Diagnostic accuracy of multi-parametric MRI and TRUS biopsy in prostate cancer (PROMIS): a paired validating confirmatory study. Lancet [Internet]. 2017 Jan 19;380:1–8. Available from: http://dx.doi.org/10.1016/S0140-6736(16)32401-1

11. Kasivisvanathan V, Rannikko AS, Borghi M, Panebianco V, Mynderse LA, Vaarala MH, et al. MRI-Targeted or Standard Biopsy for Prostate-Cancer Diagnosis. N Engl J Med [Internet]. 2018; Available from: http://www.nejm.org/doi/10.1056/NEJMoa1801993

12. Bray F, Ferlay J, Soerjomataram I, Siegel R, Torre L, Jemal A. Global cancer statistics 2018: GLOBOCAN estimates of incidence and mortality worldwide for 36 cancers in 185 countries. CA A J Clin. 2018;00(00):1–31.

13. Office for National Statistics. Cancer registration statistics, England: first release, 2015. Statistical Bulletin. 2017.

14. Sandhu GS, Andriole GL. Overdiagnosis of prostate cancer. J Natl Cancer Inst - Monogr [Internet]. 2012 Dec 27;2012(45):146–51. Available from: http://jncimono.oxfordjournals.org/cgi/doi/10.1093/jncimonographs/lgs031

15. Boesen L, Norgaard N, Logager V, Balslev I, Thestrup K, Bisbjerg R, et al. Assessment of the Diagnostic Accuracy of Biparametric Magnetic Resonance Imaging for Prostate Cancer in Biopsy-Naive Men: The Biparametric MRI for Detection of Prostate Cancer (BIDOC) study. JAMA Netw Open. 2018;1(2).

16. Otti VC, Miller C, Powell RJ, Thomas RM, McGrath JS. The diagnostic accuracy of multiparametric MRI prior to biopsy in the detection of prostate cancer. BJU Int [Internet]. 2018; Available from: http://www.ncbi.nlm.nih.gov/pubmed/29804315%0Ahttp://doi.wiley.com/10.1111/bju.14420

17. NHS England. Implementing a timed prostate cancer diagnostic pathway. NHS; 2018.

18. Peters JL, Thompson AC, Mcnicholas TA, Hines JEW, Hanbury DC, Boustead GB. Increased patient satisfaction from transrectal ultrasonography and biopsy under sedation. BJU Int. 2001;87(9):827–30.

19. Makinen T, Auvinen A, Hakama M, Stenman UH, Tammela TLJ. Acceptability and

complications of prostate biopsy in population-based PSA screening versus routine clinical practice: A prospective, controlled study. Urology. 2002;60(5):846–50.

20. Clements R, Aideyan OU, Griffiths GJ, Peeling WB. Side effects and patient acceptability of transrectal biopsy of the prostate. Clin Radiol. 1993;47(2):125–6.

21. Egbers N, Schwenke C, Maxeiner A, Teichgräber U, Franie T. MRI-guided core needle biopsy of the prostate: Acceptance and side effects. Diagnostic Interv Radiol. 2015;21(3):215–21.

22. Ullrich T, Schimmöller L, Oymanns M, Blondin D, Dietzel F, Kirchner J, et al. Current Utilization and Acceptance of Multiparametric MRI in the Diagnosis of Prostate Cancer. A Regional Survey. RöFo - Fortschritte auf dem Gebiet der Röntgenstrahlen und der Bildgeb Verfahren [Internet]. 2017;419–26. Available from: http://www.thieme-connect.de/DOI/DOI?10.1055/s-0043-118128

23. Sekhon M, Cartwright M, Francis JJ. Acceptability of health care interventions: A theoretical framework and proposed research agenda. Br J Health Psychol. 2018;1–13.

24. Sekhon M, Cartwright M, Francis JJ. Acceptability of healthcare interventions: An overview of reviews and development of a theoretical framework. BMC Health Serv Res [Internet]. 2017;17(1):1–13. Available from: http://dx.doi.org/10.1186/s12913-017-2031-8

25. Strauss A, Corbin J. Basics of qualitative research. Sage; 1990.

26. Silverman D. Doing qualitative research. Sage; 2013.

27. Ahmed HU, El-Shater Bosaily A, Brown LC, Gabe R, Kaplan R, Parmar MK, et al. Diagnostic accuracy of multi-parametric MRI and TRUS biopsy in prostate cancer (PROMIS): a paired validating confirmatory study. Lancet [Internet]. 2017;389(10071):815–22. Available from: http://dx.doi.org/10.1016/S0140-6736(16)32401-1

28. Panebianco V, Barchetti G, Simone G, Del Monte M, Ciardi A, Grompone MD, et al. Negative Multiparametric Magnetic Resonance Imaging for Prostate Cancer: What's Next? Eur Urol [Internet]. 2018;74(1):48–54. Available from: https://doi.org/10.1016/j.eururo.2018.03.007

29. Ritchie J, Spencer EA. Qualitative data analysis for applied policy research. In: Analyzing Qualitative Data. 1st ed. London: Routledge; 1994. p. 173–94.

30. Gale NK, Heath G, Cameron E, Rashid S, Redwood S. Using the framework method for the analysis of qualitative data in multi-disciplinary health research. BMC Med Res Methodol [Internet]. 2013;13(1):1. Available from: BMC Medical Research Methodology

31. Birt L, Hall N, Emery J, Banks J, Mills K, Johnson M, et al. Responding to symptoms suggestive of lung cancer: A qualitative interview study. BMJ Open Respir Res. 2014;1(1).

32. Hall N, Birt L, Banks J, Emery J, Mills K, Johnson M, et al. Symptom appraisal and healthcare-seeking for symptoms suggestive of colorectal cancer: A qualitative study. BMJ Open. 2015;5(10).

33. Walter FM, Birt L, Cavers D, Scott S, Emery J, Burrows N, et al. "This isn't what mine looked like": A qualitative study of symptom appraisal and help seeking in people recently diagnosed with melanoma. BMJ Open. 2014;4(7):1–12.

10. APPENDICIES

10.1 Amendment History

Amendment No.	Protocol version no.	Date issued	Author(s) of changes	Details of changes made
