# Supplementary material for: Experiences of ‘traditional’ and ‘one-stop’ MRI-based prostate cancer diagnostic pathways in England: a qualitative study with patients and GPs
Source: BMJ Open. 2022 Jul 26;12(7):e054045. doi: 10.1136/bmjopen-2021-054045 (PMC9330318; doi:10.1136/bmjopen-2021-054045)
Supplement: Author's manuscript [file bmjopen-2021-054045.draft_revisions.pdf]

BMJ Open

BMJ Open is committed to open peer review. As part of this commitment we make the peer review history of every article we publish publicly available.

When an article is published we post the peer reviewers' comments and the authors' responses online. We also post the versions of the paper that were used during peer review. These are the versions that the peer review comments apply to.

The versions of the paper that follow are the versions that were submitted during the peer review process. They are not the versions of record or the final published versions. They should not be cited or distributed as the published version of this manuscript.

BMJ Open is an open access journal and the full, final, typeset and author-corrected version of record of the manuscript is available on our site with no access controls, subscription charges or pay-per-view fees (http://bmjopen.bmj.com).

If you have any questions on BMJ Open's open peer review process please email info.bmjopen@bmj.com

BMJ Open

Experiences of ‘traditional’ and ‘one-stop’ MRI-based prostate cancer diagnostic pathways in England: a qualitative study with patients and GPs

Journal:	*BMJ Open*
Manuscript ID	bmjopen-2021-054045
Article Type:	Original research
Date Submitted by the Author:	02-Jun-2021

Complete List of Authors:	Merriel, Samuel; University of Exeter, Institute of Health Research<br>Archer, Stephanie; University of Cambridge School of Clinical Medicine, Public Health and Primary Care; University of Cambridge<br>Forster, Alice; UCL, Behavioural Science and Health<br>Eldred-Evans, David; Imperial College London, Imperial Prostate<br>McGrath, John; Royal Devon and Exeter NHS Foundation Trust, Urology<br>Ahmed, Hashim; Imperial College London, Imperial Prostate<br>Hamilton, Willie; University of Exeter, Peninsula College of Medicine and Dentistry<br>Walter, Fiona; University of Cambridge, Dept of Public Health and Primary Care

Keywords:	PRIMARY CARE, QUALITATIVE RESEARCH, Prostate disease < UROLOGY, Urological tumours < ONCOLOGY, Magnetic resonance imaging < RADIOLOGY & IMAGING

SCHOLARONE™
Manuscripts

BMJ

I, the Submitting Author has the right to grant and does grant on behalf of all authors of the Work (as defined in the below author licence), an exclusive licence and/or a non-exclusive licence for contributions from authors who are: i) UK Crown employees; ii) where BMJ has agreed a CC-BY licence shall apply, and/or iii) in accordance with the terms applicable for US Federal Government officers or employees acting as part of their official duties; on a worldwide, perpetual, irrevocable, royalty-free basis to BMJ Publishing Group Ltd ("BMJ") its licensees and where the relevant Journal is co-owned by BMJ to the co-owners of the Journal, to publish the Work in this journal and any other BMJ products and to exploit all rights, as set out in our licence.

The Submitting Author accepts and understands that any supply made under these terms is made by BMJ to the Submitting Author unless you are acting as an employee on behalf of your employer or a postgraduate student of an affiliated institution which is paying any applicable article publishing charge ("APC") for Open Access articles. Where the Submitting Author wishes to make the Work available on an Open Access basis (and intends to pay the relevant APC), the terms of reuse of such Open Access shall be governed by a Creative Commons licence – details of these licences and which Creative Commons licence will apply to this Work are set out in our licence referred to above.

Other than as permitted in any relevant BMJ Author's Self Archiving Policies, I confirm this Work has not been accepted for publication elsewhere, is not being considered for publication elsewhere and does not duplicate material already published. I confirm all authors consent to publication of this Work and authorise the granting of this licence.

For peer review only

Experiences of ‘traditional’ and ‘one-stop’ MRI-based prostate cancer diagnostic pathways in England: a qualitative study with patients and GPs

For peer review only

Dr Samuel W D Merriel MSc, University of Exeter Medical School, University of Exeter, UK

s.w.d.merriel@exeter.ac.uk

Dr Stephanie Archer PhD, Department of Public Health and Primary Care, University of Cambridge, UK

saa71@medschl.cam.ac.uk

Dr Alice Forster PhD, Institute of Epidemiology & Health, University College London, UK

alice.forster@ucl.ac.uk

Dr David Eldred-Evans PhD, Imperial Prostate, Department of Surgery & Cancer, Imperial College London, UK; Imperial Urology, Charing Cross Hospital, Imperial College Healthcare NHS Trust, London, UK

d.eldred-evans@imperial.ac.uk

Mr John McGrath FRCS, Department of Urological Surgery, Royal Devon & Exeter NHS Foundation Trust, UK

john.mcgrath4@nhs.net

Prof Hashim U. Ahmed FRCS, Imperial Prostate, Department of Surgery & Cancer, Imperial College London, UK; Imperial Urology, Charing Cross Hospital, Imperial College Healthcare NHS Trust, London, UK

Hashim.ahmed@imperial.ac.uk

Prof Willie Hamilton MD, University of Exeter Medical School, University of Exeter, UK

w.hamilton@exeter.ac.uk

Dr Fiona M Walter MD, The Primary Care Unit, Department of Public Health and Primary Care, University of Cambridge, UK

fmw22@medschl.cam.ac.uk

Corresponding author

Dr Samuel W D Merriel

1.18 College House, St Luke's campus, University of Exeter

Heavitree Road, Exeter, UK EX1 2LU

+441392 726002

s.w.d.merriel@exeter.ac.uk

Keywords: Prostate cancer, diagnostic pathway, primary care

For peer review only

Abstract

Objectives

This study aimed to understand and explore patient and GP experiences of 'traditional' and 'one-stop' prostate cancer diagnostic pathways in England.

Design

Qualitative study using semi-structured interviews, analysed using inductive thematic analysis

Setting

Patients were recruited from National Health Service (NHS) Trusts in London and in Devon; GPs were recruited via National Institute for Health Research (NIHR) Clinical Research Networks. Interviews were conducted in person or via telephone.

Participants

Patients who had undergone a magnetic resonance imaging (MRI) scan of the prostate as part of their diagnostic work-up for possible prostate cancer, and GPs who had referred at least one patient for possible prostate cancer in the preceding 12 months.

Results

22 patients (aged 47 – 80 years) and 10 GPs (6 female, aged 38 – 58 years) were interviewed. Patients described three key themes: cancer beliefs in relation to patient's attitudes towards prostate cancer; communication with their GP and specialist having a significant impact on experience of the pathway; and pathway experience being influenced by appointment and test burden. GP interview themes included: the challenges of dealing with imperfect information in the current pathway; managing uncertainty in identifying patients with possible prostate cancer, and sharing this uncertainty with them, and other social, cultural and personal contextual influences.

Conclusions

Patients and GPs reported a range of experiences and views of the current prostate cancer diagnostic pathways in England. Patients valued 'one-stop' pathways integrating prostate MRI and diagnostic consultations with specialists over the more traditional approach of several hospital appointments. GPs remain uncertain how best to identify patients needing referral for urgent prostate cancer testing due to the lack of accurate triage and risk assessment strategies.

For peer review only

Article summary

Strengths and limitations

- Patient experiences of two very different prostate cancer diagnostic pathways compared and contrasted
- Patient sample feature a broad range of ages, geographical regions, and cancer investigation journeys to generate rich data
- First study to explore GP experience and understanding of new prostate cancer diagnostic pathways incorporating magnetic resonance imaging (MRI)
- Limited knowledge of prostate MRI curtailed interviews with some GP participants

For peer review only

Introduction

National Institute for Health and Care Excellence (NICE) guidance for diagnosing prostate cancer in England was updated in 2019 to recommend pre-biopsy magnetic resonance imaging (MRI) for men suspected of having prostate cancer, and in those with a suspicious MRI to have an MRI-influenced biopsy of visible lesions [1]. In response, Cancer Alliances and Hospital Trusts in the National Health Service (NHS) of the UK have updated local prostate cancer diagnostic pathways, with significant variation in the integration of MRI [2]. Some have continued to follow more traditional outpatient-based pathways with multiple appointments (figure 1), whilst others have incorporated imaging, consultant review and diagnostic testing into a single, 'one-stop' outpatient attendance (figure 2).

Figure 1

Figure 2

Significant variation in cancer diagnostic pathway intervals between NHS Trusts and regions in England has previously been demonstrated, most markedly for prostate cancer [3]. Integrating additional MRI testing into the prostate cancer pathway could lengthen these pathway intervals. Furthermore, patients with shorter diagnostic intervals generally report better experiences of cancer diagnostic pathways [4]; therefore, adding MRI could adversely affect patient experience.

Worldwide, few studies have assessed the effects of implementing MRI for prostate cancer diagnosis on patient experience or clinician understanding. The aim of this study was to elicit the experience of patients and GPs following two prostate cancer diagnostic pathways that incorporate pre-biopsy MRI in different ways.

For peer review only

Methods

This qualitative study used semi-structured interviews to explore the experiences of patients referred from primary care with possible prostate cancer who had undergone an MRI, and GPs who have referred men with possible prostate cancer for further investigation. A constructivist approach was adopted to understand the experiences of patients and GPs[5] as it was assumed that patient and GP participants would construct their views based on their individual experiences (past and present) and the socio-cultural context[6,7].

Participants

This study recruited participants from two populations;

- Patients with possible prostate cancer who had undergone an MRI as part of their diagnostic workup.
- GPs who had referred at least one patient for investigation for possible prostate cancer within the preceding 12 months.

Patients who were undergoing MRI for active surveillance or watchful waiting for a previously diagnosed prostate cancer were not eligible, as the focus of this study was on the role of MRI in the diagnosis of prostate cancer rather than management.

Recruitment

Patients were recruited from two NHS Trusts in England: the Royal Devon & Exeter NHS Foundation Trust in Exeter and the Imperial College Healthcare NHS Trust in London. The Royal Devon & Exeter Hospital use separate outpatient appointments in the South West (SW) Prostate Cancer Diagnostic Pathway for a prostate MRI, consultant review, and prostate biopsy (if required), as shown in Figure 1. Imperial College employ the RAPID pathway, where patients undergo a prostate MRI scan, receive their MRI result, and potentially undergo a prostate biopsy on the same day at a single outpatient attendance (see Figure 2). Research staff at the Trusts identified potentially eligible men and contacted them within days of undergoing an MRI to discuss this study and offer the men a Patient Information Leaflet (PIL). The lead investigator and local recruitment leads were in regular contact throughout recruitment to identify any under-represented groups of men and focus recruitment where needed. Travel costs for patient

For peer review only

participants to attend a face to face interview were reimbursed, and participants were also offered a gift voucher in recognition of contributing their time.

GPs were recruited through two National Institute for Health Research (NIHR) Clinical Research Networks (CRNs) in the same regions as the hospital sites: North West London CRN and the South-West Peninsula CRN. The CRNs promoted the study to local practices, and GPs expressed their interest to the CRNs. Eligibility and basic demographics were checked to assist with purposive sampling. GPs chosen for invitation into the study were given a PIL to review prior to the arrangement of an interview. GP practices were reimbursed for the GP's time to participate in the study.

A purposive sampling approach was used, in order to obtain a diverse group of participants with a wide range of geographical locations, ages, genders (GPs) and MRI results (patients).

Data collection

One-to-one interviews were conducted with all participants in this study between July and November 2019 by SM. Patient participants were mostly interviewed face-to-face in their own home, while all GP participant interviews were conducted via telephone. Formal written consent was obtained from all participants, and patient's partners if present, prior to commencement of the interview. A semi-structured approach was followed, with separate interview topic guides for patient and GP interviews to support discussions (See supplementary file 1 and 2). The topic guide was developed to incorporate all aspects of the revised prostate cancer diagnostic pathway, and was used flexibly within the interviews to ensure that no key aspects of the diagnostic pathway experience were missed. An encrypted audio recording device was employed to record all interviews, and written notes were taken during and immediately following the interviews. Interview times ranged between 15-45 minutes each. Interview recordings were transferred securely to an independent transcribing service, and transcribed verbatim.

Data analysis

For peer review only

An inductive thematic analysis was conducted to understand the experiences of participants[8]. The researchers initially immersed themselves in the data through reading and re-reading individual transcripts and listening back to the audio recordings of the interviews. A selection of early interviews were coded, and this initial coding framework was reviewed and refined by SM, SA and FW. The remaining interview transcripts were coded inductively from the entirety of the data. The codes were reviewed and arranged into themes through an iterative process, returning to the original data as needed. Patient and GP transcripts were analysed separately. Within and between themes, the experiences of participants following different diagnostic pathways were compared and contrasted. Recruitment ceased when no new themes emerged in analysis. Transcripts were imported into NVivo v12 to manage the data for the analysis. A study summary report was sent to all study participants after completion of data analysis.

Patient & Public (PPI) Involvement statement

Eight men were recruited via the People in Health West of England (PHWE) initiative to contribute to the research: these men had a range of ages, locations, ethnic backgrounds and experiences with prostate cancer. PPI group members reviewed the plain English summary and all patient participant documents, and gave feedback prior to submission as part of the ethical approval application. PPI group members also gave input into the interview topic guides and the expected burden of involvement for participants. One of the anonymised patient interview transcripts was shared with the group at a meeting, and discussed to explore themes emerging from the text.

Ethical approval

Ethics committee approval was received from the NHS HRA South-West Frenchay research ethics committee (REC reference 19/SW/0040).

COREQ reporting guidelines

This manuscript has been written in accordance with the consolidated criterion for reporting qualitative research (COREQ) checklist[9].

For peer review only

Results

Participants

Twenty-two patients were interviewed, and two chose to have their wives present and involved in the interview: participant ages ranged from 47 – 80 years. Ten GPs were interviewed: most were female (n = 6), with an age range of 38 – 58 years (See table 1). Five further potential (three patients and two GPs) participants were approached but declined to participate.

	Patients (n = 22)		GPs (n = 10)
**Age**		**Age**	
<65	8	31-40	3
65+	14	41-50	6
		50+	1
**Location**		**Location**	
London	10	London	4
Devon	12	Devon	6
**Ethnicity**		**Gender**	
White	19	Male	4
BME	3	Female	6
**PIRADS v2***		**Role**	
1-2	6	Partner	8
3-5	15	Salaried	2
Unknown	1		

Table 1 – Patient and GP demographics

* PIRADS – Prostate Imaging-Reporting And Data System v2 score of 1-2 suggest clinically significant prostate cancer is unlikely and biopsy not indicated. A PIRADS score of 3-5 indicates at least one suspicious area of the prostate that warrants biopsy.

Patient experiences of the prostate cancer diagnostic pathway

For peer review only

We identified three main themes with interlinking sub-themes (see figure 3): Cancer beliefs, Communication, and Pathway experience.

Figure 3

Cancer beliefs

The decision for patients to see their doctor about potential prostate problems was not undertaken in isolation (*Outside influences*). The experiences of family members and friends shaped the patients' expectations, and family members and partners often encouraged men to be tested:

> *"Obviously back then he [dad] was in his mid to late 60s. And I think I didn't really know about it until he'd gone for his MRI and got the results and everything, and then all of a sudden he sat me down and told me all about it." P20 (London, <65)*

Most patients' attitudes towards the possibility of a diagnosis of cancer (*Attitude to diagnosis*) were fairly relaxed. Many seemed philosophical about the prospect:

> *"it is what it is" P03 (Devon, <65)*

A few patients expressed anxiety about being told they might have prostate cancer, often younger men with families.

> *"so I'm panicking obviously, we have a young family, I'm thinking I'm going to have problems" P08 (London, <65)*

The reactions of patients who had a diagnosis of prostate cancer (*Reaction to findings*) were mixed, ranging from despondence to quick acceptance:

For peer review only

“Not fair. No, it’s... it’s not fair on... on anyone, not just me. It isn’t fair on anyone.” P01 (Devon, 65+)

Communication

The absence of the use of the word cancer (‘*C word*’) was evident in interviews with many patients:

“And then this developed.” P01 (Devon, 65+)

“For me, my... my dad had it roughly about eight, nine years... eight to ten years ago, I suppose. He had it.” P20 (London, <65)

Patients also reported a reluctance from clinicians to raise cancer specifically as a possibility during a consultation, even if they were referred for urgent tests to rule out a diagnosis of prostate cancer:

“The only thing that I found was you were given leaflets that mention a lot about cancer but no one actually really, sort of like said to me, you know, there’s a possibility that you could have cancer or you know, that you’re just being given leaflets and such, and no one really explained to you that there is a possibility. But when you’re being given leaflets and it’s got cancer and all that written all over it, you think well, is it cancer? Isn’t it cancer? You know? Possibly, you know, could have had a bit more explanation into you know… no one’s actually said to me you have or you haven’t. A straightforward answer, you have or you haven’t. It’s more that yeah, everything looks fine, you know there’s no, but when you’re being handed leaflets all the time it does become a bit of a worry for a while.” P25 (Devon, <65)

Language and humour were key tools used by many patients to cope with discussing a private and sensitive health issue with others, allowing them to identify with men experiencing similar problems

"And, I mean, there are jokes amongst friends, you know, of my own age about how many times we need to get up in the night…" P13 (London, 65+)

The mode of communication to the patient from clinicians (*personal contact*) appeared to directly affect their experiences of the pathway. Most London patients sat down with their consultant and reviewed their test results together:

"I think it was interesting to see this sort of slightly darker little, ti… little circular area that he thought might be cancerous and… and also explain that they would need to take some samples from another area which… which was more the normal colour of the whole gland for comparison." P13 (London, 65+)

Many patients in Devon received their results via a letter:

"Most of the letters go to the GP and I just get a copy." P23 (Devon, 65+)

Communicating the meaning (*conveying significance*) of the results of various tests along the prostate cancer diagnosis pathway was very important to help patients and their partners understand what the results mean for them as an individual:

"Yeah, so apparently, because this is mid-rank they said that if you just got the first circle, the first ones in, they probably wouldn't have done anything about it and you could have had a lot of years where you just monitor that. But because P03 was mid-stage, they said we have to do something." P03's partner (Devon, <65)

Despite most of the patients having been through the diagnostic pathway by the time of their interview, there were still some areas where they reported a limited understanding or lack of knowledge (*Gaps in understanding*) with regards to the tests they underwent and the results.

For peer review only

More patients from Devon reported these gaps, which often appeared to be a result of communication breakdown between the patient and the doctor:

> *"Umm… I think, all I know is those letters passed to and fro between the urologist and my GP, and I'm copied in on these things and there was some mention of an abnormality on the left hand side or somewhere or other on the prostate. That's all I know." P23 (Devon, 65+)*

Pathway experience

Patients entered the pathway in different ways, with varied length of time and diagnostic work-up prior to urgent suspected cancer referral (*Mixed routes*). For most patients, the prostate cancer pathway required a number of individual appointments (*Appointments burden*). This was more pronounced for patients in Devon:

> *"I had a PSA of, I think it was 4.03, which was fractionally above the four limit. Then they gave me two additional PSAs every three months, so I went back three months later did another PSA and then I think it was about 3.84. Then another one three months later was 4.08. So then I saw a urologist at Exeter and as a precaution they gave me an MRI and the MRI identified an area of concern if you like [inaudible]. Then I had a biopsy and what that identified was that the area of concern that the MRI identified, there was no cancer, but there was cancer in another area." P04 (Devon, 65+)*

Most patients in the RAPID pathway received their MRI results on the same day or soon after:

> *"so… the scan, you get the result within minutes, and even though I had to wait perhaps an hour before I actually saw the doctor but that's a lot less than three months." P05 (London, 65+)*

Whereas patients in Devon sometimes waited weeks to receive their MRI report:

For peer review only

"It took about... it took nearly a month to come through, but that's it [MRI results letter] there." P19 (Devon, 65+)

Devon patients were more often affected by administrative errors or last minute changes in plans for diagnostic testing, which created delays and caused some frustration:

"Well, I wasn't too happy about it because they'd put me back two month. You know, they let that time go, the month that... you know, I think it was five weeks or something I waited for the appointment, which was five weeks wasted." P21 (Devon, 65+)

Most patient interviewees were positive about undergoing investigations for possible prostate cancer, including blood tests and MRI (*Test acceptability*):

"I'd go for any scan, anything like that. Needles don't bother me, scans don't bother me." P21 (Devon, 65+)

GP Experiences of the prostate cancer diagnostic pathway

We identified three main themes: Imperfect information, Managing uncertainty, and Contextual influences (see figure 4).

Figure 4

Imperfect information

GPs spoke at length about the limitations of the current primary care diagnostic pathway for prostate cancer, and about having imperfect information on which to base their clinical decisions. A few GPs described a sense of inevitability about patients presenting with lower urinary tract symptoms at some point as they entered their later years (*Non-specific presentation*):

For peer review only

"It's a bit of a grey area so you're kind of waiting for patients to develop symptoms and come to see you" GP03 (Male, London, 31-40)

As described earlier, GPs experienced men refusing to have a prostate examination when prostate cancer is suspected (*Examination acceptance*). GPs reported different reasons for this, and perceived that patients may still be worried even if the prostate feels normal:

"I've had patients before who even will have got a high PSA decline, a rectal examination because they've previously had some, kind of, you know, traumatic experience or whatever." GP04 (Female, Devon, 41-50)

GPs from both regions did not hold back in sharing their opinions about the PSA blood test, and its usefulness (or lack thereof) in helping them make clinical decisions about which men to refer for further testing for possible prostate cancer (*GP test limitations*):

"I think if there's one test you could un-invent, I think PSA would be that…" GP02 (Male, Devon, 31-40)

GPs working in the NHS cannot currently order an MRI of the prostate; the request must come from a secondary or tertiary care clinician. London GPs were more likely to be positive about the concept of a prostate MRI:

"I think it will be a really useful idea" GP03 (Male, London, 31-40)
"Well, it's great, but it's not available to me. It's not something I decide on." GP05 (Female, Devon, 41-50)

Managing uncertainty

GPs made efforts to share their diagnostic dilemma with patients where possible, and consulted guidelines and their local urology specialists in managing uncertainty in their decisions about

For peer review only

which men to refer to secondary care. Prior to referral, GPs tried to make their patients understand the limitations of the current diagnostic pathway (*counselling patient*):

> *"But I always would tell patients that it's not 100% and that both my examinations, whether it's a digital rectal or a PSA, are not 100% and it can be raised even without having cancer." GP03 (Male, London, 31-40)*

Whilst most GPs reported feeling satisfied with their local urology service (see *health service & guidance* above), some Devon GPs reported inconsistencies in the advice and management plans for their patients that came back from hospital specialists (*seeking advice*):

> *"I mean, we try to follow the guidelines but, as I say, we find mystifying as to the variation in the urology advice that comes back in terms of who to follow and who not to…" GP04 (Female, Devon, 41-50)*

<u>Contextual influences</u>

A spectrum of broader influences had an effect on when patients chose to present to their GP with concerns about prostate cancer, and the consultation itself (*Gender, society & culture*). Some GPs noted a reticence of men to seek healthcare:

> *"I think men don't... it's such a sweeping statement but men don't like coming to the doctor" GP07 (Female, London, 31-40)*

Consistent with the patient interviews (*outside influences*), the GPs reported that it was often the wives and partners encouraging male patients to seek help and advice:

> *"…the majority of men I see who mention prostate cancer it's because their wives have asked them to come and they're worried." GP07 (Female, London 31-40)*

For peer review only

Cultural and ethnic norms relating to the patient and their partners also influenced the consultation and acceptance of prostate examination, which were more commonly reported by GPs working in London:

> *"And over here I notice there are some patients of south Indian descent where, it's [DRE] almost like a taboo really." GP03 (Male, London, 31-40)*

GPs in both regions were aware of the influence of news and media stories relating to prostate cancer that were encouraging patients with symptoms or concerns to see their GP and get tested:

> *"…there was a lot in the media recently with prostate and testicular cancer, actually which is a good thing, because we had a… I had suddenly quite a few men coming in requesting the blood test." GP09 (Female, London, 41-50)*

Some GPs felt that most patients were aware of prostate cancer and that tests were available for it. Awareness of MRI of the prostate was lower than for the PSA blood test (*Patient awareness*):

> *"Lots of people are aware of the PSA" GP07 (Female, London, 31-40)*
> *"I think a few of them might have said, "I've heard there's a new test around." I don't think anyone's come in and said, "I'd like to have that MRI test." GP04 (Female, Devon, 41-50)*

The decision-making of GPs was also affected by their own experiences in their personal and professional lives (*Personal & professional experience*). GPs demonstrated an awareness of how these experiences shaped their approach:

> *"…my dad has prostate cancer that was picked up with a raised PSA. And my stepfather has prostate cancer which was picked up by a raised PSA. Both completely asymptomatic. So I think that also affects how you... how you practice and you know, as clinicians we do take on our life experiences and we can't help but have that shape how... how we work." GP07 (Female, London, 31-40)*

For peer review only

The health service context in which GPs practise was another significant influence on their approach to patients with possible prostate cancer (*Health services & guidelines*). They often rely on guidance from a number of sources, including national guidelines and local urology services:

> *"I think we've got some, you know, very good local colleagues who offer good pragmatic advice and are very approachable."* GP02 (Male, Devon, 31-40)

For peer review only

Discussion

Principal findings

Patients' experiences of different prostate cancer diagnostic pathways incorporating MRI showed some key similarities and differences. The communication between patients and healthcare teams significantly affected the patients' overall experience and their understanding of test results, and both patients and their clinicians appeared hesitant to talk directly about cancer. Compared to patients attending a 'one-stop' clinic, patients following more traditional diagnostic pathways felt that longer waits for tests, more appointments to attend, and increased travel requirements all impacted on their pathway experience. GPs faced challenges in dealing with uncertainty and the perceived limitations of symptoms, examination and tests available to them for diagnosing prostate cancer with confidence. GP awareness, understanding and access relating to MRI was limited in both regions, and they reported some variation in local guidelines and specialist urology advice.

Strengths and weaknesses

A range of views and experiences of two prostate cancer diagnostic pathways were obtained in this study, via purposively recruiting participants with a range of ages, genders and geographical locations across two English regions. The researchers adopted a constructivist approach on the assumption that patient and GP participants' views will be based on their individual experiences and the socio-cultural context[6,7], and this approach appears to be supported by the data collected.

The influence of the researcher on data collection and analysis is important to consider in qualitative research. SM conducted all the interviews for this study. Participants were aware that SM was a clinician, and that may have given some level of respectability and authority to the interviewer and the study. Some patients and GPs reported that men were less comfortable seeing a female GP about problems relating to the prostate, so having a male interviewer may have helped patient participants be more comfortable and open in the interviews. GP participants may have been more comfortable in talking to a peer in these interviews; peer discussions are a

For peer review only

common part of professional practice for GPs in the form of Balint groups[15] and annual appraisal by a fellow GP[16].

MRI is a new test for prostate cancer, and has only recently been integrated into diagnostic pathways. GPs are not currently able to request an MRI of the prostate, and access to MRI for other indications varies across the NHS. Some GPs were reluctant to engage in any discussion about prostate MRI as they felt it was outside their current scope of practice, and may have been focused on the more traditional (pre-MRI) prostate cancer pathway.

Relation to published literature

This is the first study the authors are aware of to explore experiences of the modern pre-biopsy MRI prostate cancer diagnostic pathway from the perspective of patients and GPs. Similar studies with patients or GPs have been conducted before MRI emerged as a diagnostic test for prostate cancer. Ruseckaite *et al* interviewed 10 GPs from metropolitan Melbourne and a regional part of Victoria, Australia regarding their perceptions of prostate cancer care. In line with the findings of this study, most men were willing to have a PSA blood test, and some GPs had to grapple with inconsistent guidance from specialist bodies[10]. Evans *et al* assessed men's experience of PSA testing in primary care in Wales, and also found that social networks and media stories influenced patient demand for testing. In contrast to the views of GPs in this study, the men in the study by Evans *et al* felt decision-making about testing was doctor-centred rather than shared or patient-centred[11].

Delivering test results occurs in the latter stages of the diagnostic pathway. Some patient interviewees felt that they preferred to receive and discuss their test results in person with their doctor rather than over the telephone or via a letter, which is consistent with other literature in the field[12]. The language used in these consultations is key to avoid miscommunication or misinterpretation about the results and their significance[13]. Doctors have a tendency to communicate in a less open manner when delivering bad news to patients[14]; however in this study it appeared both doctors and patients had a hesitancy to discuss openly the possibility of a prostate cancer diagnosis.

For peer review only

Implications for clinicians and health service design

Men's experiences of the prostate cancer diagnostic pathway are influenced by the appointment burden they face to receive a diagnosis; the mode of communication used by GPs and specialists to communicate test results; and requirements for travel to attend clinic appointments and tests. Significant challenges remain for GPs owing to the limitations of the current clinical signs and tests they rely on to identify possible prostate cancer cases. Men seemed broadly positive about MRI as a new test for prostate cancer, whereas GPs were equivocal owing to a lack of awareness and access.

For peer review only

Funding

SWDM is supported by the Can Test Collaborative, which is funded by CRUK (C8640/A23385). FMW and WH are co-directors of CanTest. HA's research is supported by core funding from the United Kingdom's National Institute of Health Research (NIHR) Imperial Biomedical Research Centre. Ahmed currently receives funding from the Wellcome Trust, Medical Research Council (UK), Cancer Research UK, Prostate Cancer UK, National Institute for Health Research (UK), The Urology Foundation, BMA Foundation, Imperial Health Charity, NIHR Imperial BRC, Sonacare Inc., Trod Medical and Sophiris Biocorp for trials in prostate cancer. DE-E receives funding from the Urology Foundation, the BMA Foundation for Medical Research, Imperial Health Charity and the Royal College of Surgeons of England.

Competing interests

HA was a paid medical consultant for Sophiris Biocorp in the previous 3 years. The remaining authors have no conflicts of interest to declare.

Acknowledgements

The authors would like to thank the participants for sharing their time and experiences for this study. We would also like to thank Pauline Sibley and Victor Mariano (Royal Devon & Exeter NHS Foundation Trust) for their assistance with patient recruitment.

Contributors

SWDM, WH and FMW conceived the study. SWDM developed the research protocol with contributions from FMW, AF, and SA. HA, JM and DE-E were local investigators who supervised recruitment into the study. SWDM undertook all interviews. SWDM, FMW, AF and SA performed the analysis. SWDM drafted the first version of the manuscript. All authors contributed to the development of the manuscript, and approved the final submitted version.

Ethics approval

Ethics committee approval was received from the NHS HRA South-West Frenchay research ethics committee (REC reference 19/SW/0040).

For peer review only

Patient consent for publication

Patient consent for publication of anonymised individual remarks was obtained at the time of recruitment into the study.

Data sharing statement

All data requests should be submitted in writing to the corresponding author

Disclaimer

The views expressed in this publication are those of the author(s) and not necessarily those of the NIHR or the Department of Health and Social Care.

For peer review only

References

1 NICE. Prostate Cancer: Diagnosis and Management. ng131. 2019. doi:10.1097/00005392-200308000-00097

2 Merriel SWD, Walter FM, Hamilton W. Integrating multiparametric MRI into prostate cancer diagnostic pathways within the NHS and internationally – A diagnostic pathways mapping study. In: *Cancer REsearch UK 5th Biennial Early Diagnosis Research Conference*. Birmingham: : Cancer Research UK 2019. https://www.cancerresearchuk.org/health-professional/diagnosis/early-diagnosis-initiative/early-diagnosis-research-conferences/2019-conference-programme-and-registration

3 Fraser J, Pearson C, Shelton J, *et al.* Using linked English cancer registration data to assess variation in diagnostic pathway length. *Int J Popul Data Sci* 2019;**4**:121.

4 Sandager M, Jensen H, Lipczak H, *et al.* Cancer patients' experiences with urgent referrals to cancer patient pathways. *Eur J Cancer Care (Engl)* 2019;**28**:1–11. doi:10.1111/ecc.12927

5 Silverman D. *Doing qualitative research*. Sage 2013.

6 Guba EG, Lincoln YS. Competing paradigms in qualitative research. In: *Handbook of Qualitative Resarch*. 1994. 105–17.

7 Denzin NK, Lincoln YS. *The sage handbook of qualitative research*. Sage 2011.

8 Braun V, Clarke V. Using thematic analysis in psychology. *Qual Res Psychol* 2006;**3**:77–101. doi:10.1111/j.1460-2466.1978.tb01621.x

9 Tong A, Sainsbury P, Craig J. Consolidated criterion for reporting qualitative research (COREQ): a 32- item checklist for interviews and focus group. *Int J Qual Heal Care* 2007;**19**:349–57. doi:10.1093/intqhc/mzm042

10 Ruseckaite R, Evans S, Millar J, *et al.* GPs' Insights into Prostate Cancer Diagnosis and Care in Regional Victoria, Australia. *Qual Rep* 2016;**21**:2365–79.papers3://publication/uuid/177704E7-F6A4-40ED-9865-AFA4880DA2CB

11 Evans R, Edwards AGK, Elwyn G, *et al.* It's a maybe test': men's experiences of prostate specific antigen testing in primary care. *Br J Gen Pract* 2007;**57**:303–10.

12 Brown VA, Parker PA, Furber L, *et al.* Patient preferences for the delivery of bad news - the experience of a UK Cancer Centre. *Eur J Cancer Care (Engl)* 2011;**20**:56–61. doi:10.1111/j.1365-2354.2009.01156.x

For peer review only

13 Partin MR, Lillie SE, White KM, *et al.* Similar perspectives on prostate cancer screening value and new guidelines across patient demographic and PSA level subgroups: A qualitative study. *Heal Expect* 2017;**20**:779–87. doi:10.1111/hex.12517

14 Furber L, Cox K, Murphy R, *et al.* Investigating communication in cancer consultations: What can be learned from doctor and patient accounts of their experience? *Eur J Cancer Care (Engl)* 2013;**22**:653–62. doi:10.1111/ecc.12074

15 Roberts M. Balint groups: A tool for personal and professional resilience. *Can Fam Physician* 2012;**58**:2012.

16 NHS England. Appraisal. 2020.https://www.england.nhs.uk/medical-revalidation/appraisers/ (accessed 1 Apr 2020).

For peer review only

Figure 1 – South West Prostate Cancer Diagnostic Pathway, NHS Cancer Alliances in South-West Peninsula and Somerset, Wiltshire, Avon & Gloucester (SWAG)

mpMRI – multiparametric magnetic resonance imaging; TRUS – transrectal ultrasound guided biopsy; MDT – multidisciplinary team

Figure 2 – RAPID pathway, Imperial College Healthcare NHS Trust, London

2WW – Two Week Wait pathway; mpMRI – multiparametric magnetic resonance imaging; MRI – magnetic resonance imaging

Figure 3 – Thematic diagram from patient participant interviews

Figure 4 – Thematic diagram from GP participant interviews

For peer review only

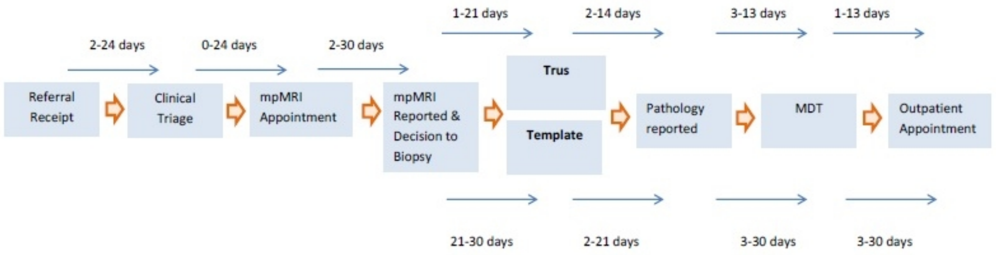

Figure 1 – South West Prostate Cancer Diagnostic Pathway, NHS Cancer Alliances in South-West Peninsula and Somerset, Wiltshire, Avon & Gloucester (SWAG)
mpMRI – multiparametric magnetic resonance imaging; TRUS – transrectal ultrasound guided biopsy; MDT – multidisciplinary team

207x55mm (96 x 96 DPI)

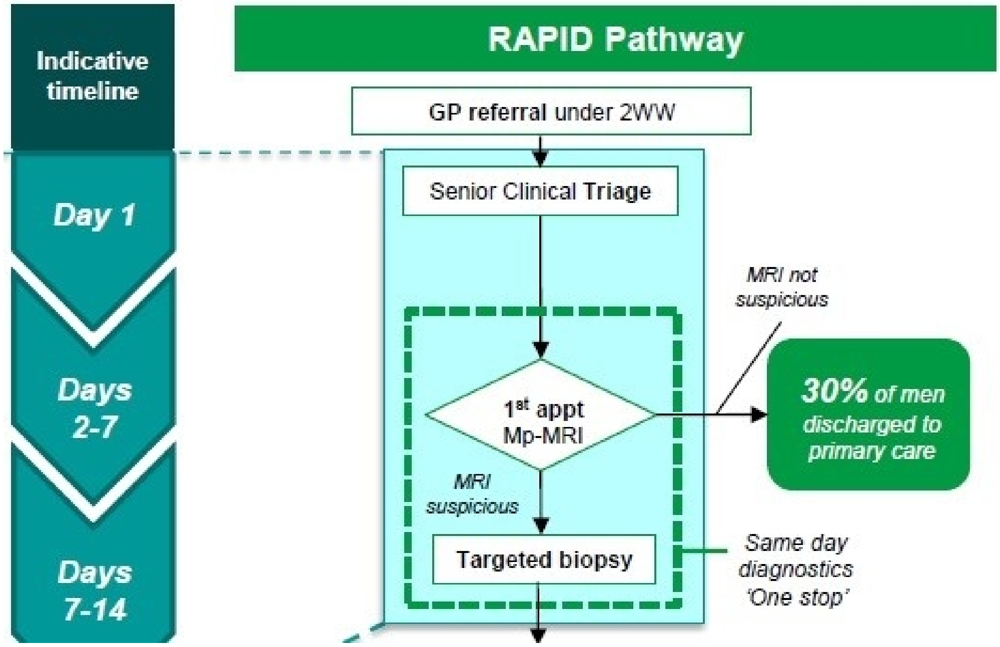

Figure 2 – RAPID pathway, Imperial College Healthcare NHS Trust, London
2WW – Two Week Wait pathway; mpMRI – multiparametric magnetic resonance imaging; MRI – magnetic resonance imaging

140x91mm (96 x 96 DPI)

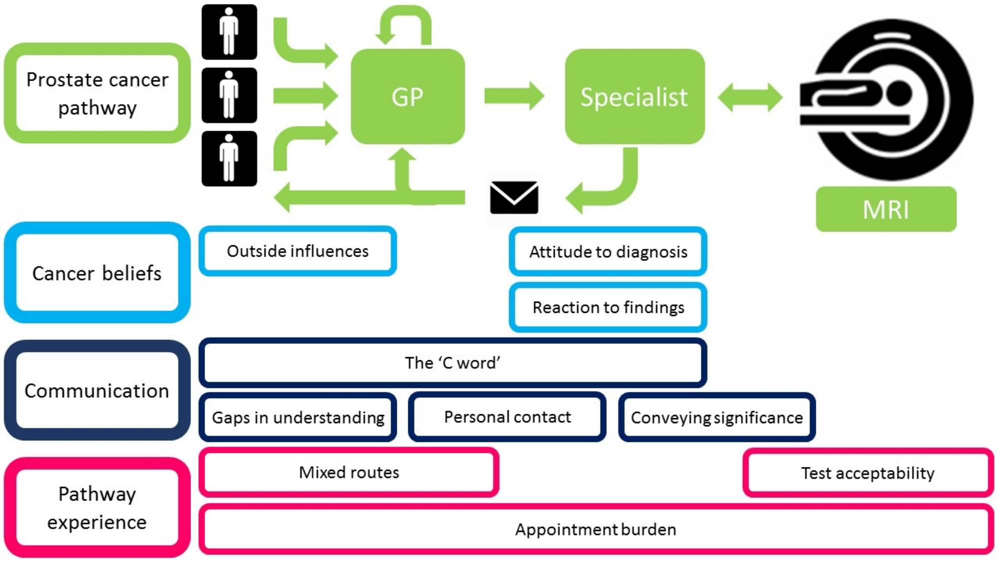

Figure 3 – Thematic diagram from patient participant interviews

338x190mm (96 x 96 DPI)

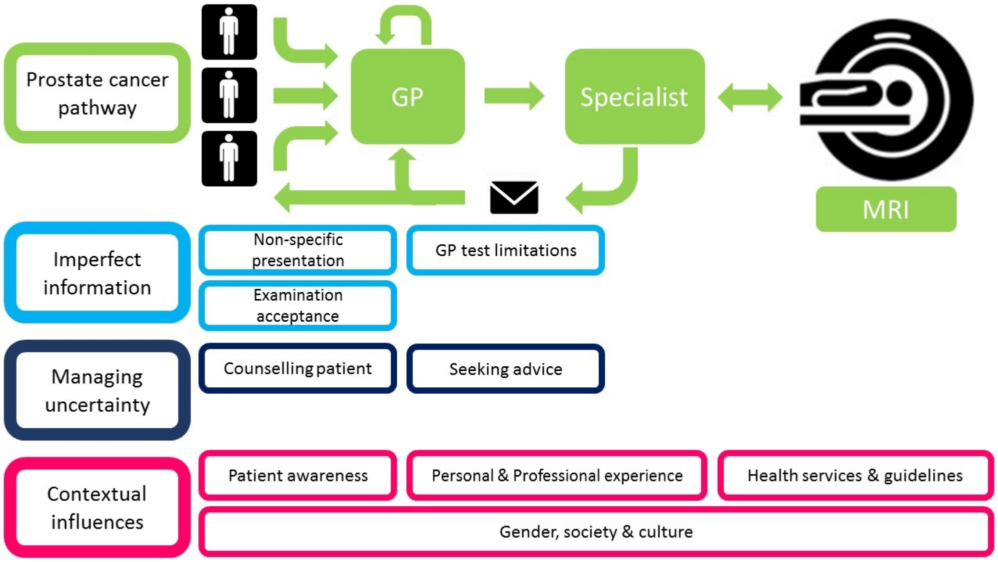

Figure 4 – Thematic diagram from GP participant interviews

338x190mm (96 x 96 DPI)

Patient participant interview guide

Introduction (5mins)

Thank you for agreeing to participate in this interview.

Introduce myself and my role.

This study seeks to understand your knowledge and understanding of diagnostic tests for prostate cancer, and your experiences of the current prostate cancer diagnostic pathway in the region where you live. It is part of my PhD at the University of Exeter. This study has been funded by Cancer Research UK, and has ethical approval from the NHS Health Research Authority and the University of Exeter.

As we've talked about with the consent form, participating in this study is voluntary and you can stop at any time. We want to know about your experiences and what you think, so there are no right or wrong answers.

This interview is being recorded for the purposes of qualitative analysis by the researchers. You can ask for the recording to be stopped at any time. What you say will be kept confidential and anonymous, unless we discuss something that suggests there is a significant risk to yourself or someone else. Everyone being interviewed will be asked the same questions, so if you don't have an answer to any of the questions that's fine, just say so and we can move on.

This interview study if focused on your experience of diagnostic test for prostate cancer. However, if you have a partner, family member or significant other who you wish to be present that's fine. Ideally we would start the interview without them, and then invite them in later on. If you and they are happy for them to participate, they would need to complete a consent form as well.

Ensure participant has copy of participant information sheet

Answer any questions

Ensure consent form is completed correctly

Commence audio recording

For peer review only

For peer review only - http://bmjopen.bmj.com/site/about/guidelines.xhtml

Basic demographics (5 minutes)

"To start with, can you tell me a little bit about yourself and your background"

Check - Age, Ethnicity, City/town lived in

Prostate cancer diagnosis journey (5-10 minutes)

"Now I would like to talk a bit about how you came to have tests for possible prostate cancer."

When did you first notice symptoms (if any)? Which symptoms were they?
How long until consulted you consulted your GP? What affected that decision?
How did GP assess? PSA? DRE?
Decision to refer – what do you remember about that discussion?

mpMRI for prostate cancer (10-15 minutes)

"I would now like to ask some questions about having an MRI scan for possible prostate cancer."

Did you have any concerns or reservations about having an MRI? What were your expectations?
What do you understand about how an MRI works?
What was your experience of having an MRI?

mpMRI result (10 minutes)

"If you are happy to discuss, I would like to ask a few questions about the results of your MRI scan and the next steps."

What was the result of your mpMRI? Did you understand it? How much did you trust the findings?
How were the results communicated? Did you have any questions as a result?
What were you told about the results' meaning?
What else did you discuss with the specialist?

Doctor(s) involved in cancer diagnosis (5 minutes)

"Finally, I would like to ask about your thoughts regarding the way we organise testing for prostate cancer."

What works well? What could be done better?
Who do you feel should be involved in investigating for a possible cancer diagnosis?
If you could design the ideal cancer diagnosis testing service, how would it work?

Interview close (2 minutes)

Thank you for participating in this interview. The data you have provided will be transcribed under a pseudonym, and analysed by the research team. You will be sent a final study report after the analysis has been completed.

If you have any questions or concerns about the study, please contact Ms Pam Baxter at the Research Ethics and Governance Office at the University of Exeter on 01392 723588 or via email p.r.baxter2@exeter.ac.uk Her details are on your patient information leaflet.

For peer review only

For peer review only - http://bmjopen.bmj.com/site/about/guidelines.xhtml

GP participant interview guide

Introduction (5mins)

Thank you for agreeing to participate in this interview.

Introduce myself and my role.

This study seeks to understand your knowledge and understanding of diagnostic tests for prostate cancer, and your experiences of the current prostate cancer diagnostic pathway in the region where you work. It is part of my PhD at the University of Exeter. This study has been funded by Cancer Research UK, and has ethical approval from the NHS Health Research Authority and the University of Exeter.

As we've talked about with the consent form, participating in this study is voluntary and you can stop at any time. We want to know about your experiences and what you think, so there are no right or wrong answers.

This interview is being recorded for the purposes of qualitative analysis by the researchers. You can ask for the recording to be stopped at any time. What you say will be kept confidential and anonymous, unless we discuss something that suggests there is a significant risk to yourself or someone else. This interview is not assessing your clinical competence, and we want to hear about your approach and experiences. Everyone being interviewed will be asked the same questions, so if you don't have an answer to any of the questions that's fine, just say so and we can move on.

This interview study if focused on your experience of diagnostic test for prostate cancer. However, if you have a partner, family member or significant other who you wish to be present that's fine. Ideally we would start the interview without them, and then invite them in later on. If you and they are happy for them to participate, they would need to complete a consent form as well.

Ensure participant has copy of participant information sheet

Answer any questions

Ensure consent form is completed correctly

Commence audio recording

For peer review only

For peer review only - http://bmjopen.bmj.com/site/about/guidelines.xhtml

Basic demographics (5 minutes)

"To start with, can you tell me a little bit about yourself and your background"

Age, Gender, Years of GP experience, Main CCG area you work in

Decision to refer for suspected prostate cancer (10-15 minutes)

"I would like to now move on to your current practice around referring men with suspected prostate cancer for further investigation"

"What symptoms/signs do you enquire about when assessing a man for suspected prostate cancer? How do they affect your decision to refer?"

PSA use – When would you offer it to a man? What are the important points you make about PSA when counselling a man about the test? What do you do with a negative PSA?

"What other factors, if any, affect your decision to refer a man for further investigation?"

"What are the key points you discuss with men when making a referral?"

Diagnostic testing for prostate cancer (15-20 minutes)

"Now I would like to ask some questions about diagnostic tests for prostate cancer."

What is your experience of the prostate cancer diagnosis pathway in your region?
What do you know about current diagnostic tests? How accurate do you believe current diagnostic tests are for prostate cancer?
PROMIS trial – have you heard of it? Are you aware of use of mpMRI for prostate cancer?
Do you feel incorporating mpMRI into the prostate cancer diagnosis pathway would be beneficial for patients? Do you believe it could be cost effective?
What would be the characteristics of an ideal diagnostic test for prostate cancer?

Men diagnosed with prostate cancer (5-10 minutes)

"Finally, I would like to ask about any of your patients who have been diagnosed with prostate cancer"

Are you aware of any of your patients diagnosed with low-grade prostate cancer?
If so, what has been the impact of the diagnosis on patient?
Are you aware of any of your patients being put on active surveillance – what is your experience of interacting with these men after diagnosis?

Interview close (2 minutes)

Thank you for participating in this interview. The data you have provided will be transcribed under a pseudonym, and analysed by the researchers. You will be sent a final study report after the analysis has been completed. If you have any questions or concerns about the study, please contact Ms Pam Baxter at the Research Ethics and Governance Office at the University of Exeter on 01392 723588 or via email p.r.baxter2@exeter.ac.uk

For peer review only - http://bmjopen.bmj.com/site/about/guidelines.xhtml

Consolidated criteria for reporting qualitative studies (COREQ): 32-item checklist

Developed from:
Tong A, Sainsbury P, Craig J. Consolidated criteria for reporting qualitative research (COREQ): a 32-item checklist for interviews and focus groups. *International Journal for Quality in Health Care*. 2007. Volume 19, Number 6: pp. 349 – 357

No. Item	Guide questions/description	Reported on Page #
**Domain 1: Research team and reflexivity**		
Personal Characteristics		
1. Inter viewer/facilitator	Which author/s conducted the inter view or focus group?	7
2. Credentials	What were the researcher's credentials? E.g. PhD, MD	1
3. Occupation	What was their occupation at the time of the study?	19
4. Gender	Was the researcher male or female?	19
5. Experience and training	What experience or training did the researcher have?	1
Relationship with participants		
6. Relationship established	Was a relationship established prior to study commencement?	N/A
7. Participant knowledge of the interviewer	What did the participants know about the researcher? e.g. personal goals, reasons for doing the research	19
8. Interviewer characteristics	What characteristics were reported about the inter viewer/facilitator? e.g. Bias, assumptions, reasons and interests in the research topic	19
**Domain 2: study design**		
Theoretical framework		
9. Methodological orientation and Theory	What methodological orientation was stated to underpin the study? e.g. grounded theory, discourse analysis, ethnography, phenomenology, content analysis	6
Participant selection		
10. Sampling	How were participants selected? e.g. purposive, convenience, consecutive, snowball	6
11. Method of approach	How were participants approached? e.g. face-to-face, telephone, mail, email	6-7
12. Sample size	How many participants were in the study?	9
13. Non-participation	How many people refused to participate or dropped out? Reasons?	9

For peer review only

Setting		
14. Setting of data collection	Where was the data collected? e.g. home, clinic, workplace	7
15. Presence of non-participants	Was anyone else present besides the participants and researchers?	9
16. Description of sample	What are the important characteristics of the sample? e.g. demographic data, date	9
Data collection		
17. Interview guide	Were questions, prompts, guides provided by the authors? Was it pilot tested?	7
18. Repeat interviews	Were repeat inter views carried out? If yes, how many?	N/A
19. Audio/visual recording	Did the research use audio or visual recording to collect the data?	7
20. Field notes	Were field notes made during and/or after the inter view or focus group?	7
21. Duration	What was the duration of the inter views or focus group?	7
22. Data saturation	Was data saturation discussed?	7
23. Transcripts returned	Were transcripts returned to participants for comment and/or correction?	8
**Domain 3: analysis and findings**		
Data analysis		
24. Number of data coders	How many data coders coded the data?	8
25. Description of the coding tree	Did authors provide a description of the coding tree?	8
26. Derivation of themes	Were themes identified in advance or derived from the data?	8
27. Software	What software, if applicable, was used to manage the data?	NVivo
28. Participant checking	Did participants provide feedback on the findings?	18
Reporting		
29. Quotations presented	Were participant quotations presented to illustrate the themes/findings? Was each quotation identified? e.g. participant number	10-18
30. Data and findings consistent	Was there consistency between the data presented and the findings?	10-18
31. Clarity of major themes	Were major themes clearly presented in the findings?	10-18
32. Clarity of minor themes	Is there a description of diverse cases or discussion of minor themes?	10-18

For peer review only

Experiences of ‘traditional’ and ‘one-stop’ MRI-based prostate cancer diagnostic pathways in England: a qualitative study with patients and GPs

Journal:	*BMJ Open*
Manuscript ID	bmjopen-2021-054045.R1
Article Type:	Original research
Date Submitted by the Author:	13-Nov-2021
Complete List of Authors:	Merriel, Samuel; University of Exeter, Institute of Health Research<br>Archer, Stephanie; University of Cambridge School of Clinical Medicine, Public Health and Primary Care; University of Cambridge<br>Forster, Alice; UCL, Behavioural Science and Health<br>Eldred-Evans, David; Imperial College London, Imperial Prostate<br>McGrath, John; Royal Devon and Exeter NHS Foundation Trust, Urology<br>Ahmed, Hashim; Imperial College London, Imperial Prostate<br>Hamilton, Willie; University of Exeter, Peninsula College of Medicine and Dentistry<br>Walter, Fiona; Queen Mary University of London, Wolfson Institute of Public Health; University of Cambridge, Department of Public Health and Primary Care
**Primary Subject Heading**:	Qualitative research
Secondary Subject Heading:	Urology, General practice / Family practice
Keywords:	PRIMARY CARE, QUALITATIVE RESEARCH, Prostate disease < UROLOGY, Urological tumours < ONCOLOGY, Magnetic resonance imaging < RADIOLOGY & IMAGING

SCHOLARONE™ Manuscripts

I, the Submitting Author has the right to grant and does grant on behalf of all authors of the Work (as defined in the below author licence), an exclusive licence and/or a non-exclusive licence for contributions from authors who are: i) UK Crown employees; ii) where BMJ has agreed a CC-BY licence shall apply, and/or iii) in accordance with the terms applicable for US Federal Government officers or employees acting as part of their official duties; on a worldwide, perpetual, irrevocable, royalty-free basis to BMJ Publishing Group Ltd ("BMJ") its licensees and where the relevant Journal is co-owned by BMJ to the co-owners of the Journal, to publish the Work in this journal and any other BMJ products and to exploit all rights, as set out in our licence.

The Submitting Author accepts and understands that any supply made under these terms is made by BMJ to the Submitting Author unless you are acting as an employee on behalf of your employer or a postgraduate student of an affiliated institution which is paying any applicable article publishing charge ("APC") for Open Access articles. Where the Submitting Author wishes to make the Work available on an Open Access basis (and intends to pay the relevant APC), the terms of reuse of such Open Access shall be governed by a Creative Commons licence – details of these licences and which Creative Commons licence will apply to this Work are set out in our licence referred to above.

Other than as permitted in any relevant BMJ Author's Self Archiving Policies, I confirm this Work has not been accepted for publication elsewhere, is not being considered for publication elsewhere and does not duplicate material already published. I confirm all authors consent to publication of this Work and authorise the granting of this licence.

For peer review only

Experiences of 'traditional' and 'one-stop' MRI-based prostate cancer diagnostic pathways in England: a qualitative study with patients and GPs

For peer review only

Dr Samuel W D Merriel MSc, University of Exeter Medical School, University of Exeter, UK

s.w.d.merriel@exeter.ac.uk

Dr Stephanie Archer PhD, Department of Public Health and Primary Care, University of Cambridge, UK

saa71@medschl.cam.ac.uk

Dr Alice Forster PhD, Institute of Epidemiology & Health, University College London, UK

alice.forster@ucl.ac.uk

Dr David Eldred-Evans PhD, Imperial Prostate, Department of Surgery & Cancer, Imperial College London, UK; Imperial Urology, Charing Cross Hospital, Imperial College Healthcare NHS Trust, London, UK

d.eldred-evans@imperial.ac.uk

Mr John McGrath FRCS, Department of Urological Surgery, Royal Devon & Exeter NHS Foundation Trust, UK

john.mcgrath4@nhs.net

Prof Hashim U. Ahmed FRCS, Imperial Prostate, Department of Surgery & Cancer, Imperial College London, UK; Imperial Urology, Charing Cross Hospital, Imperial College Healthcare NHS Trust, London, UK

Hashim.ahmed@imperial.ac.uk

Prof Willie Hamilton MD, University of Exeter Medical School, University of Exeter, UK

w.hamilton@exeter.ac.uk

Dr Fiona M Walter MD, Wolfson Institute of Population Health, Queen Mary University of London, UK, & The Primary Care Unit, Department of Public Health and Primary Care, University of Cambridge, UK fiona.walter@qmul.ac.uk

Corresponding author

Dr Samuel W D Merriel

1.18 College House, St Luke's campus, University of Exeter

Heavitree Road, Exeter, UK EX1 2LU

+441392 726002

s.w.d.merriel@exeter.ac.uk

Keywords: Prostate cancer, diagnostic pathway, primary care

For peer review only

Abstract

Objectives

This study aimed to understand and explore patient and GP experiences of 'traditional' and 'one-stop' prostate cancer diagnostic pathways in England.

Design

Qualitative study using semi-structured interviews, analysed using inductive thematic analysis

Setting

Patients were recruited from National Health Service (NHS) Trusts in London and in Devon; GPs were recruited via National Institute for Health Research (NIHR) Clinical Research Networks. Interviews were conducted in person or via telephone.

Participants

Patients who had undergone a magnetic resonance imaging (MRI) scan of the prostate as part of their diagnostic work-up for possible prostate cancer, and GPs who had referred at least one patient for possible prostate cancer in the preceding 12 months.

Results

22 patients (aged 47 – 80 years) and 10 GPs (6 female, aged 38 – 58 years) were interviewed. Patients described three key themes: cancer beliefs in relation to patient's attitudes towards prostate cancer; communication with their GP and specialist having a significant impact on experience of the pathway; and pathway experience being influenced by appointment and test burden. GP interview themes included: the challenges of dealing with imperfect information in the current pathway; managing uncertainty in identifying patients with possible prostate cancer, and sharing this uncertainty with them, and other social, cultural and personal contextual influences.

Conclusions

Patients and GPs reported a range of experiences and views of the current prostate cancer diagnostic pathways in England. Patients valued 'one-stop' pathways integrating prostate MRI and diagnostic consultations with specialists over the more traditional approach of several hospital appointments. GPs remain uncertain how best to identify patients needing referral for urgent prostate cancer testing due to the lack of accurate triage and risk assessment strategies.

For peer review only

Article summary

Strengths and limitations

- Patient experiences of two very different prostate cancer diagnostic pathways compared and contrasted
- Patient sample feature a broad range of ages, geographical regions, and cancer investigation journeys to generate rich data
- First study to explore GP experience and understanding of new prostate cancer diagnostic pathways incorporating magnetic resonance imaging (MRI)
- Limited knowledge of prostate MRI curtailed interviews with some GP participants

For peer review only

Introduction

Patient experience of healthcare has developed as an important marker of quality of care in recent decades. However, measuring and understanding patient experience of diagnostic pathways and services is underexplored and poorly prioritised compared to other aspects of healthcare such as access or treatments[1]. Assessment of the impact of variations in pathway design between health services may also identify elements associated with better patient experiences that could be implemented more widely.

Cancer diagnostic pathways are prioritised for urgent access to diagnostic tests in many healthcare systems as early stage diagnosis is associated with increased survival[2]. Not only do shorter diagnostic intervals improve outcomes for patients, but patients also report better experiences of care[3]. The National Health Service (NHS) in England has a Two Week Wait (2WW) urgent cancer referral pathway system, where any patient with symptoms or signs of a potential undiagnosed cancer referred by their General Practitioner (GP) should have a specialist review for further investigation within two weeks[4]. Significant variation in cancer diagnostic pathways between NHS Trusts and regions in England exists, most markedly for prostate cancer[5]. Identifying patients for 2WW prostate cancer referral in primary care is also challenging for GPs owing to limitations of existing tests, including prostate specific antigen (PSA), which can impact on doctor-patient communication and patient experience of the early stages of the prostate cancer diagnostic pathway[6,7].

National Institute for Health and Care Excellence (NICE) guidance for diagnosing prostate cancer in England was updated in 2019 to recommend pre-biopsy magnetic resonance imaging (MRI) for men suspected of having prostate cancer[8]. In response, Cancer Alliances and Hospital Trusts in the NHS have updated local prostate cancer diagnostic pathways, with significant variation in the implementation of MRI[9]. Despite the potential benefits prostate MRI brings in terms of more accurate prostate cancer diagnosis[10], adding further testing into the prostate cancer diagnostic pathway could lengthen the diagnostic interval, adversely impacting patient experience. Experiences of the prostate cancer diagnostic pathway for patients and GPs since the advent of prostate MRI is unknown. The aim of this study was to elicit the experience of patients and GPs

For peer review only

following two prostate cancer diagnostic pathways that incorporate pre-biopsy MRI in different ways. In the context of the Model of Pathways to Treatment, a key theoretical framework in cancer diagnostic pathways, this study focuses on the 'Help-seeking' and 'Diagnostic' intervals and explores the perspectives of both patient and clinician[11,12].

For peer review only

Methods

This qualitative study used semi-structured interviews to explore the experiences of patients referred from primary care with possible prostate cancer who had undergone an MRI, and GPs who have referred men with possible prostate cancer for further investigation. A constructivist approach was adopted to access the data and understand the experiences of patients and GPs[13] based on their individual experiences (past and present) and the socio-cultural context[14,15].

Participants

This study recruited participants from two populations;

- Patients with possible prostate cancer who had undergone an MRI as part of their diagnostic workup.
- GPs who had referred at least one patient for investigation for possible prostate cancer within the preceding 12 months.

Patients who were undergoing MRI for active surveillance or watchful waiting for a previously diagnosed prostate cancer were not eligible, as the focus of this study was on the role of MRI in the diagnosis of prostate cancer rather than management.

Recruitment

Patients were recruited from two NHS Trusts in England: the Royal Devon & Exeter NHS Foundation Trust in Exeter and the Imperial College Healthcare NHS Trust in London. The Royal Devon & Exeter Hospital use separate outpatient appointments in the South West (SW) Prostate Cancer Diagnostic Pathway for a prostate MRI, consultant review, and prostate biopsy (if required), as shown in Figure 1. Imperial College employ the RAPID pathway, where patients undergo a prostate MRI scan, receive their MRI result, and potentially undergo a prostate biopsy on the same day at a single outpatient attendance (see Figure 2). These Trusts were selected as prostate MRI has been implemented in very different ways, creating the opportunity to explore and compare patient and clinician experiences in different clinical contexts. Research staff at the Trusts identified potentially eligible men and contacted them within days of undergoing an MRI to discuss this study and offer the men a Patient Information Leaflet (PIL). The lead investigator and local recruitment leads were in regular contact throughout recruitment to identify any under-

For peer review only

represented groups of men and focus recruitment where needed. Travel costs for patient participants to attend a face to face interview were reimbursed, and participants were also offered a gift voucher in recognition of contributing their time.

Figure 1

Figure 2

GPs were recruited through two National Institute for Health Research (NIHR) Clinical Research Networks (CRNs) in the same regions as the hospital sites: North West London CRN and the South-West Peninsula CRN. The CRNs promoted the study to local practices, and GPs expressed their interest to the CRNs. Eligibility and basic demographics were checked to assist with purposive sampling. GPs chosen for invitation into the study were given a PIL to review prior to the arrangement of an interview. GP practices were reimbursed for the GP's time to participate in the study.

A purposive sampling approach was used, in order to obtain a diverse group of participants with a wide range of geographical locations, ages, genders (GPs) and MRI results (patients).

Data collection

One-to-one interviews were conducted with all participants in this study between July and November 2019 by SM (a male GP). Patient participants were either interviewed face-to-face in their own home or via telephone, while all GP participant interviews were conducted via telephone. Formal written consent was obtained from all participants, and patient's partners if present (n=2), prior to commencement of the interview. A semi-structured approach was followed, with separate interview topic guides for patient and GP interviews to support discussions (See supplementary file 1 and 2). The topic guide was developed to incorporate all aspects of the revised prostate cancer diagnostic pathway, and was used flexibly within the interviews to ensure that no key aspects of the diagnostic pathway experience were missed. An

For peer review only

encrypted audio recording device was employed to record all interviews, and written notes were taken during and immediately following the interviews. Interview times ranged between 15-45 minutes each. Interview recordings were transferred securely to an independent transcribing service, and transcribed verbatim.

Data analysis

An inductive thematic analysis was conducted to understand the experiences of participants[16], using the conceptual framework of the Model of Pathways to Treatment[11,12]. The researchers initially immersed themselves in the data through reading and re-reading individual transcripts and listening back to the audio recordings of the interviews. A selection of early interviews were coded, and this initial coding framework was reviewed and refined by SM, SA and FW. The remaining interview transcripts were coded inductively from the entirety of the data. The codes were reviewed and arranged into themes through an iterative process, returning to the original data as needed. Patient and GP transcripts were analysed separately. Within and between themes, the experiences of participants following different diagnostic pathways were compared and contrasted. Recruitment ceased when no new themes emerged in analysis. Transcripts were imported into NVivo v12 to manage the data for the analysis. A study summary report was sent to all study participants after completion of data analysis.

Patient & Public (PPI) Involvement statement

Eight men were recruited via the People in Health West of England (PHWE) initiative to contribute to the research: these men had a range of ages, locations, ethnic backgrounds and experiences with prostate cancer. PPI group members reviewed the plain English summary and all patient participant documents, and gave feedback prior to submission as part of the ethical approval application. PPI group members also gave input into the interview topic guides and the expected burden of involvement for participants. One of the anonymised patient interview transcripts was shared with the group at a meeting, and discussed to explore themes emerging from the text.

Ethical approval

For peer review only

Ethics committee approval was received from the NHS HRA South-West Frenchay research ethics committee (REC reference 19/SW/0040).

COREQ reporting guidelines

This manuscript has been written in accordance with the consolidated criterion for reporting qualitative research (COREQ) checklist[17]. Further detail regarding the methods can be found in the study protocol (see Supplementary file 3).

For peer review only

Results

Participants

Twenty-two patients were interviewed; two with their wives present and involved in the interview: participant ages ranged from 47 – 80 years. Ten GPs were interviewed: most were female (n = 6), with an age range of 38 – 58 years (See table 1). Five further potential (three patients and two GPs) participants were approached but declined to participate.

	Patients (n = 22)		GPs (n = 10)
**Age**		**Age**	
<65	8	31-40	3
65+	14	41-50	6
		50+	1
**Location**		**Location**	
London	10	London	4
Devon	12	Devon	6
**Ethnicity**		**Gender**	
White	19	Male	4
BME	3	Female	6
**PIRADS v2***		**Role**	
1-2	6	Partner	8
3-5	15	Salaried	2
Unknown	1		

Table 1 – Patient and GP demographics

* PIRADS – Prostate Imaging-Reporting And Data System v2 score of 1-2 suggest clinically significant prostate cancer is unlikely and biopsy not indicated. A PIRADS score of 3-5 indicates at least one suspicious area of the prostate that warrants biopsy.

Patient experiences of the prostate cancer diagnostic pathway

For peer review only

We identified three main themes with interlinking sub-themes (see figure 3): Cancer beliefs, Communication, and Pathway experience.

Figure 3

Cancer beliefs

The decision for patients to see their doctor about potential prostate problems was not undertaken in isolation (*Outside influences*). The experiences of family members and friends shaped the patients' expectations, and family members and partners often encouraged men to be tested:

> *"Obviously back then he [dad] was in his mid to late 60s. And I think I didn't really know about it until he'd gone for his MRI and got the results and everything, and then all of a sudden he sat me down and told me all about it." P20 (London, <65)*

Most patients' attitudes towards the possibility of a diagnosis of cancer (*Attitude to diagnosis*) were fairly relaxed. Many seemed philosophical about the prospect:

> *"it is what it is" P03 (Devon, <65)*

The reactions of patients who had a diagnosis of prostate cancer (*Reaction to findings*) were mixed, ranging from despondence to quick acceptance:

> *"Not fair. No, it's... it's not fair on... on anyone, not just me. It isn't fair on anyone." P01 (Devon, 65+)*

Communication

For peer review only

The absence of the use of the word cancer (‘*C word*’) was evident in interviews with many patients. Patients also reported a reluctance from clinicians to raise cancer specifically as a possibility during a consultation, even if they were referred for urgent tests to rule out a diagnosis of prostate cancer:

> *“And then this developed.” P01 (Devon, 65+)*
>
> *“For me, my... my dad had it roughly about eight, nine years... eight to ten years ago, I suppose. He had it.” P20 (London, <65)*
>
> *“The only thing that I found was you were given leaflets that mention a lot about cancer but no one actually really, sort of like said to me, you know, there’s a possibility that you could have cancer or you know, that you’re just being given leaflets and such, and no one really explained to you that there is a possibility. ” P25 (Devon, <65)*

The mode of communication to the patient from clinicians (*personal contact*) appeared to directly affect their experiences of the pathway. Most London patients sat down with their consultant and reviewed their MRI results together, whereas many patients in Devon received their results via a letter:

> *“I think it was interesting to see this sort of slightly darker little, ti… little circular area that he thought might be cancerous and… and also explain that they would need to take some samples from another area which… which was more the normal colour of the whole gland for comparison.” P13 (London, 65+)*
>
> *“Most of the letters go to the GP and I just get a copy.” P23 (Devon, 65+)*

Communicating the meaning (*conveying significance*) of the results of the MRI and other tests performed was very important to help patients and their partners understand what the results mean for them as an individual:

> *“Yeah, so apparently, because this is mid-rank they said that if you just got the first circle, the first ones in, they probably wouldn’t have done anything about it and you could have*

For peer review only

had a lot of years where you just monitor that. But because P03 was mid-stage, they said we have to do something." P03's partner (Devon, <65)

Despite most of the patients having undergone a prostate MRI by the time of their interview, there were still limited understanding of the MRI results for some patients (*Gaps in understanding*). More patients from Devon reported these gaps, which often appeared to be a result of communication breakdown between the patient and the doctor:

"Umm… I think, all I know is those letters passed to and fro between the urologist and my GP, and I'm copied in on these things and there was some mention of an abnormality on the left hand side or somewhere or other on the prostate. That's all I know." P23 (Devon, 65+)

Pathway experience

Patients entered the pathway in different ways, with varied length of time and diagnostic work-up prior to urgent suspected cancer referral (*Mixed routes*). For patients in Devon, the prostate cancer pathway required a number of individual appointments, whereas most patients in London received their MRI results on the same day or soon after which was well received (*Appointments burden*):

"I had a PSA of, I think it was 4.03, which was fractionally above the four limit. Then they gave me two additional PSAs every three months, so I went back three months later did another PSA and then I think it was about 3.84. Then another one three months later was 4.08. So then I saw a urologist at Exeter and as a precaution they gave me an MRI and the MRI identified an area of concern if you like [inaudible]. Then I had a biopsy and what that identified was that the area of concern that the MRI identified, there was no cancer, but there was cancer in another area." P04 (Devon, 65+)

"so… the scan, you get the result within minutes, and even though I had to wait perhaps an hour before I actually saw the doctor but that's a lot less than three months." P05 (London, 65+)

For peer review only

Patient interviewees were generally positive about undergoing investigations for possible prostate cancer, including blood tests and MRI. Most, but not all, patients reported that undergoing an MRI of the prostate was not a significant undertaking (*Test acceptability*):

> *"I'd go for any scan, anything like that. Needles don't bother me, scans don't bother me." P21 (Devon, 65+)*

GP Experiences of the prostate cancer diagnostic pathway

We identified three main themes: Imperfect information, Managing uncertainty, and Contextual influences (see figure 4).

Figure 4

Imperfect information

GPs spoke at length about the limitations of the current primary care diagnostic pathway for prostate cancer, and about having imperfect information on which to base their clinical decisions. A few GPs described a sense of inevitability about patients presenting with lower urinary tract symptoms at some point as they entered their later years (*Non-specific presentation*):

> *"It's a bit of a grey area so you're kind of waiting for patients to develop symptoms and come to see you" GP03 (Male, London, 31-40)*

As described earlier, GPs experienced men refusing to have a prostate examination when prostate cancer is suspected (*Examination acceptance*). GPs reported different reasons for this, and perceived that patients may still be worried even if the prostate feels normal:

For peer review only

"I've had patients before who even will have got a high PSA decline, a rectal examination because they've previously had some, kind of, you know, traumatic experience or whatever." GP04 (Female, Devon, 41-50)

GPs from both regions did not hold back in sharing their opinions about the PSA blood test, and its usefulness (or lack thereof) in helping them make clinical decisions about which men to refer for further testing for possible prostate cancer (*GP test limitations*):

"I think if there's one test you could un-invent, I think PSA would be that…" GP02 (Male, Devon, 31-40)

GPs working in the NHS cannot currently order an MRI of the prostate; the request must come from a secondary or tertiary care clinician. London GPs were more likely to be positive about the concept of a prostate MRI:

"I think it will be a really useful idea" GP03 (Male, London, 31-40)
"Well, it's great, but it's not available to me. It's not something I decide on." GP05 (Female, Devon, 41-50)

Managing uncertainty

GPs made efforts to share their diagnostic dilemma with patients where possible, and consulted guidelines and their local urology specialists in managing uncertainty in their decisions about which men to refer to secondary care. Prior to referral, GPs tried to make their patients understand the limitations of the current diagnostic pathway (*counselling patient*):

"But I always would tell patients that it's not 100% and that both my examinations, whether it's a digital rectal or a PSA, are not 100% and it can be raised even without having cancer." GP03 (Male, London, 31-40)

For peer review only

Whilst most GPs reported feeling satisfied with their local urology service (see *health service & guidance* below), some Devon GPs reported inconsistencies in the advice and management plans for their patients that came back from hospital specialists (*seeking advice*):

> *"I mean, we try to follow the guidelines but, as I say, we find mystifying as to the variation in the urology advice that comes back in terms of who to follow and who not to…" GP04 (Female, Devon, 41-50)*

<u>Contextual influences</u>

A spectrum of broader influences had an effect on when patients chose to present to their GP with concerns about prostate cancer, and the consultation itself (*Gender, society & culture*). Some GPs noted a reticence of men to seek healthcare:

> *"I think men don't... it's such a sweeping statement but men don't like coming to the doctor" GP07 (Female, London, 31-40)*

Consistent with the patient interviews (*outside influences*), the GPs reported that it was often the wives and partners encouraging male patients to seek help and advice:

> *"…the majority of men I see who mention prostate cancer it's because their wives have asked them to come and they're worried." GP07 (Female, London 31-40)*

Cultural and ethnic norms relating to the patient and their partners also influenced the consultation and acceptance of prostate examination, which were more commonly reported by GPs working in London:

> *"And over here I notice there are some patients of south Indian descent where, it's [DRE] almost like a taboo really." GP03 (Male, London, 31-40)*

For peer review only

GPs in both regions were aware of the influence of news and media stories relating to prostate cancer that were encouraging patients with symptoms or concerns to see their GP and get tested:

> *"…there was a lot in the media recently with prostate and testicular cancer, actually which is a good thing, because we had a… I had suddenly quite a few men coming in requesting the blood test." GP09 (Female, London, 41-50)*

GPs felt that most patients were aware of prostate cancer and that tests were available for it. Awareness of MRI of the prostate was lower than for the PSA blood test (*Patient awareness*):

> *"Lots of people are aware of the PSA" GP07 (Female, London, 31-40)*
> *"I think a few of them might have said, "I've heard there's a new test around." I don't think anyone's come in and said, "I'd like to have that MRI test." GP04 (Female, Devon, 41-50)*

The decision-making of GPs was also affected by their own experiences in their personal and professional lives (*Personal & professional experience*). GPs demonstrated an awareness of how these experiences shaped their approach:

> *"…my dad has prostate cancer that was picked up with a raised PSA. And my stepfather has prostate cancer which was picked up by a raised PSA. Both completely asymptomatic. So I think that also affects how you... how you practice and you know, as clinicians we do take on our life experiences and we can't help but have that shape how... how we work." GP07 (Female, London, 31-40)*

The health service context in which GPs practise was another significant influence on their approach to patients with possible prostate cancer (*Health services & guidelines*). They often rely on guidance from a number of sources, including national guidelines and local urology services:

> *"I think we've got some, you know, very good local colleagues who offer good pragmatic advice and are very approachable." GP02 (Male, Devon, 31-40)*

For peer review only

Discussion

Principal findings

Patients' experiences of more traditional and 'one-stop' prostate cancer diagnostic pathways incorporating MRI showed some key similarities and differences. The communication between patients and healthcare teams significantly affected the patients' overall experience and their understanding of MRI results. MRI appeared to be an acceptable and low burden test for patients. Compared to patients attending a 'one-stop' clinic, patients following more traditional diagnostic pathways felt that longer waits for tests, more appointments to attend, and increased travel requirements all impacted on their pathway experience. GPs faced challenges in dealing with uncertainty and the perceived limitations of symptoms, examination and tests available to them for diagnosing prostate cancer with confidence. GP awareness, understanding and access relating to MRI was limited in both regions, and they reported some variation in local guidelines and specialist urology advice.

Strengths and weaknesses

Participants in this study were recruited from two regions with contrasting prostate cancer diagnostic pathways in terms of number of appointments, length of diagnostic interval, and integration of prostate MRI. This enabled identification of key similarities and differences in the experiences of patients and GPs engaging with the different pathways.

The influence of the researcher on data collection and analysis is important to consider in qualitative research. Participants were aware that SM was a clinician, and that may have given some level of respectability and authority to the interviewer and the study. Some patients and GPs reported that men were less comfortable seeing a female GP about problems relating to the prostate, so having a male interviewer may have helped patient participants be more comfortable and open in the interviews. GP participants may have been more comfortable in talking to a peer in these interviews; peer discussions are a common part of professional practice for GPs in the form of Balint groups[18] and annual appraisal by a fellow GP[19].

For peer review only

MRI is a new test for prostate cancer, and has only recently been integrated into diagnostic pathways. GPs are not currently able to request an MRI of the prostate, and access to MRI for other indications varies across the NHS. Some GPs were reluctant to engage in any discussion about prostate MRI as they felt it was outside their current scope of practice, and may have been focused on the more traditional (pre-MRI) prostate cancer pathway. In this context, data gathered from GP participants was not as rich as the data collected from the patients.

Relation to published literature

This is the first study the authors are aware of to explore experiences of the modern pre-biopsy MRI prostate cancer diagnostic pathway from the perspective of patients and GPs. Similar studies with patients or GPs have been conducted before MRI emerged as a diagnostic test for prostate cancer. Ruseckaite *et al* interviewed 10 GPs from metropolitan Melbourne and a regional part of Victoria, Australia regarding their perceptions of prostate cancer care. In line with the findings of this study, most men were willing to have a PSA blood test, and some GPs had to grapple with inconsistent guidance from specialist bodies[20]. Evans *et al* assessed men's experience of PSA testing in primary care in Wales, and also found that social networks and media stories influenced patient demand for testing. In contrast to the views of GPs in this study, the men in the study by Evans *et al* felt decision-making about testing was doctor-centred rather than shared or patient-centred[21].

A number of qualitative studies have explored patient and GP experiences of diagnostic pathways for other cancer types, including lung[22,23], haematological[24], and breast or bowel cancer[25]. Lung, breast and bowel cancer patients expressed a desire to progress through the diagnostic pathway as fast as possible, which was valued by London patients in this study attending the 'one-stop' clinic. Consistent with GP's concerns about non-specific presentations of prostate cancer, patients and clinicians in studies of other cancer types also report presenting with 'intermittent, vague' symptoms[25] and facing 'uncertainty' through their experience of the diagnostic pathway[23]. This reflects the challenge of making an early diagnosis of cancer in some cases for a range of cancers.

For peer review only

Implications for clinicians and health service design

Men's experiences of the prostate cancer diagnostic pathway are influenced by the appointment burden they face to receive a diagnosis; the mode of communication used by GPs and specialists to communicate test results; and requirements for travel to attend clinic appointments and tests. Significant challenges remain for GPs owing to the limitations of the current clinical signs and tests they rely on to identify possible prostate cancer cases. Men seemed broadly positive about MRI as a new test for prostate cancer, whereas GPs were equivocal owing to a lack of awareness and access. Cancer alliances and health service designers should consider the implications of how MRI is integrated into prostate cancer diagnostic pathways in terms of patient access to MRI, outpatient appointment burden, and communication of clinical guidelines for prostate cancer diagnostic testing and results to patients.

For peer review only

Funding

SWDM is supported by the Can Test Collaborative, which is funded by CRUK (C8640/A23385). FMW and WH are co-directors of CanTest. HA's research is supported by core funding from the United Kingdom's National Institute of Health Research (NIHR) Imperial Biomedical Research Centre. Ahmed currently receives funding from the Wellcome Trust, Medical Research Council (UK), Cancer Research UK, Prostate Cancer UK, National Institute for Health Research (UK), The Urology Foundation, BMA Foundation, Imperial Health Charity, NIHR Imperial BRC, Sonacare Inc., Trod Medical and Sophiris Biocorp for trials in prostate cancer. DE-E receives funding from the Urology Foundation, the BMA Foundation for Medical Research, Imperial Health Charity and the Royal College of Surgeons of England.

Competing interests

HA was a paid medical consultant for Sophiris Biocorp in the previous 3 years. The remaining authors have no conflicts of interest to declare.

Acknowledgements

The authors would like to thank the participants for sharing their time and experiences for this study. We would also like to thank Pauline Sibley and Victor Mariano (Royal Devon & Exeter NHS Foundation Trust) for their assistance with patient recruitment.

Contributors

SWDM, WH and FMW conceived the study. SWDM developed the research protocol with contributions from FMW, AF, and SA. HA, JM and DE-E were local investigators who supervised recruitment into the study. SWDM undertook all interviews. SWDM, FMW, AF and SA performed the analysis. SWDM drafted the first version of the manuscript. All authors contributed to the development of the manuscript, and approved the final submitted version.

Ethics approval

Ethics committee approval was received from the NHS HRA South-West Frenchay research ethics committee (REC reference 19/SW/0040).

For peer review only

Patient consent for publication

Patient consent for publication of anonymised individual remarks was obtained at the time of recruitment into the study.

Data sharing statement

All data requests should be submitted in writing to the corresponding author

Disclaimer

The views expressed in this publication are those of the author(s) and not necessarily those of the NIHR or the Department of Health and Social Care.

For peer review only

References

1 Forster AS, Rubin G, Emery JD, *et al.* Measuring patient experience of diagnostic care and acceptability of testing. *Diagnosis* 2021;**0**. doi:10.1515/dx-2020-0112

2 McPhail S, Johnson S, Greenberg D, *et al.* Stage at diagnosis and early mortality from cancer in England. *British journal of cancer* 2015;**112**:S108–15. doi:10.1038/bjc.2015.49

3 Sandager M, Jensen H, Lipczak H, *et al.* Cancer patients' experiences with urgent referrals to cancer patient pathways. *European Journal of Cancer Care* 2019;**28**:1–11. doi:10.1111/ecc.12927

4 NICE. Suspected cancer: recognition and referral. 2015;:1–95.https://www.nice.org.uk/guidance/ng12 (accessed 13 Oct 2021).

5 Fraser J, Pearson C, Shelton J, *et al.* Using linked English cancer registration data to assess variation in diagnostic pathway length. *International Journal of Population Data Science* 2019;**4**:121.

6 Clements A, Watson E, Rai T, *et al.* The PSA testing dilemma: GPs' reports of consultations with asymptomatic men: a qualitative study. *BMC Family Practice* 2007;**8**:35. doi:10.1186/1471-2296-8-35

7 Pickles K, Carter SM, Rychetnik L. Doctors' approaches to PSA testing and overdiagnosis in primary healthcare: A qualitative study. *BMJ Open* 2015;**5**. doi:10.1136/bmjopen-2014-006367

8 NICE. Prostate Cancer: Diagnosis and Management. ng131. 2019. doi:10.1097/00005392-200308000-00097

9 Merriel SWD, Walter FM, Hamilton W. Integrating multiparametric MRI into prostate cancer diagnostic pathways within the NHS and internationally – A diagnostic pathways mapping study. In: *Cancer REsearch UK 5th Biennial Early Diagnosis Research Conference*. Birmingham: Cancer Research UK 2019. https://www.cancerresearchuk.org/health-professional/diagnosis/early-diagnosis-initiative/early-diagnosis-research-conferences/2019-conference-programme-and-registration

10 Drost FJ, Osses DF, Nieboer D, *et al.* Prostate MRI, with or without targeted biopsy and standard biopsy for detecting prostate cancer: A Cochrane systematic review and meta-analysis. *Cochrane Database of Systematic Reviews* 2019; CD012663. doi:10.1016/s1569-9056(19)30534-2

11 Scott SE, Walter FM, Webster A, *et al.* The model of pathways to treatment: Conceptualization and integration with existing theory. *British Journal of Health Psychology* 2013;**18**:45–64. doi:10.1111/j.2044-8287.2012.02077.x

12 Walter F, Webster A, Scott S, *et al.* The Andersen Model of Total Patient Delay: A systematic review of its application in cancer diagnosis. *Journal of Health Services Research and Policy* 2012;**17**:110–8. doi:10.1258/jhsrp.2011.010113

13 Silverman D. *Doing qualitative research*. Sage 2013.

14 Guba EG, Lincoln YS. Competing paradigms in qualitative research. In: *Handbook of Qualitative Research*. 1994. 105–17.

15 Denzin NK, Lincoln YS. *The sage handbook of qualitative research*. Sage 2011.

16 Braun V, Clarke V. Using thematic analysis in psychology. *Qualitative Research in Psychology* 2006;**3**:77–101. doi:10.1111/j.1460-2466.1978.tb01621.x

For peer review only

17 Tong A, Sainsbury P, Craig J. Consolidated criterion for reporting qualitative research (COREQ): a 32- item checklist for interviews and focus group. *International Journal of Qualitative in Health Care* 2007;**19**:349–57. doi:10.1093/intqhc/mzm042
18 Roberts M. Balint groups: A tool for personal and professional resilience. *Canadian Family Physician* 2012;**58**:2012.
19 NHS England. Appraisal. 2020. https://www.england.nhs.uk/medical-revalidation/appraisers/ (accessed 1 Apr 2020).
20 Ruseckaite R, Evans S, Millar J, *et al.* GPs' Insights into Prostate Cancer Diagnosis and Care in Regional Victoria, Australia. *The Qualitative Report* 2016;**21**:2365–79
21 Evans R, Edwards AGK, Elwyn G, *et al.* It's a maybe test': men's experiences of prostate specific antigen testing in primary care. *British Journal of General Practice* 2007;**57**:303–10.
22 Christensen HM, Huniche L. Patient perspectives and experience on the diagnostic pathway of lung cancer: A qualitative study. *SAGE Open Medicine* 2020;**8**:1–10. doi:10.1177/2050312120918996
23 Rankin NM, York S, Stone E, *et al.* Pathways to lung cancer diagnosis: A Qualitative study of patients and general practitioners about diagnostic and pretreatment intervals. *Annals of the American Thoracic Society* 2017;**14**:742–53. doi:10.1513/AnnalsATS.201610-817OC
24 Howell D, Hart R, Smith A, *et al.* "Unpacking" pathways to lymphoma and myeloma diagnosis: Do experiences align with the Model of Pathways to Treatment? Findings from a UK qualitative study with patients and relatives. *BMJ Open* 2020;**10**. doi:10.1136/bmjopen-2019-034244
25 Bergin RJ, Emery JD, Bollard R, *et al.* Comparing Pathways to Diagnosis and Treatment for Rural and Urban Patients With Colorectal or Breast Cancer: A Qualitative Study. *Journal of Rural Health* 2020;**00**:1–19. doi:10.1111/jrh.12437

For peer review only

Figure 1 – South West Prostate Cancer Diagnostic Pathway, NHS Cancer Alliances in South-West Peninsula and Somerset, Wiltshire, Avon & Gloucester (SWAG)

mpMRI – multiparametric magnetic resonance imaging; TRUS – transrectal ultrasound guided biopsy; MDT – multidisciplinary team

Figure 2 – RAPID pathway, Imperial College Healthcare NHS Trust, London

2WW – Two Week Wait pathway; mpMRI – multiparametric magnetic resonance imaging; MRI – magnetic resonance imaging

Figure 3 – Thematic diagram from patient participant interviews

Figure 4 – Thematic diagram from GP participant interviews

For peer review only

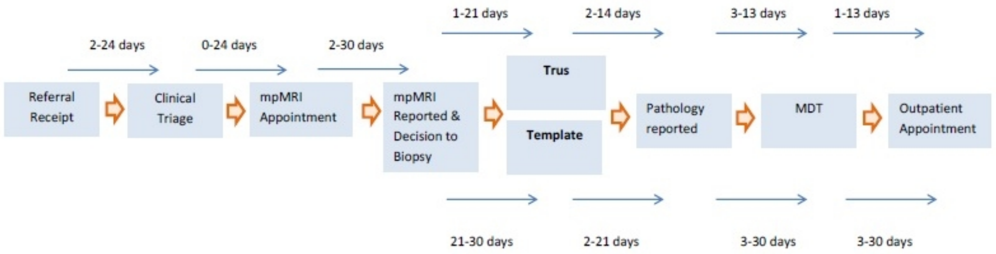

Figure 1 – South West Prostate Cancer Diagnostic Pathway, NHS Cancer Alliances in South-West Peninsula and Somerset, Wiltshire, Avon & Gloucester (SWAG)
mpMRI – multiparametric magnetic resonance imaging; TRUS – transrectal ultrasound guided biopsy; MDT – multidisciplinary team

207x55mm (96 x 96 DPI)

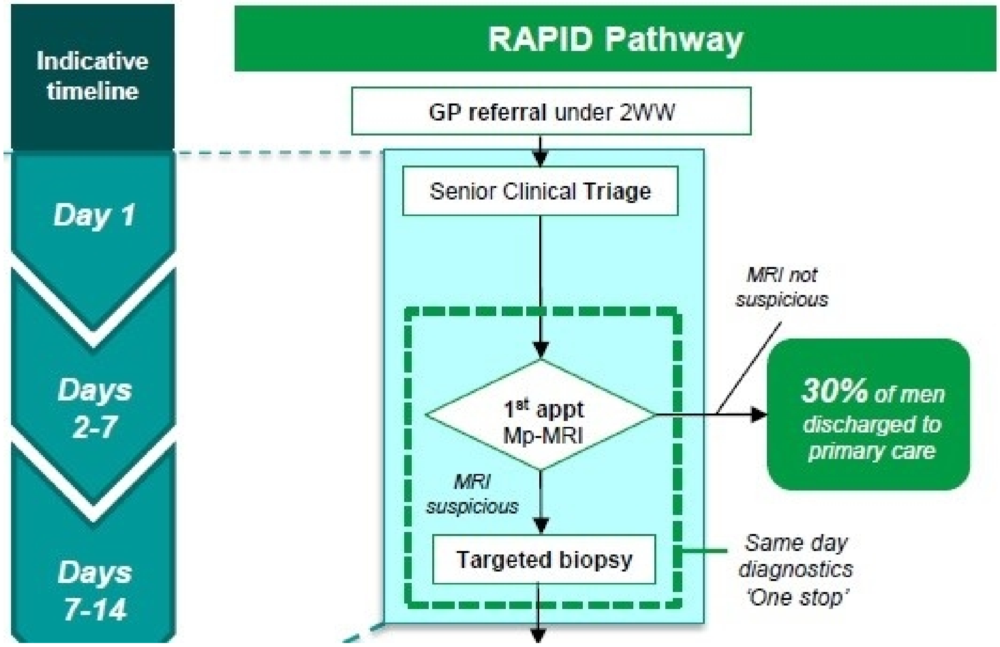

Figure 2 – RAPID pathway, Imperial College Healthcare NHS Trust, London
2WW – Two Week Wait pathway; mpMRI – multiparametric magnetic resonance imaging; MRI – magnetic resonance imaging

140x91mm (96 x 96 DPI)

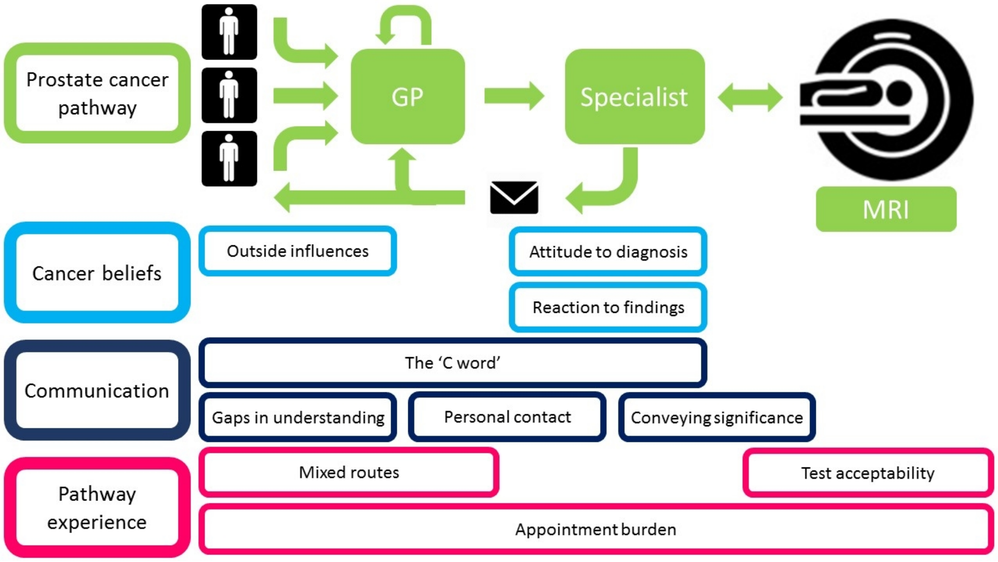

Figure 3 – Thematic diagram from patient participant interviews

338x190mm (96 x 96 DPI)

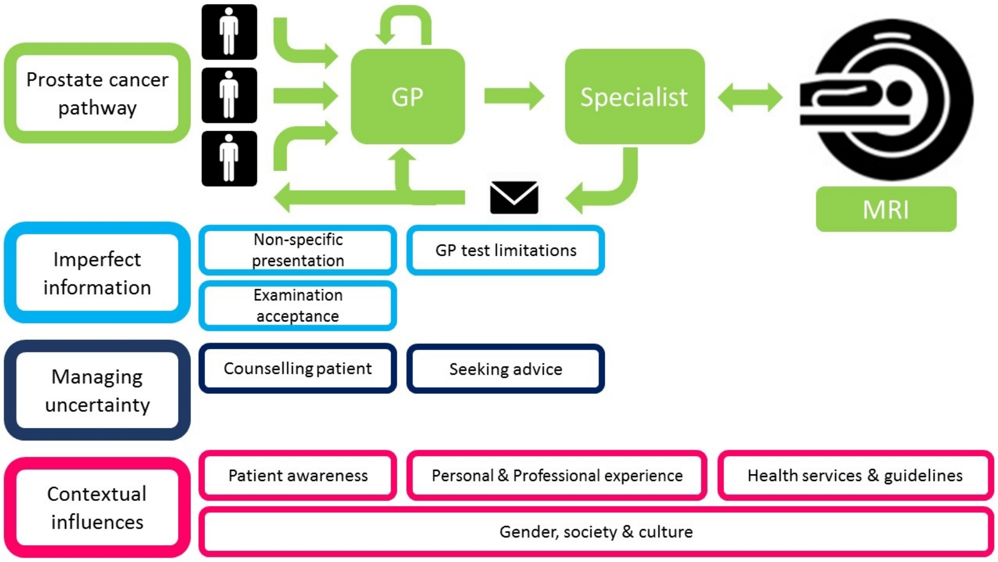

Figure 4 – Thematic diagram from GP participant interviews

338x190mm (96 x 96 DPI)

Patient participant interview guide

Introduction (5mins)
Thank you for agreeing to participate in this interview.

Introduce myself and my role.

This study seeks to understand your knowledge and understanding of diagnostic tests for prostate cancer, and your experiences of the current prostate cancer diagnostic pathway in the region where you live. It is part of my PhD at the University of Exeter. This study has been funded by Cancer Research UK, and has ethical approval from the NHS Health Research Authority and the University of Exeter.

As we've talked about with the consent form, participating in this study is voluntary and you can stop at any time. We want to know about your experiences and what you think, so there are no right or wrong answers.

This interview is being recorded for the purposes of qualitative analysis by the researchers. You can ask for the recording to be stopped at any time. What you say will be kept confidential and anonymous, unless we discuss something that suggests there is a significant risk to yourself or someone else. Everyone being interviewed will be asked the same questions, so if you don't have an answer to any of the questions that's fine, just say so and we can move on.

This interview study if focused on your experience of diagnostic test for prostate cancer. However, if you have a partner, family member or significant other who you wish to be present that's fine. Ideally we would start the interview without them, and then invite them in later on. If you and they are happy for them to participate, they would need to complete a consent form as well.

Ensure participant has copy of participant information sheet

Answer any questions

Ensure consent form is completed correctly

Commence audio recording

For peer review only

For peer review only - http://bmjopen.bmj.com/site/about/guidelines.xhtml

Basic demographics (5 minutes)

"To start with, can you tell me a little bit about yourself and your background"

Check - Age, Ethnicity, City/town lived in

Prostate cancer diagnosis journey (5-10 minutes)

"Now I would like to talk a bit about how you came to have tests for possible prostate cancer."

When did you first notice symptoms (if any)? Which symptoms were they?

How long until consulted you consulted your GP? What affected that decision?

How did GP assess? PSA? DRE?

Decision to refer – what do you remember about that discussion?

mpMRI for prostate cancer (10-15 minutes)

"I would now like to ask some questions about having an MRI scan for possible prostate cancer."

Did you have any concerns or reservations about having an MRI? What were your expectations?

What do you understand about how an MRI works?

What was your experience of having an MRI?

mpMRI result (10 minutes)

"If you are happy to discuss, I would like to ask a few questions about the results of your MRI scan and the next steps."

What was the result of your mpMRI? Did you understand it? How much did you trust the findings?

How were the results communicated? Did you have any questions as a result?

What were you told about the results' meaning?

What else did you discuss with the specialist?

Doctor(s) involved in cancer diagnosis (5 minutes)

"Finally, I would like to ask about your thoughts regarding the way we organise testing for prostate cancer."

What works well? What could be done better?

Who do you feel should be involved in investigating for a possible cancer diagnosis?

If you could design the ideal cancer diagnosis testing service, how would it work?

Interview close (2 minutes)

Thank you for participating in this interview. The data you have provided will be transcribed under a pseudonym, and analysed by the research team. You will be sent a final study report after the analysis has been completed.

If you have any questions or concerns about the study, please contact Ms Pam Baxter at the Research Ethics and Governance Office at the University of Exeter on 01392 723588 or via email p.r.baxter2@exeter.ac.uk Her details are on your patient information leaflet.

For peer review only

For peer review only - http://bmjopen.bmj.com/site/about/guidelines.xhtml

GP participant interview guide

Introduction (5mins)

Thank you for agreeing to participate in this interview.

Introduce myself and my role.

This study seeks to understand your knowledge and understanding of diagnostic tests for prostate cancer, and your experiences of the current prostate cancer diagnostic pathway in the region where you work. It is part of my PhD at the University of Exeter. This study has been funded by Cancer Research UK, and has ethical approval from the NHS Health Research Authority and the University of Exeter.

As we've talked about with the consent form, participating in this study is voluntary and you can stop at any time. We want to know about your experiences and what you think, so there are no right or wrong answers.

This interview is being recorded for the purposes of qualitative analysis by the researchers. You can ask for the recording to be stopped at any time. What you say will be kept confidential and anonymous, unless we discuss something that suggests there is a significant risk to yourself or someone else. This interview is not assessing your clinical competence, and we want to hear about your approach and experiences. Everyone being interviewed will be asked the same questions, so if you don't have an answer to any of the questions that's fine, just say so and we can move on.

This interview study if focused on your experience of diagnostic test for prostate cancer. However, if you have a partner, family member or significant other who you wish to be present that's fine. Ideally we would start the interview without them, and then invite them in later on. If you and they are happy for them to participate, they would need to complete a consent form as well.

Ensure participant has copy of participant information sheet

Answer any questions

Ensure consent form is completed correctly

Commence audio recording

For peer review only

For peer review only - http://bmjopen.bmj.com/site/about/guidelines.xhtml

Basic demographics (5 minutes)
"To start with, can you tell me a little bit about yourself and your background"

Age, Gender, Years of GP experience, Main CCG area you work in

Decision to refer for suspected prostate cancer (10-15 minutes)
"I would like to now move on to your current practice around referring men with suspected prostate cancer for further investigation"

"What symptoms/signs do you enquire about when assessing a man for suspected prostate cancer? How do they affect your decision to refer?"

PSA use – When would you offer it to a man? What are the important points you make about PSA when counselling a man about the test? What do you do with a negative PSA?

"What other factors, if any, affect your decision to refer a man for further investigation?"

"What are the key points you discuss with men when making a referral?"

Diagnostic testing for prostate cancer (15-20 minutes)
"Now I would like to ask some questions about diagnostic tests for prostate cancer."

What is your experience of the prostate cancer diagnosis pathway in your region?
What do you know about current diagnostic tests? How accurate do you believe current diagnostic tests are for prostate cancer?
PROMIS trial – have you heard of it? Are you aware of use of mpMRI for prostate cancer?
Do you feel incorporating mpMRI into the prostate cancer diagnosis pathway would be beneficial for patients? Do you believe it could be cost effective?
What would be the characteristics of an ideal diagnostic test for prostate cancer?

Men diagnosed with prostate cancer (5-10 minutes)
"Finally, I would like to ask about any of your patients who have been diagnosed with prostate cancer"

Are you aware of any of your patients diagnosed with low-grade prostate cancer?
If so, what has been the impact of the diagnosis on patient?
Are you aware of any of your patients being put on active surveillance – what is your experience of interacting with these men after diagnosis?

Interview close (2 minutes)
Thank you for participating in this interview. The data you have provided will be transcribed under a pseudonym, and analysed by the researchers. You will be sent a final study report after the analysis has been completed. If you have any questions or concerns about the study, please contact Ms Pam Baxter at the Research Ethics and Governance Office at the University of Exeter on 01392 723588 or via email p.r.baxter2@exeter.ac.uk

For peer review only

For peer review only - http://bmjopen.bmj.com/site/about/guidelines.xhtml

'Acceptability, understanding and experience of diagnostic tests for prostate cancer: a qualitative study with patients and GPs'

IRAS ID: 259602

Protocol Version Number: 1.0

Protocol date: 21/01/2019

Sponsor: University of Exeter

Sponsor Protocol Reference Number: 1819/03

Chief Investigator: Dr Sam Merriel

Supervisors:

Dr Fiona Walter, University of Cambridge

Dr Alice Forster, University College London

Professor Willie Hamilton, University of Exeter

This protocol has regard for the HRA guidance and order of content

For peer review only

For peer review only - http://bmjopen.bmj.com/site/about/guidelines.xhtml

SIGNATURE PAGE

The undersigned confirm that the following protocol has been agreed and accepted and that the Chief Investigator agrees to conduct the study in compliance with the approved protocol and will adhere to the principles outlined in the Declaration of Helsinki, the Sponsor's SOPs, and other regulatory requirement.

I agree to ensure that the confidential information contained in this document will not be used for any other purpose other than the evaluation or conduct of the investigation without the prior written consent of the Sponsor

I also confirm that I will make the findings of the study publically available through publication or other dissemination tools without any unnecessary delay and that an honest accurate and transparent account of the study will be given; and that any discrepancies from the study as planned in this protocol will be explained.

For and on behalf of the Study Sponsor:

Signature: Date: 30/01/2019

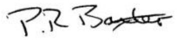

Name (please print): Ms Pam Baxter

Position: Senior Research Governance Officer

University of Exeter

Chief Investigator:

Date: 30/01/2019

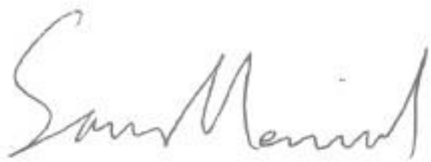

Signature:

Name: (please print): Dr Sam Merriel

For peer review only

For peer review only - http://bmjopen.bmj.com/site/about/guidelines.xhtml

LIST of CONTENTS

GENERAL INFORMATION	Page No.
HRA PROTOCOL COMPLIANCE DECLARATION AND SIGNATURE PAGE	ii
LIST OF CONTENTS	iii
KEY STUDY CONTACTS	iv
STUDY SUMMARY	v
FUNDING	v
ROLE OF SPONSOR AND FUNDER	vi
ROLES & RESPONSIBILITIES OF STUDY STEERING GROUPS AND INDIVIDUALS	vi
**SECTION**	
STUDY FLOW CHARTS	1
1. BACKGROUND	4
2. RATIONALE	4
3. THEORETICAL FRAMEWORK	5
4. RESEARCH QUESTION/AIM(S)	5
5. STUDY DESIGN/METHODS	6
6. SAMPLE AND RECRUITMENT	7
7. ETHICAL AND REGULATORY COMPLIANCE	8
8. DISSEMINATION POLICY	12
9. REFERENCES	12
10. APPENDICES	15

For peer review only

For peer review only - http://bmjopen.bmj.com/site/about/guidelines.xhtml

KEY STUDY CONTACTS

Chief Investigator<br>('The researcher')	Dr Sam Merriel<br>Clinical Senior Research Fellow, Exeter Medical School, University of Exeter<br>1.18, College House, St Luke's Campus, University of Exeter, Heavitree Road, Exeter EX1 2LU<br>s.w.d.merriel@exeter.ac.uk<br>01392 726090
Supervisors	Dr Fiona Walter<br>Principal Researcher in Primary Care Cancer Research, Department of Public Health and Primary Care, University of Cambridge<br>The Primary Care Unit, University of Cambridge School of Clinical Medicine, Box 113 Cambridge Biomedical Campus, Cambridge, CB2 0SR<br>fmw22@medschl.cam.ac.uk<br>01223 762514<br>Dr Alice Forster<br>Senior Research Fellow, Institute of Epidemiology & Health, Faculty of Population Health Sciences, University College London<br>1-19 Torrington place, London, WC1E 6BT<br>alice.forster@ucl.ac.uk<br>Professor Willie Hamilton<br>Professor of Primary Care Diagnostics, Exeter Medical School, University of Exeter<br>1.17, College House, St Luke's Campus, University of Exeter, Heavitree Road, Exeter EX1 2LU<br>w.hamilton@exeter.ac.uk<br>01392 726097
Sponsor	University of Exeter<br>Sponsor's representative<br>Ms Pam Baxter<br>Senior Research Governance Officer<br>Research Ethics and Governance Office,<br>University of Exeter<br>Lafrowda House, St Germans Road, Exeter,<br>Devon, EX4 6TL<br>p.r.baxter2@exeter.ac.uk<br>01392 723588

For peer review only

For peer review only - http://bmjopen.bmj.com/site/about/guidelines.xhtml

Funder(s)	Cancer Research UK

STUDY SUMMARY

Study Title	'Acceptability, understanding and experience of diagnostic tests for prostate cancer: a qualitative study with patients and GPs'
Internal ref. no. (or short title)	Acceptability of mpMRI for prostate cancer diagnosis in primary care
Study Design	Qualitative interview study
Study Participants	Males who have undergone multiparametric Magnetic Resonance Imaging (mpMRI) for suspected prostate cancer.<br>General Practitioners (GPs)
Planned Size of Sample (if applicable)	Purposive sample of approximately 10 GPs and 20 patients
Planned Study Period	01/03/2019 – 01/07/2020
Research Question/Aim(s)	**Aim**<br>To understand, from the perspective of patients and GPs, the acceptability of multiparametric magnetic resonance imaging for men as a diagnostic test for prostate cancer<br>**Objectives**<br>1. Elicit men's experiences of diagnostic tests for suspected prostate cancer<br>2. Explore the knowledge and understanding of diagnostic tests for suspected prostate cancer amongst patients and GPs<br>3. Understand the acceptability of mpMRI as a diagnostic test for prostate cancer from a patient's perspective<br>4. Understand the acceptability of mpMRI as a diagnostic test for prostate cancer from a GP's perspective

FUNDING AND SUPPORT IN KIND

FUNDER(S)	FINANCIAL AND NON FINANCIALSUPPORT GIVEN

For peer review only - http://bmjopen.bmj.com/site/about/guidelines.xhtml

Cancer Research UK	Funding: CanTest (Cancer Research UK Catalyst Award) - Clinical Research Fellow<br>Duration: 3 years 8 months (at 60% FTE)<br>Start date: 02/08/2018<br>Travel costs for researchers and participants to attend face to face interviews<br>Service support costs for recruitment sites<br>Transcription of interviews
National Institute for Health Research Clinical Research Network	Adoption of study onto NIHR portfolio<br>Recruitment of GP participants through CRN practices

ROLES AND RESPONSIBILITIES OF STUDY MANAGEMENT COMMITEES/GROUPS & INDIVIDUALS

The study management group with comprise the researcher, the supervisors, and a PPI representative. The researcher will report to the group, who will oversee the recruitment, data collection and analysis for the duration of the study. The study management group will be independent from the sponsor and the funders.

PROTOCOL CONTRIBUTORS

The study sponsor provided support to the researcher in the preparation of the study protocol, consent forms, patient information leaflets and interview guides. The sponsor will have no role in any other aspect of the conduct, analysis, or dissemination of the study.

The funder had no role in any aspect of the preparation of the study, and will have no role in the conduct, analysis or dissemination of the study.

Patients and members of the public have been involved with writing the lay summary, consent forms, patient information leaflets and interview schedules.

SPONSOR

The study sponsor (the University of Exeter) has ensured that the research team, research protocol and research sites are suitable and that indemnity arrangements are in place. In reviewing the research and ethics documentation, the sponsor has further ensured that appropriate risk management is in place and that the study is managed and conducted in accordance with relevant legislation and codes of good practice. The sponsor will ensure that relevant approvals are in place before the study begins, that the study is conducted in accordance with the protocol and relevant approvals, and that appropriate record-keeping and data management is maintained. The sponsor must approve any study amendments or modifications and will be notified of any significant developments or adverse events in accordance with appropriate guidelines. The sponsor has reviewed plans for data storage and retention and plans for dissemination of the research findings.

KEY WORDS: Prostate cancer; MRI; diagnosis; primary care; acceptability

For peer review only - http://bmjopen.bmj.com/site/about/guidelines.xhtml

For peer review only

For peer review only - http://bmjopen.bmj.com/site/about/guidelines.xhtml

STUDY FLOW CHART 1 – Patient participant recruitment at Imperial College Healthcare

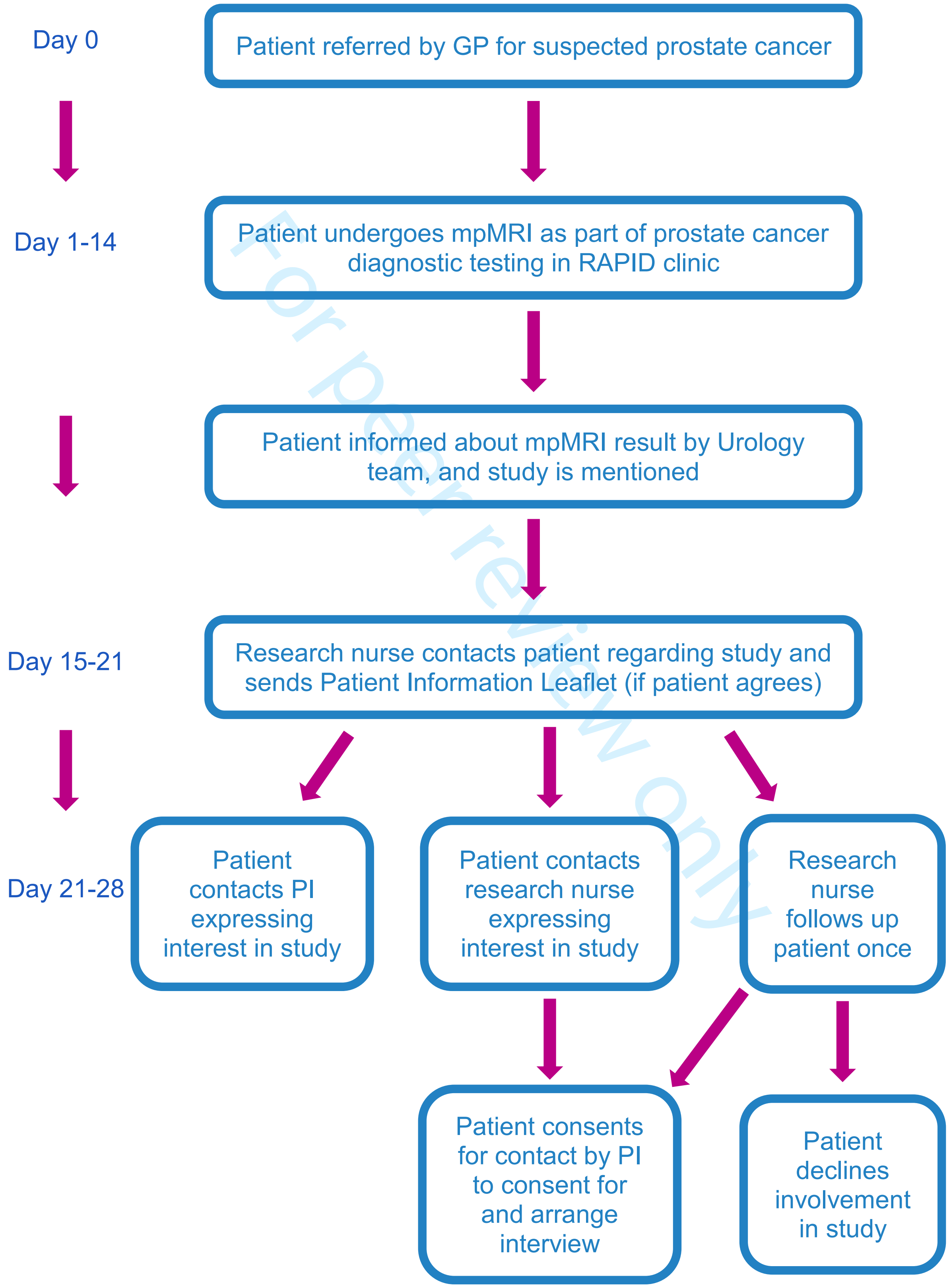

For peer review only

For peer review only - http://bmjopen.bmj.com/site/about/guidelines.xhtml

STUDY FLOW CHART 2 – Patient participant recruitment at Royal Devon & Exeter

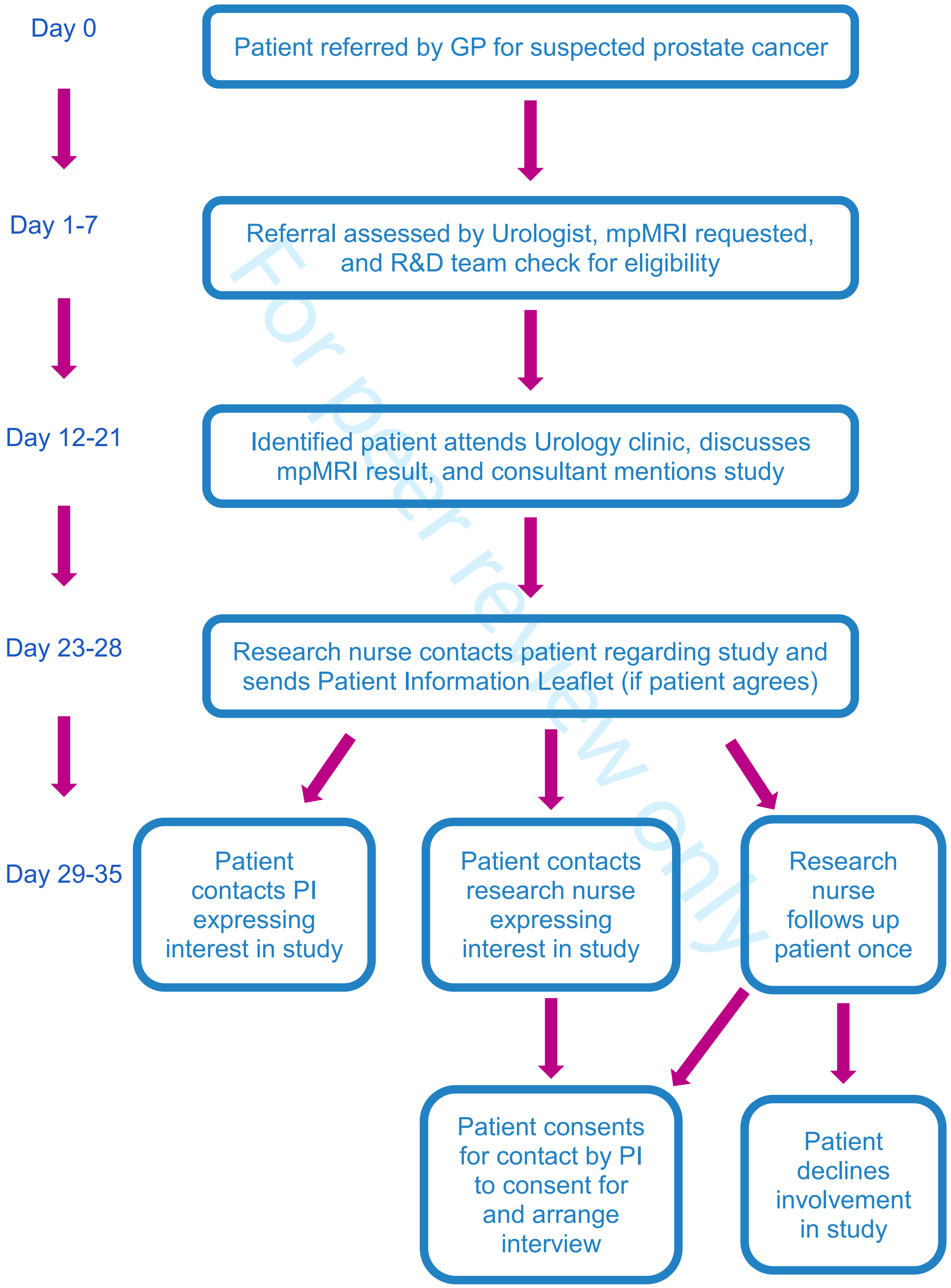

For peer review only - http://bmjopen.bmj.com/site/about/guidelines.xhtml

STUDY FLOW CHART 3 – GP participant recruitment via Clinical Research Networks

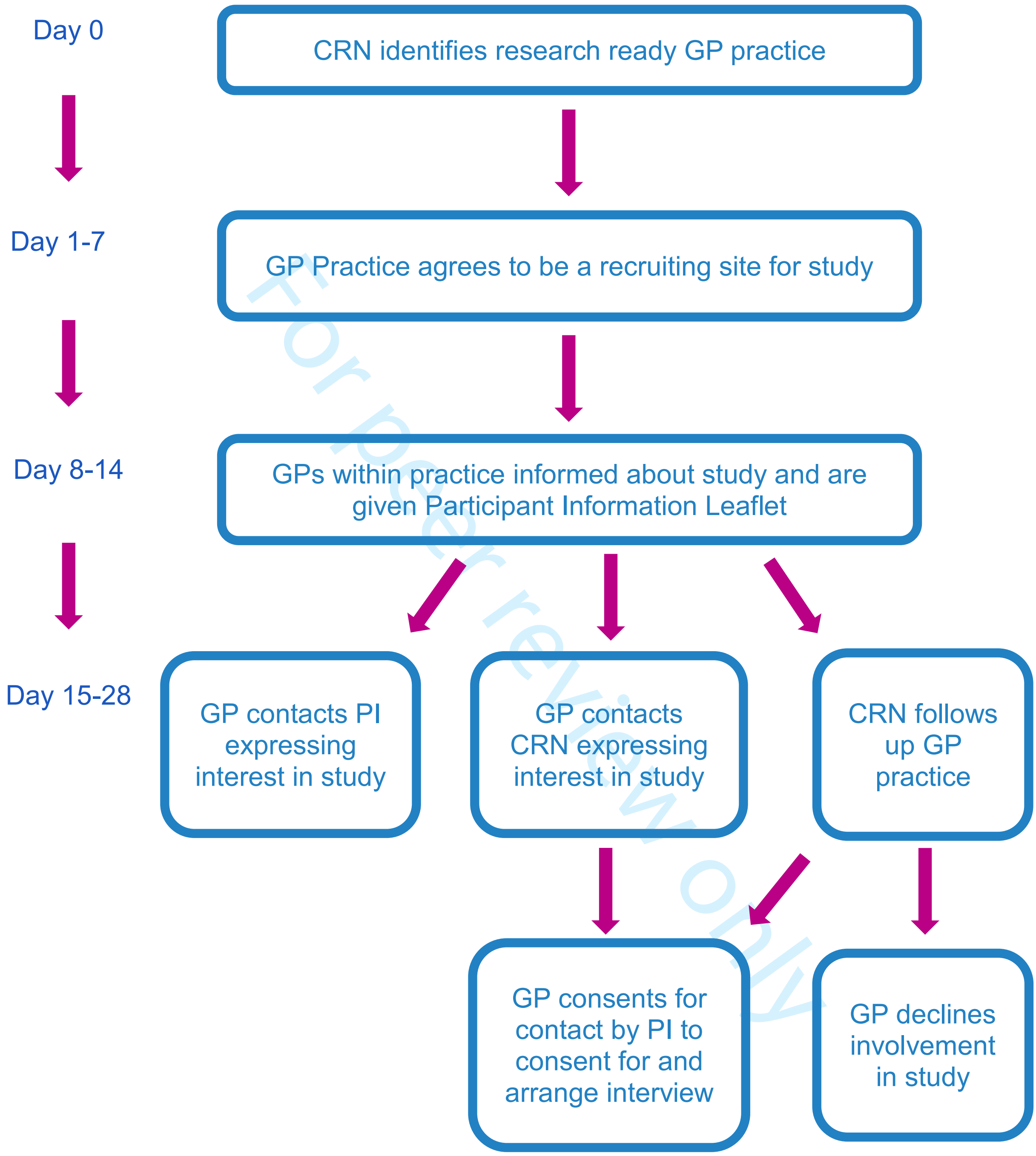

For peer review only

For peer review only - http://bmjopen.bmj.com/site/about/guidelines.xhtml

STUDY PROTOCOL

'Acceptability, understanding and experience of diagnostic tests for prostate cancer: a qualitative study with patients and GPs'

1 BACKGROUND

Implementation of diagnostic tests into routine clinical practice should follow a rigorous process of evaluation from showing analytical validity and diagnostic accuracy, through to acceptability and cost effectiveness. A number of frameworks for assessing and evaluating tests for use in healthcare have been proposed(1–5). They suggest the test should be able to be performed by the operator(s); it should demonstrate more patient benefit than harm; it should be cost effective relative to currently available tests; it should be able to be integrated into the diagnostic pathway; and it should be acceptable to patients and clinicians.

Cancer diagnosis pathways in the NHS in the UK involve primary and secondary care clinicians(6). Some diagnostic tests can be ordered through 'direct access' by a patient's General Practitioner (GP) if they present with symptoms or signs that could indicate an undiagnosed cancer. These include gastroscopy for oesophageal or gastric cancer; colonoscopy for bowel cancer; flexible sigmoidoscopy for rectal cancer; and CT or MRI head for brain tumours(6). Diagnostic tests for prostate cancer currently requires a referral to secondary care.

The current gold standard diagnostic test for prostate cancer is a transrectal ultrasound-guided (TRUS) biopsy of the prostate. TRUS biopsy procedures take 6-12 samples from different regions of the prostate, which are then examined by a histopathologist for signs of prostate cancer(7). TRUS biopsy carries a risk of infection and sepsis, and there is a risk of under- or misdiagnosis as a result of the random nature of sampling the prostate(8). Multiparametric MRI (mpMRI) scanning of the prostate, and reporting using the PiRADS(9) reporting system, has recently been compared to TRUS biopsy in recent large, multicentre trials(10,11) with favourable results in terms of diagnostic accuracy. Few studies have been performed assessing other aspects of the implementation of mpMRI for prostate cancer diagnosis, including patient experience and clinician acceptability.

2 RATIONALE

Prostate cancer is the most common malignancy in males in the United Kingdom(UK)(12). Whilst prostate cancer is responsible for a significant number of cancer-related deaths, the 5- and 10-year survival rates for men with prostate cancer is high(13). This has partly been driven by an increase in the numbers of clinically insignificant prostate cancer cases being diagnosed in the last three decades(14). Better diagnostic tests and diagnostic pathways are needed to reduce rates of over-diagnosis of clinically insignificant prostate cancer, and mpMRI may have a role in this. Following on from the PROMIS trial(10) and others like it(11,15,16), NHS England issued guidance for 'Implementing a timed prostate cancer diagnostic pathway' to NHS Cancer Alliances, reinforcing the benefits for patients of integrating mpMRI into local diagnostic pathways(17).

Studies of patient acceptance of TRUS prostate biopsy for prostate cancer, the current diagnostic test, focus on prevalence of side effects and patient anxiety relating to the test(18–20). Two studies to date have assessed patient acceptance of mpMRI guided biopsy, which also involved questionnaires assessing side effects and attitudes towards the test(21,22). There are no studies that examine acceptability of mpMRI as a diagnostic test for prostate cancer with any theoretical underpinning, and questions remain about men's experience of undergoing the test and receiving the results. There are also very few studies of General Practitioners (GPs), or primary care clinicians, exploring their understanding of diagnostic tests for prostate cancer.

For peer review only - http://bmjopen.bmj.com/site/about/guidelines.xhtml

3 THEORETICAL FRAMEWORK

Acceptability of diagnostic tests has been measured in a number of ways, but no agreed definition for acceptability exists(23). Sekhon et al have proposed a 'Theoretical Framework of Acceptability' relating to healthcare interventions, not just diagnostic tests, which includes seven key constructs (See Figure 1): Affective attitude, Burden, Ethicality, Intervention coherence, Opportunity costs, Perceived effectiveness, Self-efficacy(24). This Framework has been developed to be applicable to both patients and clinicians involved in healthcare interventions, and has a number of key constructs that are particularly relevant to the study aims. Eliciting how a patient feels about undergoing mpMRI ('Affective attitude'), the extent to which they understand the test and its purpose ('Intervention coherence'), and how likely they perceive mpMRI will achieve the purpose of diagnosing prostate cancer ('Perceived effectiveness') will aid understanding in the acceptability of mpMRI as a diagnostic test.

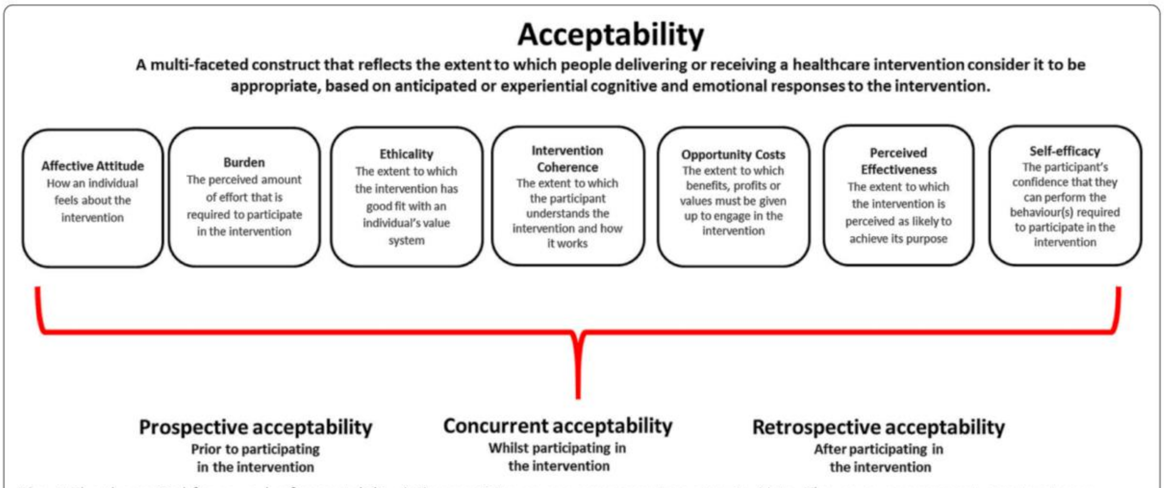

Fig. 3 The theoretical framework of acceptability (v2) comprising seven component constructs. Note: The seven component constructs are presented alphabetically with their anticipated definitions. The extent to which they may cluster or influence each of the temporal assessments of acceptability is an empirical question

Figure 1 – Sekhon's Theoretical Framework of Acceptability(24)

Qualitative research methods lend themselves to answering questions of patient and clinician acceptability with regard to diagnostic tests. Such methods allow researchers to "uncover the nature of a person's experience with a phenomenon", such as cancer and "understand what lies behind any phenomena"(25). Interview studies provide the opportunity to dig deeper and explore how and why patients and clinicians form their beliefs and understanding. It is assumed by the researchers that every man will experience the prostate cancer diagnostic pathway and the diagnostic tests differently, influenced by both internal and external factors. Therefore, a constructivist approach will be taken to capture a range of experiences(26).

4 RESEARCH QUESTION/AIM(S)

Aim - To understand, from the perspective of patients and GPs, the acceptability of multiparametric magnetic resonance imaging for men as a diagnostic test for prostate cancer

4.1 Objectives

1. Elicit men's experiences of diagnostic tests for suspected prostate cancer
2. Explore the knowledge and understanding of diagnostic tests for suspected prostate cancer amongst patients and GPs

For peer review only - http://bmjopen.bmj.com/site/about/guidelines.xhtml

3. Understand the acceptability of mpMRI as a diagnostic test for prostate cancer from a patient's perspective
4. Understand the acceptability of mpMRI as a diagnostic test for prostate cancer from a GP's perspective

4.2 Outcome

This study seeks to understand the acceptability of mpMRI as a diagnostic test for prostate cancer amongst patients and GPs. mpMRI is increasingly being used as part of the assessment of men with suspected prostate cancer by Urologists prior to undertaking a prostate biopsy, however it is unknown how men experience mpMRI scanning. Studies suggest that mpMRI has a negative predictive value of 85-89%(16,27), and that up to 27% of men could avoid a prostate biopsy based on mpMRI findings(11). Within the NHS, some GPs already have the ability to order 'direct access' diagnostic tests for suspected cancers of the oesophagus, stomach, colon, pancreas and brain, and there is some evidence that pre-biopsy mpMRI could also be used as a 'rule out' test in a prostate cancer diagnostic pathway for some patients(28). Before such an approach could be tested and implemented, the experience of patients undergoing mpMRI, and the acceptability of mpMRI as a diagnostic test for patients and GPs needs to be understood.

5 STUDY DESIGN and METHODS of DATA COLLECTION AND DATA ANALYIS

5.1 Study design

This qualitative study will employ semi-structured interviews with men referred from primary care with suspected prostate cancer who have undergone mpMRI, and GPs who have referred men with suspected prostate cancer for further investigation.

5.2 Data collection

Interviews will be conducted by the lead researcher (SM). Interview data will be gathered using an encrypted recording device. The location of the patient interviews will be agreed between the participant and the interviewer prior to the day of the interview. Ideally they will be conducted face-to-face in the patient's home, but other venues and telephone/Skype interviews will be considered. GP interviews will either be held face-to-face at the GP clinic, or via telephone/Skype. The interviewer will utilise a 'buddy system' of informing a colleague if they are travelling to a private residence unaccompanied to conduct an interview with a patient participant.

The interviews will be conducted in a semi-structured manner, allowing participants to share their experiences of diagnostic tests for prostate cancer freely, whilst also meeting the study objectives. Interviews will be supported by topic guides (see 10.1.6 and 10.1.7) for patient and GP participants, which will be used by the interviewer in a flexible way depending on the length and direction of the interview. These topic guides were developed by the researchers based on their experience and knowledge in the field and the study objectives, and will be adapted iteratively as the initial interviews are conducted to enrich data collection.

It is important to treat all participants equally, regardless of their age, culture, education, language ability, or beliefs. Efforts will be made to respect participants' needs, however this study is not sufficiently funded to meet all possible participant needs, such as interpreters for participants with English as their second language.

Following completion of each interview, audio recordings will immediately be downloaded onto an encrypted university laptop computer and the interviewer will make reflective and summary notes. Audio recordings will be transferred to an independent transcribing service securely and transcribed

For peer review only

For peer review only - http://bmjopen.bmj.com/site/about/guidelines.xhtml

verbatim. All participants will be given a pseudonym and any potentially identifying information will be removed.

5.3 Data analysis

A Framework Analysis approach will be taken for this study. Framework analysis is a type of thematic analysis developed by Jane Ritchie and Liz Spencer for applied social policy research(29), and is used in a wide range of areas including health research. Framework Analysis follows seven stages(30):

1. Transcription
2. Familiarisation
3. Coding
4. Developing a working framework
5. Applying framework
6. Charting data into matrix
7. Interpretation

After transcription of the interviews and familiarisation with the data by the investigators, early interview data will be coded using pre-specified codes based on Sekhon's Framework. Refinement and addition of codes and themes will occur with a second researcher (AF) and patient/public representative after coding of initial transcripts using constant-comparison method. The analysis team (SM, AF, and FW) will meet regularly to iteratively develop and agree a coding structure to underpin coding of the remaining transcripts, and a framework will be developed and applied using the agreed codes. SM will perform the final coding of the data. Key themes and narratives within the data will be drawn together from the matrix. Charted data will be imported into NVivo v12 to help manage the data to complete the analysis. Convergence and divergence of views from patients with positive and negative mpMRI scans, and between patients and GPs, will be sought to triangulate key findings.

5.4 Role of the researchers

Three of the researchers (SM, FW, and WH) are trained as GPs, and two are still practicing (SM, FW). All members of the research team will maintain an awareness of their individual biases, beliefs and attitudes that could influence the undertaking of research into men being investigated for prostate cancer. Reflective notes, analysis team meetings, and constant comparison techniques will be used to understand these influences.

6 SAMPLE AND RECRUITMENT

6.1 Eligibility Criteria

This study will recruit participants from two populations;

Patients with suspected prostate cancer who have undergone mpMRI as part of their diagnostic workup.

GPs who have referred at least one male for investigation for suspected prostate cancer within the preceding 12 months.

6.2 Sampling

6.2.1 Size of sample

Approximately 30 participants (10 GPs and 20 patients) will be interviewed for this study, although the final number of participants will depend on when no new themes emerge during interview coding.

For peer review only - http://bmjopen.bmj.com/site/about/guidelines.xhtml

6.2.2 Sampling technique

A purposive sampling approach will be taken for participant recruitment to this study. This will allow recruitment of a sample of men with a range of PiRADS scores (1-2 being low risk of prostate cancer; 3-5 being medium-high risk), ages (<70 years or 70+ years), geographical locations (urban or rural/countryside), and ethnic backgrounds (any white background or BME). In terms of GPs, a purposive sampling approach will allow recruitment of clinicians with a range of ages, genders, practice locations (urban or rural/countryside) and levels of experience.

6.3 Recruitment

6.3.1 Sample identification

6.3.1.1 Patient participants

Patient participants for this study will be recruited from two NHS Trusts; the Imperial College Healthcare NHS Trust in London and the Royal Devon & Exeter NHS Foundation Trust in Exeter. Men referred by their GP for suspected prostate cancer undergo an mpMRI prior to clinical review by a Urologist and potentially a prostate biopsy, depending on the mpMRI report (see study flow charts [pg 1-2]). Research nurses and/or fellows working within the clinic will identify potentially eligible men and contact them within days of undergoing an mpMRI to discuss this study and offer the men a Patient Information Leaflet (PIL – See 11.1.2). The PI and staff at the study sites will regularly communicate about potential recruits to ensure a range of age, ethnicity and geographical backgrounds are present in the included participants. Follow-up contact will be made by the research nurse/fellow if the man does not contact the lead researcher to check whether they wish to participate in the study or not.

Both NHS Trusts involved with this study have reviewed and approved this protocol, and they have expressed confidence that recruitment targets will be met. However, in the event that insufficient men are recruited for participation in this study 12 months after commencing recruitment, a further NHS Trust providing urology services that includes mpMRI for possible prostate cancer will be approached to aid recruitment.

Reasonable travel costs for patient participants to attend any face to face interview will be reimbursed, and participants will be offered a £20 gift voucher in recognition of their participation in the study.

6.3.1.2 GP participants

GP participants will be recruited through two National Institute for Health Research (NIHR) Clinical Research Networks (CRNs); North West London CRN and the South-West Peninsula CRN (see study flow chart [pg 3]). The CRNs will identify local practices from which to recruit eligible GPs to participate in this study, favouring Research Site Initiative (RSI) practices as these practices have an ongoing commitment to research and may have allocated research clinician time. In practices that do not have funded research clinician time, the CRN may provide support for participation in the study. Eligible GPs will be identified by the CRN and the practices, and they will regularly communicate with the PI to determine which GPs to approach for participation. GPs chosen for invitation into the study will be given a PIL (See 11.1.3) to consider participating in the study, and follow-up contact will be made by the CRN to confirm participation.

The NIHR CRN in South-West Peninsula and North West London have reviewed and approved this protocol, and they have expressed confidence in meeting recruitment targets. National adoption of this study within the NIHR CRN portfolio will allow the possibility of recruitment from other CRNs if there is any difficulty recruiting GPs in these two regions.

GP practices will be reimbursed £44.10 per 30 minute interview for the GP’s time to participate.

6.3.2 Consent

For peer review only

For peer review only - http://bmjopen.bmj.com/site/about/guidelines.xhtml

All participants contacted for participation in this study will be given a PIL after being contacted as a potentially eligible participant. After reading the PIL, if the participant is willing to participate they will be contacted by the lead researcher to arrange an interview.

Consent will be taken at the start of the interview. The purpose of the study and the interview will be explained in conjunction with the information presented in the PIL. The lead researcher will assess the patient's ability to consent for participation. The participant will then be presented with a consent form (See 10.1.2 and 10.1.3) to complete if they are still willing to participate in the study.

Conducting patient interviews in their own home may result in other parties being present during the interview, such as the patient's spouse. If another person is present, the patient participant will be asked if they are happy to initially be interviewed in private. If the patient participant wishes for another person to be present, the additional person will be consented for participation and asked to complete a consent form before they join the interview.

6.3.3 Completing recruitment

Recruitment of patients and GPs will continue alongside analysis of interviews conducted to date, until no new themes or ideas emerge from the data.

7 ETHICAL AND REGULATORY CONSIDERATIONS

7.1 Data protection

All data will be collected, stored and processed in accordance with the General Data Protection Regulations 2018 and the Data Protection Act 2018. Informed consent will be obtained from all participants for all aspects of the study. Permission for the collection, storage and use of patient identifiable data (PID) in the study will be provided by consenting participants.

Participants will be free to withdraw consent for participation for any reason and at any time. Where consent is withdrawn, all participant identifiable information held by the research team will be destroyed, and the participant will not receive any further contact regarding the study.

7.2 Data anonymisation

All collected data will be fully anonymised before transfer to professional transcription services. Direct quotations from interview may be used in presenting the study results, however interviewees will not be identifiable in any way in any quotations used.

7.3 Data Storage

Encrypted voice recorders will be used for the interviews. Audio data will be downloaded and kept on secure servers at the University of Exeter until fully anonymised transcripts are created. Any audio files sent to professional transcription services will be anonymously labelled with a unique code, and encrypted for transfer. All physical data such as consent forms and transcripts of interviews will be stored within locked filing cabinets, within a locked office within the University of Exeter Medical School. The keys will be stored separately and only be accessed by the local research team.

All personal data will be securely destroyed within 12 months after the end of the study.

7.4 Assessment and management of risk

This study may be viewed as potentially sensitive in that it explores experiences of personal and intimate symptoms and body systems, in the context of a potentially serious diagnosis (cancer). Although there is a potential ethical problem with interviewing patients around the time of a cancer diagnosis, the proposed recruitment approaches have been successfully used in previous UK early

For peer review only - http://bmjopen.bmj.com/site/about/guidelines.xhtml

diagnosis of cancer studies, led by my supervisor, Dr Fiona Walter. These studies include questionnaire and interview studies people in patients with symptoms suggestive of lung, colorectal and pancreatic cancer (the SYMPTOM study)(31,32), an interview study set among people recently diagnosed with melanoma(33), and the on-going ECASS study (weekly case note reviews and subsequent GP and patient interviews in people with gastro-oesophageal symptoms).

Nevertheless, there are a number of strategies in place to minimise any possible distress that may arise in patients (and informal carers) during the study:

Interviews will be conducted by SM, who has been trained in qualitative interviewing. Combining with his experience as a GP, he would have had experience of communicating sensitively, empathetically and sympathetically with patients when breaking bad news and around sensitive topics, including cancer. Interviews will be conducted at a time and location convenient to participants, and in an unhurried manner, with participants being free to stop the discussion at any time. Appropriate leave-taking will also be practised to ensure that participants are not left in a distressed state following the interview.

It would be reasonable to assume that patients being recruited for interview are aware that they are being investigated with mpMRI due to the suspicion of them having prostate cancer. Local NHS protocols for referring men on the two-week wait urgent cancer referral pathway include the need for GPs to inform the patient they are being referred for a suspected diagnosis of cancer. However, the interviews for this study will not be conducted with that assumption. Early in the interview the patient's understanding for the reason for their referral for mpMRI and other investigations will be explored, to ensure that they patient's underlying knowledge and assumptions are clear to the interviewer.

SM is a practicing GP, and there is the possibility that patients will want to seek advice or an opinion form SM about their healthcare relating to the issue of possible prostate cancer or another health issue. SM will be clear that he is conducting the interview in his capacity as a researcher, and will refer any questions about the patient's healthcare back to their own GP.

A procedure is followed in the event of a participant becoming distressed, which includes the interviewer expressing concern as early as possible about the participant's comfort, offering them tissues or water, and asking whether they would like to take a break or discontinue the interview. Support mechanisms are in place (see Box 1) and the interviewer will inform the participant of these. Participants will also be reminded that they can withdraw from the study or complete the interview at another time, and that this is entirely their decision.

SM will meet regularly during data collection with his supervisors, FW and AF, who have extensive clinical and research experience with patients about cancer symptoms and pathways to diagnosis. This will ensure that should any issues arise they can be dealt with expediently, and learning applied for subsequent interviews.

For peer review only

For peer review only - http://bmjopen.bmj.com/site/about/guidelines.xhtml

Box 1: Guidelines for researchers conducting sensitive interviews

1. All interviews are to be conducted from the outset with the greatest of sensitivity and concern for the respondent's welfare.

2. The interviewer should be observant of the respondent's level of comfort and watch for early signs of distress, such as breaks in speech or nervous body movements. Should early signs appear the interviewer should express concern about the respondent's comfort and ask questions such as (gauged by respondent's signs): would they like a glass of water; if a break is needed; if they would prefer to complete the interview another time; or if they would prefer to discontinue.

3. If overt distress occurs, the interview should cease immediately and actions taken to support the respondent, such as offering tissues or water; seeking immediate additional support from a more familiar person, if available; and staying with the respondent until they are ready to express their wishes on the options available to them.

4. If it becomes apparent that a distressed respondent has particular areas of need concerning their illness or circumstances, where appropriate, the interviewer should offer to assist the respondent to make contact with a relevant support, such as their GP surgery.

5. Concerning the interview, the options eventually offered to a distressed respondent should be (in order): withdraw from the study; or complete the interview another time. The interview should only be continued after a break if the respondent requests this as their unprompted decision.

7.5 Adverse Events

An adverse event will be defined as 'an event that arises directly from participation in the research', including complications that occur in the course of investigation. All adverse events will be discussed with the Supervisors, both of whom (especially FW) have had extensive experience in carrying out similar studies involving early diagnosis of cancer. Appropriate subsequent course of action will be taken after discussion with the full research team, and the Sponsor will be notified.

Appropriate safety procedures will be followed by the researcher(s) when interviewing participants. Should any disclosures requiring action be made, the researchers will have access to the support of the full research team.

7.6 Insurance

Arrangements have been made through the University of Exeter for insurance and/or indemnity to meet the potential legal liability of the sponsor for harm to participants arising from the management, design or conduct of the research.

NHS indemnity scheme will apply for insurance and/or indemnity to meet the potential legal liability of the investigator arising from harm to participants in the conduct of the research at NHS sites.

There are no arrangements in place for payment of compensation in the event of harm to the research participants where no legal liability arises.

7.7 Research Ethics Committee (REC) and other Regulatory review & reports

The researcher will seek NHS research governance and compliance approval, and NHS ethical review, through the HRA approvals process via the Integrated Research Application System (IRAS).

For peer review only - http://bmjopen.bmj.com/site/about/guidelines.xhtml

The researcher will ensure that the protocol and all supporting participant-facing documentation receive HRA approval. Following review, research will only take place once appropriate HRA and Sponsor approvals are in place and confirmation of capacity and capability received from each local NHS site.

A copy of the approved study documents will be submitted to the R&D Office or practice manager at each local site prior to the commencement of any study procedures.

The Chief Investigator is responsible for keeping all correspondence with the REC, producing annual reports, notifying the REC at the end of the study and producing final reports.

7.8 Protocol compliance

Any accidental protocol deviations will be adequately documented on the relevant forms and reported to the Researcher and Sponsor. All deviations from the protocol which are found to frequently recur will require immediate action and could potentially be classified as a serious breach.

Notification of Serious Breaches to GCP and/or the protocol: A 'serious breach' is a breach which is likely to affect to a significant degree;
The safety or physical or mental integrity of the subjects of the study; or
The scientific value of the study

The Sponsor will be notified immediately of any case where the above definition applies during the study.

7.9 Amendments

For any amendment to the study, the Chief Investigator or designee, in agreement with the sponsor will submit information to the appropriate body in order for them to issue approval for the amendment. The Chief Investigator or designee will work with sites (R&D departments at NHS sites as well as the study delivery team) so they can put the necessary arrangements in place to implement the amendment to confirm their support for the study as amended.

If the sponsor wishes to make a substantial amendment to the REC application or the supporting documents, the sponsor must submit a valid notice of amendment to the REC for consideration. The REC will provide a response regarding the amendment within 35 days of receipt of the notice. It is the sponsor's responsibility to decide whether an amendment is substantial or non-substantial for the purposes of submission to the REC.

Amendments will also be notified to the HRA national coordinating function of England where the lead NHS R&D office is based and communicated to the participating organisations (R&D office and local research team) and departments of participating sites to assess whether the amendment affects the NHS permission for that site.

Amendments considered to be non-substantial for the purposes of REC will still be notified to the HRA for approval after confirmation from the Sponsor.

7.10 Peer review

The PhD proposal, which this study forms a key part of, has been subject to peer review by two senior researchers within the CanTest programme. Both reviewers are external to the University of Exeter and are not involved with this study in any way. They are both very experienced and widely published primary care cancer researchers. Feedback from the peer review was utilised to refine and enchance the development of this study.

7.11 Patient & Public Involvement

For peer review only - http://bmjopen.bmj.com/site/about/guidelines.xhtml

The NIHR Clinical Research Facility Peninsula Research Bank steering committee includes a panel of lay members. 14 members of the panel, including two members who had previously been service users investigated for possible prostate cancer, provided input into the acceptability and design of this study. The lay members assisted in the writing of the lay summary, and they reviewed drafts of the consent form, participant information leaflets, and interview guides. Feedback received from these service users and members of the public has been integrated into this protocol and associated documents.

Separately, a PPI group of men (with no history of prostate cancer) is currently being assembled to help steer the PhD that this study forms a part of. This PPI group will be involved with the analysis of results and the dissemination of findings from this study.

7.12 Access to the final study dataset

Access to the full dataset will be limited to the researcher and the supervisors for this study. In line with Cancer Research UK (CRUK) policy, fully anonymised interview transcripts will potentially be made available to researchers for analysis in future related studies, subject to consent obtained from participants.

Any research nurses or fellows involved with recruitment will not have any access to the data collected.

8 DISSEMINIATION POLICY

8.1 Dissemination policy

The data arising from the study will be owned by the University of Exeter.

On completion of the study, data will be analysed and synthesised into a chapter for the PhD of the researcher, registered at the University of Exeter. Access to the full study report, including the protocol, will be made through the Open Research Exeter (ORE) online portal, hosted by the University of Exeter, after the thesis has been accepted by The University.

CRUK are the major funders of this study, through a Catalyst Award (‘CanTest’). CanTest funds the researcher’s salary, training, PhD fees and research costs. CRUK will be acknowledged in any publications associated with this study.

All participants will receive an abridged study report, outlining the major findings of the study.

8.2 Authorship eligibility guidelines and any intended use of professional writers

Authorship for the final study report and any publications associated with this study will be agreed in accordance with the International Committee of Medical Journal Editors guidance.

9 REFERENCES

1. Lijmer JG, Leeflang M, Bossuyt PMM. Proposals for a phased evaluation of medical tests. Med Decis Mak. 2009;29(5):13–21.
2. Fryback DG, Thornbury JR, Fryback DG, Thornbury JR. Medical Decision Making The Efficacy of Diagnostic Imaging. Med Decis Mak. 1991;11:88–94.
3. Horvath AR, Lord SJ, StJohn A, Sandberg S, Cobbaert CM, Lorenz S, et al. From biomarkers to medical tests: The changing landscape of test evaluation. Clin Chim Acta [Internet]. 2014;427:49–57. Available from: http://dx.doi.org/10.1016/j.cca.2013.09.018
4. Lin JS, Thompson M, Goddard KAB, Piper MA, Heneghan C, Whitlock EP. Evaluating genomic

For peer review only - http://bmjopen.bmj.com/site/about/guidelines.xhtml

tests from bench to bedside: A practical framework. BMC Med Inform Decis Mak [Internet]. 2012;12(1):1. Available from: BMC Medical Informatics and Decision Making

5. Thompson M, Weigl B, Fitzpatrick A, Ide N. More Than Just Accuracy: A Novel Method to Incorporate Multiple Test Attributes in Evaluating Diagnostic Tests Including Point of Care Tests. IEEE J Transl Eng Heal Med. 2016;4(January).

6. National Collaborating Centre for Cancer. Suspected cancer [Internet]. NICE. London; 2015. Available from: http://journals.rcni.com/doi/10.7748/ns.29.47.17.s20

7. Mottet N, Bellmunt J, Briers E, Bolla M, Bourke L, Cornford P, et al. EAU - ESTRO - ESUR - SIOG Guidelines on Prostate Cancer [Internet]. European Association of Urology; 2017. Available from: https://uroweb.org/wp-content/uploads/09-Prostate-Cancer_2017_web.pdf

8. Loeb S, Vellekoop A, Ahmed HU, Catto J, Emberton M, Nam R, et al. Systematic review of complications of prostate biopsy. Eur Urol [Internet]. 2013 Dec 1;64(6):876–92. Available from: http://dx.doi.org/10.1016/j.eururo.2013.05.049

9. Hassanzadeh E, Glazer DI, Dunne RM, Fennessy FM, Harisinghani MG, Tempany CM. Prostate imaging reporting and data system version 2 (PI-RADS v2): a pictorial review. Abdom Radiol (New York) [Internet]. 2017;42(1):278–89. Available from: http://www.ncbi.nlm.nih.gov/pubmed/27522352%0Ahttp://www.pubmedcentral.nih.gov/articlerender.fcgi?artid=PMC5247306%0Ahttp://www.ncbi.nlm.nih.gov/pubmed/27522352%5Cnhttp://www.pubmedcentral.nih.gov/articlerender.fcgi?artid=PMC5247306

10. Ahmed HU, Bosaily AE-S, Brown LC, Gabe R, Kaplan R, Parmar MK, et al. Diagnostic accuracy of multi-parametric MRI and TRUS biopsy in prostate cancer (PROMIS): a paired validating confirmatory study. Lancet [Internet]. 2017 Jan 19;380:1–8. Available from: http://dx.doi.org/10.1016/S0140-6736(16)32401-1

11. Kasivisvanathan V, Rannikko AS, Borghi M, Panebianco V, Mynderse LA, Vaarala MH, et al. MRI-Targeted or Standard Biopsy for Prostate-Cancer Diagnosis. N Engl J Med [Internet]. 2018; Available from: http://www.nejm.org/doi/10.1056/NEJMoa1801993

12. Bray F, Ferlay J, Soerjomataram I, Siegel R, Torre L, Jemal A. Global cancer statistics 2018: GLOBOCAN estimates of incidence and mortality worldwide for 36 cancers in 185 countries. CA A J Clin. 2018;00(00):1–31.

13. Office for National Statistics. Cancer registration statistics, England: first release, 2015. Statistical Bulletin. 2017.

14. Sandhu GS, Andriole GL. Overdiagnosis of prostate cancer. J Natl Cancer Inst - Monogr [Internet]. 2012 Dec 27;2012(45):146–51. Available from: http://jncimono.oxfordjournals.org/cgi/doi/10.1093/jncimonographs/lgs031

15. Boesen L, Norgaard N, Logager V, Balslev I, Thestrup K, Bisbjerg R, et al. Assessment of the Diagnostic Accuracy of Biparametric Magnetic Resonance Imaging for Prostate Cancer in Biopsy-Naive Men: The Biparametric MRI for Detection of Prostate Cancer (BIDOC) study. JAMA Netw Open. 2018;1(2).

16. Otti VC, Miller C, Powell RJ, Thomas RM, McGrath JS. The diagnostic accuracy of multiparametric MRI prior to biopsy in the detection of prostate cancer. BJU Int [Internet]. 2018; Available from: http://www.ncbi.nlm.nih.gov/pubmed/29804315%0Ahttp://doi.wiley.com/10.1111/bju.14420

17. NHS England. Implementing a timed prostate cancer diagnostic pathway. NHS; 2018.

18. Peters JL, Thompson AC, Mcnicholas TA, Hines JEW, Hanbury DC, Boustead GB. Increased patient satisfaction from transrectal ultrasonography and biopsy under sedation. BJU Int. 2001;87(9):827–30.

19. Makinen T, Auvinen A, Hakama M, Stenman UH, Tammela TLJ. Acceptability and

For peer review only

For peer review only - http://bmjopen.bmj.com/site/about/guidelines.xhtml

complications of prostate biopsy in population-based PSA screening versus routine clinical practice: A prospective, controlled study. Urology. 2002;60(5):846–50.

20. Clements R, Aideyan OU, Griffiths GJ, Peeling WB. Side effects and patient acceptability of transrectal biopsy of the prostate. Clin Radiol. 1993;47(2):125–6.

21. Egbers N, Schwenke C, Maxeiner A, Teichgräber U, Franie T. MRI-guided core needle biopsy of the prostate: Acceptance and side effects. Diagnostic Interv Radiol. 2015;21(3):215–21.

22. Ullrich T, Schimmöller L, Oymanns M, Blondin D, Dietzel F, Kirchner J, et al. Current Utilization and Acceptance of Multiparametric MRI in the Diagnosis of Prostate Cancer. A Regional Survey. RöFo - Fortschritte auf dem Gebiet der Röntgenstrahlen und der Bildgeb Verfahren [Internet]. 2017;419–26. Available from: http://www.thieme-connect.de/DOI/DOI?10.1055/s-0043-118128

23. Sekhon M, Cartwright M, Francis JJ. Acceptability of health care interventions: A theoretical framework and proposed research agenda. Br J Health Psychol. 2018;1–13.

24. Sekhon M, Cartwright M, Francis JJ. Acceptability of healthcare interventions: An overview of reviews and development of a theoretical framework. BMC Health Serv Res [Internet]. 2017;17(1):1–13. Available from: http://dx.doi.org/10.1186/s12913-017-2031-8

25. Strauss A, Corbin J. Basics of qualitative research. Sage; 1990.

26. Silverman D. Doing qualitative research. Sage; 2013.

27. Ahmed HU, El-Shater Bosaily A, Brown LC, Gabe R, Kaplan R, Parmar MK, et al. Diagnostic accuracy of multi-parametric MRI and TRUS biopsy in prostate cancer (PROMIS): a paired validating confirmatory study. Lancet [Internet]. 2017;389(10071):815–22. Available from: http://dx.doi.org/10.1016/S0140-6736(16)32401-1

28. Panebianco V, Barchetti G, Simone G, Del Monte M, Ciardi A, Grompone MD, et al. Negative Multiparametric Magnetic Resonance Imaging for Prostate Cancer: What's Next? Eur Urol [Internet]. 2018;74(1):48–54. Available from: https://doi.org/10.1016/j.eururo.2018.03.007

29. Ritchie J, Spencer EA. Qualitative data analysis for applied policy research. In: Analyzing Qualitative Data. 1st ed. London: Routledge; 1994. p. 173–94.

30. Gale NK, Heath G, Cameron E, Rashid S, Redwood S. Using the framework method for the analysis of qualitative data in multi-disciplinary health research. BMC Med Res Methodol [Internet]. 2013;13(1):1. Available from: BMC Medical Research Methodology

31. Birt L, Hall N, Emery J, Banks J, Mills K, Johnson M, et al. Responding to symptoms suggestive of lung cancer: A qualitative interview study. BMJ Open Respir Res. 2014;1(1).

32. Hall N, Birt L, Banks J, Emery J, Mills K, Johnson M, et al. Symptom appraisal and healthcare-seeking for symptoms suggestive of colorectal cancer: A qualitative study. BMJ Open. 2015;5(10).

33. Walter FM, Birt L, Cavers D, Scott S, Emery J, Burrows N, et al. "This isn't what mine looked like": A qualitative study of symptom appraisal and help seeking in people recently diagnosed with melanoma. BMJ Open. 2014;4(7):1–12.

For peer review only

For peer review only - http://bmjopen.bmj.com/site/about/guidelines.xhtml

10. APPENDICIES

10.1 Amendment History

Amendment No.	Protocol version no.	Date issued	Author(s) of changes	Details of changes made

For peer review only

For peer review only - http://bmjopen.bmj.com/site/about/guidelines.xhtml

Consolidated criteria for reporting qualitative studies (COREQ): 32-item checklist

Developed from:
Tong A, Sainsbury P, Craig J. Consolidated criteria for reporting qualitative research (COREQ): a 32-item checklist for interviews and focus groups. *International Journal for Quality in Health Care*. 2007. Volume 19, Number 6: pp. 349 – 357

No. Item	Guide questions/description	Reported on Page #
**Domain 1: Research team and reflexivity**		
Personal Characteristics		
1. Inter viewer/facilitator	Which author/s conducted the interview or focus group?	8
2. Credentials	What were the researcher's credentials? E.g. PhD, MD	1-2
3. Occupation	What was their occupation at the time of the study?	8
4. Gender	Was the researcher male or female?	8
5. Experience and training	What experience or training did the researcher have?	1
Relationship with participants		
6. Relationship established	Was a relationship established prior to study commencement?	N/A
7. Participant knowledge of the interviewer	What did the participants know about the researcher? e.g. personal goals, reasons for doing the research	19
8. Interviewer characteristics	What characteristics were reported about the inter viewer/facilitator? e.g. Bias, assumptions, reasons and interests in the research topic	19
**Domain 2: study design**		
Theoretical framework		
9. Methodological orientation and Theory	What methodological orientation was stated to underpin the study? e.g. grounded theory, discourse analysis, ethnography, phenomenology, content analysis	7
Participant selection		
10. Sampling	How were participants selected? e.g. purposive, convenience, consecutive, snowball	7
11. Method of approach	How were participants approached? e.g. face-to-face, telephone, mail, email	7-8
12. Sample size	How many participants were in the study?	11
13. Non-participation	How many people refused to participate or dropped out? Reasons?	11

For peer review only

Setting		
14. Setting of data collection	Where was the data collected? e.g. home, clinic, workplace	8
15. Presence of non-participants	Was anyone else present besides the participants and researchers?	11
16. Description of sample	What are the important characteristics of the sample? e.g. demographic data, date	11
Data collection		
17. Interview guide	Were questions, prompts, guides provided by the authors? Was it pilot tested?	8
18. Repeat interviews	Were repeat inter views carried out? If yes, how many?	N/A
19. Audio/visual recording	Did the research use audio or visual recording to collect the data?	9
20. Field notes	Were field notes made during and/or after the inter view or focus group?	9
21. Duration	What was the duration of the inter views or focus group?	9
22. Data saturation	Was data saturation discussed?	9
23. Transcripts returned	Were transcripts returned to participants for comment and/or correction?	9
**Domain 3: analysis and findings**		
Data analysis		
24. Number of data coders	How many data coders coded the data?	9
25. Description of the coding tree	Did authors provide a description of the coding tree?	9
26. Derivation of themes	Were themes identified in advance or derived from the data?	9
27. Software	What software, if applicable, was used to manage the data?	NVivo
28. Participant checking	Did participants provide feedback on the findings?	19
Reporting		
29. Quotations presented	Were participant quotations presented to illustrate the themes/findings? Was each quotation identified? e.g. participant number	12-18
30. Data and findings consistent	Was there consistency between the data presented and the findings?	12-18
31. Clarity of major themes	Were major themes clearly presented in the findings?	12-18
32. Clarity of minor themes	Is there a description of diverse cases or discussion of minor themes?	12-18

For peer review only

BMJ Open

Experiences of 'traditional' and 'one-stop' MRI-based prostate cancer diagnostic pathways in England: a qualitative study with patients and GPs

Journal:	*BMJ Open*
Manuscript ID	bmjopen-2021-054045.R2
Article Type:	Original research
Date Submitted by the Author:	02-Mar-2022
Complete List of Authors:	Merriel, Samuel; University of Exeter, Institute of Health Research<br>Archer, Stephanie; University of Cambridge School of Clinical Medicine, Public Health and Primary Care; University of Cambridge<br>Forster, Alice; UCL, Behavioural Science and Health<br>Eldred-Evans, David; Imperial College London, Imperial Prostate<br>McGrath, John; Royal Devon and Exeter NHS Foundation Trust, Urology<br>Ahmed, Hashim; Imperial College London, Imperial Prostate<br>Hamilton, Willie; University of Exeter, Peninsula College of Medicine and Dentistry<br>Walter, Fiona; Queen Mary University of London, Wolfson Institute of Public Health; University of Cambridge, Department of Public Health and Primary Care
**Primary Subject Heading**:	Qualitative research
Secondary Subject Heading:	Urology, General practice / Family practice
Keywords:	PRIMARY CARE, QUALITATIVE RESEARCH, Prostate disease < UROLOGY, Urological tumours < ONCOLOGY, Magnetic resonance imaging < RADIOLOGY & IMAGING

SCHOLARONE™
Manuscripts

I, the Submitting Author has the right to grant and does grant on behalf of all authors of the Work (as defined in the below author licence), an exclusive licence and/or a non-exclusive licence for contributions from authors who are: i) UK Crown employees; ii) where BMJ has agreed a CC-BY licence shall apply, and/or iii) in accordance with the terms applicable for US Federal Government officers or employees acting as part of their official duties; on a worldwide, perpetual, irrevocable, royalty-free basis to BMJ Publishing Group Ltd ("BMJ") its licensees and where the relevant Journal is co-owned by BMJ to the co-owners of the Journal, to publish the Work in this journal and any other BMJ products and to exploit all rights, as set out in our licence.

The Submitting Author accepts and understands that any supply made under these terms is made by BMJ to the Submitting Author unless you are acting as an employee on behalf of your employer or a postgraduate student of an affiliated institution which is paying any applicable article publishing charge ("APC") for Open Access articles. Where the Submitting Author wishes to make the Work available on an Open Access basis (and intends to pay the relevant APC), the terms of reuse of such Open Access shall be governed by a Creative Commons licence – details of these licences and which Creative Commons licence will apply to this Work are set out in our licence referred to above.

Other than as permitted in any relevant BMJ Author's Self Archiving Policies, I confirm this Work has not been accepted for publication elsewhere, is not being considered for publication elsewhere and does not duplicate material already published. I confirm all authors consent to publication of this Work and authorise the granting of this licence.

For peer review only

Experiences of 'traditional' and 'one-stop' MRI-based prostate cancer diagnostic pathways in England: a qualitative study with patients and GPs

Dr Samuel W D Merriel MSc, University of Exeter Medical School, University of Exeter, UK

s.w.d.merriel@exeter.ac.uk

Dr Stephanie Archer PhD, Department of Public Health and Primary Care / Department of Psychology, University of Cambridge, UK

saa71@medschl.cam.ac.uk

Dr Alice Forster PhD, Institute of Epidemiology & Health, University College London, UK

alice.forster@ucl.ac.uk

Dr David Eldred-Evans PhD, Imperial Prostate, Department of Surgery & Cancer, Imperial College London, UK; Imperial Urology, Charing Cross Hospital, Imperial College Healthcare NHS Trust, London, UK

d.eldred-evans@imperial.ac.uk

Mr John McGrath FRCS, Department of Urological Surgery, Royal Devon & Exeter NHS Foundation Trust, UK

For peer review only

john.mcgrath4@nhs.net

Prof Hashim U. Ahmed FRCS, Imperial Prostate, Department of Surgery & Cancer, Imperial College London, UK; Imperial Urology, Charing Cross Hospital, Imperial College Healthcare NHS Trust, London, UK

Hashim.ahmed@imperial.ac.uk

Prof Willie Hamilton MD, University of Exeter Medical School, University of Exeter, UK

w.hamilton@exeter.ac.uk

Dr Fiona M Walter MD, Wolfson Institute of Population Health, Queen Mary University of London, UK, & The Primary Care Unit, Department of Public Health and Primary Care, University of Cambridge, UK fiona.walter@qmul.ac.uk

Corresponding author

Dr Samuel W D Merriel

1.18 College House, St Luke's campus, University of Exeter

Heavitree Road, Exeter, UK EX1 2LU

+441392 726002

s.w.d.merriel@exeter.ac.uk

Keywords: Prostate cancer, diagnostic pathway, primary care

For peer review only

Abstract

Objectives

This study aimed to understand and explore patient and GP experiences of 'traditional' and 'one-stop' prostate cancer diagnostic pathways in England.

Design

Qualitative study using semi-structured interviews, analysed using inductive thematic analysis

Setting

Patients were recruited from National Health Service (NHS) Trusts in London and in Devon; GPs were recruited via National Institute for Health Research (NIHR) Clinical Research Networks. Interviews were conducted in person or via telephone.

Participants

Patients who had undergone a magnetic resonance imaging (MRI) scan of the prostate as part of their diagnostic work-up for possible prostate cancer, and GPs who had referred at least one patient for possible prostate cancer in the preceding 12 months.

Results

22 patients (aged 47 – 80 years) and 10 GPs (6 female, aged 38 – 58 years) were interviewed. Patients described three key themes: cancer beliefs in relation to patient's attitudes towards prostate cancer; communication with their GP and specialist having a significant impact on experience of the pathway; and pathway experience being influenced by appointment and test burden. GP interview themes included: the challenges of dealing with imperfect information in the current pathway; managing uncertainty in identifying patients with possible prostate cancer, and sharing this uncertainty with them, and other social, cultural and personal contextual influences.

Conclusions

Patients and GPs reported a range of experiences and views of the current prostate cancer diagnostic pathways in England. Patients valued 'one-stop' pathways integrating prostate MRI and diagnostic consultations with specialists over the more traditional approach of several hospital appointments. GPs remain uncertain how best to identify patients needing referral for urgent prostate cancer testing due to the lack of accurate triage and risk assessment strategies.

For peer review only

Article summary

Strengths and limitations

- Patient experiences of two very different prostate cancer diagnostic pathways compared and contrasted
- Patient sample feature a broad range of ages, geographical regions, and cancer investigation journeys to generate rich data
- First study to explore GP experience and understanding of new prostate cancer diagnostic pathways incorporating magnetic resonance imaging (MRI)
- Limited knowledge of prostate MRI curtailed interviews with some GP participants

For peer review only

Introduction

Patient experience of healthcare has developed as an important marker of quality of care in recent decades. However, measuring and understanding patient experience of diagnostic pathways and services is underexplored and poorly prioritised compared to other aspects of healthcare such as access or treatments[1]. Assessment of the impact of variations in pathway design between health services may also identify elements associated with better patient experience, such as quicker access to testing, that could be implemented more widely and elements associated with adverse experience, such as high appointment burden, that can be avoided.

The National Health Service (NHS) in England has a Two Week Wait (2WW) urgent cancer referral pathway system, where any patient with symptoms or signs of a potential undiagnosed cancer referred by their General Practitioner (GP) should have a specialist review for further investigation within two weeks[2]. Cancer diagnostic pathways are prioritised for urgent access to specialist assessment and diagnostic tests as early stage diagnosis is associated with increased survival[3]. Not only do shorter diagnostic intervals improve outcomes for patients, but patients also report better experiences of care[4]. Significant variation in cancer diagnostic pathways between NHS Trusts and regions in England exists, most markedly for prostate cancer[5]. Identifying patients for 2WW prostate cancer referral in primary care is also challenging for GPs owing to limitations of existing tests, including prostate specific antigen (PSA), which can impact on doctor-patient communication and patient experience of the early stages of the prostate cancer diagnostic pathway[6,7].

National Institute for Health and Care Excellence (NICE) guidance for diagnosing prostate cancer in England was updated in 2019 to recommend pre-biopsy magnetic resonance imaging (MRI) for men suspected of having prostate cancer[8]. In response, Cancer Alliances and Hospital Trusts in the NHS have updated local prostate cancer diagnostic pathways, with significant variation in the implementation of MRI[9]. Despite the potential benefits prostate MRI brings in terms of more accurate prostate cancer diagnosis[10], adding further testing into the prostate cancer diagnostic pathway could lengthen the diagnostic interval, adversely impacting patient experience.

For peer review only

Experiences of the prostate cancer diagnostic pathway for patients and GPs since the advent of prostate MRI is unknown. The aim of this study was to elicit the experience of patients and GPs following two prostate cancer diagnostic pathways that incorporate pre-biopsy MRI in different ways to inform optimal prostate cancer diagnostic pathway design. In the context of the Model of Pathways to Treatment, a key theoretical framework in cancer diagnostic pathways, this study focuses on the 'Help-seeking' and 'Diagnostic' intervals and explores the perspectives of both patient and clinician[11,12].

For peer review only

Methods

This qualitative study used semi-structured interviews to explore the experiences of patients referred from primary care with possible prostate cancer who had undergone an MRI, and GPs who have referred men with possible prostate cancer for further investigation. A constructivist approach was adopted to access the data and understand the experiences of patients and GPs[13] based on their individual experiences (past and present) and the socio-cultural context[14,15].

Participants

This study recruited participants from two populations through purposive sampling;

- Patients with possible prostate cancer who had undergone an MRI as part of their diagnostic workup.
- GPs who had referred at least one patient for investigation for possible prostate cancer within the preceding 12 months.

Patients who were undergoing MRI for active surveillance or watchful waiting for a previously diagnosed prostate cancer were not eligible, as the focus of this study was on the role of MRI in the diagnosis of prostate cancer rather than management.

Recruitment

Patients were recruited from two NHS Trusts in England: the Royal Devon & Exeter NHS Foundation Trust in Exeter and the Imperial College Healthcare NHS Trust in London. The Royal Devon & Exeter Hospital use separate outpatient appointments in the South West (SW) Prostate Cancer Diagnostic Pathway for a prostate MRI, consultant review, and prostate biopsy (if required), as shown in Figure 1. Imperial College employ the RAPID pathway, where patients undergo a prostate MRI scan, receive their MRI result, and potentially undergo a prostate biopsy on the same day at a single outpatient attendance (see Figure 2). These Trusts were selected as prostate MRI has been implemented in very different ways, creating the opportunity to explore and compare patient and clinician experiences in different clinical contexts. Research staff at the Trusts identified potentially eligible men and contacted them within days of undergoing an MRI to discuss this study and offer the men a Patient Information Leaflet (PIL). The lead investigator and local recruitment leads were in regular contact throughout recruitment to identify any under-

For peer review only

represented groups of men and focus recruitment where needed. Travel costs for patient participants to attend a face to face interview were reimbursed, and participants were also offered a gift voucher in recognition of contributing their time.

Figure 1

Figure 2

GPs were recruited through two National Institute for Health Research (NIHR) Clinical Research Networks (CRNs) in the same regions as the hospital sites: North West London CRN and the South-West Peninsula CRN. The CRNs promoted the study to local practices, and GPs expressed their interest to the CRNs. Eligibility and basic demographics were checked to assist with purposive sampling. GPs chosen for invitation into the study were given a PIL to review prior to the arrangement of an interview. GP practices were reimbursed for the GP's time to participate in the study.

A purposive sampling approach was used, in order to obtain a diverse group of participants with a wide range of geographical locations, ages, ethnicities, genders (GPs) and MRI results (patients).

Data collection

One-to-one interviews were conducted with all participants in this study between July and November 2019 by SM (a male GP with training in qualitative interviewing). Patient participants were either interviewed face-to-face in their own home or via telephone, while all GP participant interviews were conducted via telephone. The interviewer and participant were not known to each other before the study. Formal written consent was obtained from all participants, and patient's partners if present (n=2), prior to commencement of the interview. A semi-structured approach was followed, with separate interview topic guides for patient and GP interviews to support discussions (See supplementary file 1 and 2). The topic guide was developed iteratively with input from our patient/public partners; it was further refined during the first three interviews

For peer review only

to incorporate all aspects of the revised prostate cancer diagnostic pathway, and was used flexibly to ensure that no key aspects of the diagnostic pathway experience were missed. An encrypted audio recording device was employed to record all interviews, and written notes were taken during and immediately following the interviews. Interview times ranged between 15-45 minutes each, and no repeat or follow-up interviews were undertaken. Interview recordings were transferred securely to an independent transcribing service, and transcribed verbatim.

Data analysis

An inductive thematic analysis was conducted to understand the experiences of participants[16], using the conceptual framework of the Model of Pathways to Treatment[11,12]. The researchers initially immersed themselves in the data through reading and re-reading individual transcripts and listening back to the audio recordings of the interviews. A selection of early interviews were coded, and this initial coding framework was reviewed and refined by SM, SA and FW. The remaining interview transcripts were coded by SM inductively from the entirety of the data. The codes were reviewed and arranged into themes through an iterative process, returning to the original data as needed. Patient and GP transcripts were analysed separately. Within and between themes, the experiences of participants following different diagnostic pathways were compared and contrasted. Recruitment ceased when no new themes emerged in analysis. Transcripts were imported into NVivo v12 to manage the data for the analysis. A study summary report was sent to all study participants after completion of data analysis.

Patient & Public (PPI) Involvement statement

Eight men were recruited via the People in Health West of England (PHWE) initiative to contribute to the research: these men had a range of ages, locations, ethnic backgrounds and experiences with prostate cancer. PPI group members reviewed the plain English summary and all patient participant documents, and gave feedback prior to submission as part of the ethical approval application. PPI group members also gave input into the interview topic guides and the expected burden of involvement for participants. One of the anonymised patient interview transcripts was shared with the group at a meeting, and discussed to explore themes emerging from the text.

For peer review only

Ethical approval

Ethics committee approval was received from the NHS HRA South-West Frenchay research ethics committee (REC reference 19/SW/0040).

COREQ reporting guidelines

This manuscript has been written in accordance with the consolidated criterion for reporting qualitative research (COREQ) checklist[17]. Further detail regarding the methods can be found in the study protocol (see Supplementary file 3).

For peer review only

Results

Participants

Twenty-two patients were interviewed between July and November 2019; two with their wives present and involved in the interview: participant ages ranged from 47 – 80 years. Ten GPs were interviewed: most were female (n = 6), with an age range of 38 – 58 years (See table 1). Five further potential (three patients and two GPs) participants were approached but declined to participate without giving a reason.

	Patients (n = 22)		GPs (n = 10)
**Age**		**Age**	
<65	8	31-40	3
65+	14	41-50	6
		50+	1
**Location**		**Location**	
London	10	London	4
Devon	12	Devon	6
**Ethnicity**		**Gender**	
White	19	Male	4
BME	3	Female	6
**PIRADS v2***		**Role**	
1-2	6	Partner	8
3-5	15	Salaried	2
Unknown	1		

For peer review only

Table 1 – Patient and GP demographics

* PIRADS – Prostate Imaging-Reporting And Data System v2 score of 1-2 suggest clinically significant prostate cancer is unlikely and biopsy not indicated. A PIRADS score of 3-5 indicates at least one suspicious area of the prostate that warrants biopsy.

Patient experiences of the prostate cancer diagnostic pathway

For peer review only - http://bmjopen.bmj.com/site/about/guidelines.xhtml

We identified three main themes with interlinking sub-themes (see figure 3): Cancer beliefs, Communication, and Pathway experience.

Figure 3

Cancer beliefs

The decision for patients to see their doctor about potential prostate problems was not undertaken in isolation (*Outside influences*). The experiences of family members and friends shaped the patients' expectations, and family members and partners often encouraged men to be tested:

> *"Obviously back then he [dad] was in his mid to late 60s. And I think I didn't really know about it until he'd gone for his MRI and got the results and everything, and then all of a sudden he sat me down and told me all about it." P20 (London, <65)*

Most patients' attitudes towards the possibility of a diagnosis of cancer (*Attitude to diagnosis*) were fairly relaxed. Many seemed philosophical about the prospect:

> *"it is what it is" P03 (Devon, <65)*

The reactions of patients who had a diagnosis of prostate cancer (*Reaction to findings*) were mixed, ranging from despondence to quick acceptance:

> *"Not fair. No, it's... it's not fair on... on anyone, not just me. It isn't fair on anyone." P01 (Devon, 65+)*

Communication

For peer review only

The absence of the use of the word cancer (‘*C word*’) was evident in interviews with many patients. Patients also reported a reluctance from clinicians to raise cancer specifically as a possibility during a consultation, even if they were referred for urgent tests to rule out a diagnosis of prostate cancer:

> *“And then this developed.” P01 (Devon, 65+)*
>
> *“For me, my... my dad had it roughly about eight, nine years... eight to ten years ago, I suppose. He had it.” P20 (London, <65)*
>
> *“The only thing that I found was you were given leaflets that mention a lot about cancer but no one actually really, sort of like said to me, you know, there’s a possibility that you could have cancer or you know, that you’re just being given leaflets and such, and no one really explained to you that there is a possibility. ” P25 (Devon, <65)*

The mode of communication to the patient from clinicians (*personal contact*) appeared to directly affect their experiences of the pathway. Most London patients sat down with their consultant and reviewed their MRI results together, whereas many patients in Devon received their results via a letter:

> *“I think it was interesting to see this sort of slightly darker little, ti… little circular area that he thought might be cancerous and… and also explain that they would need to take some samples from another area which… which was more the normal colour of the whole gland for comparison.” P13 (London, 65+)*
>
> *“Most of the letters go to the GP and I just get a copy.” P23 (Devon, 65+)*

Communicating the meaning (*conveying significance*) of the results of the MRI and other tests performed was very important to help patients and their partners understand what the results mean for them as an individual:

> *“Yeah, so apparently, because this is mid-rank they said that if you just got the first circle, the first ones in, they probably wouldn’t have done anything about it and you could have*

For peer review only - http://bmjopen.bmj.com/site/about/guidelines.xhtml

For peer review only

had a lot of years where you just monitor that. But because P03 was mid-stage, they said we have to do something." P03's partner (Devon, <65)

Despite most of the patients having undergone a prostate MRI by the time of their interview, there were still limited understanding of the MRI results for some patients (*Gaps in understanding*). More patients from Devon reported these gaps, which often appeared to be a result of communication breakdown between the patient and the doctor:

"Umm… I think, all I know is those letters passed to and fro between the urologist and my GP, and I'm copied in on these things and there was some mention of an abnormality on the left hand side or somewhere or other on the prostate. That's all I know." P23 (Devon, 65+)

Pathway experience

Patients entered the pathway in different ways, with varied length of time and diagnostic work-up prior to urgent suspected cancer referral (*Mixed routes*). For patients in Devon, the prostate cancer pathway required a number of individual appointments, whereas most patients in London received their MRI results on the same day or soon after which was well received (*Appointments burden*):

"I had a PSA of, I think it was 4.03, which was fractionally above the four limit. Then they gave me two additional PSAs every three months, so I went back three months later did another PSA and then I think it was about 3.84. Then another one three months later was 4.08. So then I saw a urologist at Exeter and as a precaution they gave me an MRI and the MRI identified an area of concern if you like [inaudible]. Then I had a biopsy and what that identified was that the area of concern that the MRI identified, there was no cancer, but there was cancer in another area." P04 (Devon, 65+)

For peer review only - http://bmjopen.bmj.com/site/about/guidelines.xhtml

"so… the scan, you get the result within minutes, and even though I had to wait perhaps an hour before I actually saw the doctor but that's a lot less than three months." P05 (London, 65+)

Patient interviewees were generally positive about undergoing investigations for possible prostate cancer, including blood tests and MRI. Most, but not all, patients reported that undergoing an MRI of the prostate was not a significant undertaking (*Test acceptability*):

"I'd go for any scan, anything like that. Needles don't bother me, scans don't bother me." P21 (Devon, 65+)

GP Experiences of the prostate cancer diagnostic pathway

We identified three main themes: Imperfect information, Managing uncertainty, and Contextual influences (see figure 4).

Figure 4

Imperfect information

GPs spoke at length about the limitations of the current primary care diagnostic pathway for prostate cancer, and about having imperfect information on which to base their clinical decisions. A few GPs described a sense of inevitability about patients presenting with lower urinary tract symptoms at some point as they entered their later years (*Non-specific presentation*):

"It's a bit of a grey area so you're kind of waiting for patients to develop symptoms and come to see you" GP03 (Male, London, 31-40)

For peer review only

As described earlier, GPs experienced men refusing to have a prostate examination when prostate cancer is suspected (*Examination acceptance*). GPs reported different reasons for this, and perceived that patients may still be worried even if the prostate feels normal:

> *"I've had patients before who even will have got a high PSA decline, a rectal examination because they've previously had some, kind of, you know, traumatic experience or whatever." GP04 (Female, Devon, 41-50)*

GPs from both regions did not hold back in sharing their opinions about the PSA blood test, and its usefulness (or lack thereof) in helping them make clinical decisions about which men to refer for further testing for possible prostate cancer (*GP test limitations*):

> *"I think if there's one test you could un-invent, I think PSA would be that…" GP02 (Male, Devon, 31-40)*

GPs working in the NHS cannot currently order an MRI of the prostate; the request must come from a secondary or tertiary care clinician. London GPs were more likely to be positive about the concept of a prostate MRI:

> *"I think it will be a really useful idea" GP03 (Male, London, 31-40)*
> *"Well, it's great, but it's not available to me. It's not something I decide on." GP05 (Female, Devon, 41-50)*

<u>Managing uncertainty</u>

GPs made efforts to share their diagnostic dilemma with patients where possible, and consulted guidelines and their local urology specialists in managing uncertainty in their decisions about which men to refer to secondary care. Prior to referral, GPs tried to make their patients understand the limitations of the current diagnostic pathway (*counselling patient*):

For peer review only

"But I always would tell patients that it's not 100% and that both my examinations, whether it's a digital rectal or a PSA, are not 100% and it can be raised even without having cancer." GP03 (Male, London, 31-40)

Whilst most GPs reported feeling satisfied with their local urology service (see *health service & guidance* below), some Devon GPs reported inconsistencies in the advice and management plans for their patients that came back from hospital specialists (*seeking advice*):

"I mean, we try to follow the guidelines but, as I say, we find mystifying as to the variation in the urology advice that comes back in terms of who to follow and who not to…" GP04 (Female, Devon, 41-50)

<u>Contextual influences</u>

A spectrum of broader influences had an effect on when patients chose to present to their GP with concerns about prostate cancer, and the consultation itself (*Gender, society & culture*). Some GPs noted a reticence of men to seek healthcare:

"I think men don't... it's such a sweeping statement but men don't like coming to the doctor" GP07 (Female, London, 31-40)

Consistent with the patient interviews (*outside influences*), the GPs reported that it was often the wives and partners encouraging male patients to seek help and advice:

"…the majority of men I see who mention prostate cancer it's because their wives have asked them to come and they're worried." GP07 (Female, London 31-40)

Cultural and ethnic norms relating to the patient and their partners also influenced the consultation and acceptance of prostate examination, which were more commonly reported by GPs working in London:

For peer review only - http://bmjopen.bmj.com/site/about/guidelines.xhtml

"And over here I notice there are some patients of south Indian descent where, it's [DRE] almost like a taboo really." GP03 (Male, London, 31-40)

GPs in both regions were aware of the influence of news and media stories relating to prostate cancer that were encouraging patients with symptoms or concerns to see their GP and get tested:

"…there was a lot in the media recently with prostate and testicular cancer, actually which is a good thing, because we had a… I had suddenly quite a few men coming in requesting the blood test." GP09 (Female, London, 41-50)

GPs felt that most patients were aware of prostate cancer and that tests were available for it. Awareness of MRI of the prostate was lower than for the PSA blood test (*Patient awareness*):

"Lots of people are aware of the PSA" GP07 (Female, London, 31-40)
"I think a few of them might have said, "I've heard there's a new test around." I don't think anyone's come in and said, "I'd like to have that MRI test." GP04 (Female, Devon, 41-50)

The decision-making of GPs was also affected by their own experiences in their personal and professional lives (*Personal & professional experience*). GPs demonstrated an awareness of how these experiences shaped their approach:

"…my dad has prostate cancer that was picked up with a raised PSA. And my stepfather has prostate cancer which was picked up by a raised PSA. Both completely asymptomatic. So I think that also affects how you... how you practice and you know, as clinicians we do take on our life experiences and we can't help but have that shape how... how we work." GP07 (Female, London, 31-40)

The health service context in which GPs practise was another significant influence on their approach to patients with possible prostate cancer (*Health services & guidelines*). They often rely on guidance from a number of sources, including national guidelines and local urology services:

For peer review only

"I think we've got some, you know, very good local colleagues who offer good pragmatic advice and are very approachable." *GP02 (Male, Devon, 31-40)*

For peer review only

Discussion

Principal findings

Patients' experiences of more traditional and 'one-stop' prostate cancer diagnostic pathways incorporating MRI showed some key similarities and differences. The communication between patients and healthcare teams significantly affected the patients' overall experience and their understanding of MRI results. MRI appeared to be an acceptable and low burden test for patients. Compared to patients attending a 'one-stop' clinic, patients following more traditional diagnostic pathways felt that longer waits for tests, more appointments to attend, and increased travel requirements all impacted on their pathway experience. GPs faced challenges in dealing with uncertainty and the perceived limitations of symptoms, examination and tests available to them for diagnosing prostate cancer with confidence. GP awareness, understanding and access relating to MRI was limited in both regions, and they reported some variation in local guidelines and specialist urology advice.

Strengths and weaknesses

This study recruited a diverse sample of patients undergoing prostate MRI in terms of age, geographical distribution and ethnicity. Recruitment of patients from a range of ethnic minority backgrounds in cancer research can be challenging[18], so identifying and interviewing these patients as part of the study was key. Participants were recruited from two regions with contrasting prostate cancer diagnostic pathway designs. This enabled identification of key similarities and differences in the experiences of patients and GPs engaging with 'one stop' and more traditional pathways to help inform pathway design that could improve patient experience.

The influence of the researcher on data collection and analysis is important to consider in qualitative research. Participants were aware that SM was a clinician performing the study as part of a Cancer Research UK funded PhD, and that may have given some level of respectability and authority to the interviewer and the study. Some patients and GPs reported that men were less comfortable seeing a female GP about problems relating to the prostate, so having a male interviewer may have helped patient participants be more comfortable and open in the interviews. GP participants may have been more comfortable in talking to a peer in these

For peer review only - http://bmjopen.bmj.com/site/about/guidelines.xhtml

interviews; peer discussions are a common part of professional practice for GPs in the form of Balint groups[19] and annual appraisal by a fellow GP[20].

Some GPs were reluctant to engage in any discussion about prostate MRI as they felt it was outside their current scope of practice, and may have been focused on the more traditional (pre-MRI) prostate cancer pathway. MRI is a new test for prostate cancer, and has only recently been integrated into diagnostic pathways. GPs are not currently able to request an MRI of the prostate, and access to MRI for other indications varies across the NHS. In this context, data gathered from GP participants was not as rich as the data collected from the patients and more limited insights were generated. A further potential limitation to the clinician insights gained from this study is that only GPs were recruited, and not other clinicians involved in the prostate cancer diagnostic pathway such as urologists or radiologists

Relation to published literature

This is the first study the authors are aware of to explore experiences of the modern pre-biopsy MRI prostate cancer diagnostic pathway from the perspective of patients and GPs. Similar studies with patients or GPs have been conducted before MRI emerged as a diagnostic test for prostate cancer. Ruseckaite *et al* interviewed 10 GPs from metropolitan Melbourne and a regional part of Victoria, Australia regarding their perceptions of prostate cancer care. In line with the findings of this study, most men were willing to have a PSA blood test, and some GPs had to grapple with inconsistent guidance from specialist bodies[21]. Evans *et al* assessed men's experience of PSA testing in primary care in Wales, and also found that social networks and media stories influenced patient demand for testing. In contrast to the views of GPs in this study, the men in the study by Evans *et al* felt decision-making about testing was doctor-centred rather than shared or patient-centred[22].

In contrast to the limited amount of published evidence on patient experience of cancer diagnostic pathways, there have been many more studies on patient perspectives of prostate cancer screening that identified some key themes consistent with the findings of this study[27]. James et al found 'social prompting' from family and friends to consult their doctor about prostate

For peer review only

cancer testing is a prominent theme in prostate cancer screening studies, similar to the 'outside influences' sub-theme that came from this research. Interestingly patients in prostate cancer screening studies also describe the 'physiological and symptomatic obscurity' of prostate cancer, which the GPs in this study were acutely aware of.

'Communication' of the results of diagnostic testing and a new diagnosis of cancer was another key theme emerging from interviews in this study that has a wealth of published research, and quality of communication can impact on patient and clinician experiences of diagnostic pathways. A number of studies have found deficiencies in communication from clinicians to patients about prostate cancer diagnostic testing and the results of tests, similar to the experience of some patients interviewed[28,29]. This study found patients valued the opportunity to discuss test results and understand the implications of the findings when they had it, whilst others felt communication about their test results was largely bypassing them between the specialist and GP. Interventions for improving prostate cancer patient engagement and empowerment have previously been developed[30], which may have a role in improving patient experience of modern prostate cancer diagnostic pathways.

Implications for patients, clinicians and health service design

Men's experiences of the prostate cancer diagnostic pathway are influenced by the appointment burden they face to receive a diagnosis; the mode of communication used by GPs and specialists to communicate test results; and requirements for travel to attend clinic appointments and tests. Significant challenges remain for GPs owing to the limitations of the current clinical signs and tests they rely on to identify possible prostate cancer cases. Men seemed broadly positive about MRI as a new test for prostate cancer, whereas GPs were equivocal owing to a lack of awareness and access. Improvements to patient experience of prostate cancer diagnostic pathways could be achievable through shorter time intervals to MRI, reduced outpatient appointment burden for patients, and access to more accurate and reliable triage testing in primary care.

For peer review only

Funding

SWDM is supported by the Can Test Collaborative, which is funded by CRUK (C8640/A23385). FMW and WH are co-directors of CanTest. HA's research is supported by core funding from the United Kingdom's National Institute of Health Research (NIHR) Imperial Biomedical Research Centre. Ahmed currently receives funding from the Wellcome Trust, Medical Research Council (UK), Cancer Research UK, Prostate Cancer UK, National Institute for Health Research (UK), The Urology Foundation, BMA Foundation, Imperial Health Charity, NIHR Imperial BRC, Sonacare Inc., Trod Medical and Sophiris Biocorp for trials in prostate cancer. DE-E receives funding from the Urology Foundation, the BMA Foundation for Medical Research, Imperial Health Charity and the Royal College of Surgeons of England.

Competing interests

HA was a paid medical consultant for Sophiris Biocorp in the previous 3 years. The remaining authors have no conflicts of interest to declare.

Acknowledgements

The authors would like to thank the participants for sharing their time and experiences for this study. We would also like to thank Pauline Sibley and Victor Mariano (Royal Devon & Exeter NHS Foundation Trust) for their assistance with patient recruitment.

Contributors

SWDM, WH and FMW conceived the study. SWDM developed the research protocol with contributions from FMW, AF, and SA. HA, JM and DE-E were local investigators who supervised recruitment into the study. SWDM undertook all interviews. SWDM, FMW, AF and SA performed the analysis. SWDM drafted the first version of the manuscript. All authors contributed to the development of the manuscript, and approved the final submitted version.

Ethics approval

Ethics committee approval was received from the NHS HRA South-West Frenchay research ethics committee (REC reference 19/SW/0040).

For peer review only

Patient consent for publication

Patient consent for publication of anonymised individual remarks was obtained at the time of recruitment into the study.

Data sharing statement

All data requests should be submitted in writing to the corresponding author

Disclaimer

The views expressed in this publication are those of the author(s) and not necessarily those of the NIHR or the Department of Health and Social Care.

For peer review only

References

1 Forster AS, Rubin G, Emery JD, *et al.* Measuring patient experience of diagnostic care and acceptability of testing. *Diagnosis* 2021;**0**. doi:10.1515/dx-2020-0112

2 NICE. Suspected cancer: recognition and referral. 2015;:1–95.https://www.nice.org.uk/guidance/ng12 (accessed 13 Oct 2021).

3 McPhail S, Johnson S, Greenberg D, *et al.* Stage at diagnosis and early mortality from cancer in England. *British journal of cancer* 2015;**112**:S108–15. doi:10.1038/bjc.2015.49

4 Sandager M, Jensen H, Lipczak H, *et al.* Cancer patients' experiences with urgent referrals to cancer patient pathways. *European Journal of Cancer Care* 2019;**28**:1–11. doi:10.1111/ecc.12927

5 Fraser J, Pearson C, Shelton J, *et al.* Using linked English cancer registration data to assess variation in diagnostic pathway length. *International Journal of Population Data Science* 2019;**4**:121.

6 Clements A, Watson E, Rai T, *et al.* The PSA testing dilemma: GPs' reports of consultations with asymptomatic men: a qualitative study. *BMC Family Practice* 2007;**8**:35. doi:10.1186/1471-2296-8-35

7 Pickles K, Carter SM, Rychetnik L. Doctors' approaches to PSA testing and overdiagnosis in primary healthcare: A qualitative study. *BMJ Open* 2015;**5**. doi:10.1136/bmjopen-2014-006367

8 NICE. Prostate Cancer: Diagnosis and Management. ng131. 2019. doi:10.1097/00005392-200308000-00097

9 Merriel SWD, Walter FM, Hamilton W. Integrating multiparametric MRI into prostate cancer diagnostic pathways within the NHS and internationally – A diagnostic pathways mapping study. In: *Cancer REsearch UK 5th Biennial Early Diagnosis Research Conference*. Birmingham: : Cancer Research UK 2019. https://www.cancerresearchuk.org/health-professional/diagnosis/early-diagnosis-initiative/early-diagnosis-research-conferences/2019-conference-programme-and-registration

10 Drost FJ, Osses DF, Nieboer D, *et al.* Prostate MRI, with or without targeted biopsy and standard biopsy for detecting prostate cancer: A Cochrane systematic review and meta-analysis. *Cochrane Database of Systematic Reviews* 2019;:CD012663. doi:10.1016/s1569-9056(19)30534-2

11 Scott SE, Walter FM, Webster A, *et al.* The model of pathways to treatment: Conceptualization and integration with existing theory. *British Journal of Health Psychology* 2013;**18**:45–64. doi:10.1111/j.2044-8287.2012.02077.x

12 Walter F, Webster A, Scott S, *et al.* The Andersen Model of Total Patient Delay: A systematic review of its application in cancer diagnosis. *Journal of Health Services Research and Policy* 2012;**17**:110–8. doi:10.1258/jhsrp.2011.010113

13 Silverman D. *Doing qualitative research*. Sage 2013.

14 Guba EG, Lincoln YS. Competing paradigms in qualitative research. In: *Handbook of Qualitative Resarch*. 1994. 105–17.

15 Denzin NK, Lincoln YS. *The sage handbook of qualitative research*. Sage 2011.

16 Braun V, Clarke V. Using thematic analysis in psychology. *Qualitative Research in Psychology* 2006;**3**:77–101. doi:10.1111/j.1460-2466.1978.tb01621.x

For peer review only - http://bmjopen.bmj.com/site/about/guidelines.xhtml

17 Tong A, Sainsbury P, Craig J. Consolidated criterion for reporting qualitative research (COREQ): a 32- item checklist for interviews and focus group. *International Journal of Qualitative in Health Care* 2007;**19**:349–57. doi:10.1093/intqhc/mzm042

18 Symonds RP, Lord K, Mitchell AJ, *et al.* Recruitment of ethnic minorities into cancer clinical trials: Experience from the front lines. British Journal of Cancer. 2012;**107**:1017–21. doi:10.1038/bjc.2012.240

19 Roberts M. Balint groups: A tool for personal and professional resilience. *Canadian Family Physician* 2012;**58**:2012.

20 NHS England. Appraisal. 2020.https://www.england.nhs.uk/medical-revalidation/appraisers/ (accessed 1 Apr 2020).

21 Ruseckaite R, Evans S, Millar J, *et al.* GPs' Insights into Prostate Cancer Diagnosis and Care in Regional Victoria, Australia. *The Qualitative Report* 2016;**21**:2365–79

22 Evans R, Edwards AGK, Elwyn G, *et al.* It's a maybe test': men's experiences of prostate specific antigen testing in primary care. *British Journal of General Practice* 2007;**57**:303–10.

23 Christensen HM, Huniche L. Patient perspectives and experience on the diagnostic pathway of lung cancer: A qualitative study. *SAGE Open Medicine* 2020;**8**:1–10. doi:10.1177/2050312120918996

24 Rankin NM, York S, Stone E, *et al.* Pathways to lung cancer diagnosis: A Qualitative study of patients and general practitioners about diagnostic and pretreatment intervals. *Annals of the American Thoracic Society* 2017;**14**:742–53. doi:10.1513/AnnalsATS.201610-817OC

25 Howell D, Hart R, Smith A, *et al.* "Unpacking" pathways to lymphoma and myeloma diagnosis: Do experiences align with the Model of Pathways to Treatment? Findings from a UK qualitative study with patients and relatives. *BMJ Open* 2020;**10**. doi:10.1136/bmjopen-2019-034244

26 Bergin RJ, Emery JD, Bollard R, *et al.* Comparing Pathways to Diagnosis and Treatment for Rural and Urban Patients With Colorectal or Breast Cancer: A Qualitative Study. *Journal of Rural Health* 2020;**00**:1–19. doi:10.1111/jrh.12437

27 James LJ, Wong G, Craig JC, *et al.* Men's perspectives of prostate cancer screening: A systematic review of qualitative studies. *PLoS ONE* 2017;**12**. doi:10.1371/journal.pone.0188258

28 Bhuyan SS, Chandak A, Gupta N, *et al.* Patient–Provider Communication About Prostate Cancer Screening and Treatment: New Evidence From the Health Information National Trends Survey. *American Journal of Men's Health* 2017;**11**:134–46. doi:10.1177/1557988315614082

29 Henry SG, Czarnecki D, Kahn VC, *et al.* Patient-physician communication about early stage prostate cancer: Analysis of overall visit structure. *Health Expectations* 2015;**18**:1757–68. doi:10.1111/hex.12168

30 Waldie M, Smylie J. Communication: the key to improving the prostate cancer patient experience. *Canadian oncology nursing journal = Revue canadienne de nursing oncologique* 2012;**22**:129–39. doi:10.5737/1181912x222129133

For peer review only

Figure 1 – South West Prostate Cancer Diagnostic Pathway, NHS Cancer Alliances in South-West Peninsula and Somerset, Wiltshire, Avon & Gloucester (SWAG)

mpMRI – multiparametric magnetic resonance imaging; TRUS – transrectal ultrasound guided biopsy; MDT – multidisciplinary team

Figure 2 – RAPID pathway, Imperial College Healthcare NHS Trust, London

2WW – Two Week Wait pathway; mpMRI – multiparametric magnetic resonance imaging; MRI – magnetic resonance imaging

Figure 3 – Thematic diagram from patient participant interviews

Figure 4 – Thematic diagram from GP participant interviews

For peer review only

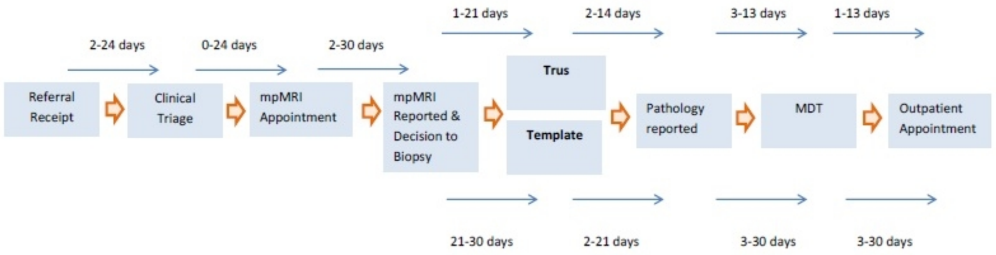

Figure 1 – South West Prostate Cancer Diagnostic Pathway, NHS Cancer Alliances in South-West Peninsula and Somerset, Wiltshire, Avon & Gloucester (SWAG)
mpMRI – multiparametric magnetic resonance imaging; TRUS – transrectal ultrasound guided biopsy; MDT – multidisciplinary team

207x55mm (96 x 96 DPI)

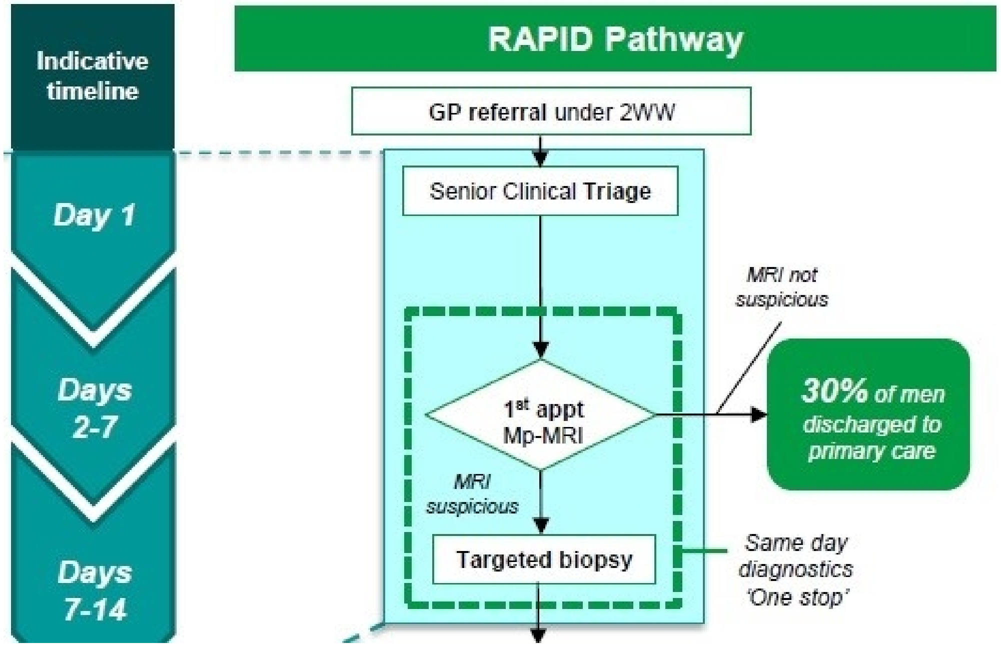

Figure 2 – RAPID pathway, Imperial College Healthcare NHS Trust, London
2WW – Two Week Wait pathway; mpMRI – multiparametric magnetic resonance imaging; MRI – magnetic resonance imaging

140x91mm (96 x 96 DPI)

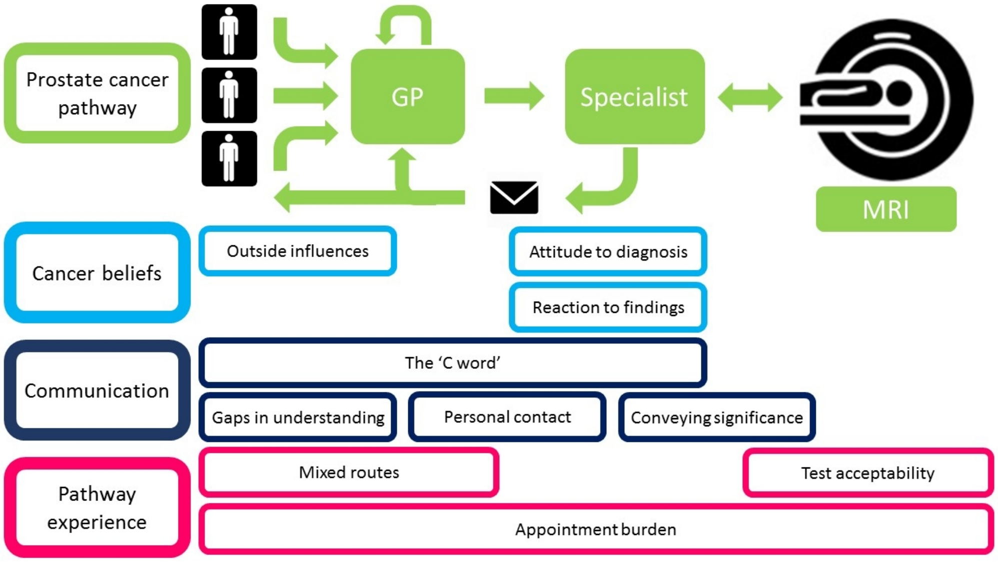

Figure 3 – Thematic diagram from patient participant interviews

338x190mm (96 x 96 DPI)

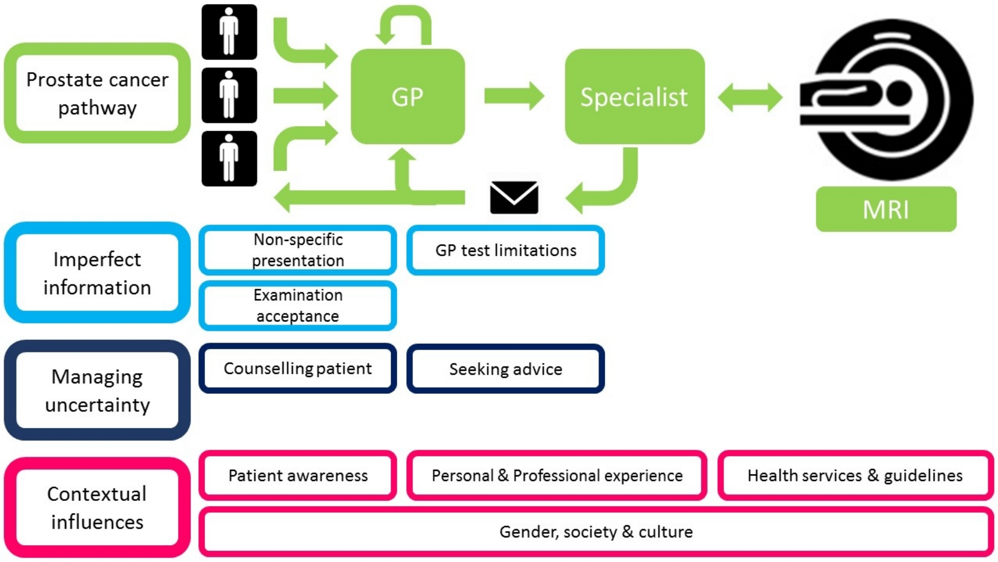

Figure 4 – Thematic diagram from GP participant interviews

338x190mm (96 x 96 DPI)

Patient participant interview guide

Introduction (5mins)
Thank you for agreeing to participate in this interview.

Introduce myself and my role.

This study seeks to understand your knowledge and understanding of diagnostic tests for prostate cancer, and your experiences of the current prostate cancer diagnostic pathway in the region where you live. It is part of my PhD at the University of Exeter. This study has been funded by Cancer Research UK, and has ethical approval from the NHS Health Research Authority and the University of Exeter.

As we've talked about with the consent form, participating in this study is voluntary and you can stop at any time. We want to know about your experiences and what you think, so there are no right or wrong answers.

This interview is being recorded for the purposes of qualitative analysis by the researchers. You can ask for the recording to be stopped at any time. What you say will be kept confidential and anonymous, unless we discuss something that suggests there is a significant risk to yourself or someone else. Everyone being interviewed will be asked the same questions, so if you don't have an answer to any of the questions that's fine, just say so and we can move on.

This interview study if focused on your experience of diagnostic test for prostate cancer. However, if you have a partner, family member or significant other who you wish to be present that's fine. Ideally we would start the interview without them, and then invite them in later on. If you and they are happy for them to participate, they would need to complete a consent form as well.

Ensure participant has copy of participant information sheet

Answer any questions

Ensure consent form is completed correctly

Commence audio recording

For peer review only

For peer review only - http://bmjopen.bmj.com/site/about/guidelines.xhtml

Basic demographics (5 minutes)

"To start with, can you tell me a little bit about yourself and your background"

Check - Age, Ethnicity, City/town lived in

Prostate cancer diagnosis journey (5-10 minutes)

"Now I would like to talk a bit about how you came to have tests for possible prostate cancer."

When did you first notice symptoms (if any)? Which symptoms were they?

How long until consulted you consulted your GP? What affected that decision?

How did GP assess? PSA? DRE?

Decision to refer – what do you remember about that discussion?

mpMRI for prostate cancer (10-15 minutes)

"I would now like to ask some questions about having an MRI scan for possible prostate cancer."

Did you have any concerns or reservations about having an MRI? What were your expectations?

What do you understand about how an MRI works?

What was your experience of having an MRI?

mpMRI result (10 minutes)

"If you are happy to discuss, I would like to ask a few questions about the results of your MRI scan and the next steps."

What was the result of your mpMRI? Did you understand it? How much did you trust the findings?

How were the results communicated? Did you have any questions as a result?

What were you told about the results' meaning?

What else did you discuss with the specialist?

Doctor(s) involved in cancer diagnosis (5 minutes)

"Finally, I would like to ask about your thoughts regarding the way we organise testing for prostate cancer."

What works well? What could be done better?

Who do you feel should be involved in investigating for a possible cancer diagnosis?

If you could design the ideal cancer diagnosis testing service, how would it work?

Interview close (2 minutes)

Thank you for participating in this interview. The data you have provided will be transcribed under a pseudonym, and analysed by the research team. You will be sent a final study report after the analysis has been completed.

If you have any questions or concerns about the study, please contact Ms Pam Baxter at the Research Ethics and Governance Office at the University of Exeter on 01392 723588 or via email p.r.baxter2@exeter.ac.uk Her details are on your patient information leaflet.

For peer review only

For peer review only - http://bmjopen.bmj.com/site/about/guidelines.xhtml

GP participant interview guide

Introduction (5mins)

Thank you for agreeing to participate in this interview.

Introduce myself and my role.

This study seeks to understand your knowledge and understanding of diagnostic tests for prostate cancer, and your experiences of the current prostate cancer diagnostic pathway in the region where you work. It is part of my PhD at the University of Exeter. This study has been funded by Cancer Research UK, and has ethical approval from the NHS Health Research Authority and the University of Exeter.

As we've talked about with the consent form, participating in this study is voluntary and you can stop at any time. We want to know about your experiences and what you think, so there are no right or wrong answers.

This interview is being recorded for the purposes of qualitative analysis by the researchers. You can ask for the recording to be stopped at any time. What you say will be kept confidential and anonymous, unless we discuss something that suggests there is a significant risk to yourself or someone else. This interview is not assessing your clinical competence, and we want to hear about your approach and experiences. Everyone being interviewed will be asked the same questions, so if you don't have an answer to any of the questions that's fine, just say so and we can move on.

This interview study if focused on your experience of diagnostic test for prostate cancer. However, if you have a partner, family member or significant other who you wish to be present that's fine. Ideally we would start the interview without them, and then invite them in later on. If you and they are happy for them to participate, they would need to complete a consent form as well.

Ensure participant has copy of participant information sheet

Answer any questions

Ensure consent form is completed correctly

Commence audio recording

For peer review only

For peer review only - http://bmjopen.bmj.com/site/about/guidelines.xhtml

Basic demographics (5 minutes)
"To start with, can you tell me a little bit about yourself and your background"

Age, Gender, Years of GP experience, Main CCG area you work in

Decision to refer for suspected prostate cancer (10-15 minutes)
"I would like to now move on to your current practice around referring men with suspected prostate cancer for further investigation"

"What symptoms/signs do you enquire about when assessing a man for suspected prostate cancer? How do they affect your decision to refer?"

PSA use – When would you offer it to a man? What are the important points you make about PSA when counselling a man about the test? What do you do with a negative PSA?

"What other factors, if any, affect your decision to refer a man for further investigation?"

"What are the key points you discuss with men when making a referral?"

Diagnostic testing for prostate cancer (15-20 minutes)
"Now I would like to ask some questions about diagnostic tests for prostate cancer."

What is your experience of the prostate cancer diagnosis pathway in your region?
What do you know about current diagnostic tests? How accurate do you believe current diagnostic tests are for prostate cancer?
PROMIS trial – have you heard of it? Are you aware of use of mpMRI for prostate cancer?
Do you feel incorporating mpMRI into the prostate cancer diagnosis pathway would be beneficial for patients? Do you believe it could be cost effective?
What would be the characteristics of an ideal diagnostic test for prostate cancer?

Men diagnosed with prostate cancer (5-10 minutes)
"Finally, I would like to ask about any of your patients who have been diagnosed with prostate cancer"

Are you aware of any of your patients diagnosed with low-grade prostate cancer?
If so, what has been the impact of the diagnosis on patient?
Are you aware of any of your patients being put on active surveillance – what is your experience of interacting with these men after diagnosis?

Interview close (2 minutes)
Thank you for participating in this interview. The data you have provided will be transcribed under a pseudonym, and analysed by the researchers. You will be sent a final study report after the analysis has been completed. If you have any questions or concerns about the study, please contact Ms Pam Baxter at the Research Ethics and Governance Office at the University of Exeter on 01392 723588 or via email p.r.baxter2@exeter.ac.uk

For peer review only - http://bmjopen.bmj.com/site/about/guidelines.xhtml

‘Acceptability, understanding and experience of diagnostic tests for prostate cancer: a qualitative study with patients and GPs’

IRAS ID: 259602

Protocol Version Number: 1.0

Protocol date: 21/01/2019

Sponsor: University of Exeter

Sponsor Protocol Reference Number: 1819/03

Chief Investigator: Dr Sam Merriel

Supervisors:

Dr Fiona Walter, University of Cambridge

Dr Alice Forster, University College London

Professor Willie Hamilton, University of Exeter

This protocol has regard for the HRA guidance and order of content

For peer review only

For peer review only - http://bmjopen.bmj.com/site/about/guidelines.xhtml

SIGNATURE PAGE

The undersigned confirm that the following protocol has been agreed and accepted and that the Chief Investigator agrees to conduct the study in compliance with the approved protocol and will adhere to the principles outlined in the Declaration of Helsinki, the Sponsor's SOPs, and other regulatory requirement.

I agree to ensure that the confidential information contained in this document will not be used for any other purpose other than the evaluation or conduct of the investigation without the prior written consent of the Sponsor

I also confirm that I will make the findings of the study publically available through publication or other dissemination tools without any unnecessary delay and that an honest accurate and transparent account of the study will be given; and that any discrepancies from the study as planned in this protocol will be explained.

For and on behalf of the Study Sponsor:

Signature:

Date: 30/01/2019

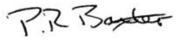

Name (please print): Ms Pam Baxter

Position: Senior Research Governance Officer

University of Exeter

Chief Investigator:

Date: 30/01/2019

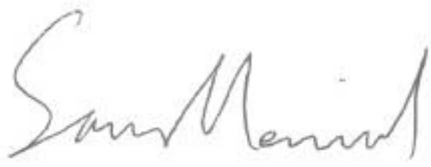

Signature:

Name: (please print): Dr Sam Merriel

For peer review only

For peer review only - http://bmjopen.bmj.com/site/about/guidelines.xhtml

LIST of CONTENTS

**GENERAL INFORMATION**	**Page No.**
HRA PROTOCOL COMPLIANCE DECLARATION AND SIGNATURE PAGE	ii
LIST OF CONTENTS	iii
KEY STUDY CONTACTS	iv
STUDY SUMMARY	v
FUNDING	v
ROLE OF SPONSOR AND FUNDER	vi
ROLES & RESPONSIBILITIES OF STUDY STEERING GROUPS AND INDIVIDUALS	vi
**SECTION**	
STUDY FLOW CHARTS	1
1. BACKGROUND	4
2. RATIONALE	4
3. THEORETICAL FRAMEWORK	5
4. RESEARCH QUESTION/AIM(S)	5
5. STUDY DESIGN/METHODS	6
6. SAMPLE AND RECRUITMENT	7
7. ETHICAL AND REGULATORY COMPLIANCE	8
8. DISSEMINATION POLICY	12
9. REFERENCES	12
10. APPENDICES	15

For peer review only - http://bmjopen.bmj.com/site/about/guidelines.xhtml

KEY STUDY CONTACTS

Chief Investigator<br>('The researcher')	Dr Sam Merriel<br>Clinical Senior Research Fellow, Exeter Medical School, University of Exeter<br>1.18, College House, St Luke's Campus, University of Exeter, Heavitree Road, Exeter EX1 2LU<br>s.w.d.merriel@exeter.ac.uk<br>01392 726090
Supervisors	Dr Fiona Walter<br>Principal Researcher in Primary Care Cancer Research, Department of Public Health and Primary Care, University of Cambridge<br>The Primary Care Unit, University of Cambridge School of Clinical Medicine, Box 113 Cambridge Biomedical Campus, Cambridge, CB2 0SR<br>fmw22@medschl.cam.ac.uk<br>01223 762514<br>Dr Alice Forster<br>Senior Research Fellow, Institute of Epidemiology & Health, Faculty of Population Health Sciences, University College London<br>1-19 Torrington place, London, WC1E 6BT<br>alice.forster@ucl.ac.uk<br>Professor Willie Hamilton<br>Professor of Primary Care Diagnostics, Exeter Medical School, University of Exeter<br>1.17, College House, St Luke's Campus, University of Exeter, Heavitree Road, Exeter EX1 2LU<br>w.hamilton@exeter.ac.uk<br>01392 726097
Sponsor	University of Exeter<br>Sponsor's representative<br>Ms Pam Baxter<br>Senior Research Governance Officer<br>Research Ethics and Governance Office,<br>University of Exeter<br>Lafrowda House, St Germans Road, Exeter,<br>Devon, EX4 6TL<br>p.r.baxter2@exeter.ac.uk<br>01392 723588

For peer review only

For peer review only - http://bmjopen.bmj.com/site/about/guidelines.xhtml

Funder(s)	Cancer Research UK

STUDY SUMMARY

Study Title	'Acceptability, understanding and experience of diagnostic tests for prostate cancer: a qualitative study with patients and GPs'
Internal ref. no. (or short title)	Acceptability of mpMRI for prostate cancer diagnosis in primary care
Study Design	Qualitative interview study
Study Participants	Males who have undergone multiparametric Magnetic Resonance Imaging (mpMRI) for suspected prostate cancer.<br>General Practitioners (GPs)
Planned Size of Sample (if applicable)	Purposive sample of approximately 10 GPs and 20 patients
Planned Study Period	01/03/2019 – 01/07/2020
Research Question/Aim(s)	**Aim**<br>To understand, from the perspective of patients and GPs, the acceptability of multiparametric magnetic resonance imaging for men as a diagnostic test for prostate cancer<br>**Objectives**<br>1. Elicit men's experiences of diagnostic tests for suspected prostate cancer<br>2. Explore the knowledge and understanding of diagnostic tests for suspected prostate cancer amongst patients and GPs<br>3. Understand the acceptability of mpMRI as a diagnostic test for prostate cancer from a patient's perspective<br>4. Understand the acceptability of mpMRI as a diagnostic test for prostate cancer from a GP's perspective

FUNDING AND SUPPORT IN KIND

FUNDER(S)	FINANCIAL AND NON FINANCIALSUPPORT GIVEN

For peer review only - http://bmjopen.bmj.com/site/about/guidelines.xhtml

Cancer Research UK	Funding: CanTest (Cancer Research UK Catalyst Award) - Clinical Research Fellow<br>Duration: 3 years 8 months (at 60% FTE)<br>Start date: 02/08/2018<br>Travel costs for researchers and participants to attend face to face interviews<br>Service support costs for recruitment sites<br>Transcription of interviews
National Institute for Health Research Clinical Research Network	Adoption of study onto NIHR portfolio<br>Recruitment of GP participants through CRN practices

ROLES AND RESPONSIBILITIES OF STUDY MANAGEMENT COMMITEES/GROUPS & INDIVIDUALS

The study management group with comprise the researcher, the supervisors, and a PPI representative. The researcher will report to the group, who will oversee the recruitment, data collection and analysis for the duration of the study. The study management group will be independent from the sponsor and the funders.

PROTOCOL CONTRIBUTORS

The study sponsor provided support to the researcher in the preparation of the study protocol, consent forms, patient information leaflets and interview guides. The sponsor will have no role in any other aspect of the conduct, analysis, or dissemination of the study.

The funder had no role in any aspect of the preparation of the study, and will have no role in the conduct, analysis or dissemination of the study.

Patients and members of the public have been involved with writing the lay summary, consent forms, patient information leaflets and interview schedules.

SPONSOR

The study sponsor (the University of Exeter) has ensured that the research team, research protocol and research sites are suitable and that indemnity arrangements are in place. In reviewing the research and ethics documentation, the sponsor has further ensured that appropriate risk management is in place and that the study is managed and conducted in accordance with relevant legislation and codes of good practice. The sponsor will ensure that relevant approvals are in place before the study begins, that the study is conducted in accordance with the protocol and relevant approvals, and that appropriate record-keeping and data management is maintained. The sponsor must approve any study amendments or modifications and will be notified of any significant developments or adverse events in accordance with appropriate guidelines. The sponsor has reviewed plans for data storage and retention and plans for dissemination of the research findings.

KEY WORDS: Prostate cancer; MRI; diagnosis; primary care; acceptability

For peer review only - http://bmjopen.bmj.com/site/about/guidelines.xhtml

For peer review only

For peer review only - http://bmjopen.bmj.com/site/about/guidelines.xhtml

STUDY FLOW CHART 1 – Patient participant recruitment at Imperial College Healthcare

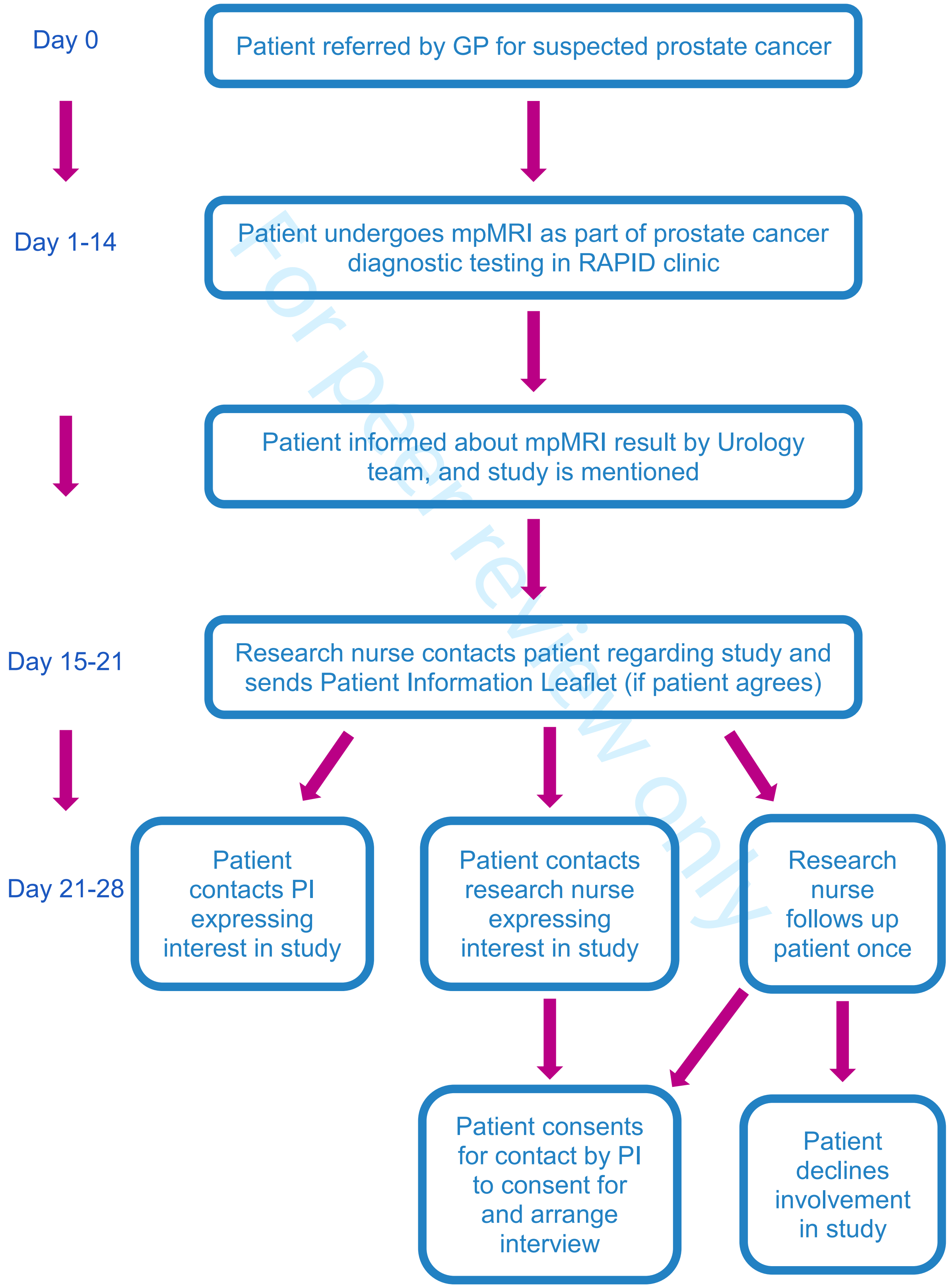

For peer review only - http://bmjopen.bmj.com/site/about/guidelines.xhtml

STUDY FLOW CHART 2 – Patient participant recruitment at Royal Devon & Exeter

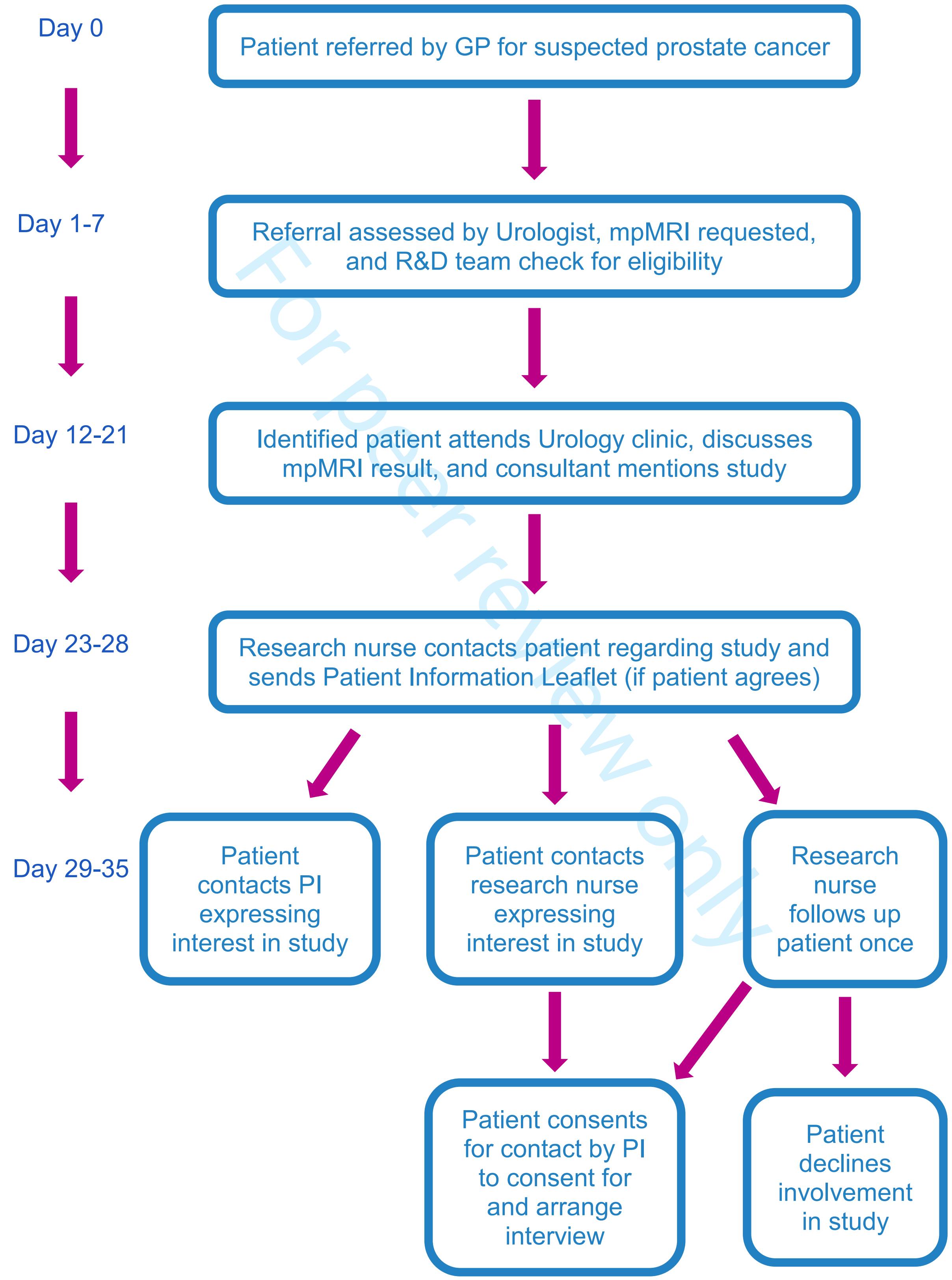

For peer review only

For peer review only - http://bmjopen.bmj.com/site/about/guidelines.xhtml

STUDY FLOW CHART 3 – GP participant recruitment via Clinical Research Networks

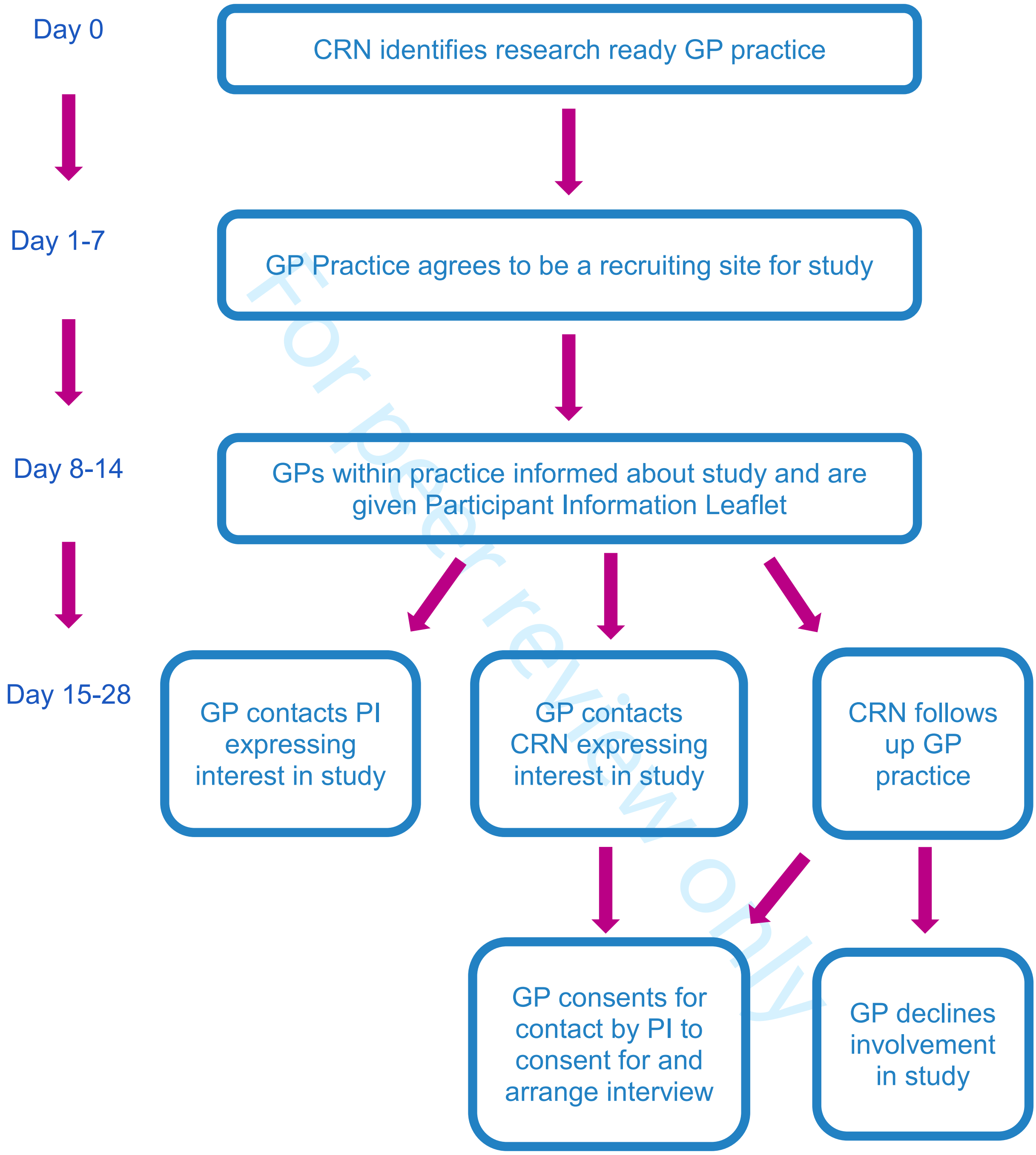

For peer review only - http://bmjopen.bmj.com/site/about/guidelines.xhtml

STUDY PROTOCOL

'Acceptability, understanding and experience of diagnostic tests for prostate cancer: a qualitative study with patients and GPs'

1 BACKGROUND

Implementation of diagnostic tests into routine clinical practice should follow a rigorous process of evaluation from showing analytical validity and diagnostic accuracy, through to acceptability and cost effectiveness. A number of frameworks for assessing and evaluating tests for use in healthcare have been proposed(1–5). They suggest the test should be able to be performed by the operator(s); it should demonstrate more patient benefit than harm; it should be cost effective relative to currently available tests; it should be able to be integrated into the diagnostic pathway; and it should be acceptable to patients and clinicians.

Cancer diagnosis pathways in the NHS in the UK involve primary and secondary care clinicians(6). Some diagnostic tests can be ordered through 'direct access' by a patient's General Practitioner (GP) if they present with symptoms or signs that could indicate an undiagnosed cancer. These include gastroscopy for oesophageal or gastric cancer; colonoscopy for bowel cancer; flexible sigmoidoscopy for rectal cancer; and CT or MRI head for brain tumours(6). Diagnostic tests for prostate cancer currently requires a referral to secondary care.

The current gold standard diagnostic test for prostate cancer is a transrectal ultrasound-guided (TRUS) biopsy of the prostate. TRUS biopsy procedures take 6-12 samples from different regions of the prostate, which are then examined by a histopathologist for signs of prostate cancer(7). TRUS biopsy carries a risk of infection and sepsis, and there is a risk of under- or misdiagnosis as a result of the random nature of sampling the prostate(8). Multiparametric MRI (mpMRI) scanning of the prostate, and reporting using the PiRADS(9) reporting system, has recently been compared to TRUS biopsy in recent large, multicentre trials(10,11) with favourable results in terms of diagnostic accuracy. Few studies have been performed assessing other aspects of the implementation of mpMRI for prostate cancer diagnosis, including patient experience and clinician acceptability.

2 RATIONALE

Prostate cancer is the most common malignancy in males in the United Kingdom(UK)(12). Whilst prostate cancer is responsible for a significant number of cancer-related deaths, the 5- and 10-year survival rates for men with prostate cancer is high(13). This has partly been driven by an increase in the numbers of clinically insignificant prostate cancer cases being diagnosed in the last three decades(14). Better diagnostic tests and diagnostic pathways are needed to reduce rates of over-diagnosis of clinically insignificant prostate cancer, and mpMRI may have a role in this. Following on from the PROMIS trial(10) and others like it(11,15,16), NHS England issued guidance for 'Implementing a timed prostate cancer diagnostic pathway' to NHS Cancer Alliances, reinforcing the benefits for patients of integrating mpMRI into local diagnostic pathways(17).

Studies of patient acceptance of TRUS prostate biopsy for prostate cancer, the current diagnostic test, focus on prevalence of side effects and patient anxiety relating to the test(18–20). Two studies to date have assessed patient acceptance of mpMRI guided biopsy, which also involved questionnaires assessing side effects and attitudes towards the test(21,22). There are no studies that examine acceptability of mpMRI as a diagnostic test for prostate cancer with any theoretical underpinning, and questions remain about men's experience of undergoing the test and receiving the results. There are also very few studies of General Practitioners (GPs), or primary care clinicians, exploring their understanding of diagnostic tests for prostate cancer.

For peer review only - http://bmjopen.bmj.com/site/about/guidelines.xhtml

3 THEORETICAL FRAMEWORK

Acceptability of diagnostic tests has been measured in a number of ways, but no agreed definition for acceptability exists(23). Sekhon et al have proposed a ‘Theoretical Framework of Acceptability’ relating to healthcare interventions, not just diagnostic tests, which includes seven key constructs (See Figure 1): Affective attitude, Burden, Ethicality, Intervention coherence, Opportunity costs, Perceived effectiveness, Self-efficacy(24). This Framework has been developed to be applicable to both patients and clinicians involved in healthcare interventions, and has a number of key constructs that are particularly relevant to the study aims. Eliciting how a patient feels about undergoing mpMRI (‘Affective attitude’), the extent to which they understand the test and its purpose (‘Intervention coherence’), and how likely they perceive mpMRI will achieve the purpose of diagnosing prostate cancer (‘Perceived effectiveness’) will aid understanding in the acceptability of mpMRI as a diagnostic test.

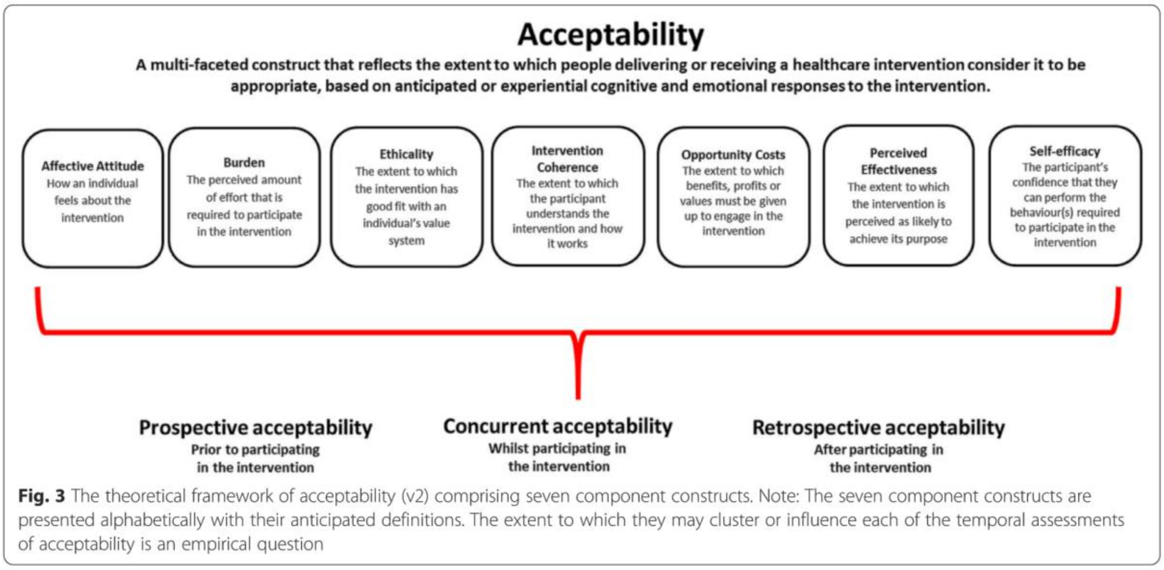

Figure 1 – Sekhon’s Theoretical Framework of Acceptability(24)

Qualitative research methods lend themselves to answering questions of patient and clinician acceptability with regard to diagnostic tests. Such methods allow researchers to “uncover the nature of a person’s experience with a phenomenon”, such as cancer and “understand what lies behind any phenomena”(25). Interview studies provide the opportunity to dig deeper and explore how and why patients and clinicians form their beliefs and understanding. It is assumed by the researchers that every man will experience the prostate cancer diagnostic pathway and the diagnostic tests differently, influenced by both internal and external factors. Therefore, a constructivist approach will be taken to capture a range of experiences(26).

4 RESEARCH QUESTION/AIM(S)

Aim - To understand, from the perspective of patients and GPs, the acceptability of multiparametric magnetic resonance imaging for men as a diagnostic test for prostate cancer

4.1 Objectives

1. Elicit men’s experiences of diagnostic tests for suspected prostate cancer
2. Explore the knowledge and understanding of diagnostic tests for suspected prostate cancer amongst patients and GPs

For peer review only - http://bmjopen.bmj.com/site/about/guidelines.xhtml

3. Understand the acceptability of mpMRI as a diagnostic test for prostate cancer from a patient's perspective
4. Understand the acceptability of mpMRI as a diagnostic test for prostate cancer from a GP's perspective

4.2 Outcome

This study seeks to understand the acceptability of mpMRI as a diagnostic test for prostate cancer amongst patients and GPs. mpMRI is increasingly being used as part of the assessment of men with suspected prostate cancer by Urologists prior to undertaking a prostate biopsy, however it is unknown how men experience mpMRI scanning. Studies suggest that mpMRI has a negative predictive value of 85-89%(16,27), and that up to 27% of men could avoid a prostate biopsy based on mpMRI findings(11). Within the NHS, some GPs already have the ability to order 'direct access' diagnostic tests for suspected cancers of the oesophagus, stomach, colon, pancreas and brain, and there is some evidence that pre-biopsy mpMRI could also be used as a 'rule out' test in a prostate cancer diagnostic pathway for some patients(28). Before such an approach could be tested and implemented, the experience of patients undergoing mpMRI, and the acceptability of mpMRI as a diagnostic test for patients and GPs needs to be understood.

5 STUDY DESIGN and METHODS of DATA COLLECTION AND DATA ANALYIS

5.1 Study design

This qualitative study will employ semi-structured interviews with men referred from primary care with suspected prostate cancer who have undergone mpMRI, and GPs who have referred men with suspected prostate cancer for further investigation.

5.2 Data collection

Interviews will be conducted by the lead researcher (SM). Interview data will be gathered using an encrypted recording device. The location of the patient interviews will be agreed between the participant and the interviewer prior to the day of the interview. Ideally they will be conducted face-to-face in the patient's home, but other venues and telephone/Skype interviews will be considered. GP interviews will either be held face-to-face at the GP clinic, or via telephone/Skype. The interviewer will utilise a 'buddy system' of informing a colleague if they are travelling to a private residence unaccompanied to conduct an interview with a patient participant.

The interviews will be conducted in a semi-structured manner, allowing participants to share their experiences of diagnostic tests for prostate cancer freely, whilst also meeting the study objectives. Interviews will be supported by topic guides (see 10.1.6 and 10.1.7) for patient and GP participants, which will be used by the interviewer in a flexible way depending on the length and direction of the interview. These topic guides were developed by the researchers based on their experience and knowledge in the field and the study objectives, and will be adapted iteratively as the initial interviews are conducted to enrich data collection.

It is important to treat all participants equally, regardless of their age, culture, education, language ability, or beliefs. Efforts will be made to respect participants' needs, however this study is not sufficiently funded to meet all possible participant needs, such as interpreters for participants with English as their second language.

Following completion of each interview, audio recordings will immediately be downloaded onto an encrypted university laptop computer and the interviewer will make reflective and summary notes. Audio recordings will be transferred to an independent transcribing service securely and transcribed

For peer review only - http://bmjopen.bmj.com/site/about/guidelines.xhtml

verbatim. All participants will be given a pseudonym and any potentially identifying information will be removed.

5.3 Data analysis

A Framework Analysis approach will be taken for this study. Framework analysis is a type of thematic analysis developed by Jane Ritchie and Liz Spencer for applied social policy research(29), and is used in a wide range of areas including health research. Framework Analysis follows seven stages(30):

1. Transcription
2. Familiarisation
3. Coding
4. Developing a working framework
5. Applying framework
6. Charting data into matrix
7. Interpretation

After transcription of the interviews and familiarisation with the data by the investigators, early interview data will be coded using pre-specified codes based on Sekhon's Framework. Refinement and addition of codes and themes will occur with a second researcher (AF) and patient/public representative after coding of initial transcripts using constant-comparison method. The analysis team (SM, AF, and FW) will meet regularly to iteratively develop and agree a coding structure to underpin coding of the remaining transcripts, and a framework will be developed and applied using the agreed codes. SM will perform the final coding of the data. Key themes and narratives within the data will be drawn together from the matrix. Charted data will be imported into NVivo v12 to help manage the data to complete the analysis. Convergence and divergence of views from patients with positive and negative mpMRI scans, and between patients and GPs, will be sought to triangulate key findings.

5.4 Role of the researchers

Three of the researchers (SM, FW, and WH) are trained as GPs, and two are still practicing (SM, FW). All members of the research team will maintain an awareness of their individual biases, beliefs and attitudes that could influence the undertaking of research into men being investigated for prostate cancer. Reflective notes, analysis team meetings, and constant comparison techniques will be used to understand these influences.

6 SAMPLE AND RECRUITMENT

6.1 Eligibility Criteria

This study will recruit participants from two populations;

Patients with suspected prostate cancer who have undergone mpMRI as part of their diagnostic workup.

GPs who have referred at least one male for investigation for suspected prostate cancer within the preceding 12 months.

6.2 Sampling

6.2.1 Size of sample

Approximately 30 participants (10 GPs and 20 patients) will be interviewed for this study, although the final number of participants will depend on when no new themes emerge during interview coding.

For peer review only - http://bmjopen.bmj.com/site/about/guidelines.xhtml

6.2.2 Sampling technique

A purposive sampling approach will be taken for participant recruitment to this study. This will allow recruitment of a sample of men with a range of PiRADS scores (1-2 being low risk of prostate cancer; 3-5 being medium-high risk), ages (<70 years or 70+ years), geographical locations (urban or rural/countryside), and ethnic backgrounds (any white background or BME). In terms of GPs, a purposive sampling approach will allow recruitment of clinicians with a range of ages, genders, practice locations (urban or rural/countryside) and levels of experience.

6.3 Recruitment

6.3.1 Sample identification

6.3.1.1 Patient participants

Patient participants for this study will be recruited from two NHS Trusts; the Imperial College Healthcare NHS Trust in London and the Royal Devon & Exeter NHS Foundation Trust in Exeter. Men referred by their GP for suspected prostate cancer undergo an mpMRI prior to clinical review by a Urologist and potentially a prostate biopsy, depending on the mpMRI report (see study flow charts [pg 1-2]). Research nurses and/or fellows working within the clinic will identify potentially eligible men and contact them within days of undergoing an mpMRI to discuss this study and offer the men a Patient Information Leaflet (PIL – See 11.1.2). The PI and staff at the study sites will regularly communicate about potential recruits to ensure a range of age, ethnicity and geographical backgrounds are present in the included participants. Follow-up contact will be made by the research nurse/fellow if the man does not contact the lead researcher to check whether they wish to participate in the study or not.

Both NHS Trusts involved with this study have reviewed and approved this protocol, and they have expressed confidence that recruitment targets will be met. However, in the event that insufficient men are recruited for participation in this study 12 months after commencing recruitment, a further NHS Trust providing urology services that includes mpMRI for possible prostate cancer will be approached to aid recruitment.

Reasonable travel costs for patient participants to attend any face to face interview will be reimbursed, and participants will be offered a £20 gift voucher in recognition of their participation in the study.

6.3.1.2 GP participants

GP participants will be recruited through two National Institute for Health Research (NIHR) Clinical Research Networks (CRNs); North West London CRN and the South-West Peninsula CRN (see study flow chart [pg 3]). The CRNs will identify local practices from which to recruit eligible GPs to participate in this study, favouring Research Site Initiative (RSI) practices as these practices have an ongoing commitment to research and may have allocated research clinician time. In practices that do not have funded research clinician time, the CRN may provide support for participation in the study. Eligible GPs will be identified by the CRN and the practices, and they will regularly communicate with the PI to determine which GPs to approach for participation. GPs chosen for invitation into the study will be given a PIL (See 11.1.3) to consider participating in the study, and follow-up contact will be made by the CRN to confirm participation.

The NIHR CRN in South-West Peninsula and North West London have reviewed and approved this protocol, and they have expressed confidence in meeting recruitment targets. National adoption of this study within the NIHR CRN portfolio will allow the possibility of recruitment from other CRNs if there is any difficulty recruiting GPs in these two regions.

GP practices will be reimbursed £44.10 per 30 minute interview for the GP's time to participate.

6.3.2 Consent

For peer review only

For peer review only - http://bmjopen.bmj.com/site/about/guidelines.xhtml

All participants contacted for participation in this study will be given a PIL after being contacted as a potentially eligible participant. After reading the PIL, if the participant is willing to participate they will be contacted by the lead researcher to arrange an interview.

Consent will be taken at the start of the interview. The purpose of the study and the interview will be explained in conjunction with the information presented in the PIL. The lead researcher will assess the patient's ability to consent for participation. The participant will then be presented with a consent form (See 10.1.2 and 10.1.3) to complete if they are still willing to participate in the study.

Conducting patient interviews in their own home may result in other parties being present during the interview, such as the patient's spouse. If another person is present, the patient participant will be asked if they are happy to initially be interviewed in private. If the patient participant wishes for another person to be present, the additional person will be consented for participation and asked to complete a consent form before they join the interview.

6.3.3 Completing recruitment

Recruitment of patients and GPs will continue alongside analysis of interviews conducted to date, until no new themes or ideas emerge from the data.

7 ETHICAL AND REGULATORY CONSIDERATIONS

7.1 Data protection

All data will be collected, stored and processed in accordance with the General Data Protection Regulations 2018 and the Data Protection Act 2018. Informed consent will be obtained from all participants for all aspects of the study. Permission for the collection, storage and use of patient identifiable data (PID) in the study will be provided by consenting participants.

Participants will be free to withdraw consent for participation for any reason and at any time. Where consent is withdrawn, all participant identifiable information held by the research team will be destroyed, and the participant will not receive any further contact regarding the study.

7.2 Data anonymisation

All collected data will be fully anonymised before transfer to professional transcription services. Direct quotations from interview may be used in presenting the study results, however interviewees will not be identifiable in any way in any quotations used.

7.3 Data Storage

Encrypted voice recorders will be used for the interviews. Audio data will be downloaded and kept on secure servers at the University of Exeter until fully anonymised transcripts are created. Any audio files sent to professional transcription services will be anonymously labelled with a unique code, and encrypted for transfer. All physical data such as consent forms and transcripts of interviews will be stored within locked filing cabinets, within a locked office within the University of Exeter Medical School. The keys will be stored separately and only be accessed by the local research team.

All personal data will be securely destroyed within 12 months after the end of the study.

7.4 Assessment and management of risk

This study may be viewed as potentially sensitive in that it explores experiences of personal and intimate symptoms and body systems, in the context of a potentially serious diagnosis (cancer). Although there is a potential ethical problem with interviewing patients around the time of a cancer diagnosis, the proposed recruitment approaches have been successfully used in previous UK early

For peer review only - http://bmjopen.bmj.com/site/about/guidelines.xhtml

diagnosis of cancer studies, led by my supervisor, Dr Fiona Walter. These studies include questionnaire and interview studies people in patients with symptoms suggestive of lung, colorectal and pancreatic cancer (the SYMPTOM study)(31,32), an interview study set among people recently diagnosed with melanoma(33), and the on-going ECASS study (weekly case note reviews and subsequent GP and patient interviews in people with gastro-oesophageal symptoms).

Nevertheless, there are a number of strategies in place to minimise any possible distress that may arise in patients (and informal carers) during the study:

Interviews will be conducted by SM, who has been trained in qualitative interviewing. Combining with his experience as a GP, he would have had experience of communicating sensitively, empathetically and sympathetically with patients when breaking bad news and around sensitive topics, including cancer. Interviews will be conducted at a time and location convenient to participants, and in an unhurried manner, with participants being free to stop the discussion at any time. Appropriate leave-taking will also be practised to ensure that participants are not left in a distressed state following the interview.

It would be reasonable to assume that patients being recruited for interview are aware that they are being investigated with mpMRI due to the suspicion of them having prostate cancer. Local NHS protocols for referring men on the two-week wait urgent cancer referral pathway include the need for GPs to inform the patient they are being referred for a suspected diagnosis of cancer. However, the interviews for this study will not be conducted with that assumption. Early in the interview the patient's understanding for the reason for their referral for mpMRI and other investigations will be explored, to ensure that they patient's underlying knowledge and assumptions are clear to the interviewer.

SM is a practicing GP, and there is the possibility that patients will want to seek advice or an opinion form SM about their healthcare relating to the issue of possible prostate cancer or another health issue. SM will be clear that he is conducting the interview in his capacity as a researcher, and will refer any questions about the patient's healthcare back to their own GP.

A procedure is followed in the event of a participant becoming distressed, which includes the interviewer expressing concern as early as possible about the participant's comfort, offering them tissues or water, and asking whether they would like to take a break or discontinue the interview. Support mechanisms are in place (see Box 1) and the interviewer will inform the participant of these. Participants will also be reminded that they can withdraw from the study or complete the interview at another time, and that this is entirely their decision.

SM will meet regularly during data collection with his supervisors, FW and AF, who have extensive clinical and research experience with patients about cancer symptoms and pathways to diagnosis. This will ensure that should any issues arise they can be dealt with expediently, and learning applied for subsequent interviews.

For peer review only

For peer review only - http://bmjopen.bmj.com/site/about/guidelines.xhtml

Box 1: Guidelines for researchers conducting sensitive interviews

1. All interviews are to be conducted from the outset with the greatest of sensitivity and concern for the respondent's welfare.

2. The interviewer should be observant of the respondent's level of comfort and watch for early signs of distress, such as breaks in speech or nervous body movements. Should early signs appear the interviewer should express concern about the respondent's comfort and ask questions such as (gauged by respondent's signs): would they like a glass of water; if a break is needed; if they would prefer to complete the interview another time; or if they would prefer to discontinue.

3. If overt distress occurs, the interview should cease immediately and actions taken to support the respondent, such as offering tissues or water; seeking immediate additional support from a more familiar person, if available; and staying with the respondent until they are ready to express their wishes on the options available to them.

4. If it becomes apparent that a distressed respondent has particular areas of need concerning their illness or circumstances, where appropriate, the interviewer should offer to assist the respondent to make contact with a relevant support, such as their GP surgery.

5. Concerning the interview, the options eventually offered to a distressed respondent should be (in order): withdraw from the study; or complete the interview another time. The interview should only be continued after a break if the respondent requests this as their unprompted decision.

7.5 Adverse Events

An adverse event will be defined as 'an event that arises directly from participation in the research', including complications that occur in the course of investigation. All adverse events will be discussed with the Supervisors, both of whom (especially FW) have had extensive experience in carrying out similar studies involving early diagnosis of cancer. Appropriate subsequent course of action will be taken after discussion with the full research team, and the Sponsor will be notified.

Appropriate safety procedures will be followed by the researcher(s) when interviewing participants. Should any disclosures requiring action be made, the researchers will have access to the support of the full research team.

7.6 Insurance

Arrangements have been made through the University of Exeter for insurance and/or indemnity to meet the potential legal liability of the sponsor for harm to participants arising from the management, design or conduct of the research.

NHS indemnity scheme will apply for insurance and/or indemnity to meet the potential legal liability of the investigator arising from harm to participants in the conduct of the research at NHS sites.

There are no arrangements in place for payment of compensation in the event of harm to the research participants where no legal liability arises.

7.7 Research Ethics Committee (REC) and other Regulatory review & reports

The researcher will seek NHS research governance and compliance approval, and NHS ethical review, through the HRA approvals process via the Integrated Research Application System (IRAS).

For peer review only - http://bmjopen.bmj.com/site/about/guidelines.xhtml

The researcher will ensure that the protocol and all supporting participant-facing documentation receive HRA approval. Following review, research will only take place once appropriate HRA and Sponsor approvals are in place and confirmation of capacity and capability received from each local NHS site.

A copy of the approved study documents will be submitted to the R&D Office or practice manager at each local site prior to the commencement of any study procedures.

The Chief Investigator is responsible for keeping all correspondence with the REC, producing annual reports, notifying the REC at the end of the study and producing final reports.

7.8 Protocol compliance

Any accidental protocol deviations will be adequately documented on the relevant forms and reported to the Researcher and Sponsor. All deviations from the protocol which are found to frequently recur will require immediate action and could potentially be classified as a serious breach.

Notification of Serious Breaches to GCP and/or the protocol: A 'serious breach' is a breach which is likely to affect to a significant degree;

The safety or physical or mental integrity of the subjects of the study; or

The scientific value of the study

The Sponsor will be notified immediately of any case where the above definition applies during the study.

7.9 Amendments

For any amendment to the study, the Chief Investigator or designee, in agreement with the sponsor will submit information to the appropriate body in order for them to issue approval for the amendment. The Chief Investigator or designee will work with sites (R&D departments at NHS sites as well as the study delivery team) so they can put the necessary arrangements in place to implement the amendment to confirm their support for the study as amended.

If the sponsor wishes to make a substantial amendment to the REC application or the supporting documents, the sponsor must submit a valid notice of amendment to the REC for consideration. The REC will provide a response regarding the amendment within 35 days of receipt of the notice. It is the sponsor's responsibility to decide whether an amendment is substantial or non-substantial for the purposes of submission to the REC.

Amendments will also be notified to the HRA national coordinating function of England where the lead NHS R&D office is based and communicated to the participating organisations (R&D office and local research team) and departments of participating sites to assess whether the amendment affects the NHS permission for that site.

Amendments considered to be non-substantial for the purposes of REC will still be notified to the HRA for approval after confirmation from the Sponsor.

7.10 Peer review

The PhD proposal, which this study forms a key part of, has been subject to peer review by two senior researchers within the CanTest programme. Both reviewers are external to the University of Exeter and are not involved with this study in any way. They are both very experienced and widely published primary care cancer researchers. Feedback from the peer review was utilised to refine and enchance the development of this study.

7.11 Patient & Public Involvement

For peer review only - http://bmjopen.bmj.com/site/about/guidelines.xhtml

The NIHR Clinical Research Facility Peninsula Research Bank steering committee includes a panel of lay members. 14 members of the panel, including two members who had previously been service users investigated for possible prostate cancer, provided input into the acceptability and design of this study. The lay members assisted in the writing of the lay summary, and they reviewed drafts of the consent form, participant information leaflets, and interview guides. Feedback received from these service users and members of the public has been integrated into this protocol and associated documents.

Separately, a PPI group of men (with no history of prostate cancer) is currently being assembled to help steer the PhD that this study forms a part of. This PPI group will be involved with the analysis of results and the dissemination of findings from this study.

7.12 Access to the final study dataset

Access to the full dataset will be limited to the researcher and the supervisors for this study. In line with Cancer Research UK (CRUK) policy, fully anonymised interview transcripts will potentially be made available to researchers for analysis in future related studies, subject to consent obtained from participants.

Any research nurses or fellows involved with recruitment will not have any access to the data collected.

8 DISSEMINIATION POLICY

8.1 Dissemination policy

The data arising from the study will be owned by the University of Exeter.

On completion of the study, data will be analysed and synthesised into a chapter for the PhD of the researcher, registered at the University of Exeter. Access to the full study report, including the protocol, will be made through the Open Research Exeter (ORE) online portal, hosted by the University of Exeter, after the thesis has been accepted by The University.

CRUK are the major funders of this study, through a Catalyst Award ('CanTest'). CanTest funds the researcher's salary, training, PhD fees and research costs. CRUK will be acknowledged in any publications associated with this study.

All participants will receive an abridged study report, outlining the major findings of the study.

8.2 Authorship eligibility guidelines and any intended use of professional writers

Authorship for the final study report and any publications associated with this study will be agreed in accordance with the International Committee of Medical Journal Editors guidance.

9 REFERENCES

1. Lijmer JG, Leeflang M, Bossuyt PMM. Proposals for a phased evaluation of medical tests. Med Decis Mak. 2009;29(5):13–21.
2. Fryback DG, Thornbury JR, Fryback DG, Thornbury JR. Medical Decision Making The Efficacy of Diagnostic Imaging. Med Decis Mak. 1991;11:88–94.
3. Horvath AR, Lord SJ, StJohn A, Sandberg S, Cobbaert CM, Lorenz S, et al. From biomarkers to medical tests: The changing landscape of test evaluation. Clin Chim Acta [Internet]. 2014;427:49–57. Available from: http://dx.doi.org/10.1016/j.cca.2013.09.018
4. Lin JS, Thompson M, Goddard KAB, Piper MA, Heneghan C, Whitlock EP. Evaluating genomic

For peer review only - http://bmjopen.bmj.com/site/about/guidelines.xhtml

tests from bench to bedside: A practical framework. BMC Med Inform Decis Mak [Internet]. 2012;12(1):1. Available from: BMC Medical Informatics and Decision Making

5. Thompson M, Weigl B, Fitzpatrick A, Ide N. More Than Just Accuracy: A Novel Method to Incorporate Multiple Test Attributes in Evaluating Diagnostic Tests Including Point of Care Tests. IEEE J Transl Eng Heal Med. 2016;4(January).

6. National Collaborating Centre for Cancer. Suspected cancer [Internet]. NICE. London; 2015. Available from: http://journals.rcni.com/doi/10.7748/ns.29.47.17.s20

7. Mottet N, Bellmunt J, Briers E, Bolla M, Bourke L, Cornford P, et al. EAU - ESTRO - ESUR - SIOG Guidelines on Prostate Cancer [Internet]. European Association of Urology; 2017. Available from: https://uroweb.org/wp-content/uploads/09-Prostate-Cancer_2017_web.pdf

8. Loeb S, Vellekoop A, Ahmed HU, Catto J, Emberton M, Nam R, et al. Systematic review of complications of prostate biopsy. Eur Urol [Internet]. 2013 Dec 1;64(6):876–92. Available from: http://dx.doi.org/10.1016/j.eururo.2013.05.049

9. Hassanzadeh E, Glazer DI, Dunne RM, Fennessy FM, Harisinghani MG, Tempany CM. Prostate imaging reporting and data system version 2 (PI-RADS v2): a pictorial review. Abdom Radiol (New York) [Internet]. 2017;42(1):278–89. Available from: http://www.ncbi.nlm.nih.gov/pubmed/27522352%0Ahttp://www.pubmedcentral.nih.gov/articlerender.fcgi?artid=PMC5247306%0Ahttp://www.ncbi.nlm.nih.gov/pubmed/27522352%5Cnhttp://www.pubmedcentral.nih.gov/articlerender.fcgi?artid=PMC5247306

10. Ahmed HU, Bosaily AE-S, Brown LC, Gabe R, Kaplan R, Parmar MK, et al. Diagnostic accuracy of multi-parametric MRI and TRUS biopsy in prostate cancer (PROMIS): a paired validating confirmatory study. Lancet [Internet]. 2017 Jan 19;380:1–8. Available from: http://dx.doi.org/10.1016/S0140-6736(16)32401-1

11. Kasivisvanathan V, Rannikko AS, Borghi M, Panebianco V, Mynderse LA, Vaarala MH, et al. MRI-Targeted or Standard Biopsy for Prostate-Cancer Diagnosis. N Engl J Med [Internet]. 2018; Available from: http://www.nejm.org/doi/10.1056/NEJMoa1801993

12. Bray F, Ferlay J, Soerjomataram I, Siegel R, Torre L, Jemal A. Global cancer statistics 2018: GLOBOCAN estimates of incidence and mortality worldwide for 36 cancers in 185 countries. CA A J Clin. 2018;00(00):1–31.

13. Office for National Statistics. Cancer registration statistics, England: first release, 2015. Statistical Bulletin. 2017.

14. Sandhu GS, Andriole GL. Overdiagnosis of prostate cancer. J Natl Cancer Inst - Monogr [Internet]. 2012 Dec 27;2012(45):146–51. Available from: http://jncimono.oxfordjournals.org/cgi/doi/10.1093/jncimonographs/lgs031

15. Boesen L, Norgaard N, Logager V, Balslev I, Thestrup K, Bisbjerg R, et al. Assessment of the Diagnostic Accuracy of Biparametric Magnetic Resonance Imaging for Prostate Cancer in Biopsy-Naive Men: The Biparametric MRI for Detection of Prostate Cancer (BIDOC) study. JAMA Netw Open. 2018;1(2).

16. Otti VC, Miller C, Powell RJ, Thomas RM, McGrath JS. The diagnostic accuracy of multiparametric MRI prior to biopsy in the detection of prostate cancer. BJU Int [Internet]. 2018; Available from: http://www.ncbi.nlm.nih.gov/pubmed/29804315%0Ahttp://doi.wiley.com/10.1111/bju.14420

17. NHS England. Implementing a timed prostate cancer diagnostic pathway. NHS; 2018.

18. Peters JL, Thompson AC, Mcnicholas TA, Hines JEW, Hanbury DC, Boustead GB. Increased patient satisfaction from transrectal ultrasonography and biopsy under sedation. BJU Int. 2001;87(9):827–30.

19. Makinen T, Auvinen A, Hakama M, Stenman UH, Tammela TLJ. Acceptability and

For peer review only

For peer review only - http://bmjopen.bmj.com/site/about/guidelines.xhtml

complications of prostate biopsy in population-based PSA screening versus routine clinical practice: A prospective, controlled study. Urology. 2002;60(5):846–50.

20. Clements R, Aideyan OU, Griffiths GJ, Peeling WB. Side effects and patient acceptability of transrectal biopsy of the prostate. Clin Radiol. 1993;47(2):125–6.

21. Egbers N, Schwenke C, Maxeiner A, Teichgräber U, Franie T. MRI-guided core needle biopsy of the prostate: Acceptance and side effects. Diagnostic Interv Radiol. 2015;21(3):215–21.

22. Ullrich T, Schimmöller L, Oymanns M, Blondin D, Dietzel F, Kirchner J, et al. Current Utilization and Acceptance of Multiparametric MRI in the Diagnosis of Prostate Cancer. A Regional Survey. RöFo - Fortschritte auf dem Gebiet der Röntgenstrahlen und der Bildgeb Verfahren [Internet]. 2017;419–26. Available from: http://www.thieme-connect.de/DOI/DOI?10.1055/s-0043-118128

23. Sekhon M, Cartwright M, Francis JJ. Acceptability of health care interventions: A theoretical framework and proposed research agenda. Br J Health Psychol. 2018;1–13.

24. Sekhon M, Cartwright M, Francis JJ. Acceptability of healthcare interventions: An overview of reviews and development of a theoretical framework. BMC Health Serv Res [Internet]. 2017;17(1):1–13. Available from: http://dx.doi.org/10.1186/s12913-017-2031-8

25. Strauss A, Corbin J. Basics of qualitative research. Sage; 1990.

26. Silverman D. Doing qualitative research. Sage; 2013.

27. Ahmed HU, El-Shater Bosaily A, Brown LC, Gabe R, Kaplan R, Parmar MK, et al. Diagnostic accuracy of multi-parametric MRI and TRUS biopsy in prostate cancer (PROMIS): a paired validating confirmatory study. Lancet [Internet]. 2017;389(10071):815–22. Available from: http://dx.doi.org/10.1016/S0140-6736(16)32401-1

28. Panebianco V, Barchetti G, Simone G, Del Monte M, Ciardi A, Grompone MD, et al. Negative Multiparametric Magnetic Resonance Imaging for Prostate Cancer: What's Next? Eur Urol [Internet]. 2018;74(1):48–54. Available from: https://doi.org/10.1016/j.eururo.2018.03.007

29. Ritchie J, Spencer EA. Qualitative data analysis for applied policy research. In: Analyzing Qualitative Data. 1st ed. London: Routledge; 1994. p. 173–94.

30. Gale NK, Heath G, Cameron E, Rashid S, Redwood S. Using the framework method for the analysis of qualitative data in multi-disciplinary health research. BMC Med Res Methodol [Internet]. 2013;13(1):1. Available from: BMC Medical Research Methodology

31. Birt L, Hall N, Emery J, Banks J, Mills K, Johnson M, et al. Responding to symptoms suggestive of lung cancer: A qualitative interview study. BMJ Open Respir Res. 2014;1(1).

32. Hall N, Birt L, Banks J, Emery J, Mills K, Johnson M, et al. Symptom appraisal and healthcare-seeking for symptoms suggestive of colorectal cancer: A qualitative study. BMJ Open. 2015;5(10).

33. Walter FM, Birt L, Cavers D, Scott S, Emery J, Burrows N, et al. "This isn't what mine looked like": A qualitative study of symptom appraisal and help seeking in people recently diagnosed with melanoma. BMJ Open. 2014;4(7):1–12.

For peer review only

10. APPENDICIES

10.1 Amendment History

Amendment No.	Protocol version no.	Date issued	Author(s) of changes	Details of changes made

For peer review only

Consolidated criteria for reporting qualitative studies (COREQ): 32-item checklist

Developed from:
Tong A, Sainsbury P, Craig J. Consolidated criteria for reporting qualitative research (COREQ): a 32-item checklist for interviews and focus groups. *International Journal for Quality in Health Care*. 2007. Volume 19, Number 6: pp. 349 – 357

**No. Item**	**Guide questions/description**	**Reported on Page #**
**Domain 1: Research team and reflexivity**		
Personal Characteristics		
1. Inter viewer/facilitator	Which author/s conducted the interview or focus group?	8
2. Credentials	What were the researcher's credentials? E.g. PhD, MD	1-2
3. Occupation	What was their occupation at the time of the study?	8
4. Gender	Was the researcher male or female?	8
5. Experience and training	What experience or training did the researcher have?	8
Relationship with participants		
6. Relationship established	Was a relationship established prior to study commencement?	8
7. Participant knowledge of the interviewer	What did the participants know about the researcher? e.g. personal goals, reasons for doing the research	19
8. Interviewer characteristics	What characteristics were reported about the inter viewer/facilitator? e.g. Bias, assumptions, reasons and interests in the research topic	19
**Domain 2: study design**		
Theoretical framework		
9. Methodological orientation and Theory	What methodological orientation was stated to underpin the study? e.g. grounded theory, discourse analysis, ethnography, phenomenology, content analysis	7
Participant selection		
10. Sampling	How were participants selected? e.g. purposive, convenience, consecutive, snowball	7
11. Method of approach	How were participants approached? e.g. face-to-face, telephone, mail, email	7-8
12. Sample size	How many participants were in the study?	11
13. Non-participation	How many people refused to participate or dropped out? Reasons?	11

For peer review only

Setting		
14. Setting of data collection	Where was the data collected? e.g. home, clinic, workplace	8
15. Presence of non-participants	Was anyone else present besides the participants and researchers?	11
16. Description of sample	What are the important characteristics of the sample? e.g. demographic data, date	11
Data collection		
17. Interview guide	Were questions, prompts, guides provided by the authors? Was it pilot tested?	8
18. Repeat interviews	Were repeat inter views carried out? If yes, how many?	9
19. Audio/visual recording	Did the research use audio or visual recording to collect the data?	9
20. Field notes	Were field notes made during and/or after the inter view or focus group?	9
21. Duration	What was the duration of the inter views or focus group?	9
22. Data saturation	Was data saturation discussed?	9
23. Transcripts returned	Were transcripts returned to participants for comment and/or correction?	9
**Domain 3: analysis and findings**		
Data analysis		
24. Number of data coders	How many data coders coded the data?	9
25. Description of the coding tree	Did authors provide a description of the coding tree?	9
26. Derivation of themes	Were themes identified in advance or derived from the data?	9
27. Software	What software, if applicable, was used to manage the data?	NVivo
28. Participant checking	Did participants provide feedback on the findings?	9
Reporting		
29. Quotations presented	Were participant quotations presented to illustrate the themes/findings? Was each quotation identified? e.g. participant number	12-18
30. Data and findings consistent	Was there consistency between the data presented and the findings?	12-18
31. Clarity of major themes	Were major themes clearly presented in the findings?	12-18
32. Clarity of minor themes	Is there a description of diverse cases or discussion of minor themes?	12-18

For peer review only

BMJ Open

Experiences of ‘traditional’ and ‘one-stop’ MRI-based prostate cancer diagnostic pathways in England: a qualitative study with patients and GPs

Journal:	*BMJ Open*
Manuscript ID	bmjopen-2021-054045.R3
Article Type:	Original research
Date Submitted by the Author:	05-Jul-2022
Complete List of Authors:	Merriel, Samuel; University of Exeter, Institute of Health Research<br>Archer, Stephanie; University of Cambridge School of Clinical Medicine, Public Health and Primary Care; University of Cambridge<br>Forster, Alice; UCL, Behavioural Science and Health<br>Eldred-Evans, David; Imperial College London, Imperial Prostate<br>McGrath, John; Royal Devon and Exeter NHS Foundation Trust, Urology<br>Ahmed, Hashim; Imperial College London, Imperial Prostate<br>Hamilton, Willie; University of Exeter, Peninsula College of Medicine and Dentistry<br>Walter, Fiona; Queen Mary University of London, Wolfson Institute of Public Health; University of Cambridge, Department of Public Health and Primary Care
**Primary Subject Heading**:	Qualitative research
Secondary Subject Heading:	Urology, General practice / Family practice
Keywords:	PRIMARY CARE, QUALITATIVE RESEARCH, Prostate disease < UROLOGY, Urological tumours < ONCOLOGY, Magnetic resonance imaging < RADIOLOGY & IMAGING

SCHOLARONE™
Manuscripts

I, the Submitting Author has the right to grant and does grant on behalf of all authors of the Work (as defined in the below author licence), an exclusive licence and/or a non-exclusive licence for contributions from authors who are: i) UK Crown employees; ii) where BMJ has agreed a CC-BY licence shall apply, and/or iii) in accordance with the terms applicable for US Federal Government officers or employees acting as part of their official duties; on a worldwide, perpetual, irrevocable, royalty-free basis to BMJ Publishing Group Ltd ("BMJ") its licensees and where the relevant Journal is co-owned by BMJ to the co-owners of the Journal, to publish the Work in this journal and any other BMJ products and to exploit all rights, as set out in our licence.

The Submitting Author accepts and understands that any supply made under these terms is made by BMJ to the Submitting Author unless you are acting as an employee on behalf of your employer or a postgraduate student of an affiliated institution which is paying any applicable article publishing charge ("APC") for Open Access articles. Where the Submitting Author wishes to make the Work available on an Open Access basis (and intends to pay the relevant APC), the terms of reuse of such Open Access shall be governed by a Creative Commons licence – details of these licences and which Creative Commons licence will apply to this Work are set out in our licence referred to above.

Other than as permitted in any relevant BMJ Author's Self Archiving Policies, I confirm this Work has not been accepted for publication elsewhere, is not being considered for publication elsewhere and does not duplicate material already published. I confirm all authors consent to publication of this Work and authorise the granting of this licence.

For peer review only

Experiences of 'traditional' and 'one-stop' MRI-based prostate cancer diagnostic pathways in England: a qualitative study with patients and GPs

For peer review only

Dr Samuel W D Merriel MSc, University of Exeter Medical School, University of Exeter, UK

s.w.d.merriel@exeter.ac.uk

Dr Stephanie Archer PhD, Department of Public Health and Primary Care / Department of Psychology, University of Cambridge, UK

saa71@medschl.cam.ac.uk

Dr Alice Forster PhD, Institute of Epidemiology & Health, University College London, UK

alice.forster@ucl.ac.uk

Dr David Eldred-Evans PhD, Imperial Prostate, Department of Surgery & Cancer, Imperial College London, UK; Imperial Urology, Charing Cross Hospital, Imperial College Healthcare NHS Trust, London, UK

d.eldred-evans@imperial.ac.uk

Mr John McGrath FRCS, Department of Urological Surgery, Royal Devon & Exeter NHS Foundation Trust, UK

john.mcgrath4@nhs.net

Prof Hashim U. Ahmed FRCS, Imperial Prostate, Department of Surgery & Cancer, Imperial College London, UK; Imperial Urology, Charing Cross Hospital, Imperial College Healthcare NHS Trust, London, UK

Hashim.ahmed@imperial.ac.uk

Prof Willie Hamilton MD, University of Exeter Medical School, University of Exeter, UK

w.hamilton@exeter.ac.uk

Dr Fiona M Walter MD, Wolfson Institute of Population Health, Queen Mary University of London, UK, & The Primary Care Unit, Department of Public Health and Primary Care, University of Cambridge, UK fiona.walter@qmul.ac.uk

Corresponding author

Dr Samuel W D Merriel

1.18 College House, St Luke's campus, University of Exeter

Heavitree Road, Exeter, UK EX1 2LU

+441392 726002

s.w.d.merriel@exeter.ac.uk

Keywords: Prostate cancer, diagnostic pathway, primary care

For peer review only

Abstract

Objectives

This study aimed to understand and explore patient and GP experiences of 'traditional' and 'one-stop' prostate cancer diagnostic pathways in England.

Design

Qualitative study using semi-structured interviews, analysed using inductive thematic analysis

Setting

Patients were recruited from National Health Service (NHS) Trusts in London and in Devon; GPs were recruited via National Institute for Health Research (NIHR) Clinical Research Networks. Interviews were conducted in person or via telephone.

Participants

Patients who had undergone a magnetic resonance imaging (MRI) scan of the prostate as part of their diagnostic work-up for possible prostate cancer, and GPs who had referred at least one patient for possible prostate cancer in the preceding 12 months.

Results

22 patients (aged 47 – 80 years) and 10 GPs (6 female, aged 38 – 58 years) were interviewed. Patients described three key themes: cancer beliefs in relation to patient's attitudes towards prostate cancer; communication with their GP and specialist having a significant impact on experience of the pathway; and pathway experience being influenced by appointment and test burden. GP interview themes included: the challenges of dealing with imperfect information in the current pathway; managing uncertainty in identifying patients with possible prostate cancer, and sharing this uncertainty with them, and other social, cultural and personal contextual influences.

Conclusions

Patients and GPs reported a range of experiences and views of the current prostate cancer diagnostic pathways in England. Patients valued 'one-stop' pathways integrating prostate MRI and diagnostic consultations with specialists over the more traditional approach of several hospital appointments. GPs remain uncertain how best to identify patients needing referral for urgent prostate cancer testing due to the lack of accurate triage and risk assessment strategies.

For peer review only - http://bmjopen.bmj.com/site/about/guidelines.xhtml

Article summary

Strengths and limitations

- Patient experiences of two very different prostate cancer diagnostic pathways compared and contrasted
- Patient sample feature a broad range of ages, geographical regions, and cancer investigation journeys to generate rich data
- First study to explore GP experience and understanding of new prostate cancer diagnostic pathways incorporating magnetic resonance imaging (MRI)
- Limited knowledge of prostate MRI curtailed interviews with some GP participants

For peer review only

Introduction

Patient experience of healthcare has developed as an important marker of quality of care in recent decades. However, measuring and understanding patient experience of diagnostic pathways and services is underexplored and poorly prioritised compared to other aspects of healthcare such as access or treatments[1]. Assessment of the impact of variations in pathway design between health services may also identify elements associated with better patient experience, such as quicker access to testing, that could be implemented more widely and elements associated with adverse experience, such as high appointment burden, that can be avoided.

The National Health Service (NHS) in England has a Two Week Wait (2WW) urgent cancer referral pathway system, where any patient with symptoms or signs of a potential undiagnosed cancer referred by their General Practitioner (GP) should have a specialist review for further investigation within two weeks[2]. Cancer diagnostic pathways are prioritised for urgent access to specialist assessment and diagnostic tests as early-stage diagnosis is associated with increased survival[3]. Not only do shorter diagnostic intervals improve outcomes for patients, but patients also report better experiences of care[4]. Significant variation in cancer diagnostic pathways between NHS Trusts and regions in England exists, most markedly for prostate cancer[5]. Identifying patients for 2WW prostate cancer referral in primary care is also challenging for GPs owing to limitations of existing tests, including prostate specific antigen (PSA), which can impact on doctor-patient communication and patient experience of the early stages of the prostate cancer diagnostic pathway[6,7].

National Institute for Health and Care Excellence (NICE) guidance for diagnosing prostate cancer in England was updated in 2019 to recommend pre-biopsy magnetic resonance imaging (MRI) for men suspected of having prostate cancer[8]. In response, Cancer Alliances and Hospital Trusts in the NHS have updated local prostate cancer diagnostic pathways, with significant variation in the implementation of MRI[9]. Despite the potential benefits prostate MRI brings in terms of more accurate prostate cancer diagnosis[10], adding further testing into the prostate cancer diagnostic pathway could lengthen the diagnostic interval, adversely impacting patient experience.

For peer review only

Experiences of the prostate cancer diagnostic pathway for patients and GPs since the advent of prostate MRI is unknown. The aim of this study was to elicit the experience of patients and GPs following two prostate cancer diagnostic pathways that incorporate pre-biopsy MRI in different ways to inform optimal prostate cancer diagnostic pathway design. In the context of the Model of Pathways to Treatment, a key theoretical framework in cancer diagnostic pathways, this study focuses on the 'Help-seeking' and 'Diagnostic' intervals and explores the perspectives of both patient and clinician[11,12].

For peer review only

Methods

This qualitative study used semi-structured interviews to explore the experiences of patients referred from primary care with possible prostate cancer who had undergone an MRI, and GPs who have referred men with possible prostate cancer for further investigation. A constructivist approach was adopted to access the data and understand the experiences of patients and GPs[13] based on their individual experiences (past and present) and the socio-cultural context[14,15].

Participants

This study recruited participants from two populations through purposive sampling;

- Patients with possible prostate cancer who had undergone an MRI as part of their diagnostic workup.
- GPs who had referred at least one patient for investigation for possible prostate cancer within the preceding 12 months.

Patients who were undergoing MRI for active surveillance or watchful waiting for a previously diagnosed prostate cancer were not eligible, as the focus of this study was on the role of MRI in the diagnosis of prostate cancer rather than management.

Recruitment

Patients were recruited from two NHS Trusts in England: The Royal Devon & Exeter NHS Foundation Trust in Exeter and the Imperial College Healthcare NHS Trust in London. The Royal Devon & Exeter Hospital use separate outpatient appointments in the South West (SW) Prostate Cancer Diagnostic Pathway for a prostate MRI, consultant review, and prostate biopsy (if required), as shown in Figure 1. Imperial College employ the RAPID pathway, where patients undergo a prostate MRI scan, receive their MRI result, and potentially undergo a prostate biopsy on the same day at a single outpatient attendance (see Figure 2). These Trusts were selected as prostate MRI has been implemented in very different ways, creating the opportunity to explore and compare patient and clinician experiences in different clinical contexts. Research staff at the Trusts identified potentially eligible men and contacted them within days of undergoing an MRI to discuss this study and offer the men a Patient Information Leaflet (PIL). The lead investigator and local recruitment leads were in regular contact throughout recruitment to identify any under-

For peer review only - http://bmjopen.bmj.com/site/about/guidelines.xhtml

represented groups of men and focus recruitment where needed. Travel costs for patient participants to attend a face-to-face interview were reimbursed, and participants were also offered a gift voucher in recognition of contributing their time.

Figure 1

Figure 2

GPs were recruited through two National Institute for Health Research (NIHR) Clinical Research Networks (CRNs) in the same regions as the hospital sites: Northwest London CRN and the South-West Peninsula CRN. The CRNs promoted the study to local practices, and GPs expressed their interest to the CRNs. Eligibility and basic demographics were checked to assist with purposive sampling. GPs chosen for invitation into the study were given a PIL to review prior to the arrangement of an interview. GP practices were reimbursed for the GP's time to participate in the study.

A purposive sampling approach was used, in order to obtain a diverse group of participants with a wide range of geographical locations, ages, ethnicities, genders (GPs) and MRI results (patients).

Data collection

One-to-one interviews were conducted with all participants in this study between July and November 2019 by SM (a male GP with training in qualitative interviewing). Patient participants were either interviewed face-to-face in their own home or via telephone, while all GP participant interviews were conducted via telephone. The interviewer and participant were not known to each other before the study. Formal written consent was obtained from all participants, and patient's partners if present (n=2), prior to commencement of the interview. A semi-structured approach was followed, with separate interview topic guides for patient and GP interviews to support discussions (See supplementary file 1 and 2). The topic guide was developed iteratively with input from our patient/public partners; it was further refined during the first three interviews

For peer review only

to incorporate all aspects of the revised prostate cancer diagnostic pathway and was used flexibly to ensure that no key aspects of the diagnostic pathway experience were missed. An encrypted audio recording device was employed to record all interviews, and written notes were taken during and immediately following the interviews. Interview times ranged between 15-45 minutes each, and no repeat or follow-up interviews were undertaken. Interview recordings were transferred securely to an independent transcribing service, and transcribed verbatim.

Data analysis

An inductive thematic analysis was conducted to understand the experiences of participants[16], using the conceptual framework of the Model of Pathways to Treatment[11,12]. The researchers initially immersed themselves in the data through reading and re-reading individual transcripts and listening back to the audio recordings of the interviews. A selection of early interviews were coded, and this initial coding framework was reviewed and refined by SM, SA and FW. The remaining interview transcripts were coded by SM inductively from the entirety of the data. The codes were reviewed and arranged into themes through an iterative process, returning to the original data as needed. Patient and GP transcripts were analysed separately. Within and between themes, the experiences of participants following different diagnostic pathways were compared and contrasted. Recruitment ceased when no new themes emerged in analysis. Transcripts were imported into NVivo v12 to manage the data for the analysis. A study summary report was sent to all study participants after completion of data analysis.

Patient & Public (PPI) Involvement statement

Eight men were recruited via the People in Health West of England (PHWE) initiative to contribute to the research: these men had a range of ages, locations, ethnic backgrounds and experiences with prostate cancer. PPI group members reviewed the plain English summary and all patient participant documents and gave feedback prior to submission as part of the ethical approval application. PPI group members also gave input into the interview topic guides and the expected burden of involvement for participants. One of the anonymised patient interview transcripts was shared with the group at a meeting and discussed to explore themes emerging from the text.

For peer review only

Ethical approval

Ethics committee approval was received from the NHS HRA South-West Frenchay research ethics committee (REC reference 19/SW/0040).

COREQ reporting guidelines

This manuscript has been written in accordance with the consolidated criterion for reporting qualitative research (COREQ) checklist[17]. Further detail regarding the methods can be found in the study protocol (see Supplementary file 3).

For peer review only

Results

Participants

Twenty-two patients were interviewed between July and November 2019; two with their wives present and involved in the interview: participant ages ranged from 47 – 80 years. Ten GPs were interviewed: most were female (n = 6), with an age range of 38 – 58 years (See table 1). Five further potential (three patients and two GPs) participants were approached but declined to participate without giving a reason.

	**Patients (n = 22)**		**GPs (n = 10)**
**Age**		**Age**	
<65	8	31-40	3
65+	14	41-50	6
		50+	1
**Location**		**Location**	
London	10	London	4
Devon	12	Devon	6
**Ethnicity**		**Gender**	
White	19	Male	4
Non-white	3	Female	6
**PIRADS v2***		**Role**	
1-2	6	Partner	8
3-5	15	Salaried	2
Unknown	1		

Table 1 – Patient and GP demographics

* PIRADS – Prostate Imaging-Reporting And Data System v2 score of 1-2 suggest clinically significant prostate cancer is unlikely and biopsy not indicated. A PIRADS score of 3-5 indicates at least one suspicious area of the prostate that warrants biopsy.

Patient experiences of the prostate cancer diagnostic pathway

For peer review only

We identified three main themes with interlinking sub-themes (see figure 3): Cancer beliefs, Communication, and Pathway experience.

Figure 3

Cancer beliefs

The decision for patients to see their doctor about potential prostate problems was not undertaken in isolation (*Outside influences*). The experiences of family members and friends shaped the patients' expectations, and family members and partners often encouraged men to be tested:

> *"Obviously back then he [dad] was in his mid to late 60s. And I think I didn't really know about it until he'd gone for his MRI and got the results and everything, and then all of a sudden he sat me down and told me all about it." P20 (London, <65)*

Most patients' attitudes towards the possibility of a diagnosis of cancer (*Attitude to diagnosis*) were fairly relaxed. Many seemed philosophical about the prospect:

> *"it is what it is" P03 (Devon, <65)*

The reactions of patients who had a diagnosis of prostate cancer (*Reaction to findings*) were mixed, ranging from despondence to quick acceptance:

> *"Not fair. No, it's... it's not fair on... on anyone, not just me. It isn't fair on anyone." P01 (Devon, 65+)*

Communication

For peer review only - http://bmjopen.bmj.com/site/about/guidelines.xhtml

The absence of the use of the word cancer (‘*C word*’) was evident in interviews with many patients. Patients also reported a reluctance from clinicians to raise cancer specifically as a possibility during a consultation, even if they were referred for urgent tests to rule out a diagnosis of prostate cancer:

> *“And then this developed.” P01 (Devon, 65+)*
>
> *“For me, my... my dad had it roughly about eight, nine years... eight to ten years ago, I suppose. He had it.” P20 (London, <65)*
>
> *“The only thing that I found was you were given leaflets that mention a lot about cancer but no one actually really, sort of like said to me, you know, there’s a possibility that you could have cancer or you know, that you’re just being given leaflets and such, and no one really explained to you that there is a possibility.” P25 (Devon, <65)*

The mode of communication to the patient from clinicians (*personal contact*) appeared to directly affect their experiences of the pathway. Most London patients sat down with their consultant and reviewed their MRI results together, whereas many patients in Devon received their results via a letter:

> *“I think it was interesting to see this sort of slightly darker little, ti… little circular area that he thought might be cancerous and… and also explain that they would need to take some samples from another area which… which was more the normal colour of the whole gland for comparison.” P13 (London, 65+)*
>
> *“Most of the letters go to the GP and I just get a copy.” P23 (Devon, 65+)*

Communicating the meaning (*conveying significance*) of the results of the MRI and other tests performed was very important to help patients and their partners understand what the results mean for them as an individual:

> *“Yeah, so apparently, because this is mid-rank they said that if you just got the first circle, the first ones in, they probably wouldn’t have done anything about it and you could have*

For peer review only

had a lot of years where you just monitor that. But because P03 was mid-stage, they said we have to do something." P03's partner (Devon, <65)

Despite most of the patients having undergone a prostate MRI by the time of their interview, there were still limited understanding of the MRI results for some patients (*Gaps in understanding*). More patients from Devon reported these gaps, which often appeared to be a result of communication breakdown between the patient and the doctor:

"Umm… I think, all I know is those letters passed to and fro between the urologist and my GP, and I'm copied in on these things and there was some mention of an abnormality on the left hand side or somewhere or other on the prostate. That's all I know." P23 (Devon, 65+)

<u>Pathway experience</u>

Patients entered the pathway in different ways, with varied length of time and diagnostic work-up prior to urgent suspected cancer referral (*Mixed routes*). For patients in Devon, the prostate cancer pathway required a number of individual appointments, whereas most patients in London received their MRI results on the same day or soon after which was well received (*Appointments burden*):

"I had a PSA of, I think it was 4.03, which was fractionally above the four limit. Then they gave me two additional PSAs every three months, so I went back three months later did another PSA and then I think it was about 3.84. Then another one three months later was 4.08. So then I saw a urologist at Exeter and as a precaution they gave me an MRI and the MRI identified an area of concern if you like [inaudible]. Then I had a biopsy and what that identified was that the area of concern that the MRI identified, there was no cancer, but there was cancer in another area." P04 (Devon, 65+)

For peer review only

"so… the scan, you get the result within minutes, and even though I had to wait perhaps an hour before I actually saw the doctor but that's a lot less than three months." P05 (London, 65+)

Patient interviewees were generally positive about undergoing investigations for possible prostate cancer, including blood tests and MRI. Most, but not all, patients reported that undergoing an MRI of the prostate was not a significant undertaking (*Test acceptability*):

"I'd go for any scan, anything like that. Needles don't bother me, scans don't bother me." P21 (Devon, 65+)

GP Experiences of the prostate cancer diagnostic pathway

We identified three main themes: Imperfect information, Managing uncertainty, and Contextual influences (see figure 4).

Figure 4

Imperfect information

GPs spoke at length about the limitations of the current primary care diagnostic pathway for prostate cancer, and about having imperfect information on which to base their clinical decisions. A few GPs described a sense of inevitability about patients presenting with lower urinary tract symptoms at some point as they entered their later years (*Non-specific presentation*):

"It's a bit of a grey area so you're kind of waiting for patients to develop symptoms and come to see you" GP03 (Male, London, 31-40)

For peer review only

As described earlier, GPs experienced men refusing to have a prostate examination when prostate cancer is suspected (*Examination acceptance*). GPs reported different reasons for this, and perceived that patients may still be worried even if the prostate feels normal:

> *"I've had patients before who even will have got a high PSA decline, a rectal examination because they've previously had some, kind of, you know, traumatic experience or whatever." GP04 (Female, Devon, 41-50)*

GPs from both regions did not hold back in sharing their opinions about the PSA blood test, and its usefulness (or lack thereof) in helping them make clinical decisions about which men to refer for further testing for possible prostate cancer. PSA appeared to hold poor face validity with GPs, and they expressed a hesitance in ordering the test (*GP test limitations*):

> *"I think if there's one test you could un-invent, I think PSA would be that…" GP02 (Male, Devon, 31-40)*
> *"So it's [PSA] quite a pain in the neck actually, to be honest…" GP05 (Female, Devon, 41-50)*
> *"Well, I don't like doing the PSA levels I suppose is one thing to say." GP07 (Female, London, 31-40)*

GPs working in the NHS cannot currently order an MRI of the prostate; the request must come from a secondary or tertiary care clinician. London GPs were more likely to be positive about the concept of a prostate MRI:

> *"I think it will be a really useful idea" GP03 (Male, London, 31-40)*
> *"Well, it's great, but it's not available to me. It's not something I decide on." GP05 (Female, Devon, 41-50)*

Managing uncertainty

For peer review only - http://bmjopen.bmj.com/site/about/guidelines.xhtml

For peer review only

GPs made efforts to share their diagnostic dilemma with patients where possible and consulted guidelines and their local urology specialists in managing uncertainty in their decisions about which men to refer to secondary care. Prior to referral, GPs tried to make their patients understand the limitations of the current diagnostic pathway (*counselling patient*):

> *"But I always would tell patients that it's not 100% and that both my examinations, whether it's a digital rectal or a PSA, are not 100% and it can be raised even without having cancer." GP03 (Male, London, 31-40)*

Whilst most GPs reported feeling satisfied with their local urology service (see *health service & guidance* below), some Devon GPs reported inconsistencies in the advice and management plans for their patients that came back from hospital specialists (*seeking advice*):

> *"I mean, we try to follow the guidelines but, as I say, we find mystifying as to the variation in the urology advice that comes back in terms of who to follow and who not to…" GP04 (Female, Devon, 41-50)*

<u>Contextual influences</u>

A spectrum of broader influences had an effect on when patients chose to present to their GP with concerns about prostate cancer, and the consultation itself (*Gender, society & culture*). Some GPs noted a reticence of men to seek healthcare:

> *"I think men don't... it's such a sweeping statement but men don't like coming to the doctor" GP07 (Female, London, 31-40)*

Consistent with the patient interviews (*outside influences*), the GPs reported that it was often the wives and partners encouraging male patients to seek help and advice:

> *"…the majority of men I see who mention prostate cancer it's because their wives have asked them to come and they're worried." GP07 (Female, London 31-40)*

For peer review only

Cultural and ethnic norms relating to the patient and their partners also influenced the consultation and acceptance of prostate examination, which were more commonly reported by GPs working in London:

> *"And over here I notice there are some patients of south Indian descent where, it's [DRE] almost like a taboo really."* GP03 (Male, London, 31-40)

GPs in both regions were aware of the influence of news and media stories relating to prostate cancer that were encouraging patients with symptoms or concerns to see their GP and get tested:

> *"…there was a lot in the media recently with prostate and testicular cancer, actually which is a good thing, because we had a… I had suddenly quite a few men coming in requesting the blood test."* GP09 (Female, London, 41-50)

GPs felt that most patients were aware of prostate cancer and that tests were available for it. Awareness of MRI of the prostate was lower than for the PSA blood test (*Patient awareness*):

> *"Lots of people are aware of the PSA"* GP07 (Female, London, 31-40)
> *"I think a few of them might have said, "I've heard there's a new test around." I don't think anyone's come in and said, "I'd like to have that MRI test."* GP04 (Female, Devon, 41-50)

The decision-making of GPs was also affected by their own experiences in their personal and professional lives (*Personal & professional experience*). GPs demonstrated an awareness of how these experiences shaped their approach:

> *"…my dad has prostate cancer that was picked up with a raised PSA. And my stepfather has prostate cancer which was picked up by a raised PSA. Both completely asymptomatic. So I think that also affects how you... how you practice and you know, as clinicians we do*

For peer review only

take on our life experiences and we can't help but have that shape how... how we work." GP07 (Female, London, 31-40)

The health service context in which GPs practise was another significant influence on their approach to patients with possible prostate cancer (*Health services & guidelines*). They often rely on guidance from a number of sources, including national guidelines and local urology services:

"I think we've got some, you know, very good local colleagues who offer good pragmatic advice and are very approachable." GP02 (Male, Devon, 31-40)

For peer review only

Discussion

Principal findings

Patient and GP experiences of more traditional and 'one-stop' prostate cancer diagnostic pathways incorporating MRI showed some key similarities and differences. Communication was a key element in the experience of the prostate cancer diagnostic pathway for both patients and GPs. The communication between patients and healthcare teams significantly affected the patients' overall experience and their understanding of MRI results. GPs valued the ability to communicate with specialists to obtain pragmatic advice and guidance, particularly in the context of their hesitancy in relying on PSA test results. Family and personal experiences also shaped the awareness of both patients and GPs in relation to prostate cancer diagnosis.

Compared to patients attending a 'one-stop' clinic, patients following more traditional diagnostic pathways felt that longer waits for tests, more appointments to attend, and increased travel requirements all impacted on their pathway experience. GPs faced challenges in dealing with uncertainty and the perceived limitations of symptoms, examination and tests available to them for diagnosing prostate cancer with confidence. GP awareness, understanding and access relating to MRI was limited in both regions.

Relation to published literature

This is the first study the authors are aware of to explore experiences of the modern pre-biopsy MRI prostate cancer diagnostic pathway from the perspective of patients and GPs. Ruseckaite *et al* interviewed 10 GPs from metropolitan Melbourne and a regional part of Victoria, Australia in 2015 regarding their perceptions of prostate cancer care. In line with the findings of this study, most men were willing to have a PSA blood test, and some GPs had to grapple with inconsistent guidance from specialist bodies[18]. Evans *et al* assessed men's experience of PSA testing in primary care in Wales in 2003-4, and also found that social networks and media stories influenced patient demand for testing. In contrast to the views of GPs in this study, the men in the study by Evans *et al* felt decision-making about testing was doctor-centred rather than shared or patient-centred[19].

For peer review only

In contrast to the limited amount of published evidence on patient experience of cancer diagnostic pathways, there have been many more studies on patient perspectives of prostate cancer screening that identified some key themes consistent with the findings of this study[20]. James et al found 'social prompting' from family and friends to consult their doctor about prostate cancer testing is a prominent theme in prostate cancer screening studies, similar to the 'outside influences' sub-theme that came from this research. Interestingly patients in prostate cancer screening studies also describe the 'physiological and symptomatic obscurity' of prostate cancer, which the GPs in this study were acutely aware of.

'Communication' of the results of diagnostic testing and a new diagnosis of cancer was another key theme emerging from interviews in this study that has a wealth of published research, and quality of communication can impact on patient and clinician experiences of diagnostic pathways. A number of studies have found deficiencies in communication from clinicians to patients about prostate cancer diagnostic testing and the results of tests, similar to the experience of some patients interviewed[21,22]. Some patients had the opportunity to discuss test results and understand the implications of the findings when they had it, whilst others felt communication about their test results was largely bypassing them between the specialist and GP. Interventions for improving prostate cancer patient engagement and empowerment have previously been developed[23], which may have a role in improving patient experience of modern prostate cancer diagnostic pathways.

Strengths and weaknesses

This study recruited a diverse sample of patients undergoing prostate MRI in terms of age, geographical distribution and ethnicity. Recruitment of patients from a range of ethnic minority backgrounds in cancer research can be challenging[24], so identifying and interviewing these patients as part of the study was key. Participants were recruited from two regions with contrasting prostate cancer diagnostic pathway designs. This enabled identification of key similarities and differences in the experiences of patients and GPs engaging with 'one stop' and more traditional pathways to help inform pathway design that could improve patient experience.

For peer review only

The influence of the researcher on data collection and analysis is important to consider in qualitative research. Participants were aware that SM was a clinician performing the study as part of a Cancer Research UK funded PhD, and that may have given some level of respectability and authority to the interviewer and the study. Some patients and GPs reported that men were less comfortable seeing a female GP about problems relating to the prostate, so having a male interviewer may have helped patient participants be more comfortable and open in the interviews. GP participants may have been more comfortable in talking to a peer in these interviews; peer discussions are a common part of professional practice for GPs in the form of Balint groups[25] and annual appraisal by a fellow GP[26].

Some GPs were reluctant to engage in any discussion about prostate MRI as they felt it was outside their current scope of practice and may have been focused on the more traditional (pre-MRI) prostate cancer pathway. MRI is a new test for prostate cancer and has only recently been integrated into diagnostic pathways. GPs are not currently able to request an MRI of the prostate, and access to MRI for other indications varies across the NHS. In this context, data gathered from GP participants was not as rich as the data collected from the patients and more limited insights were generated. A further potential limitation to the clinician insights gained from this study is that only GPs were recruited, and not other clinicians involved in the prostate cancer diagnostic pathway such as urologists or radiologists

Implications for patients, clinicians and health service design

Men's experiences of the prostate cancer diagnostic pathway are influenced by the appointment burden they face to receive a diagnosis; the mode of communication used by GPs and specialists to communicate test results; and requirements for travel to attend clinic appointments and tests. Significant challenges remain for GPs owing to the limitations of the current clinical signs and tests they rely on to identify possible prostate cancer cases. Men seemed broadly positive about MRI as a new test for prostate cancer, whereas GPs were equivocal owing to a lack of awareness and access. Improvements to patient experience of prostate cancer diagnostic pathways could be achievable through shorter time intervals to MRI, reduced outpatient appointment burden for patients, and access to more accurate and reliable triage testing in primary care.

For peer review only - http://bmjopen.bmj.com/site/about/guidelines.xhtml

For peer review only

Funding

SWDM is supported by the Can Test Collaborative, which is funded by CRUK (C8640/A23385). FMW and WH are co-directors of CanTest. HA's research is supported by core funding from the United Kingdom's National Institute of Health Research (NIHR) Imperial Biomedical Research Centre (no award/grant number). Ahmed currently receives funding from the Wellcome Trust, Medical Research Council (UK), Cancer Research UK, Prostate Cancer UK, National Institute for Health Research (UK), The Urology Foundation, BMA Foundation, Imperial Health Charity, NIHR Imperial BRC, Sonacare Inc., Trod Medical and Sophiris Biocorp for trials in prostate cancer. DE-E receives funding from the Urology Foundation, the BMA Foundation for Medical Research, Imperial Health Charity and the Royal College of Surgeons of England.

Competing interests

HA was a paid medical consultant for Sophiris Biocorp in the previous 3 years. The remaining authors have no conflicts of interest to declare.

Acknowledgements

The authors would like to thank the participants for sharing their time and experiences for this study. We would also like to thank Pauline Sibley and Victor Mariano (Royal Devon & Exeter NHS Foundation Trust) for their assistance with patient recruitment.

Contributors

SWDM, WH and FMW conceived the study. SWDM developed the research protocol with contributions from FMW, AF, and SA. HA, JM and DE-E were local investigators who supervised recruitment into the study. SWDM undertook all interviews. SWDM, FMW, AF and SA performed the analysis. SWDM drafted the first version of the manuscript. All authors contributed to the development of the manuscript, and approved the final submitted version.

Ethics approval

Ethics committee approval was received from the NHS HRA South-West Frenchay research ethics committee (REC reference 19/SW/0040).

For peer review only

Patient consent for publication

Patient consent for publication of anonymised individual remarks was obtained at the time of recruitment into the study.

Data sharing statement

All data requests should be submitted in writing to the corresponding author

Disclaimer

The views expressed in this publication are those of the author(s) and not necessarily those of the NIHR or the Department of Health and Social Care.

For peer review only

References

1 Forster AS, Rubin G, Emery JD, *et al.* Measuring patient experience of diagnostic care and acceptability of testing. *Diagnosis* 2021;**0**. doi:10.1515/dx-2020-0112

2 NICE. Suspected cancer: recognition and referral. 2015;1–95.https://www.nice.org.uk/guidance/ng12 (accessed 13 Oct 2021).

3 McPhail S, Johnson S, Greenberg D, *et al.* Stage at diagnosis and early mortality from cancer in England. *Br J Cancer* 2015;**112**:S108–15. doi:10.1038/bjc.2015.49

4 Sandager M, Jensen H, Lipczak H, *et al.* Cancer patients' experiences with urgent referrals to cancer patient pathways. *European Journal of Cancer Care* 2019;**28**:1–11. doi:10.1111/ecc.12927

5 Fraser J, Pearson C, Shelton J, *et al.* Using linked English cancer registration data to assess variation in diagnostic pathway length. *International Journal of Population Data Science* 2019;**4**:121.

6 Clements A, Watson E, Rai T, *et al.* The PSA testing dilemma: GPs' reports of consultations with asymptomatic men: a qualitative study. *BMC Family Practice* 2007;**8**:35. doi:10.1186/1471-2296-8-35

7 Pickles K, Carter SM, Rychetnik L. Doctors' approaches to PSA testing and overdiagnosis in primary healthcare: A qualitative study. *BMJ Open* 2015;**5**. doi:10.1136/bmjopen-2014-006367

8 NICE. Prostate Cancer: Diagnosis and Management. ng131. 2019. doi:10.1097/00005392-200308000-00097

9 Merriel SWD, Walter FM, Hamilton W. Integrating multiparametric MRI into prostate cancer diagnostic pathways within the NHS and internationally – A diagnostic pathways mapping study. In: *Cancer REsearch UK 5th Biennial Early Diagnosis Research Conference*. Birmingham: Cancer Research UK 2019. https://www.cancerresearchuk.org/health-professional/diagnosis/early-diagnosis-initiative/early-diagnosis-research-conferences/2019-conference-programme-and-registration

10 Drost FJ, Osses DF, Nieboer D, *et al.* Prostate MRI, with or without targeted biopsy and standard biopsy for detecting prostate cancer: A Cochrane systematic review and meta-analysis. *Cochrane Database of Systematic Reviews* 2019;CD012663. doi:10.1016/s1569-9056(19)30534-2

11 Scott SE, Walter FM, Webster A, *et al.* The model of pathways to treatment: Conceptualization and integration with existing theory. *British Journal of Health Psychology* 2013;**18**:45–64. doi:10.1111/j.2044-8287.2012.02077.x

12 Walter F, Webster A, Scott S, *et al.* The Andersen Model of Total Patient Delay: A systematic review of its application in cancer diagnosis. *Journal of Health Services Research and Policy* 2012;**17**:110–8. doi:10.1258/jhsrp.2011.010113

13 Silverman D. *Doing qualitative research*. Sage 2013.

14 Guba EG, Lincoln YS. Competing paradigms in qualitative research. In: *Handbook of Qualitative Research*. 1994. 105–17.https://instructure-uploads.s3.amazonaws.com/account_10020000000097009/attachments/62359215/Guba and Lincoln 1994.pdf?AWSAccessKeyId=AKIAJFNFXH2V2O7RPCAA&Expires=1428527480&Signature=Avqyb1esHciQeE6ROflRDQuQZ2M%253D&response-content-disposition=attachment

For peer review only - http://bmjopen.bmj.com/site/about/guidelines.xhtml

15 Denzin NK, Lincoln YS. *The sage handbook of qualitative research*. Sage 2011.
16 Braun V, Clarke V. Using thematic analysis in psychology. *Qualitative Research in Psychology* 2006;**3**:77–101. doi:10.1111/j.1460-2466.1978.tb01621.x
17 Tong A, Sainsbury P, Craig J. Consolidated criterion for reporting qualitative research (COREQ): a 32- item checklist for interviews and focus group. *International Journal of Qualitative in Health Care* 2007;**19**:349–57. doi:10.1093/intqhc/mzm042
18 Ruseckaite R, Evans S, Millar J, *et al.* GPs' Insights into Prostate Cancer Diagnosis and Care in Regional Victoria, Australia. *The Qualitative Report* 2016;**21**:2365–79
19 Evans R, Edwards AGK, Elwyn G, *et al.* It's a maybe test': men's experiences of prostate specific antigen testing in primary care. *British Journal of General Practice* 2007;**57**:303–10.
20 James LJ, Wong G, Craig JC, *et al.* Men's perspectives of prostate cancer screening: A systematic review of qualitative studies. *PLoS ONE* 2017;**12**. doi:10.1371/journal.pone.0188258
21 Bhuyan SS, Chandak A, Gupta N, *et al.* Patient–Provider Communication About Prostate Cancer Screening and Treatment: New Evidence From the Health Information National Trends Survey. *American Journal of Men's Health* 2017;**11**:134–46. doi:10.1177/1557988315614082
22 Henry SG, Czarnecki D, Kahn VC, *et al.* Patient-physician communication about early stage prostate cancer: Analysis of overall visit structure. *Health Expectations* 2015;**18**:1757–68. doi:10.1111/hex.12168
23 Waldie M, Smylie J. Communication: the key to improving the prostate cancer patient experience. *Can Oncol Nurs J* 2012;**22**:129–39. doi:10.5737/1181912x222129133
24 Symonds RP, Lord K, Mitchell AJ, *et al.* Recruitment of ethnic minorities into cancer clinical trials: Experience from the front lines. British Journal of Cancer. 2012;**107**:1017–21. doi:10.1038/bjc.2012.240
25 Roberts M. Balint groups: A tool for personal and professional resilience. *Canadian Family Physician* 2012;**58**:2012.
26 NHS England. Appraisal. 2020.https://www.england.nhs.uk/medical-revalidation/appraisers/ (accessed 1 Apr 2020).

For peer review only

Figure 1 – South West Prostate Cancer Diagnostic Pathway, NHS Cancer Alliances in South-West Peninsula and Somerset, Wiltshire, Avon & Gloucester (SWAG)

mpMRI – multiparametric magnetic resonance imaging; TRUS – transrectal ultrasound guided biopsy; MDT – multidisciplinary team

Figure 2 – RAPID pathway, Imperial College Healthcare NHS Trust, London

2WW – Two Week Wait pathway; mpMRI – multiparametric magnetic resonance imaging; MRI – magnetic resonance imaging

Figure 3 – Thematic diagram from patient participant interviews

Figure 4 – Thematic diagram from GP participant interviews

For peer review only

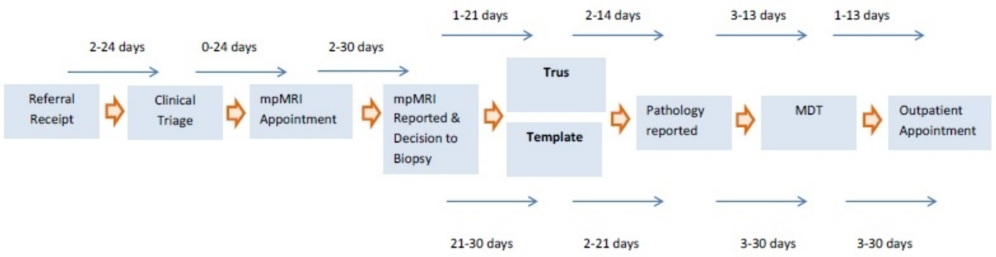

Figure 1 – South West Prostate Cancer Diagnostic Pathway, NHS Cancer Alliances in South-West Peninsula and Somerset, Wiltshire, Avon & Gloucester (SWAG)
mpMRI – multiparametric magnetic resonance imaging; TRUS – transrectal ultrasound guided biopsy; MDT – multidisciplinary team

207x55mm (96 x 96 DPI)

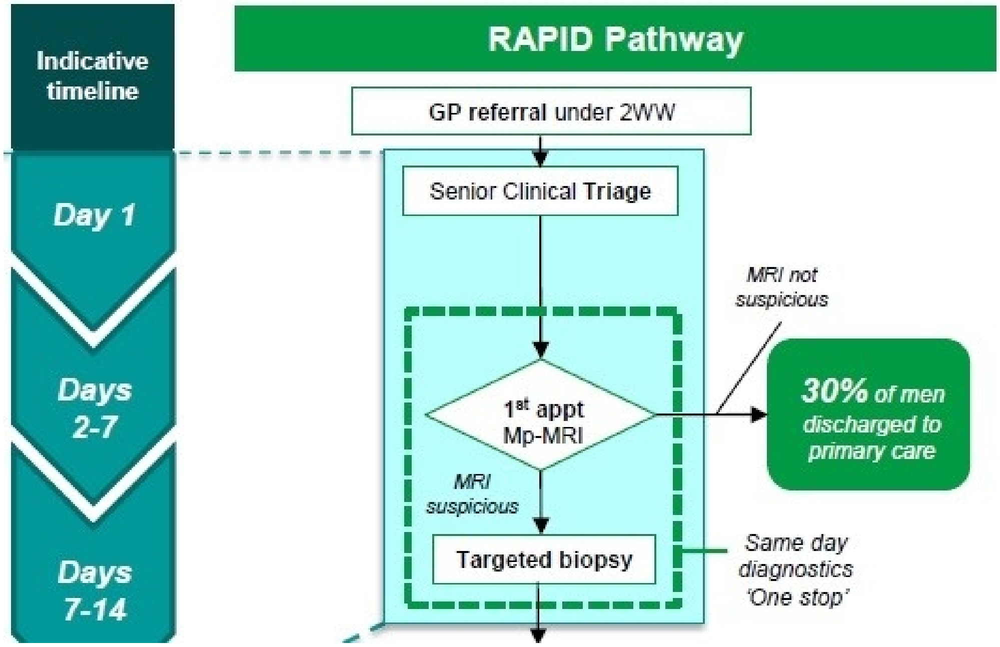

Figure 2 – RAPID pathway, Imperial College Healthcare NHS Trust, London
2WW – Two Week Wait pathway; mpMRI – multiparametric magnetic resonance imaging; MRI – magnetic resonance imaging

140x91mm (96 x 96 DPI)

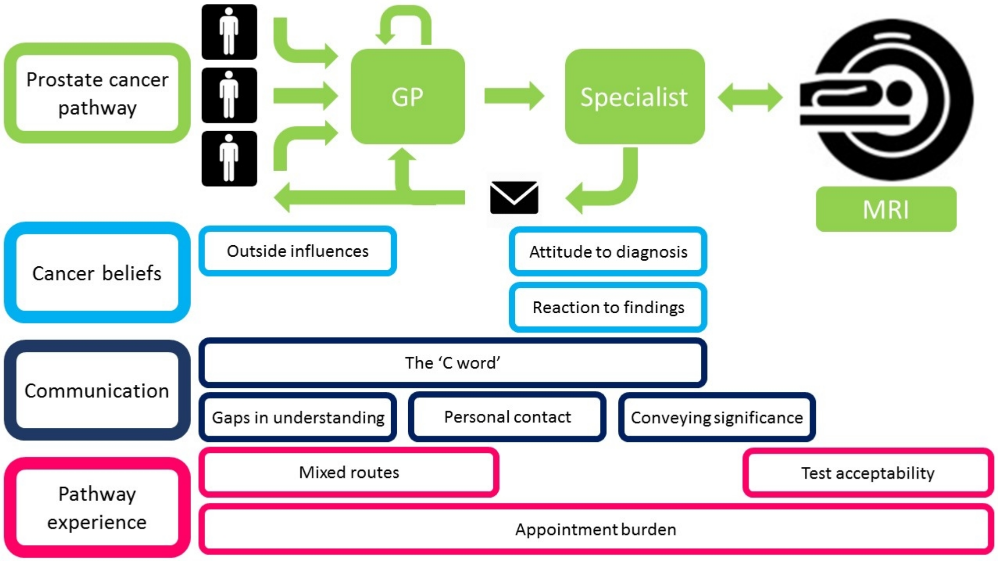

Figure 3 – Thematic diagram from patient participant interviews

338x190mm (96 x 96 DPI)

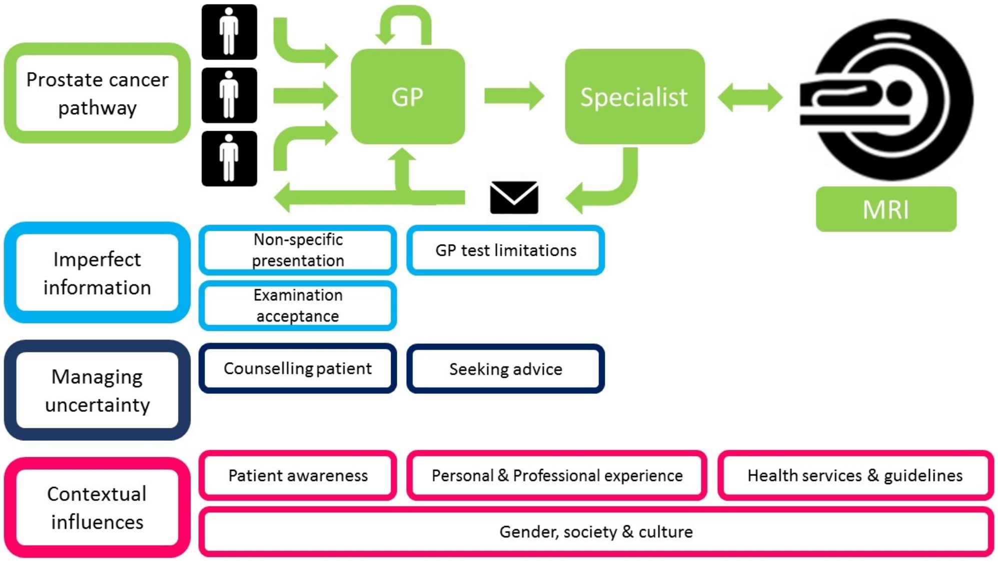

Figure 4 – Thematic diagram from GP participant interviews

338x190mm (96 x 96 DPI)

Patient participant interview guide

Introduction (5mins)
Thank you for agreeing to participate in this interview.

Introduce myself and my role.

This study seeks to understand your knowledge and understanding of diagnostic tests for prostate cancer, and your experiences of the current prostate cancer diagnostic pathway in the region where you live. It is part of my PhD at the University of Exeter. This study has been funded by Cancer Research UK, and has ethical approval from the NHS Health Research Authority and the University of Exeter.

As we've talked about with the consent form, participating in this study is voluntary and you can stop at any time. We want to know about your experiences and what you think, so there are no right or wrong answers.

This interview is being recorded for the purposes of qualitative analysis by the researchers. You can ask for the recording to be stopped at any time. What you say will be kept confidential and anonymous, unless we discuss something that suggests there is a significant risk to yourself or someone else. Everyone being interviewed will be asked the same questions, so if you don't have an answer to any of the questions that's fine, just say so and we can move on.

This interview study if focused on your experience of diagnostic test for prostate cancer. However, if you have a partner, family member or significant other who you wish to be present that's fine. Ideally we would start the interview without them, and then invite them in later on. If you and they are happy for them to participate, they would need to complete a consent form as well.

Ensure participant has copy of participant information sheet

Answer any questions

Ensure consent form is completed correctly

Commence audio recording

For peer review only

For peer review only - http://bmjopen.bmj.com/site/about/guidelines.xhtml

Basic demographics (5 minutes)
"To start with, can you tell me a little bit about yourself and your background"
Check - Age, Ethnicity, City/town lived in

Prostate cancer diagnosis journey (5-10 minutes)
"Now I would like to talk a bit about how you came to have tests for possible prostate cancer."
When did you first notice symptoms (if any)? Which symptoms were they?
How long until consulted you consulted your GP? What affected that decision?
How did GP assess? PSA? DRE?
Decision to refer – what do you remember about that discussion?

mpMRI for prostate cancer (10-15 minutes)
"I would now like to ask some questions about having an MRI scan for possible prostate cancer."
Did you have any concerns or reservations about having an MRI? What were your expectations?
What do you understand about how an MRI works?
What was your experience of having an MRI?

mpMRI result (10 minutes)
"If you are happy to discuss, I would like to ask a few questions about the results of your MRI scan and the next steps."
What was the result of your mpMRI? Did you understand it? How much did you trust the findings?
How were the results communicated? Did you have any questions as a result?
What were you told about the results' meaning?
What else did you discuss with the specialist?

Doctor(s) involved in cancer diagnosis (5 minutes)
"Finally, I would like to ask about your thoughts regarding the way we organise testing for prostate cancer."
What works well? What could be done better?
Who do you feel should be involved in investigating for a possible cancer diagnosis?
If you could design the ideal cancer diagnosis testing service, how would it work?

Interview close (2 minutes)
Thank you for participating in this interview. The data you have provided will be transcribed under a pseudonym, and analysed by the research team. You will be sent a final study report after the analysis has been completed.
If you have any questions or concerns about the study, please contact Ms Pam Baxter at the Research Ethics and Governance Office at the University of Exeter on 01392 723588 or via email p.r.baxter2@exeter.ac.uk Her details are on your patient information leaflet.

For peer review only

For peer review only - http://bmjopen.bmj.com/site/about/guidelines.xhtml

GP participant interview guide

Introduction (5mins)
Thank you for agreeing to participate in this interview.

Introduce myself and my role.

This study seeks to understand your knowledge and understanding of diagnostic tests for prostate cancer, and your experiences of the current prostate cancer diagnostic pathway in the region where you work. It is part of my PhD at the University of Exeter. This study has been funded by Cancer Research UK, and has ethical approval from the NHS Health Research Authority and the University of Exeter.

As we've talked about with the consent form, participating in this study is voluntary and you can stop at any time. We want to know about your experiences and what you think, so there are no right or wrong answers.

This interview is being recorded for the purposes of qualitative analysis by the researchers. You can ask for the recording to be stopped at any time. What you say will be kept confidential and anonymous, unless we discuss something that suggests there is a significant risk to yourself or someone else. This interview is not assessing your clinical competence, and we want to hear about your approach and experiences. Everyone being interviewed will be asked the same questions, so if you don't have an answer to any of the questions that's fine, just say so and we can move on.

This interview study if focused on your experience of diagnostic test for prostate cancer. However, if you have a partner, family member or significant other who you wish to be present that's fine. Ideally we would start the interview without them, and then invite them in later on. If you and they are happy for them to participate, they would need to complete a consent form as well.

Ensure participant has copy of participant information sheet

Answer any questions

Ensure consent form is completed correctly

Commence audio recording

For peer review only

For peer review only - http://bmjopen.bmj.com/site/about/guidelines.xhtml

Basic demographics (5 minutes)
"To start with, can you tell me a little bit about yourself and your background"

Age, Gender, Years of GP experience, Main CCG area you work in

Decision to refer for suspected prostate cancer (10-15 minutes)
"I would like to now move on to your current practice around referring men with suspected prostate cancer for further investigation"

"What symptoms/signs do you enquire about when assessing a man for suspected prostate cancer? How do they affect your decision to refer?"

PSA use – When would you offer it to a man? What are the important points you make about PSA when counselling a man about the test? What do you do with a negative PSA?

"What other factors, if any, affect your decision to refer a man for further investigation?"

"What are the key points you discuss with men when making a referral?"

Diagnostic testing for prostate cancer (15-20 minutes)
"Now I would like to ask some questions about diagnostic tests for prostate cancer."

What is your experience of the prostate cancer diagnosis pathway in your region?
What do you know about current diagnostic tests? How accurate do you believe current diagnostic tests are for prostate cancer?
PROMIS trial – have you heard of it? Are you aware of use of mpMRI for prostate cancer?
Do you feel incorporating mpMRI into the prostate cancer diagnosis pathway would be beneficial for patients? Do you believe it could be cost effective?
What would be the characteristics of an ideal diagnostic test for prostate cancer?

Men diagnosed with prostate cancer (5-10 minutes)
"Finally, I would like to ask about any of your patients who have been diagnosed with prostate cancer"

Are you aware of any of your patients diagnosed with low-grade prostate cancer?
If so, what has been the impact of the diagnosis on patient?
Are you aware of any of your patients being put on active surveillance – what is your experience of interacting with these men after diagnosis?

Interview close (2 minutes)
Thank you for participating in this interview. The data you have provided will be transcribed under a pseudonym, and analysed by the researchers. You will be sent a final study report after the analysis has been completed. If you have any questions or concerns about the study, please contact Ms Pam Baxter at the Research Ethics and Governance Office at the University of Exeter on 01392 723588 or via email p.r.baxter2@exeter.ac.uk

For peer review only - http://bmjopen.bmj.com/site/about/guidelines.xhtml

'Acceptability, understanding and experience of diagnostic tests for prostate cancer: a qualitative study with patients and GPs'

IRAS ID: 259602

Protocol Version Number: 1.0

Protocol date: 21/01/2019

Sponsor: University of Exeter

Sponsor Protocol Reference Number: 1819/03

Chief Investigator: Dr Sam Merriel

Supervisors:

Dr Fiona Walter, University of Cambridge

Dr Alice Forster, University College London

Professor Willie Hamilton, University of Exeter

This protocol has regard for the HRA guidance and order of content

For peer review only

For peer review only - http://bmjopen.bmj.com/site/about/guidelines.xhtml

SIGNATURE PAGE

The undersigned confirm that the following protocol has been agreed and accepted and that the Chief Investigator agrees to conduct the study in compliance with the approved protocol and will adhere to the principles outlined in the Declaration of Helsinki, the Sponsor's SOPs, and other regulatory requirement.

I agree to ensure that the confidential information contained in this document will not be used for any other purpose other than the evaluation or conduct of the investigation without the prior written consent of the Sponsor

I also confirm that I will make the findings of the study publically available through publication or other dissemination tools without any unnecessary delay and that an honest accurate and transparent account of the study will be given; and that any discrepancies from the study as planned in this protocol will be explained.

For peer review only

For and on behalf of the Study Sponsor:

Signature:

Date: 30/01/2019

Name (please print): Ms Pam Baxter

Position: Senior Research Governance Officer

University of Exeter

Chief Investigator:

Date: 30/01/2019

Signature:

Name: (please print): Dr Sam Merriel

For peer review only - http://bmjopen.bmj.com/site/about/guidelines.xhtml

LIST of CONTENTS

**GENERAL INFORMATION**	**Page No.**
HRA PROTOCOL COMPLIANCE DECLARATION AND SIGNATURE PAGE	ii
LIST OF CONTENTS	iii
KEY STUDY CONTACTS	iv
STUDY SUMMARY	v
FUNDING	v
ROLE OF SPONSOR AND FUNDER	vi
ROLES & RESPONSIBILITIES OF STUDY STEERING GROUPS AND INDIVIDUALS	vi
**SECTION**	
STUDY FLOW CHARTS	1
1. BACKGROUND	4
2. RATIONALE	4
3. THEORETICAL FRAMEWORK	5
4. RESEARCH QUESTION/AIM(S)	5
5. STUDY DESIGN/METHODS	6
6. SAMPLE AND RECRUITMENT	7
7. ETHICAL AND REGULATORY COMPLIANCE	8
8. DISSEMINATION POLICY	12
9. REFERENCES	12
10. APPENDICES	15

For peer review only

For peer review only - http://bmjopen.bmj.com/site/about/guidelines.xhtml

KEY STUDY CONTACTS

Chief Investigator<br>('The researcher')	Dr Sam Merriel<br>Clinical Senior Research Fellow, Exeter Medical School, University of Exeter<br>1.18, College House, St Luke's Campus, University of Exeter, Heavitree Road, Exeter EX1 2LU<br>s.w.d.merriel@exeter.ac.uk<br>01392 726090
Supervisors	Dr Fiona Walter<br>Principal Researcher in Primary Care Cancer Research, Department of Public Health and Primary Care, University of Cambridge<br>The Primary Care Unit, University of Cambridge School of Clinical Medicine, Box 113 Cambridge Biomedical Campus, Cambridge, CB2 0SR<br>fmw22@medschl.cam.ac.uk<br>01223 762514<br>Dr Alice Forster<br>Senior Research Fellow, Institute of Epidemiology & Health, Faculty of Population Health Sciences, University College London<br>1-19 Torrington place, London, WC1E 6BT<br>alice.forster@ucl.ac.uk<br>Professor Willie Hamilton<br>Professor of Primary Care Diagnostics, Exeter Medical School, University of Exeter<br>1.17, College House, St Luke's Campus, University of Exeter, Heavitree Road, Exeter EX1 2LU<br>w.hamilton@exeter.ac.uk<br>01392 726097
Sponsor	University of Exeter<br>Sponsor's representative<br>Ms Pam Baxter<br>Senior Research Governance Officer<br>Research Ethics and Governance Office,<br>University of Exeter<br>Lafrowda House, St Germans Road, Exeter,<br>Devon, EX4 6TL<br>p.r.baxter2@exeter.ac.uk<br>01392 723588

For peer review only

For peer review only - http://bmjopen.bmj.com/site/about/guidelines.xhtml

Funder(s)	Cancer Research UK

STUDY SUMMARY

Study Title	'Acceptability, understanding and experience of diagnostic tests for prostate cancer: a qualitative study with patients and GPs'
Internal ref. no. (or short title)	Acceptability of mpMRI for prostate cancer diagnosis in primary care
Study Design	Qualitative interview study
Study Participants	Males who have undergone multiparametric Magnetic Resonance Imaging (mpMRI) for suspected prostate cancer.<br>General Practitioners (GPs)
Planned Size of Sample (if applicable)	Purposive sample of approximately 10 GPs and 20 patients
Planned Study Period	01/03/2019 – 01/07/2020
Research Question/Aim(s)	**Aim**<br>To understand, from the perspective of patients and GPs, the acceptability of multiparametric magnetic resonance imaging for men as a diagnostic test for prostate cancer<br>**Objectives**<br>1. Elicit men's experiences of diagnostic tests for suspected prostate cancer<br>2. Explore the knowledge and understanding of diagnostic tests for suspected prostate cancer amongst patients and GPs<br>3. Understand the acceptability of mpMRI as a diagnostic test for prostate cancer from a patient's perspective<br>4. Understand the acceptability of mpMRI as a diagnostic test for prostate cancer from a GP's perspective

FUNDING AND SUPPORT IN KIND

FUNDER(S)	FINANCIAL AND NON FINANCIALSUPPORT GIVEN

For peer review only - http://bmjopen.bmj.com/site/about/guidelines.xhtml

Cancer Research UK	Funding: CanTest (Cancer Research UK Catalyst Award) - Clinical Research Fellow<br>Duration: 3 years 8 months (at 60% FTE)<br>Start date: 02/08/2018<br>Travel costs for researchers and participants to attend face to face interviews<br>Service support costs for recruitment sites<br>Transcription of interviews
National Institute for Health Research Clinical Research Network	Adoption of study onto NIHR portfolio<br>Recruitment of GP participants through CRN practices

ROLES AND RESPONSIBILITIES OF STUDY MANAGEMENT COMMITEES/GROUPS & INDIVIDUALS

The study management group with comprise the researcher, the supervisors, and a PPI representative. The researcher will report to the group, who will oversee the recruitment, data collection and analysis for the duration of the study. The study management group will be independent from the sponsor and the funders.

PROTOCOL CONTRIBUTORS

The study sponsor provided support to the researcher in the preparation of the study protocol, consent forms, patient information leaflets and interview guides. The sponsor will have no role in any other aspect of the conduct, analysis, or dissemination of the study.

The funder had no role in any aspect of the preparation of the study, and will have no role in the conduct, analysis or dissemination of the study.

Patients and members of the public have been involved with writing the lay summary, consent forms, patient information leaflets and interview schedules.

SPONSOR

The study sponsor (the University of Exeter) has ensured that the research team, research protocol and research sites are suitable and that indemnity arrangements are in place. In reviewing the research and ethics documentation, the sponsor has further ensured that appropriate risk management is in place and that the study is managed and conducted in accordance with relevant legislation and codes of good practice. The sponsor will ensure that relevant approvals are in place before the study begins, that the study is conducted in accordance with the protocol and relevant approvals, and that appropriate record-keeping and data management is maintained. The sponsor must approve any study amendments or modifications and will be notified of any significant developments or adverse events in accordance with appropriate guidelines. The sponsor has reviewed plans for data storage and retention and plans for dissemination of the research findings.

KEY WORDS: Prostate cancer; MRI; diagnosis; primary care; acceptability

For peer review only - http://bmjopen.bmj.com/site/about/guidelines.xhtml

For peer review only

For peer review only - http://bmjopen.bmj.com/site/about/guidelines.xhtml

UNIVERSITY OF EXETER | MEDICAL SCHOOL

CanTest
Right place, right time, by your family doctor

Acceptability of mpMRI for prostate cancer diagnosis in primary care v1.0

STUDY FLOW CHART 1 – Patient participant recruitment at Imperial College Healthcare

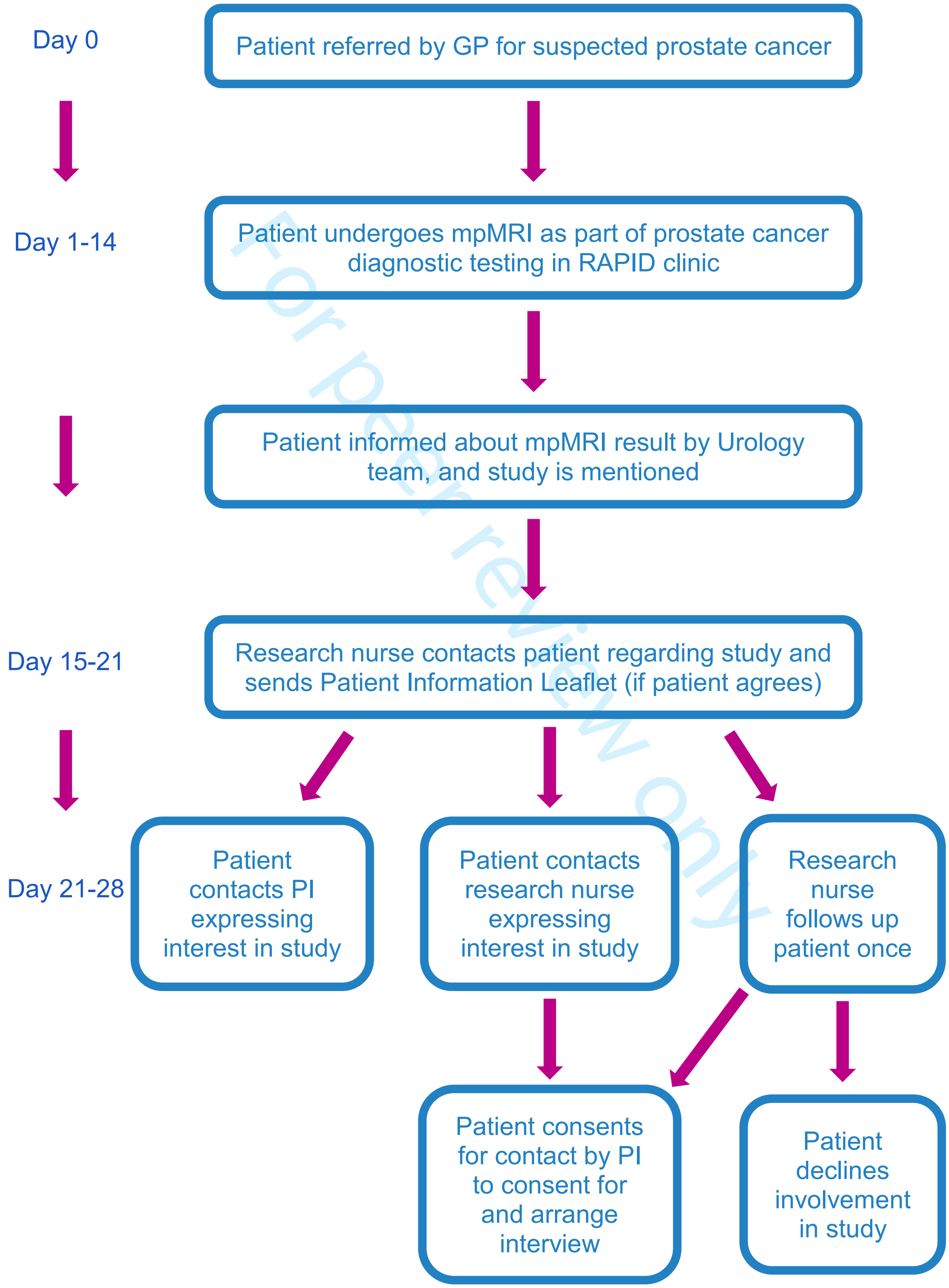

For peer review only - http://bmjopen.bmj.com/site/about/guidelines.xhtml

STUDY FLOW CHART 2 – Patient participant recruitment at Royal Devon & Exeter

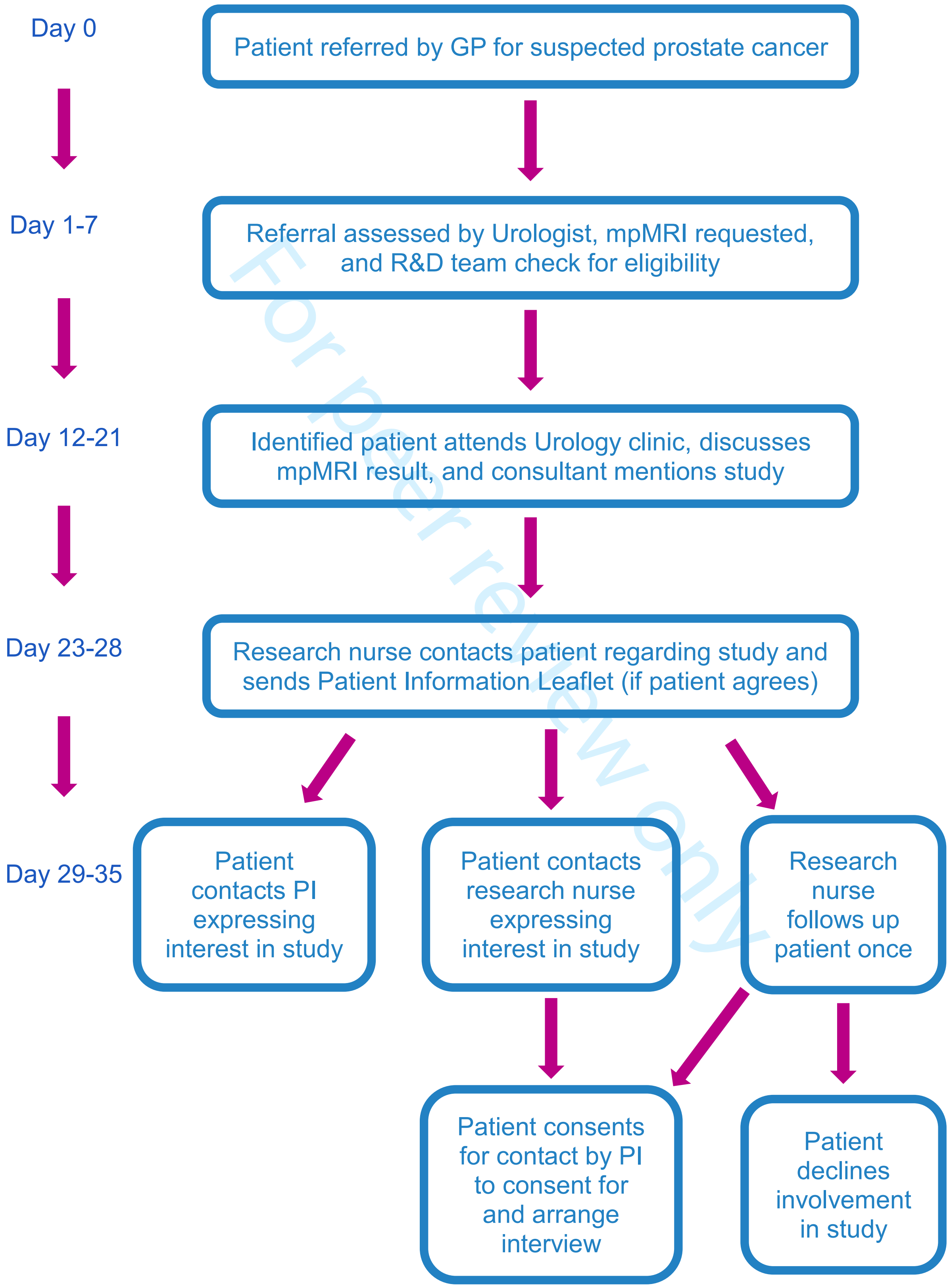

For peer review only

For peer review only - http://bmjopen.bmj.com/site/about/guidelines.xhtml

STUDY FLOW CHART 3 – GP participant recruitment via Clinical Research Networks

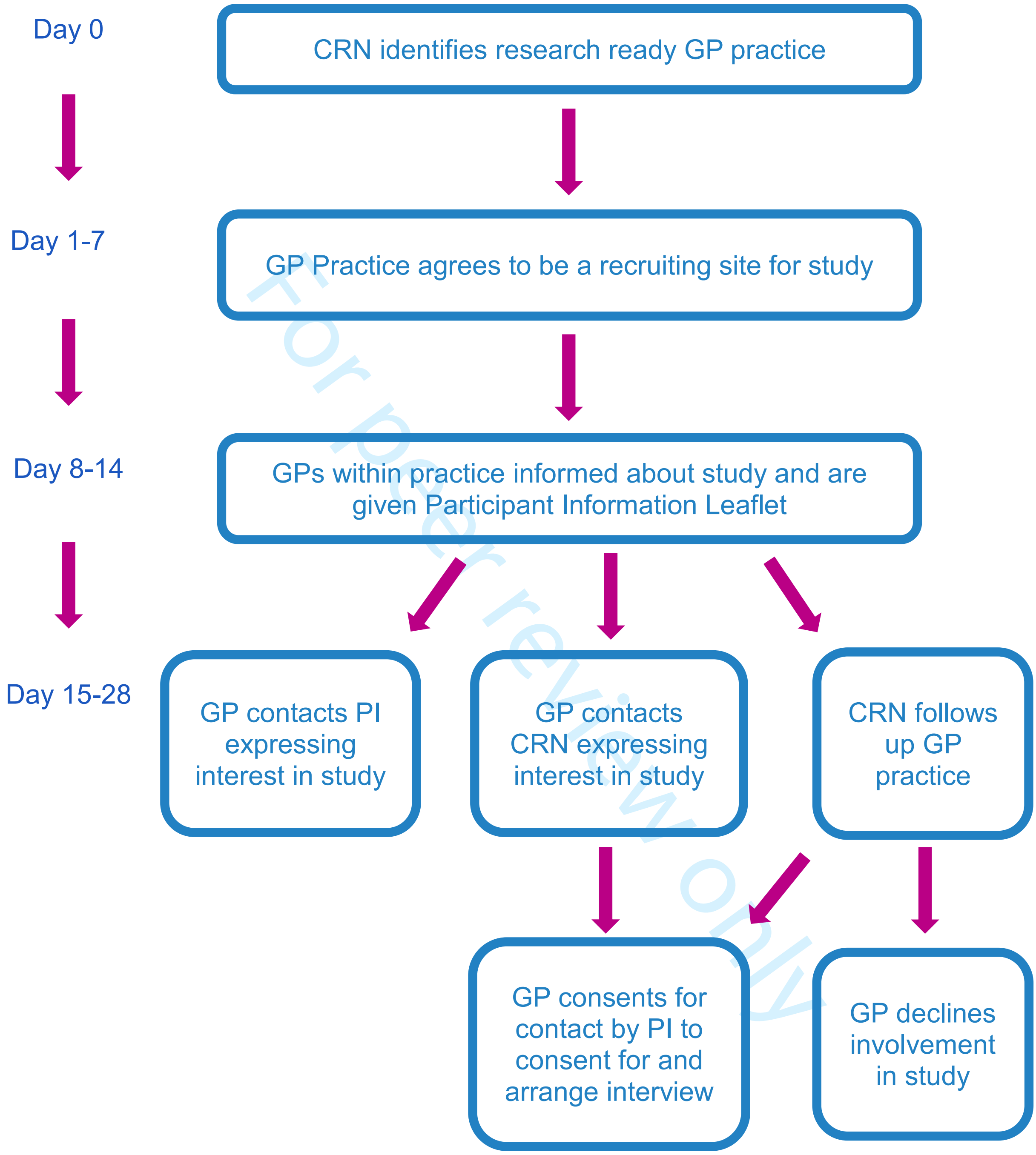

For peer review only

For peer review only - http://bmjopen.bmj.com/site/about/guidelines.xhtml

STUDY PROTOCOL

'Acceptability, understanding and experience of diagnostic tests for prostate cancer: a qualitative study with patients and GPs'

1 BACKGROUND

Implementation of diagnostic tests into routine clinical practice should follow a rigorous process of evaluation from showing analytical validity and diagnostic accuracy, through to acceptability and cost effectiveness. A number of frameworks for assessing and evaluating tests for use in healthcare have been proposed(1–5). They suggest the test should be able to be performed by the operator(s); it should demonstrate more patient benefit than harm; it should be cost effective relative to currently available tests; it should be able to be integrated into the diagnostic pathway; and it should be acceptable to patients and clinicians.

Cancer diagnosis pathways in the NHS in the UK involve primary and secondary care clinicians(6). Some diagnostic tests can be ordered through 'direct access' by a patient's General Practitioner (GP) if they present with symptoms or signs that could indicate an undiagnosed cancer. These include gastroscopy for oesophageal or gastric cancer; colonoscopy for bowel cancer; flexible sigmoidoscopy for rectal cancer; and CT or MRI head for brain tumours(6). Diagnostic tests for prostate cancer currently requires a referral to secondary care.

The current gold standard diagnostic test for prostate cancer is a transrectal ultrasound-guided (TRUS) biopsy of the prostate. TRUS biopsy procedures take 6-12 samples from different regions of the prostate, which are then examined by a histopathologist for signs of prostate cancer(7). TRUS biopsy carries a risk of infection and sepsis, and there is a risk of under- or misdiagnosis as a result of the random nature of sampling the prostate(8). Multiparametric MRI (mpMRI) scanning of the prostate, and reporting using the PiRADS(9) reporting system, has recently been compared to TRUS biopsy in recent large, multicentre trials(10,11) with favourable results in terms of diagnostic accuracy. Few studies have been performed assessing other aspects of the implementation of mpMRI for prostate cancer diagnosis, including patient experience and clinician acceptability.

2 RATIONALE

Prostate cancer is the most common malignancy in males in the United Kingdom(UK)(12). Whilst prostate cancer is responsible for a significant number of cancer-related deaths, the 5- and 10-year survival rates for men with prostate cancer is high(13). This has partly been driven by an increase in the numbers of clinically insignificant prostate cancer cases being diagnosed in the last three decades(14). Better diagnostic tests and diagnostic pathways are needed to reduce rates of over-diagnosis of clinically insignificant prostate cancer, and mpMRI may have a role in this. Following on from the PROMIS trial(10) and others like it(11,15,16), NHS England issued guidance for 'Implementing a timed prostate cancer diagnostic pathway' to NHS Cancer Alliances, reinforcing the benefits for patients of integrating mpMRI into local diagnostic pathways(17).

Studies of patient acceptance of TRUS prostate biopsy for prostate cancer, the current diagnostic test, focus on prevalence of side effects and patient anxiety relating to the test(18–20). Two studies to date have assessed patient acceptance of mpMRI guided biopsy, which also involved questionnaires assessing side effects and attitudes towards the test(21,22). There are no studies that examine acceptability of mpMRI as a diagnostic test for prostate cancer with any theoretical underpinning, and questions remain about men's experience of undergoing the test and receiving the results. There are also very few studies of General Practitioners (GPs), or primary care clinicians, exploring their understanding of diagnostic tests for prostate cancer.

For peer review only - http://bmjopen.bmj.com/site/about/guidelines.xhtml

3 THEORETICAL FRAMEWORK

Acceptability of diagnostic tests has been measured in a number of ways, but no agreed definition for acceptability exists(23). Sekhon et al have proposed a ‘Theoretical Framework of Acceptability’ relating to healthcare interventions, not just diagnostic tests, which includes seven key constructs (See Figure 1): Affective attitude, Burden, Ethicality, Intervention coherence, Opportunity costs, Perceived effectiveness, Self-efficacy(24). This Framework has been developed to be applicable to both patients and clinicians involved in healthcare interventions, and has a number of key constructs that are particularly relevant to the study aims. Eliciting how a patient feels about undergoing mpMRI (‘Affective attitude’), the extent to which they understand the test and its purpose (‘Intervention coherence’), and how likely they perceive mpMRI will achieve the purpose of diagnosing prostate cancer (‘Perceived effectiveness’) will aid understanding in the acceptability of mpMRI as a diagnostic test.

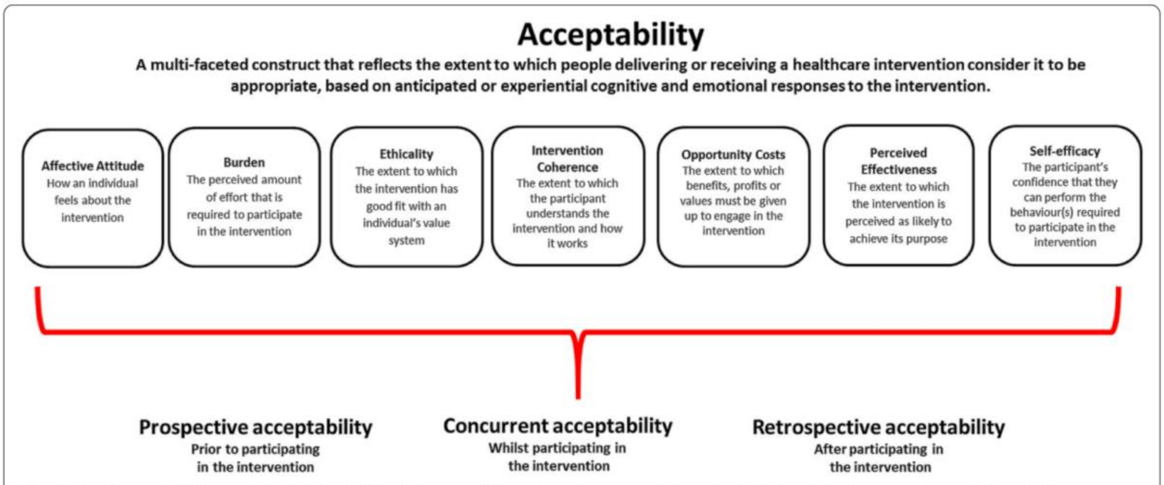

Fig. 3 The theoretical framework of acceptability (v2) comprising seven component constructs. Note: The seven component constructs are presented alphabetically with their anticipated definitions. The extent to which they may cluster or influence each of the temporal assessments of acceptability is an empirical question

Figure 1 – Sekhon’s Theoretical Framework of Acceptability(24)

Qualitative research methods lend themselves to answering questions of patient and clinician acceptability with regard to diagnostic tests. Such methods allow researchers to “uncover the nature of a person’s experience with a phenomenon”, such as cancer and “understand what lies behind any phenomena”(25). Interview studies provide the opportunity to dig deeper and explore how and why patients and clinicians form their beliefs and understanding. It is assumed by the researchers that every man will experience the prostate cancer diagnostic pathway and the diagnostic tests differently, influenced by both internal and external factors. Therefore, a constructivist approach will be taken to capture a range of experiences(26).

4 RESEARCH QUESTION/AIM(S)

Aim - To understand, from the perspective of patients and GPs, the acceptability of multiparametric magnetic resonance imaging for men as a diagnostic test for prostate cancer

4.1 Objectives

1. Elicit men’s experiences of diagnostic tests for suspected prostate cancer
2. Explore the knowledge and understanding of diagnostic tests for suspected prostate cancer amongst patients and GPs

For peer review only - http://bmjopen.bmj.com/site/about/guidelines.xhtml

3. Understand the acceptability of mpMRI as a diagnostic test for prostate cancer from a patient's perspective
4. Understand the acceptability of mpMRI as a diagnostic test for prostate cancer from a GP's perspective

4.2 Outcome

This study seeks to understand the acceptability of mpMRI as a diagnostic test for prostate cancer amongst patients and GPs. mpMRI is increasingly being used as part of the assessment of men with suspected prostate cancer by Urologists prior to undertaking a prostate biopsy, however it is unknown how men experience mpMRI scanning. Studies suggest that mpMRI has a negative predictive value of 85-89%(16,27), and that up to 27% of men could avoid a prostate biopsy based on mpMRI findings(11). Within the NHS, some GPs already have the ability to order 'direct access' diagnostic tests for suspected cancers of the oesophagus, stomach, colon, pancreas and brain, and there is some evidence that pre-biopsy mpMRI could also be used as a 'rule out' test in a prostate cancer diagnostic pathway for some patients(28). Before such an approach could be tested and implemented, the experience of patients undergoing mpMRI, and the acceptability of mpMRI as a diagnostic test for patients and GPs needs to be understood.

5 STUDY DESIGN and METHODS of DATA COLLECTION AND DATA ANALYIS

5.1 Study design

This qualitative study will employ semi-structured interviews with men referred from primary care with suspected prostate cancer who have undergone mpMRI, and GPs who have referred men with suspected prostate cancer for further investigation.

5.2 Data collection

Interviews will be conducted by the lead researcher (SM). Interview data will be gathered using an encrypted recording device. The location of the patient interviews will be agreed between the participant and the interviewer prior to the day of the interview. Ideally they will be conducted face-to-face in the patient's home, but other venues and telephone/Skype interviews will be considered. GP interviews will either be held face-to-face at the GP clinic, or via telephone/Skype. The interviewer will utilise a 'buddy system' of informing a colleague if they are travelling to a private residence unaccompanied to conduct an interview with a patient participant.

The interviews will be conducted in a semi-structured manner, allowing participants to share their experiences of diagnostic tests for prostate cancer freely, whilst also meeting the study objectives. Interviews will be supported by topic guides (see 10.1.6 and 10.1.7) for patient and GP participants, which will be used by the interviewer in a flexible way depending on the length and direction of the interview. These topic guides were developed by the researchers based on their experience and knowledge in the field and the study objectives, and will be adapted iteratively as the initial interviews are conducted to enrich data collection.

It is important to treat all participants equally, regardless of their age, culture, education, language ability, or beliefs. Efforts will be made to respect participants' needs, however this study is not sufficiently funded to meet all possible participant needs, such as interpreters for participants with English as their second language.

Following completion of each interview, audio recordings will immediately be downloaded onto an encrypted university laptop computer and the interviewer will make reflective and summary notes. Audio recordings will be transferred to an independent transcribing service securely and transcribed

For peer review only

For peer review only - http://bmjopen.bmj.com/site/about/guidelines.xhtml

verbatim. All participants will be given a pseudonym and any potentially identifying information will be removed.

5.3 Data analysis

A Framework Analysis approach will be taken for this study. Framework analysis is a type of thematic analysis developed by Jane Ritchie and Liz Spencer for applied social policy research(29), and is used in a wide range of areas including health research. Framework Analysis follows seven stages(30):

1. Transcription
2. Familiarisation
3. Coding
4. Developing a working framework
5. Applying framework
6. Charting data into matrix
7. Interpretation

After transcription of the interviews and familiarisation with the data by the investigators, early interview data will be coded using pre-specified codes based on Sekhon's Framework. Refinement and addition of codes and themes will occur with a second researcher (AF) and patient/public representative after coding of initial transcripts using constant-comparison method. The analysis team (SM, AF, and FW) will meet regularly to iteratively develop and agree a coding structure to underpin coding of the remaining transcripts, and a framework will be developed and applied using the agreed codes. SM will perform the final coding of the data. Key themes and narratives within the data will be drawn together from the matrix. Charted data will be imported into NVivo v12 to help manage the data to complete the analysis. Convergence and divergence of views from patients with positive and negative mpMRI scans, and between patients and GPs, will be sought to triangulate key findings.

5.4 Role of the researchers

Three of the researchers (SM, FW, and WH) are trained as GPs, and two are still practicing (SM, FW). All members of the research team will maintain an awareness of their individual biases, beliefs and attitudes that could influence the undertaking of research into men being investigated for prostate cancer. Reflective notes, analysis team meetings, and constant comparison techniques will be used to understand these influences.

6 SAMPLE AND RECRUITMENT

6.1 Eligibility Criteria

This study will recruit participants from two populations;

Patients with suspected prostate cancer who have undergone mpMRI as part of their diagnostic workup.

GPs who have referred at least one male for investigation for suspected prostate cancer within the preceding 12 months.

6.2 Sampling

6.2.1 Size of sample

Approximately 30 participants (10 GPs and 20 patients) will be interviewed for this study, although the final number of participants will depend on when no new themes emerge during interview coding.

For peer review only - http://bmjopen.bmj.com/site/about/guidelines.xhtml

6.2.2 Sampling technique

A purposive sampling approach will be taken for participant recruitment to this study. This will allow recruitment of a sample of men with a range of PiRADS scores (1-2 being low risk of prostate cancer; 3-5 being medium-high risk), ages (<70 years or 70+ years), geographical locations (urban or rural/countryside), and ethnic backgrounds (any white background or BME). In terms of GPs, a purposive sampling approach will allow recruitment of clinicians with a range of ages, genders, practice locations (urban or rural/countryside) and levels of experience.

6.3 Recruitment

6.3.1 Sample identification

6.3.1.1 Patient participants

Patient participants for this study will be recruited from two NHS Trusts; the Imperial College Healthcare NHS Trust in London and the Royal Devon & Exeter NHS Foundation Trust in Exeter. Men referred by their GP for suspected prostate cancer undergo an mpMRI prior to clinical review by a Urologist and potentially a prostate biopsy, depending on the mpMRI report (see study flow charts [pg 1-2]). Research nurses and/or fellows working within the clinic will identify potentially eligible men and contact them within days of undergoing an mpMRI to discuss this study and offer the men a Patient Information Leaflet (PIL – See 11.1.2). The PI and staff at the study sites will regularly communicate about potential recruits to ensure a range of age, ethnicity and geographical backgrounds are present in the included participants. Follow-up contact will be made by the research nurse/fellow if the man does not contact the lead researcher to check whether they wish to participate in the study or not.

Both NHS Trusts involved with this study have reviewed and approved this protocol, and they have expressed confidence that recruitment targets will be met. However, in the event that insufficient men are recruited for participation in this study 12 months after commencing recruitment, a further NHS Trust providing urology services that includes mpMRI for possible prostate cancer will be approached to aid recruitment.

Reasonable travel costs for patient participants to attend any face to face interview will be reimbursed, and participants will be offered a £20 gift voucher in recognition of their participation in the study.

6.3.1.2 GP participants

GP participants will be recruited through two National Institute for Health Research (NIHR) Clinical Research Networks (CRNs); North West London CRN and the South-West Peninsula CRN (see study flow chart [pg 3]). The CRNs will identify local practices from which to recruit eligible GPs to participate in this study, favouring Research Site Initiative (RSI) practices as these practices have an ongoing commitment to research and may have allocated research clinician time. In practices that do not have funded research clinician time, the CRN may provide support for participation in the study. Eligible GPs will be identified by the CRN and the practices, and they will regularly communicate with the PI to determine which GPs to approach for participation. GPs chosen for invitation into the study will be given a PIL (See 11.1.3) to consider participating in the study, and follow-up contact will be made by the CRN to confirm participation.

The NIHR CRN in South-West Peninsula and North West London have reviewed and approved this protocol, and they have expressed confidence in meeting recruitment targets. National adoption of this study within the NIHR CRN portfolio will allow the possibility of recruitment from other CRNs if there is any difficulty recruiting GPs in these two regions.

GP practices will be reimbursed £44.10 per 30 minute interview for the GP's time to participate.

6.3.2 Consent

For peer review only - http://bmjopen.bmj.com/site/about/guidelines.xhtml

All participants contacted for participation in this study will be given a PIL after being contacted as a potentially eligible participant. After reading the PIL, if the participant is willing to participate they will be contacted by the lead researcher to arrange an interview.

Consent will be taken at the start of the interview. The purpose of the study and the interview will be explained in conjunction with the information presented in the PIL. The lead researcher will assess the patient's ability to consent for participation. The participant will then be presented with a consent form (See 10.1.2 and 10.1.3) to complete if they are still willing to participate in the study.

Conducting patient interviews in their own home may result in other parties being present during the interview, such as the patient's spouse. If another person is present, the patient participant will be asked if they are happy to initially be interviewed in private. If the patient participant wishes for another person to be present, the additional person will be consented for participation and asked to complete a consent form before they join the interview.

6.3.3 Completing recruitment

Recruitment of patients and GPs will continue alongside analysis of interviews conducted to date, until no new themes or ideas emerge from the data.

7 ETHICAL AND REGULATORY CONSIDERATIONS

7.1 Data protection

All data will be collected, stored and processed in accordance with the General Data Protection Regulations 2018 and the Data Protection Act 2018. Informed consent will be obtained from all participants for all aspects of the study. Permission for the collection, storage and use of patient identifiable data (PID) in the study will be provided by consenting participants.

Participants will be free to withdraw consent for participation for any reason and at any time. Where consent is withdrawn, all participant identifiable information held by the research team will be destroyed, and the participant will not receive any further contact regarding the study.

7.2 Data anonymisation

All collected data will be fully anonymised before transfer to professional transcription services. Direct quotations from interview may be used in presenting the study results, however interviewees will not be identifiable in any way in any quotations used.

7.3 Data Storage

Encrypted voice recorders will be used for the interviews. Audio data will be downloaded and kept on secure servers at the University of Exeter until fully anonymised transcripts are created. Any audio files sent to professional transcription services will be anonymously labelled with a unique code, and encrypted for transfer. All physical data such as consent forms and transcripts of interviews will be stored within locked filing cabinets, within a locked office within the University of Exeter Medical School. The keys will be stored separately and only be accessed by the local research team.

All personal data will be securely destroyed within 12 months after the end of the study.

7.4 Assessment and management of risk

This study may be viewed as potentially sensitive in that it explores experiences of personal and intimate symptoms and body systems, in the context of a potentially serious diagnosis (cancer). Although there is a potential ethical problem with interviewing patients around the time of a cancer diagnosis, the proposed recruitment approaches have been successfully used in previous UK early

For peer review only - http://bmjopen.bmj.com/site/about/guidelines.xhtml

diagnosis of cancer studies, led by my supervisor, Dr Fiona Walter. These studies include questionnaire and interview studies people in patients with symptoms suggestive of lung, colorectal and pancreatic cancer (the SYMPTOM study)(31,32), an interview study set among people recently diagnosed with melanoma(33), and the on-going ECASS study (weekly case note reviews and subsequent GP and patient interviews in people with gastro-oesophageal symptoms).

Nevertheless, there are a number of strategies in place to minimise any possible distress that may arise in patients (and informal carers) during the study:

Interviews will be conducted by SM, who has been trained in qualitative interviewing. Combining with his experience as a GP, he would have had experience of communicating sensitively, empathetically and sympathetically with patients when breaking bad news and around sensitive topics, including cancer. Interviews will be conducted at a time and location convenient to participants, and in an unhurried manner, with participants being free to stop the discussion at any time. Appropriate leave-taking will also be practised to ensure that participants are not left in a distressed state following the interview.

It would be reasonable to assume that patients being recruited for interview are aware that they are being investigated with mpMRI due to the suspicion of them having prostate cancer. Local NHS protocols for referring men on the two-week wait urgent cancer referral pathway include the need for GPs to inform the patient they are being referred for a suspected diagnosis of cancer. However, the interviews for this study will not be conducted with that assumption. Early in the interview the patient's understanding for the reason for their referral for mpMRI and other investigations will be explored, to ensure that they patient's underlying knowledge and assumptions are clear to the interviewer.

SM is a practicing GP, and there is the possibility that patients will want to seek advice or an opinion form SM about their healthcare relating to the issue of possible prostate cancer or another health issue. SM will be clear that he is conducting the interview in his capacity as a researcher, and will refer any questions about the patient's healthcare back to their own GP.

A procedure is followed in the event of a participant becoming distressed, which includes the interviewer expressing concern as early as possible about the participant's comfort, offering them tissues or water, and asking whether they would like to take a break or discontinue the interview. Support mechanisms are in place (see Box 1) and the interviewer will inform the participant of these. Participants will also be reminded that they can withdraw from the study or complete the interview at another time, and that this is entirely their decision.

SM will meet regularly during data collection with his supervisors, FW and AF, who have extensive clinical and research experience with patients about cancer symptoms and pathways to diagnosis. This will ensure that should any issues arise they can be dealt with expediently, and learning applied for subsequent interviews.

For peer review only

For peer review only - http://bmjopen.bmj.com/site/about/guidelines.xhtml

Box 1: Guidelines for researchers conducting sensitive interviews

1. All interviews are to be conducted from the outset with the greatest of sensitivity and concern for the respondent's welfare.

2. The interviewer should be observant of the respondent's level of comfort and watch for early signs of distress, such as breaks in speech or nervous body movements. Should early signs appear the interviewer should express concern about the respondent's comfort and ask questions such as (gauged by respondent's signs): would they like a glass of water; if a break is needed; if they would prefer to complete the interview another time; or if they would prefer to discontinue.

3. If overt distress occurs, the interview should cease immediately and actions taken to support the respondent, such as offering tissues or water; seeking immediate additional support from a more familiar person, if available; and staying with the respondent until they are ready to express their wishes on the options available to them.

4. If it becomes apparent that a distressed respondent has particular areas of need concerning their illness or circumstances, where appropriate, the interviewer should offer to assist the respondent to make contact with a relevant support, such as their GP surgery.

5. Concerning the interview, the options eventually offered to a distressed respondent should be (in order): withdraw from the study; or complete the interview another time. The interview should only be continued after a break if the respondent requests this as their unprompted decision.

7.5 Adverse Events

An adverse event will be defined as 'an event that arises directly from participation in the research', including complications that occur in the course of investigation. All adverse events will be discussed with the Supervisors, both of whom (especially FW) have had extensive experience in carrying out similar studies involving early diagnosis of cancer. Appropriate subsequent course of action will be taken after discussion with the full research team, and the Sponsor will be notified.

Appropriate safety procedures will be followed by the researcher(s) when interviewing participants. Should any disclosures requiring action be made, the researchers will have access to the support of the full research team.

7.6 Insurance

Arrangements have been made through the University of Exeter for insurance and/or indemnity to meet the potential legal liability of the sponsor for harm to participants arising from the management, design or conduct of the research.

NHS indemnity scheme will apply for insurance and/or indemnity to meet the potential legal liability of the investigator arising from harm to participants in the conduct of the research at NHS sites.

There are no arrangements in place for payment of compensation in the event of harm to the research participants where no legal liability arises.

7.7 Research Ethics Committee (REC) and other Regulatory review & reports

The researcher will seek NHS research governance and compliance approval, and NHS ethical review, through the HRA approvals process via the Integrated Research Application System (IRAS).

For peer review only - http://bmjopen.bmj.com/site/about/guidelines.xhtml

The researcher will ensure that the protocol and all supporting participant-facing documentation receive HRA approval. Following review, research will only take place once appropriate HRA and Sponsor approvals are in place and confirmation of capacity and capability received from each local NHS site.

A copy of the approved study documents will be submitted to the R&D Office or practice manager at each local site prior to the commencement of any study procedures.

The Chief Investigator is responsible for keeping all correspondence with the REC, producing annual reports, notifying the REC at the end of the study and producing final reports.

7.8 Protocol compliance

Any accidental protocol deviations will be adequately documented on the relevant forms and reported to the Researcher and Sponsor. All deviations from the protocol which are found to frequently recur will require immediate action and could potentially be classified as a serious breach.

Notification of Serious Breaches to GCP and/or the protocol: A ‘serious breach’ is a breach which is likely to affect to a significant degree;

The safety or physical or mental integrity of the subjects of the study; or

The scientific value of the study

The Sponsor will be notified immediately of any case where the above definition applies during the study.

7.9 Amendments

For any amendment to the study, the Chief Investigator or designee, in agreement with the sponsor will submit information to the appropriate body in order for them to issue approval for the amendment. The Chief Investigator or designee will work with sites (R&D departments at NHS sites as well as the study delivery team) so they can put the necessary arrangements in place to implement the amendment to confirm their support for the study as amended.

If the sponsor wishes to make a substantial amendment to the REC application or the supporting documents, the sponsor must submit a valid notice of amendment to the REC for consideration. The REC will provide a response regarding the amendment within 35 days of receipt of the notice. It is the sponsor’s responsibility to decide whether an amendment is substantial or non-substantial for the purposes of submission to the REC.

Amendments will also be notified to the HRA national coordinating function of England where the lead NHS R&D office is based and communicated to the participating organisations (R&D office and local research team) and departments of participating sites to assess whether the amendment affects the NHS permission for that site.

Amendments considered to be non-substantial for the purposes of REC will still be notified to the HRA for approval after confirmation from the Sponsor.

7.10 Peer review

The PhD proposal, which this study forms a key part of, has been subject to peer review by two senior researchers within the CanTest programme. Both reviewers are external to the University of Exeter and are not involved with this study in any way. They are both very experienced and widely published primary care cancer researchers. Feedback from the peer review was utilised to refine and enchance the development of this study.

7.11 Patient & Public Involvement

For peer review only - http://bmjopen.bmj.com/site/about/guidelines.xhtml

The NIHR Clinical Research Facility Peninsula Research Bank steering committee includes a panel of lay members. 14 members of the panel, including two members who had previously been service users investigated for possible prostate cancer, provided input into the acceptability and design of this study. The lay members assisted in the writing of the lay summary, and they reviewed drafts of the consent form, participant information leaflets, and interview guides. Feedback received from these service users and members of the public has been integrated into this protocol and associated documents.

Separately, a PPI group of men (with no history of prostate cancer) is currently being assembled to help steer the PhD that this study forms a part of. This PPI group will be involved with the analysis of results and the dissemination of findings from this study.

7.12 Access to the final study dataset

Access to the full dataset will be limited to the researcher and the supervisors for this study. In line with Cancer Research UK (CRUK) policy, fully anonymised interview transcripts will potentially be made available to researchers for analysis in future related studies, subject to consent obtained from participants.

Any research nurses or fellows involved with recruitment will not have any access to the data collected.

8 DISSEMINIATION POLICY

8.1 Dissemination policy

The data arising from the study will be owned by the University of Exeter.

On completion of the study, data will be analysed and synthesised into a chapter for the PhD of the researcher, registered at the University of Exeter. Access to the full study report, including the protocol, will be made through the Open Research Exeter (ORE) online portal, hosted by the University of Exeter, after the thesis has been accepted by The University.

CRUK are the major funders of this study, through a Catalyst Award (‘CanTest’). CanTest funds the researcher’s salary, training, PhD fees and research costs. CRUK will be acknowledged in any publications associated with this study.

All participants will receive an abridged study report, outlining the major findings of the study.

8.2 Authorship eligibility guidelines and any intended use of professional writers

Authorship for the final study report and any publications associated with this study will be agreed in accordance with the International Committee of Medical Journal Editors guidance.

9 REFERENCES

1. Lijmer JG, Leeflang M, Bossuyt PMM. Proposals for a phased evaluation of medical tests. Med Decis Mak. 2009;29(5):13–21.

2. Fryback DG, Thornbury JR, Fryback DG, Thornbury JR. Medical Decision Making The Efficacy of Diagnostic Imaging. Med Decis Mak. 1991;11:88–94.

3. Horvath AR, Lord SJ, StJohn A, Sandberg S, Cobbaert CM, Lorenz S, et al. From biomarkers to medical tests: The changing landscape of test evaluation. Clin Chim Acta [Internet]. 2014;427:49–57. Available from: http://dx.doi.org/10.1016/j.cca.2013.09.018

4. Lin JS, Thompson M, Goddard KAB, Piper MA, Heneghan C, Whitlock EP. Evaluating genomic

For peer review only - http://bmjopen.bmj.com/site/about/guidelines.xhtml

For peer review only

tests from bench to bedside: A practical framework. BMC Med Inform Decis Mak [Internet]. 2012;12(1):1. Available from: BMC Medical Informatics and Decision Making

5. Thompson M, Weigl B, Fitzpatrick A, Ide N. More Than Just Accuracy: A Novel Method to Incorporate Multiple Test Attributes in Evaluating Diagnostic Tests Including Point of Care Tests. IEEE J Transl Eng Heal Med. 2016;4(January).

6. National Collaborating Centre for Cancer. Suspected cancer [Internet]. NICE. London; 2015. Available from: http://journals.rcni.com/doi/10.7748/ns.29.47.17.s20

7. Mottet N, Bellmunt J, Briers E, Bolla M, Bourke L, Cornford P, et al. EAU - ESTRO - ESUR - SIOG Guidelines on Prostate Cancer [Internet]. European Association of Urology; 2017. Available from: https://uroweb.org/wp-content/uploads/09-Prostate-Cancer_2017_web.pdf

8. Loeb S, Vellekoop A, Ahmed HU, Catto J, Emberton M, Nam R, et al. Systematic review of complications of prostate biopsy. Eur Urol [Internet]. 2013 Dec 1;64(6):876–92. Available from: http://dx.doi.org/10.1016/j.eururo.2013.05.049

9. Hassanzadeh E, Glazer DI, Dunne RM, Fennessy FM, Harisinghani MG, Tempany CM. Prostate imaging reporting and data system version 2 (PI-RADS v2): a pictorial review. Abdom Radiol (New York) [Internet]. 2017;42(1):278–89. Available from: http://www.ncbi.nlm.nih.gov/pubmed/27522352%0Ahttp://www.pubmedcentral.nih.gov/articlerender.fcgi?artid=PMC5247306%0Ahttp://www.ncbi.nlm.nih.gov/pubmed/27522352%5Cnhttp://www.pubmedcentral.nih.gov/articlerender.fcgi?artid=PMC5247306

10. Ahmed HU, Bosaily AE-S, Brown LC, Gabe R, Kaplan R, Parmar MK, et al. Diagnostic accuracy of multi-parametric MRI and TRUS biopsy in prostate cancer (PROMIS): a paired validating confirmatory study. Lancet [Internet]. 2017 Jan 19;380:1–8. Available from: http://dx.doi.org/10.1016/S0140-6736(16)32401-1

11. Kasivisvanathan V, Rannikko AS, Borghi M, Panebianco V, Mynderse LA, Vaarala MH, et al. MRI-Targeted or Standard Biopsy for Prostate-Cancer Diagnosis. N Engl J Med [Internet]. 2018; Available from: http://www.nejm.org/doi/10.1056/NEJMoa1801993

12. Bray F, Ferlay J, Soerjomataram I, Siegel R, Torre L, Jemal A. Global cancer statistics 2018: GLOBOCAN estimates of incidence and mortality worldwide for 36 cancers in 185 countries. CA A J Clin. 2018;00(00):1–31.

13. Office for National Statistics. Cancer registration statistics, England: first release, 2015. Statistical Bulletin. 2017.

14. Sandhu GS, Andriole GL. Overdiagnosis of prostate cancer. J Natl Cancer Inst - Monogr [Internet]. 2012 Dec 27;2012(45):146–51. Available from: http://jncimono.oxfordjournals.org/cgi/doi/10.1093/jncimonographs/lgs031

15. Boesen L, Norgaard N, Logager V, Balslev I, Thestrup K, Bisbjerg R, et al. Assessment of the Diagnostic Accuracy of Biparametric Magnetic Resonance Imaging for Prostate Cancer in Biopsy-Naive Men: The Biparametric MRI for Detection of Prostate Cancer (BIDOC) study. JAMA Netw Open. 2018;1(2).

16. Otti VC, Miller C, Powell RJ, Thomas RM, McGrath JS. The diagnostic accuracy of multiparametric MRI prior to biopsy in the detection of prostate cancer. BJU Int [Internet]. 2018; Available from: http://www.ncbi.nlm.nih.gov/pubmed/29804315%0Ahttp://doi.wiley.com/10.1111/bju.14420

17. NHS England. Implementing a timed prostate cancer diagnostic pathway. NHS; 2018.

18. Peters JL, Thompson AC, Mcnicholas TA, Hines JEW, Hanbury DC, Boustead GB. Increased patient satisfaction from transrectal ultrasonography and biopsy under sedation. BJU Int. 2001;87(9):827–30.

19. Makinen T, Auvinen A, Hakama M, Stenman UH, Tammela TLJ. Acceptability and

For peer review only

For peer review only - http://bmjopen.bmj.com/site/about/guidelines.xhtml

complications of prostate biopsy in population-based PSA screening versus routine clinical practice: A prospective, controlled study. Urology. 2002;60(5):846–50.

20. Clements R, Aideyan OU, Griffiths GJ, Peeling WB. Side effects and patient acceptability of transrectal biopsy of the prostate. Clin Radiol. 1993;47(2):125–6.

21. Egbers N, Schwenke C, Maxeiner A, Teichgräber U, Franie T. MRI-guided core needle biopsy of the prostate: Acceptance and side effects. Diagnostic Interv Radiol. 2015;21(3):215–21.

22. Ullrich T, Schimmöller L, Oymanns M, Blondin D, Dietzel F, Kirchner J, et al. Current Utilization and Acceptance of Multiparametric MRI in the Diagnosis of Prostate Cancer. A Regional Survey. RöFo - Fortschritte auf dem Gebiet der Röntgenstrahlen und der Bildgeb Verfahren [Internet]. 2017;419–26. Available from: http://www.thieme-connect.de/DOI/DOI?10.1055/s-0043-118128

23. Sekhon M, Cartwright M, Francis JJ. Acceptability of health care interventions: A theoretical framework and proposed research agenda. Br J Health Psychol. 2018;1–13.

24. Sekhon M, Cartwright M, Francis JJ. Acceptability of healthcare interventions: An overview of reviews and development of a theoretical framework. BMC Health Serv Res [Internet]. 2017;17(1):1–13. Available from: http://dx.doi.org/10.1186/s12913-017-2031-8

25. Strauss A, Corbin J. Basics of qualitative research. Sage; 1990.

26. Silverman D. Doing qualitative research. Sage; 2013.

27. Ahmed HU, El-Shater Bosaily A, Brown LC, Gabe R, Kaplan R, Parmar MK, et al. Diagnostic accuracy of multi-parametric MRI and TRUS biopsy in prostate cancer (PROMIS): a paired validating confirmatory study. Lancet [Internet]. 2017;389(10071):815–22. Available from: http://dx.doi.org/10.1016/S0140-6736(16)32401-1

28. Panebianco V, Barchetti G, Simone G, Del Monte M, Ciardi A, Grompone MD, et al. Negative Multiparametric Magnetic Resonance Imaging for Prostate Cancer: What's Next? Eur Urol [Internet]. 2018;74(1):48–54. Available from: https://doi.org/10.1016/j.eururo.2018.03.007

29. Ritchie J, Spencer EA. Qualitative data analysis for applied policy research. In: Analyzing Qualitative Data. 1st ed. London: Routledge; 1994. p. 173–94.

30. Gale NK, Heath G, Cameron E, Rashid S, Redwood S. Using the framework method for the analysis of qualitative data in multi-disciplinary health research. BMC Med Res Methodol [Internet]. 2013;13(1):1. Available from: BMC Medical Research Methodology

31. Birt L, Hall N, Emery J, Banks J, Mills K, Johnson M, et al. Responding to symptoms suggestive of lung cancer: A qualitative interview study. BMJ Open Respir Res. 2014;1(1).

32. Hall N, Birt L, Banks J, Emery J, Mills K, Johnson M, et al. Symptom appraisal and healthcare-seeking for symptoms suggestive of colorectal cancer: A qualitative study. BMJ Open. 2015;5(10).

33. Walter FM, Birt L, Cavers D, Scott S, Emery J, Burrows N, et al. "This isn't what mine looked like": A qualitative study of symptom appraisal and help seeking in people recently diagnosed with melanoma. BMJ Open. 2014;4(7):1–12.

For peer review only

For peer review only - http://bmjopen.bmj.com/site/about/guidelines.xhtml

10. APPENDICIES

10.1 Amendment History

Amendment No.	Protocol version no.	Date issued	Author(s) of changes	Details of changes made

For peer review only

For peer review only - http://bmjopen.bmj.com/site/about/guidelines.xhtml

Consolidated criteria for reporting qualitative studies (COREQ): 32-item checklist

Developed from:
Tong A, Sainsbury P, Craig J. Consolidated criteria for reporting qualitative research (COREQ): a 32-item checklist for interviews and focus groups. *International Journal for Quality in Health Care*. 2007. Volume 19, Number 6: pp. 349 – 357

No. Item	Guide questions/description	Reported on Page #
**Domain 1: Research team and reflexivity**		
Personal Characteristics		
1. Inter viewer/facilitator	Which author/s conducted the interview or focus group?	8
2. Credentials	What were the researcher's credentials? E.g. PhD, MD	1-2
3. Occupation	What was their occupation at the time of the study?	8
4. Gender	Was the researcher male or female?	8
5. Experience and training	What experience or training did the researcher have?	8
Relationship with participants		
6. Relationship established	Was a relationship established prior to study commencement?	8
7. Participant knowledge of the interviewer	What did the participants know about the researcher? e.g. personal goals, reasons for doing the research	19
8. Interviewer characteristics	What characteristics were reported about the inter viewer/facilitator? e.g. Bias, assumptions, reasons and interests in the research topic	19
**Domain 2: study design**		
Theoretical framework		
9. Methodological orientation and Theory	What methodological orientation was stated to underpin the study? e.g. grounded theory, discourse analysis, ethnography, phenomenology, content analysis	7
Participant selection		
10. Sampling	How were participants selected? e.g. purposive, convenience, consecutive, snowball	7
11. Method of approach	How were participants approached? e.g. face-to-face, telephone, mail, email	7-8
12. Sample size	How many participants were in the study?	11
13. Non-participation	How many people refused to participate or dropped out? Reasons?	11

For peer review only

Setting		
14. Setting of data collection	Where was the data collected? e.g. home, clinic, workplace	8
15. Presence of non-participants	Was anyone else present besides the participants and researchers?	11
16. Description of sample	What are the important characteristics of the sample? e.g. demographic data, date	11
Data collection		
17. Interview guide	Were questions, prompts, guides provided by the authors? Was it pilot tested?	8
18. Repeat interviews	Were repeat inter views carried out? If yes, how many?	9
19. Audio/visual recording	Did the research use audio or visual recording to collect the data?	9
20. Field notes	Were field notes made during and/or after the inter view or focus group?	9
21. Duration	What was the duration of the inter views or focus group?	9
22. Data saturation	Was data saturation discussed?	9
23. Transcripts returned	Were transcripts returned to participants for comment and/or correction?	9
**Domain 3: analysis and findings**		
Data analysis		
24. Number of data coders	How many data coders coded the data?	9
25. Description of the coding tree	Did authors provide a description of the coding tree?	9
26. Derivation of themes	Were themes identified in advance or derived from the data?	9
27. Software	What software, if applicable, was used to manage the data?	NVivo
28. Participant checking	Did participants provide feedback on the findings?	9
Reporting		
29. Quotations presented	Were participant quotations presented to illustrate the themes/findings? Was each quotation identified? e.g. participant number	12-18
30. Data and findings consistent	Was there consistency between the data presented and the findings?	12-18
31. Clarity of major themes	Were major themes clearly presented in the findings?	12-18
32. Clarity of minor themes	Is there a description of diverse cases or discussion of minor themes?	12-18

For peer review only
